# Sequence and structure of *Brassica rapa *chromosome A3

**DOI:** 10.1186/gb-2010-11-9-r94

**Published:** 2010-09-27

**Authors:** Jeong-Hwan Mun, Soo-Jin Kwon, Young-Joo Seol, Jin A Kim, Mina Jin, Jung Sun Kim, Myung-Ho Lim, Soo-In Lee, Joon Ki Hong, Tae-Ho Park, Sang-Choon Lee, Beom-Jin Kim, Mi-Suk Seo, Seunghoon Baek, Min-Jee Lee, Ja Young Shin, Jang-Ho Hahn, Yoon-Jung Hwang, Ki-Byung Lim, Jee Young Park, Jonghoon Lee, Tae-Jin Yang, Hee-Ju Yu, Ik-Young Choi, Beom-Soon Choi, Su Ryun Choi, Nirala Ramchiary, Yong Pyo Lim, Fiona Fraser, Nizar Drou, Eleni Soumpourou, Martin Trick, Ian Bancroft, Andrew G Sharpe, Isobel AP Parkin, Jacqueline Batley, Dave Edwards, Beom-Seok Park

**Affiliations:** 1 Department of Agricultural Biotechnology, National Academy of Agricultural Science, Rural Development Administration, 150 Suin-ro, Gwonseon-gu, Suwon 441-707, Korea; 2 Department of Horticulture, College of Agriculture and Life Science, Kyungpook National University, 1370 Sangyeok-dong, Buk-gu, Daegu 702-701, Korea; 3 Department of Plant Science, Plant Genomics and Breeding Institute, and Research Institute for Agriculture and Life Sciences, College of Agriculture and Life Sciences, Seoul National University, 599 Gwanak-ro, Gwanak-gu, Seoul 151-921, Korea; 4 Department of Life Sciences, The Catholic University of Korea, 43-1 Yeokgok 2-dong, Wonmi-gu, Bucheon 420-743, Korea; 5 National Instrumentation Center for Environmental Management, Seoul National University, 599 Gwanak-ro, Gwanak-gu, Seoul 151-921, Korea; 6 Department of Horticulture, Chungnam National University, 220 Kung-dong, Yusong-gu, Daejon 305-764, Korea; 7 John Innes Centre, Colney, Norwich NR4 7UH, UK; 8 NRC Plant Biotechnology Institute, 110 Gymnasium Place, Saskatoon, SK S7N 0W9, Canada; 9 Agriculture and Agri-Food Canada, Saskatoon Research Centre, Saskatoon, SK S7N OX2, Canada; 10 ARC Centre of Excellence for Integrative Legume Research and School of Land, Crop and Food Sciences, University of Queensland, Brisbane, QLD 4067, Australia; 11 Australian Centre for Plant Functional Genomics and School of Land Crop and Food Sciences, University of Queensland, Brisbane, QLD 4067, Australia

## Abstract

**Background:**

The species *Brassica rapa *includes important vegetable and oil crops. It also serves as an excellent model system to study polyploidy-related genome evolution because of its paleohexaploid ancestry and its close evolutionary relationships with *Arabidopsis thaliana *and other *Brassica *species with larger genomes. Therefore, its genome sequence will be used to accelerate both basic research on genome evolution and applied research across the cultivated *Brassica *species.

**Results:**

We have determined and analyzed the sequence of *B. rapa *chromosome A3. We obtained 31.9 Mb of sequences, organized into nine contigs, which incorporated 348 overlapping BAC clones. Annotation revealed 7,058 protein-coding genes, with an average gene density of 4.6 kb per gene. Analysis of chromosome collinearity with the *A. thaliana *genome identified conserved synteny blocks encompassing the whole of the *B. rapa *chromosome A3 and sections of four *A. thaliana *chromosomes. The frequency of tandem duplication of genes differed between the conserved genome segments in *B. rapa *and *A. thaliana*, indicating differential rates of occurrence/retention of such duplicate copies of genes. Analysis of 'ancestral karyotype' genome building blocks enabled the development of a hypothetical model for the derivation of the *B. rapa *chromosome A3.

**Conclusions:**

We report the near-complete chromosome sequence from a dicotyledonous crop species. This provides an example of the complexity of genome evolution following polyploidy. The high degree of contiguity afforded by the clone-by-clone approach provides a benchmark for the performance of whole genome shotgun approaches presently being applied in *B. rapa *and other species with complex genomes.

## Background

The Brassicaceae family includes approximately 3,700 species in 338 genera. The species, which include the widely studied *Arabidopsis thaliana*, have diverse characteristics and many are of agronomic importance as vegetables, condiments, fodder, and oil crops [1]. Economically, *Brassica *species contribute to approximately 10% of the world's vegetable crop produce and approximately 12% of the worldwide edible oil supplies [2]. The tribe Brassiceae, which is one of 25 tribes in the Brassicaceae, consists of approximately 240 species and contains the genus *Brassica*. The cultivated *Brassica *species are *B. rapa *(which contains the *Brassica *A genome) and *B. oleracea *(C genome), which are grown mostly as vegetable cole crops, *B. nigra *(B genome) as a source of mustard condiment, and oil crops, mainly *B. napus *(a recently formed allotetraploid containing both A and C genomes), *B. juncea *(A and B genomes), and *B. carinata *(B and C genomes) as sources of canola oil. These genome relationships between the three diploid species and their pairwise allopolyploid derivative species have long been known, and are described by 'U's triangle' [3].

*B. rapa *is a major vegetable or oil crop in Asia and Europe, and has recently become a widely used model for the study of polyploid genome structure and evolution because it has the smallest genome (529 Mb) of the *Brassica *genus and, like all members of the tribe Brassiceae, has evolved from a hexaploid ancestor [4-6]. Our previous comparative genomic study revealed conserved linkage arrangements and collinear chromosome segments between *B. rapa *and *A. thaliana*, which diverged from a common ancestor approximately 13 to 17 million years ago. The *B. rapa *genome contains triplicated homoeologous counterparts of the corresponding segments of the *A. thaliana *genome due to triplication of the entire genome (whole genome triplication), which occurred approximately 11 to 12 million years ago [6]. Furthermore, studies in *B. napus*, which was generated in the last 10,000 years, have demonstrated that overall genome structure is highly conserved compared to its progenitor species, *B. rapa *and *B. oleracea*, which diverged approximately 8 million years ago, but significantly diverged relative to *A. thaliana *at the sequence level [7,8]. Thus, investigation of the *B. rapa *genome provides substantial opportunities to study the divergence of gene function and genome evolution associated with polyploidy, extensive duplication, and hybridization. In addition, access to a complete and high-resolution *B. rapa *genome will facilitate research on other *Brassica *crops with partially sequenced or larger genomes.

Despite the importance of *Brassica *crops in plant biology and world agriculture, none of the *Brassica *species have had their genomes fully sequenced. Cytogenetic analyses have showed that the *B. rapa *genome is organized into ten chromosomes, with genes concentrated in the euchromatic space and centromeric repeat sequences and rDNAs arranged as tandem arrays primarily in the heterochromatin [9,10]. The individual mitotic metaphase chromosome size ranges from 2.1 to 5.6 μm, with a total chromosome length of 32.5 μm [9]. An alternative cytogenetic map based on a pachytene DAPI (4',6-diamidino-2-phenylindole dihydrochloride) and fluorescent *in situ *hybridization (FISH) karyogram showed that the mean lengths of ten pachytene chromosomes ranged from 23.7 to 51.3 μm, with a total chromosome length of 385.3 μm [11]. Thus, chromosomes in the meiotic prophase stage are 12 times longer than those in the mitotic metaphase, and display a well-differentiated pattern of bright fluorescent heterochromatin segments. Sequencing of selected BAC clones has confirmed that the gene density in *B. rapa *is similar to that of *A. thaliana *in the order of 1 gene per 3 to 4 kb [6]. Each of the gene-rich BAC clones examined so far by FISH (> 100 BACs) was found to be localized to the visible euchromatic region of the genome. Concurrently, a whole-genome shotgun pilot sequencing of *B. oleracea *with 0.44-fold genome coverage generated sequences enriched in transposable elements [12,13]. Taken together, these data strongly point to a tractable genome organization where the majority of the *B. rapa *euchromatic space (gene space) can be sequenced in a highly efficient manner by a clone-by-clone strategy. Based on these results, the multinational *Brassica rapa *Genome Sequencing Project (BrGSP) was launched, with the aim of sequencing the euchromatic arms of all ten chromosomes [14]. The project aimed to initially produce a 'phase 2 (fully oriented and ordered sequence with some small gaps and low quality sequences)' sequence with accessible trace files by shotgun sequencing of clones so that researchers who require complete sequences from a specific region can finish them.

To support genome sequencing, five large-insert BAC libraries of *B. rapa *ssp. *pekinensis *cv. *Chiifu *were constructed, providing approximately 53-fold genome coverage overall [15]. These libraries were constructed using several different restriction endonucleases to cleave genomic DNA (*Eco*RI, *Bam*HI, *Hin*dIII, and *Sau*3AI). Using these BAC libraries, a total of 260,637 BAC-end sequences (BESs) have been generated from 146,688 BAC clones (approximately 203 Mb) as a collaborative outcome of the multinational BrGSP community. The strategy for clone-by-clone sequencing was to start from defined and genetically/cytogenetically mapped seed BACs and build outward. Initially, a comparative tiling method of mapping BES onto the *A. thaliana *genome, combined with fingerprint-based physical mapping, along with existing genetic anchoring data provided the basis for selecting seed BAC clones and for creating a draft tiling path [6,16,17]. As a result, 589 BAC clones were sequenced and provided to the BrGSP as 'seed' BACs for chromosome sequencing. Integration of seed BACs with the physical map provided 'gene-rich' contigs spanning approximately 160 Mb. These 'gene-rich' contigs enabled the selection of clones to extend the initial sequence contigs. Here, as the first report of the BrGSP, we describe a detailed analysis of *B. rapa *chromosome A3, the largest of the ten *B. rapa *chromosomes, as assessed by both cytogenetic analysis and linkage mapping (length estimated as 140.7 cM). The A3 linkage group also contains numerous collinearity discontinuities (CDs) compared with *A. thaliana*, a recent study into which [18] revealed greater complexity than originally described for the segmental collinearity of *Brassica *and *Arabidopsis *genomes [19,20]. In accordance with the agreed standards of the BrGSP, we aimed to generate phase 2 contiguous sequences for *B. rapa *chromosome A3. We annotated these sequences for genes and other characteristics, and used the data to analyze genome composition and examine consequential features of polyploidy, such as genome rearrangement.

## Results and discussion

### General features of chromosome A3

**Figure 1:**
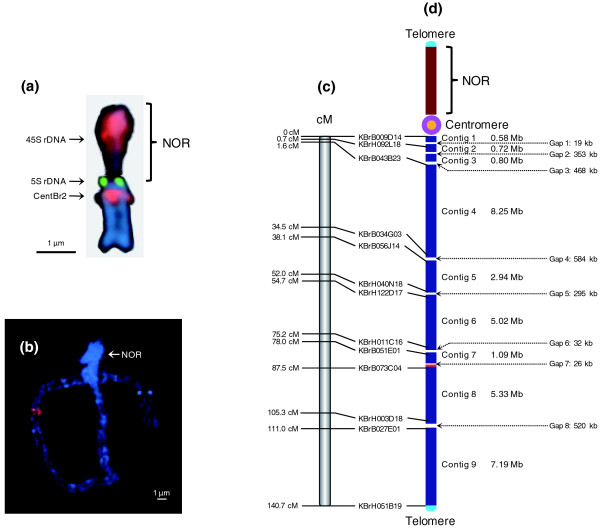
**Features of *B. rapa *chromosome A3**. (a) Mitotic metaphase structure of chromosome A3 with FISH signals of 45S (red), 5S (green) rDNAs, and CentBr2 (magenta). **(b) **Image of DAPI-stained pachytene spread of chromosome A3 showing the heterochromatic NORs of the short arm (bright blue) and euchromatic long arm (blue). **(c) ***VCS *(cv. *VC1 *ⅹ cv. *SR5*) genetic map showing the positions of the BAC clones found nearest the end of each contig. **(d) **Physical map showing the location of nine sequence contigs (blue). The chromosome is roughly 34.2 Mb long, spans a genetic map distance of 140.7 cM with 243 kb/cM, and contains 6.4% of the unique sequence of the *B. rapa *genome. The centromere is shown as a pink circle, the NOR of the rDNA repeat region in the short arm is represented as a brown bar, and telomeres are light blue. The telomere, centromere, and NOR are not drown to scale. The sizes of eight unsequenced gaps measured by pachytene FISH are given in kilobases. Red areas in (b, d) point to the position of the hybridization signal of KBrH34P23 in sequence contig 8.

Chromosome A3 is acrocentric, with a heterochromatic upper (short) arm bearing the nucleolar organizer region (NOR) and a euchromatic lower (long) arm (Figure 1a). The NOR comprises a large domain of 45S rDNA repeats and a small fraction of 5S rDNA repeats extending to the centromere. The centromere of chromosome A3 is typically characterized by hybridization of the 176-bp centromeric tandem repeat CentBr2, which resides on only chromosomes A3 and A5 [10]. The euchromatic region of chromosome A3, the lower arm, has been measured as 45.5 μm in pachytene FISH (Figure 1b). The sequence length of the lower arm from centromere to telomere was estimated to be approximately 34 to 35 Mb based on measurement of the average physical length of sequenced contigs (1 μm/755 kb). Chromosome sequencing was initiated using BAC clones that had been anchored onto the lower arm of chromosome A3 by genetic markers. Subsequently, BES and physical mapping of chromosome A3 allowed extension from these initial seed points and completion of the entire lower arm. However, no BAC clones were identified from the upper arm, possibly owing to the lack of appropriate restriction enzyme sites in these regions, the instability of the sequences in *Escherichia coli *or a complete lack of euchromatic sequences on that arm.

A total of 348 BAC clones were sequenced from the lower arm of chromosome A3 to produce 31.9 Mb of sequences of phase 2 or phase 3 (finished sequences) standard. These were assembled into nine contigs that span 140.7 cM of the genetic map (Figures 1c, d; Figure S1 in Additional file 1). The lower arm sequence starts at the proximal clone KBrH044B01 and terminates at the distal clone KBrF203I22 (Table S1 in Additional file 2). Excluding the gaps at the centromere and telomere, the pachytene spread FISH indicated that eight physical gaps, totaling approximately 2.3 Mb, remain on the pseudochromosome sequence. Despite extensive efforts, no BACs could be identified in those regions. The total length of the lower arm, from centromere to telomere, was therefore calculated to be 34.2 Mb. Thus, the 31.9 Mb of sequences we obtained represents 93% of the lower arm of the chromosome. The sequence and annotation of *B. rapa *chromosome A3 can be found in GenBank (see Materials and methods).

### Characterization of the sequences

**Figure 2:**
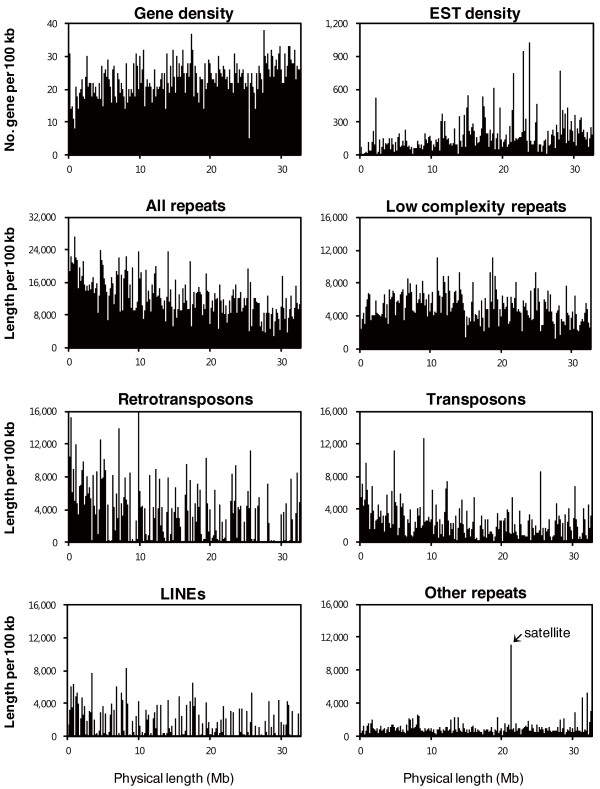
**Distribution of various repeats and features on chromosome A3**. The long arm of chromosome A3 is shown on the *x-*axis and is numbered from the beginning of contig 1 to the end of contig 9 by joining up the physical gaps. The *y-*axis represents genes, ESTs, and the various repeats plotted relative to the nucleotide position on the chromosome. The densities of genes, ESTs, and the repeats were obtained by analyzing the sequence every 100 kb using a 10-kb sliding window. LINE, long interspersed nuclear element.

The distribution of genes and various repetitive DNA elements along chromosome A3 are depicted in Figure 2, with details of the content of repetitive sequences provided in Table S2 in Additional file 2. Overall, 11% of the sequenced region in chromosome A3 is composed of repetitive sequences, which are dispersed over the lower arm. The distribution of repetitive sequences along the chromosome was not even, with fewer retrotransposons (long terminal repeats) and DNA transposons towards the distal end. In addition, low complexity repetitive sequences are relatively abundant in the lower arm, indicating *B. rapa*-specific expansion of repetitive sequences. These are the most frequently occurring class of repetitive elements, accounting for 41% of the total amount of repetitive sequence elements. Other types of repeat do not show obvious clustering except satellite sequences around 22 Mb from the centromere. These sequences have high sequence similarity to a 350-bp AT-rich tandem repeat of *B. nigra *[21].

Gene structure and density statistics are shown in Table 1. The overall G+C content of chromosome A3 is 33.8%, which is less than was reported for the euchromatic seed BAC sequences (35.2%) [6] and the entire *A. thaliana *genome (35.9%) [22]. Gene annotation was carried out using our specialized *B. rapa *annotation pipeline. This modeled a total of 7,058 protein-coding genes, of which 1,550 have just a single exon. On average, each gene model contains 4.7 exons and is 1,755 bp in length. Consistent with the results of more restricted studies [6], the average length of gene models annotated on chromosome A3 is shorter than those of *A. thaliana *genes due to reduction in both exon number per gene and exon length. The average gene density is 4,633 bp per gene, which is also lower than in *A. thaliana *(4,351 bp per gene), indicating a slightly less compact genome organization. The longest gene model, which is predicted to encode a potassium ion transmembrane transporter, consists of 8 exons across 31,311 bp.

**Table 1:** Statistics of *B. rapa *chromosome A3

	*B. rapa *chromosome A3	*A. thaliana *whole genome
Total number of BACs	348	1,633
Approximate chromosome length (Mb)	34.2	134.6
Total non-overlapping sequence (Mb)	31.9	119.1
G/C content (%)		
Overall	33.8	35.9
Exons	46.4	44.1
Introns	32.4	32.6
Intergenic regions	29.6	32.9
Number of protein coding genes	7,058	27,379
Number of exons per gene	4.7	5.7
Intron size (bp)	170	165
Exon size (bp)	222	304
Average gene size (bp)	1,755	2,467
Average gene density (bp/gene)	4,633	4,351
Alternatively spliced genes	184	4,626
Known genes	5,825	21,498
Average known gene size (bp)	1,231	2,384
Unknown genes	1,415	5,784
Average unknown gene size (bp)	547	1,489
Hypothetical genes	2	97
Average hypothetical gene size (bp)	1,681	686
tRNA genes	164	689
miRNA genes	26	215
Transposons (%)	5	13

The *B. rapa *chromosome A3 statistics were generated in this study. The *Arabidopsis *genome features are from The *Arabidopsis *Information Resource database (release TAIR9) [23].

Potential alternative splicing variants, based upon a minimum requirement for three EST matches, was identified for only 2.3% of the gene models. This finding suggests that alternative splicing may be rarer in *B. rapa *than it is in *A. thaliana*, where it occurs at a frequency of 16.9% [23]. Additional EST data will enable more precise identification of alternative spliced variants on the *B. rapa *genome.

**Figure 3:**
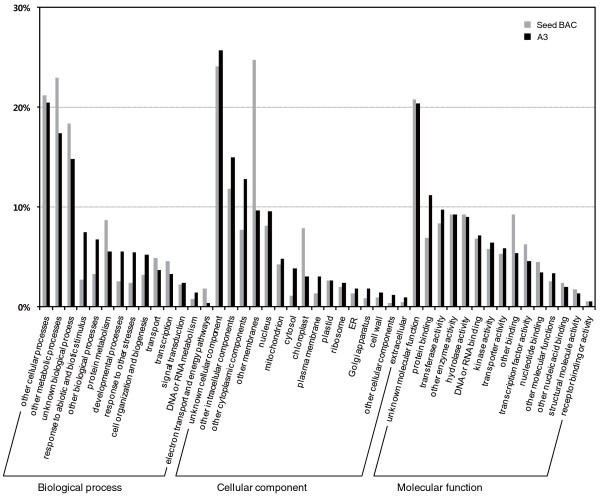
**Functional classification of the proteins encoded on chromosome A3 or seed BAC sequences through annotation using Gene Ontology**. Assignments are based on the annotations to terms in the GO biological process, cellular component, and molecular function categories.

We identified 5,825 genes as 'known' based upon EST matches, protein matches, or any detectable domain signatures. The remaining 1,417 predicted genes were assigned as 'unknown' or 'hypothetical'. The functions of 'known' genes were classified according to Gene Ontology (GO) analysis (Figure 3). We compared the results of GO-based classification of gene models from chromosome A3 with a similar analysis of gene models from the 65.8 Mb of genome-wide seed BAC sequences [6]. This revealed several categories for which the functional complement of genes on chromosome A3 is atypical of the genome as a whole. For example, it has higher proportions of genes classified as related to 'stress' or 'developmental process' under the GO biological process category compared to the collection of seed BAC sequences (*P *< 0.0001). In addition, there are differences in terms pertaining to membrane related genes and chloroplast of the GO cellular component category between the two data sets (*P *< 0.2).

**Figure 4:**
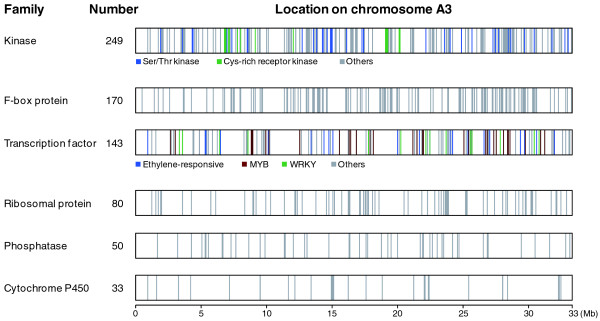
**Distribution patterns of the top six gene categories on chromosome A3**. Width of the vertical bars is proportional to the number of genes located at that position.

The predicted proteins found on chromosome A3 were categorized into gene families by BLASTP (using a minimum threshold of 50% alignment coverage at a cutoff of E^-10^). The chromosome contains 384 families of tandemly duplicated genes with 1,262 members, comprising 17.9% of all genes (Figure S2 in Additional file 1). This is lower than found in *A. thaliana*, which has 27% of genes existing as tandem duplicates in the genome. The most abundant gene family was the protein kinase family, with 249 members, followed by F-box proteins (170 members) and transcription factors (143 members). These families are distributed throughout the chromosome (Figure 4). The highest number of tandem duplicates detected at a single site was a cluster of 18 copies of the cysteine-rich receptor-like protein kinase gene family, located around coordinate 7 Mb.

The chromosome contains 164 tRNAs and 3 small nuclear RNAs. The tRNAs are evenly distributed along chromosome A3 except for one region where they cluster. This cluster, at 23.9 Mb, contains 12 tandem tRNA^Pro ^genes, which are the most abundant tRNA genes on the chromosome (Figure S3 in Additional file 1). A tRNA^Pro ^cluster was previously detected also on *A. thaliana *chromosome 1 [24]. A computational search coupled with prediction of secondary structure using reported mature microRNA (miRNA) sequences identified 26 miRNA genes, which outnumber the total number of *B. rapa *(17) recorded in miRBase (release 15.0; April 2010; Table S3 in Additional file 2). Abundant miRNAs on chromosome A3 included miR2111 and miR399. These have been implicated in regulating nutritional balance in *B. rapa *based upon observation of their induction during phosphate limitation in *A. thaliana *and rapeseed [25,26].

A sequence similarity search showed that 2.5% of the genes identified on chromosome A3 are of mitochondrial (98 genes) or chloroplast (78 genes) origin. The widespread distribution observed for organellar insertions across the chromosome indicates that mitochondrial and chloroplast gene transfer occurred independently.

### Synteny between chromosome A3 and the *A. thaliana *genome

**Figure 5:**
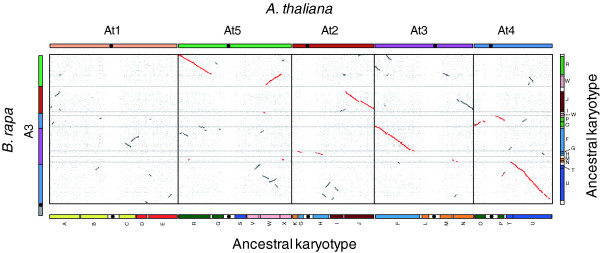
**Synteny between *B. rapa *chromosome A3 and the *A. thaliana *genome**. Chromosome correspondence between the genomes is represented by a dot-plot. Each dot represents a reciprocal best BLASTP match between gene pairs at an E value cutoff of < E^-20^. Red dots show regions of synteny with more than 50% gene conservation as identified by DiagHunter. Color bars on the upper and left margins of the dot plot indicate individual chromosomes of *A. thaliana *and *B. rapa*, respectively, demonstrating corresponding similarity. Black dots on the chromosomes are centromeres. Color bars on the bottom and right margins of the dot plot show ancestral karyotype genome building blocks mapped on the reduced karyotypes of *A. thaliana *and *B. rapa*, respectively. Bars of the same color are putative homologous counterparts.

To investigate detailed syntenic relationships between chromosome A3 and the five chromosomes of *A. thaliana*, we compared the proteomes predicted from the two genomes using BLASTP analysis (Table S4 in Additional file 2). Approximately 75.4% of the genes of chromosome A3 have similarity to genes in the *A. thaliana *genome. Figure 5 represents a dot matrix plot showing the large-scale blocks of collinearity between the two genomes. The collinearity blocks, identified by the red dots, extend the whole length of chromosome A3 and correspond to parts of four *A. thaliana *chromosomes (2, 3, 4, and 5) in a mosaic pattern. The collinearity blocks contain 6,551 gene models in *B. rapa *and 12,783 gene models in *A. thaliana*. Comparative analysis showed that 79.7% of gene models on chromosome A3 show similarity with counterparts in the collinear *A. thaliana *genome segments, whereas only 32.4% of *A. thaliana *genes show similarity with counterparts on chromosome A3. This is indicative of extensive and interspersed gene loss from *B. rapa *since divergence of the *Brassica *and *Arabidopsis *lineages, as described previously [5,27,28]. We found little evidence to support the presence of paralogous segments on chromosome A3 using self-syntenic comparison (Figure S4 in Additional file 1).

### Recombination and evolution of chromosome A3

**Figure 6:**
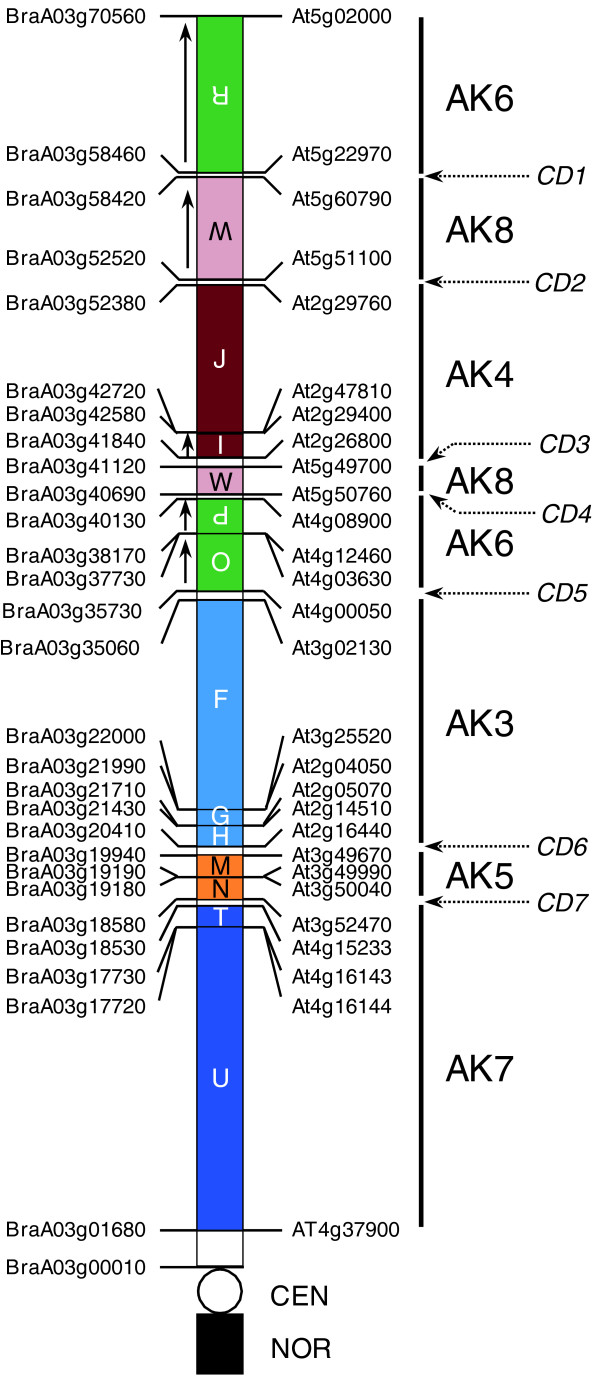
**Genome building blocks and block boundaries of the ancestral karyotype mapped onto *B. rapa *chromosome A3**. The position of AK genome building blocks in chromosome A3 was defined by a comparison of *B. rapa-A. thaliana *syntenic relationships and the *A. thaliana*-AK mapping results [20,29]. AK segments are labeled and oriented by arrows. Putative orthologs delineating the boundaries of recombination events are designated. CDs between AK blocks are indicated by dotted arrows. CEN, centromere.

Comparison of chromosome sequences between *B. rapa *chromosome A3 and *A. thaliana *allows complete mapping of the inferred ancient karyotype (AK) genome building blocks. According to genome mapping of AK blocks on the *A. thaliana *genome [20,29] and pairwise information for chromosome A3 and *A. thaliana *genome collinearity blocks, we defined conserved AK genome building blocks with pairwise boundary delineations of each block on the two genomes (Figure 6; Table S4 in Additional file 2). The order and boundaries of AK blocks on chromosome A3 were fundamentally similar to those of our previous report using seed BAC sequences [6]. Chromosome A3 is highly rearranged relative to *A. thaliana *chromosomes and compared with the AK. Overall, 14 blocks derived from 6 AK chromosomes (AK3, AK4, AK5, AK6, AK7, and AK8) were aligned with chromosome A3. All the AK blocks on chromosome A3 were shorter than those on the *A. thaliana *genome and seven CD regions were found between the blocks, suggesting that a complicated recombination of six AK chromosomes resulted in the emergence of chromosome A3.

**Figure 7:**
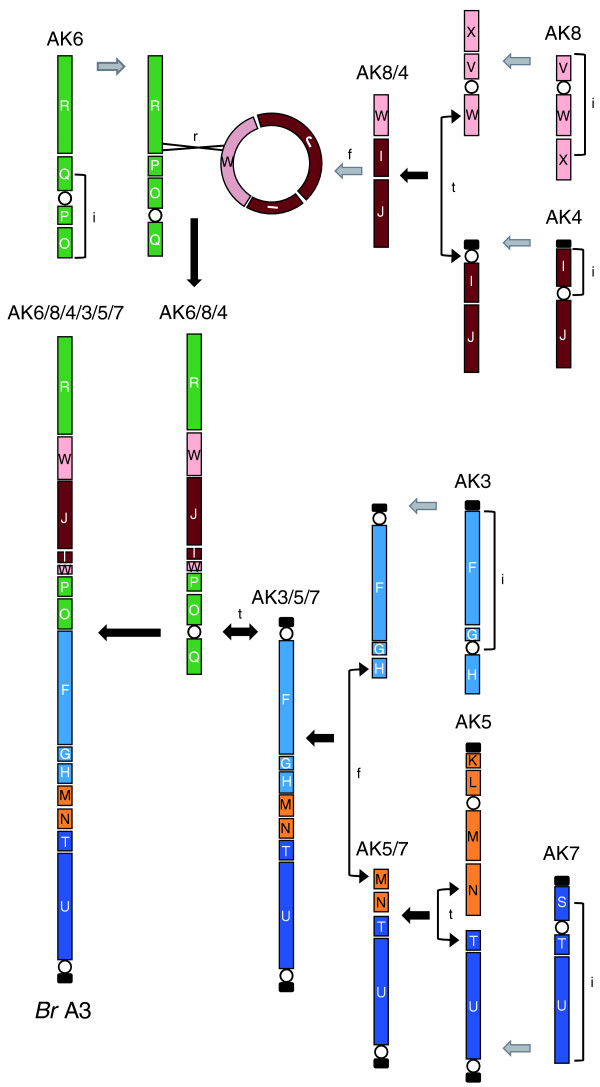
**Hypothetical derivation of chromosome A3**. Chromosome A3 has originated due to inversion (i), translocation (t), fusion (f), and recombination (r) of six AK chromosomes (AK3, AK4, AK5, AK6, AK7, and AK8). The ancestral chromosomes are presumed to bear NORs (black rectangles) and centromeres are represented as empty spheres. The minichromosomes consisting of a NOR and a centromere that resulted from translocation events have presumably been lost.

The combined analysis of AK mapping and identification of CDs on chromosome A3 enable us to hypothesize how parts of this chromosome have evolved from the AK. One hypothetical model for the reconstruction of the chromosome from the AK is presented in Figure 7. Chromosome A3 appears to have been derived from at least six AK chromosomes that were recombined in the progenitor of *B. rapa *by genome rearrangements, including inversion, translocation, fusion, and recombination. The detection of sequences from the W block of AK8 at both ends of the AK4 block indicates that there might have been a circular intermediate derived from fusion chromosome AK8/4 that was then integrated into AK6. Rearrangement of the AK seems to have taken place in the *B. rapa *genome after whole genome triplication, as none of the other chromosomes in the *B. rapa *genome show a similar arrangement of AK blocks. Furthermore, this study suggests that rearrangement events were involved in reduction of the basic chromosome number of *B. rapa *to ten. It remains uncertain, however, which group of linked events occurred earlier or later because multiple rounds of polyploidy followed by complex genome recombination yielded the current chromosome structure of *B. rapa*.

## Conclusions

Polyploid ancestry greatly complicates efforts to sequence genomes because of the presence of related sequences. Nevertheless, we have successfully sequenced, almost in its entirety, the largest chromosome of *B. rapa*, A3, using a clone-by-clone strategy. Annotation of the 31.9 Mb of sequences representing the gene space of chromosome A3 resulted in the development of models for 7,058 protein-coding genes and revealed the gene density to be only slightly lower than that observed for the related species *A. thaliana*, which is considered to have an exceptionally compact genome [22]. Comparative analysis of collinear genome segments with *A. thaliana *revealed extensive chromosome-wide interspersed gene loss from *B. rapa *since divergence of the *Brassica *and *Arabidopsis *lineages, as described previously only for small genomic regions [5,27,28]. The alignment of genome segments that the whole chromosome sequence permitted, relative to both the *A. thaliana *genome and the inferred AK of a common progenitor of *Brassica *and *Arabidopsis*, enabled the development of a model for the derivation of chromosome A3. The results confirm that the complete genome sequence of *B. rapa*, provided that it is of an appropriate standard, will have a major impact on comparative genomics and gene discovery in *Brassica *species.

## Materials and methods

### Chromosome sequencing

The *B. rapa *chromosome A3 was sequenced using a clone-by-clone sequencing strategy with a BAC-based physical map framework that was genetically anchored to the *B. rapa *genome [16]. We sequenced chromosome A3 of *B. rapa *ssp. *pekinensis *cultivar *Chiifu *from 348 overlapping BAC clones. Initially, we isolated seed BAC clones using a comparative BES tiling method and sequenced them by shotgun sequencing [6]. Seed BAC clones were then extended in both directions by searching for sequence identity in the BES database, which was then cross-examined with a physical map constructed using the KBrH, KBrB, and KBrS1 BAC libraries [16]. We also used KBrE and KBrS2 BAC libraries for additional extension and gap filling in particular. We carried out shotgun sequencing of the BAC clones to generate sequence data with eight- to ten-fold coverage of each clone using the ABI3730×l sequencer (Applied Biosystems, Foster City, CA, USA). According to the BrGSP [30], the minimal sequence goal was five phase 2 contigs. Individual BACs were assembled from the shotgun sequences using the PHRED/PHRAP [31,32] and the Consed [33] programs. The sequence contig assembly was created based on overlapping sequences using Sequencher (Gene Codes, Ann Arbor, MI, USA) program. To evaluate the accuracy of the assembly, alignment of EST unigenes, PCR amplification of the assembled sequences, and sequence comparison with fosmid clone links were performed. Contigs were ordered using sequence tagged site markers mapping to the long arm of the chromosome using *VCS *and *Jangwon *linkage maps [15], followed by estimation of non-overlapping gaps between contigs based on the results of FISH experiments. Pseudochromosome sequences were created by connecting sequence contigs with addition of filler sequences according to the estimated gap size; 10 k addition for gap sizes < 100 kb or 100 k addition for gap sizes > 100 kb. All the sequence information has been deposited in the National Center for Biotechnology and Information (NCBI) with accession numbers [NCBI:AC189184] to [NCBI:AC241201] (Table S1 in Additional file 2).

### Sequence annotation

We carried out gene prediction using our in-house automated gene prediction system [6]. The assembled sequences were masked using RepeatMasker [34] based on a dataset combining the plant repeat element database of The Institute for Genomic Research [35], Munich Information Center for Protein Sequences [36], and our specialized database of *B. rapa *repetitive sequences. Gene model prediction was performed using EVidenceModeler [37]. Putative exons and open reading frames (ORFs) were predicted *ab initio *using FGENESH [38], AUGUSTUS [39], GlimmerHMM [40], and SNAP [41] programs with the parameters trained using the *B. rapa *matrix. Putative gene splits predicted on the unfinished gaps were removed. To predict consensus gene structures, 152,253 *B. rapa *ESTs plus full-length cDNAs we have generated, *A. thaliana *coding sequences (release TAIR9), plant transcripts, and plant protein sequences were aligned to the predicted genes using PASA [42] and AAT [43] packages. The predicted genes and evidence sequences were then assembled according to the weight of each evidence type using EVidenceModeler. The highest scoring set of connected exons, introns, and noncoding regions was selected as a consensus gene model. Proteins encoded by gene models were searched against the Pfam database [44] and automatically assigned a putative name based on conserved domain hits or similarity with previously identified proteins. Annotated gene models were also searched against a database of plant transposon-encoded proteins [45]. Predicted proteins with a top match to transposon-encoded proteins were excluded from the annotation and gene counts. Transfer RNAs were identified using tRNAscan-SE [46]. To scan miRNA genes, the nonredundant miRNA sequences in miRBase v15 were mapped using BLASTN (up to two mismatches) [47]. A search of potential precursor structures was performed by extracting the genomic context (400 bp upstream and downstream) surrounding the position of the miRNA sequence predicted and by analyzing those regions with Vienna RNA package [48]. Only the putative pre-miRNA precursors with a folding energy lower than -20 kcal/mol were selected. Organellar insertions were determined using BLASTN with the *A. thaliana *mitochondrion and the *B. rapa *chloroplast genome sequence using a cutoff of 95% identity plus 90% coverage.

### Comparative genome analysis

Syntenic regions between chromosome A3 of *B. rapa *and the *A. thaliana *genome were identified by a proteome comparison based on BLASTP analysis [47]. The entire proteomes of the two genomes were compared, and only the top reciprocal BLASTP matches per chromosome pair were selected (minimum of 50% alignment coverage at a cutoff of < E^-20^). Chromosome scale synteny blocks were inferred by visual inspection of dot-plots using DiagHunter with parameters as described in the previous reports [6,49]. At least four genes with the same respective orientations in both genomes were required to establish a primary candidate synteny block. To distinguish highly homologous real synteny blocks from false positives due to multiple rounds of polyploidy followed by genome rearrangement, we manually evaluated the degree of gene conservation in all the primary candidate blocks and selected real syntenic regions showing a gene conservation index of greater than 50% (the number of conserved matches divided by the total number of genes in the blocks). Self comparison of chromosome A3 with other chromosomes of the *B. rapa *genome was also conducted using seed BAC sequences [6].

## Abbreviations

AK: ancestral karyotype; BAC: bacterial artificial chromosome; BES: BAC-end sequence; bp: base pair; BrGSP: *Brassica rapa *Genome Sequencing Project; CD: collinearity discontinuity; DAPI: 4':6-diamidino-2-phenylindole dihydrochloride; EST: expressed sequence tag; FISH: fluorescent *in situ *hybridization; GO: Gene Ontology; kb: kilobase; miRNA: microRNA; NOR: nucleolar organizer region.

## Authors' contributions

JHM conceived the project, designed research, analyzed data, and wrote the manuscript. SJK designed research, performed the experiments, and analyzed data. JHM, SJK, JAK, MHL, SIL, JKH, THP, SCL, MJL, JYP, JL, TJY, and IYC contributed to shotgun sequencing, sequence assembly, and data acquisition. MJ and JSK performed genetic mapping. YJH and KBL contributed to FISH. YJS and JHH contributed to annotation and database development. YJS, BJK, SB, JYS, MSS, HJY, and BSC analyzed data. SRC, NR, YPL, FF, ND, ES, MT, IB, AGS, IAPP, JB, and DE participated in BAC-end sequencing. HJY and IB participated in manuscript preparation. BSP conceived the project.

## Supplementary Material

Additional file 1 **Figures S1, S2, S3, and S4**. Figure S1: genetic versus physical distance on chromosome A3. The genetic map was constructed using the *VCS *population. Figure S2: frequency distribution of genes in multigene families with tandem duplicated paralog arrangements. Tandem duplicated paralogs on chromosome A3 were identified using BLASTP analysis with a minimum threshold of 50% alignment coverage at a cutoff of E^-10 ^in a 100-kb window interval. Figure S3: clusters of tRNA^Pro ^genes on chromosome A3. The tRNA^Pro ^repeat clusters at 23.68 Mb is located on BAC clone KBrH72P15. Figure S4: dot plot of chromosome A3 compared with itself. Each dot in the dot plot represents a reciprocal best BLASTP match between gene pairs at a cutoff value of < E^-20^. Black dots show the regions of synteny identified by DiagHunter Click here for file

Additional file 2 **Tables S1, S2, S3, and S4**. Table S1: summary of sequence contigs along with constituent BAC associations on minimum tiling path for chromosome A3. Table S2: comparison of repetitive sequences identified on chromosome A3 and seed BAC sequences of *B. rapa*. Table S3: miRNAs identified on chromosome A3. Table S4: synteny alignment between *B. rapa *chromosome A3 and the *A. thaliana *genome along with mapping of AK genome building blocks. Click here for file
