# Supplementary figures and images for: Sequence and structure of Brassica rapa chromosome A3
Source: Genome Biol. 2010 Sep 27;11(9):R94. doi: 10.1186/gb-2010-11-9-r94 (PMC2965386; doi:10.1186/gb-2010-11-9-r94)

Figure S1

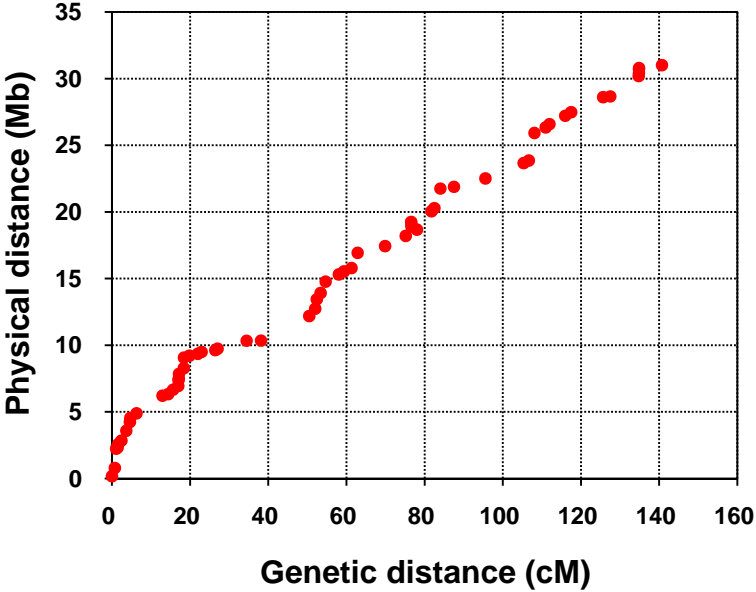

Figure S2

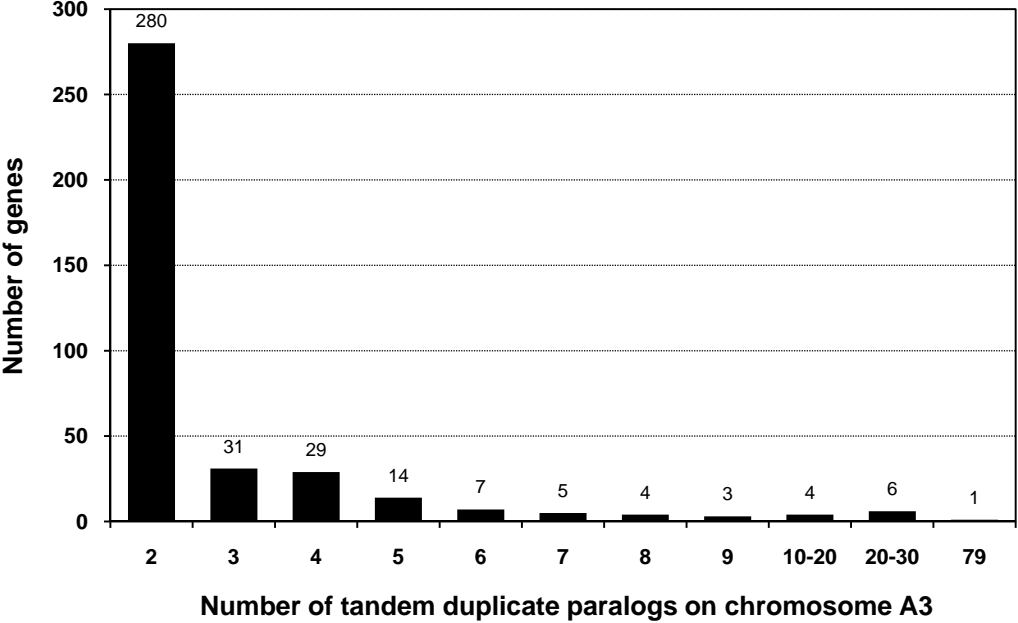

Figure S3

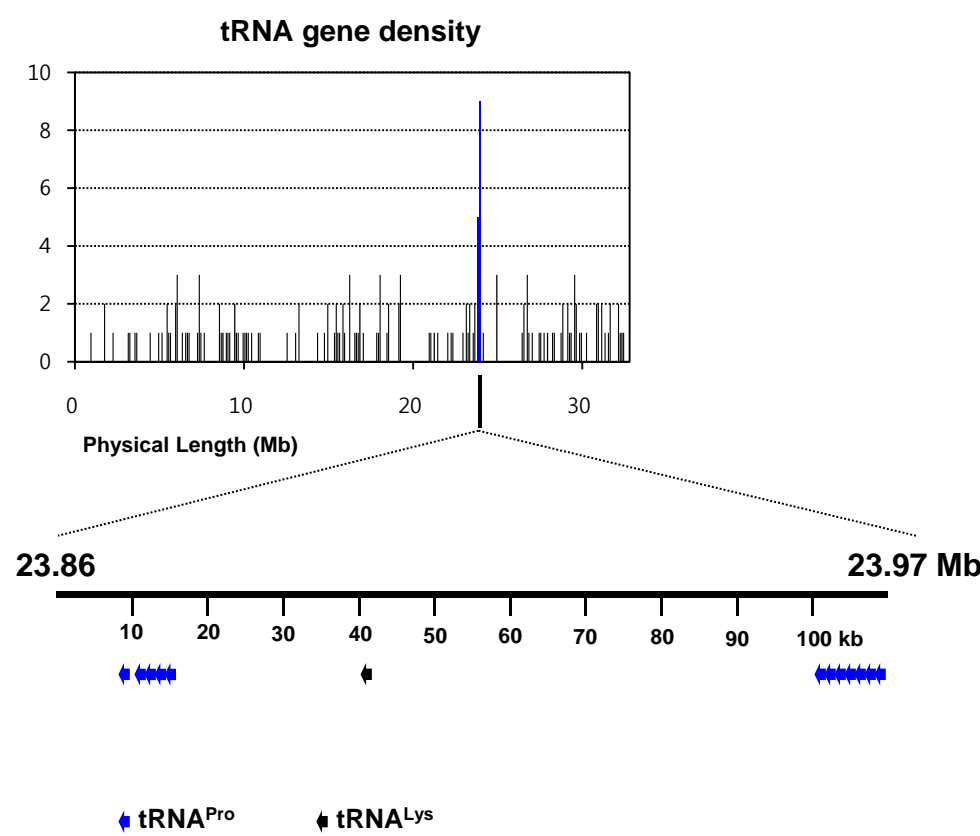

Figure S4

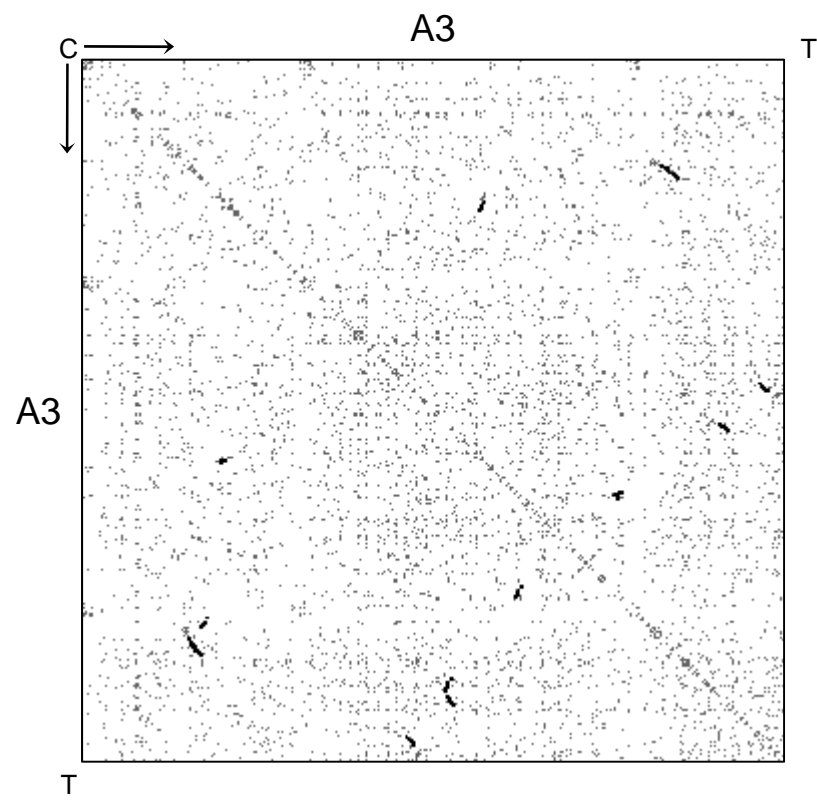

Supplement: Additional file 1 — Figures S1, S2, S3, and S4. Figure S1: genetic versus physical distance on chromosome A3. The genetic map was constructed using the VCS population. Figure S2: frequency distribution of genes in multigene families with tandem duplicated paralog arrangements. Tandem duplicated paralogs on chromosome A3 were identified using BLASTP analysis with a minimum threshold of 50% alignment coverage at a cutoff of E-10 in a 100-kb window interval. Figure S3: clusters of tRNAPro genes on chromosome A3. The tRNAPro repeat clusters at 23.68 Mb is located on BAC clone KBrH72P15. Figure S4: dot plot of chromosome A3 compared with itself. Each dot in the dot plot represents a reciprocal best BLASTP match between gene pairs at a cutoff value of < E-20. Black dots show the regions of synteny identified by DiagHunter [file gb-2010-11-9-r94-S1.pdf]
