# Supplementary material for: Sequence and structure of Brassica rapa chromosome A3
Source: Genome Biol. 2010 Sep 27;11(9):R94. doi: 10.1186/gb-2010-11-9-r94 (PMC2965386; doi:10.1186/gb-2010-11-9-r94)
Supplement: Additional file 2 — Tables S1, S2, S3, and S4. Table S1: summary of sequence contigs along with constituent BAC associations on minimum tiling path for chromosome A3. Table S2: comparison of repetitive sequences identified on chromosome A3 and seed BAC sequences of B. rapa. Table S3: miRNAs identified on chromosome A3. Table S4: synteny alignment between B. rapa chromosome A3 and the A. thaliana genome along with mapping of AK genome building blocks. [file gb-2010-11-9-r94-S2.pdf]

Table S1. Summary of sequence contigs along with constituent BAC associations on minimum tiling path for chromosome A3.

Contig	Clone Name	Accession	Size (bp)	Contig Start	Contig End	*VCS* map (cM)
Contig 1	KBrH044B01	AC241130	97,931	1	97,948	
	KBrH031P21	AC241116	102,782	89,099	190,045	
	KBrB009D14	AC189214	138,346	172,377	304,717	0.0
	KBrH014A24	AC241100	113,175	295,097	408,275	
	KBrB075G04	AC241063	126,677	374,293	506,355	
	KBrB066F15	AC241057	107,357	424,983	513,629	
	KBrH123D22	AC241191	104,793	470,666	577,273	
Contig 2	KBrH109B05	AC241180	151,133	1	151,133	
	KBrB089C22	AC241074	120,043	129,017	249,064	
	KBrH092L18	AC241160	101,362	213,130	305,348	0.7
	KBrH106H11	AC241175	162,086	277,992	440,087	
	KBrB010A02	AC240988	107,629	430,291	547,812	
	KBrH005P10	AC189552	125,330	494,184	619,528	
	KBrB102C10	AC241082	123,550	602,340	717,669	
Contig 3	KBrH004O24	AC241091	117,022	1	117,023	
	KBrH095A16	AC241163	127,733	80,532	185,557	
	KBrH109I16	AC241181	118,674	163,496	275,412	
	KBrS015D14	AC241201	134,414	285,413	419,828	
	KBrB045H16	AC241035	103,153	396,731	501,162	
	KBrH050H19	AC241138	149,767	494,475	645,116	
	KBrB055L17	AC241044	114,815	631,757	739,413	
	KBrB010M19	AC189222	163,034	635,274	798,324	
Contig 4	KBrB013F01	AC240992	147,654	1	147,655	
	KBrB063G24	AC241052	139,252	142,918	284,456	1.1
	KBrB089G06	AC241075	153,003	220,631	377,234	1.5
	KBrH029A20	AC232557	127,242	365,366	492,607	
	KBrB043B23	AC189350	135,875	475,213	611,086	1.6
	KBrB111K20	AC241084	91,259	621,087	712,332	
	KBrB107P11	AC241083	42,683	722,333	765,692	
	KBrB079E08	AC232524	108,012	746,563	854,589	2.4
	KBrB053O09	AC241043	105,196	826,981	933,419	
	KBrH120P14	AC241189	108,423	908,307	1,016,749	
	KBrB078N23	AC241069	147,940	999,511	1,153,409	
	KBrH111N19	AC241184	154,937	1,049,123	1,204,092	
	KBrH069I07	AC241144	147,187	1,191,624	1,338,405	
	KBrH014C15	AC241101	138,719	1,334,548	1,473,266	
	KBrB077E07	AC241065	134,639	1,483,267	1,617,927	3.7
	KBrH094K10	AC241161	117,793	1,586,763	1,699,192	
	KBrB047J10	AC241039	104,442	1,671,384	1,778,294	
	KBrB013N01	AC240993	137,141	1,750,391	1,887,531	
	KBrH096J02	AC241167	125,734	1,866,137	1,961,717	
	KBrB065L05	AC241056	134,519	1,926,235	2,079,567	
	KBrB070M16	AC189446	126,053	1,931,561	2,057,637	
	KBrH015E17	AC189604	113,461	2,070,409	2,183,881	
	KBrH102D05	AC241170	93,931	2,140,976	2,251,977	4.6
	KBrB006K11	AC240982	117,107	2,211,501	2,328,607	
	KBrH105I22	AC241174	102,094	2,338,608	2,440,701	
	KBrB021P17	AC189263	135,260	2,450,702	2,585,977	4.7
	KBrB069M23	AC189441	127,158	2,559,687	2,686,875	
	KBrH022C13	AC241106	106,835	2,675,507	2,782,414	
	KBrB014O06	AC189237	105,067	2,792,415	2,897,481	6.3
	KBrB042J06	AC241030	132,922	2,883,642	3,016,563	
	KBrH023N19	AC241111	111,911	3,014,146	3,126,059	
	KBrH035D15	AC241122	85,348	3,072,013	3,159,349	

	KBrB038L07	AC241019	92,314	3,169,350	3,266,248	
	KBrB028P01	AC189300	101,741	3,231,837	3,333,580	
	KBrB077I08	AC241067	141,574	3,343,581	3,485,150	
	KBrH023G06	AC241109	135,434	3,480,514	3,615,947	
	KBrB047E18	AC241038	113,677	3,607,705	3,721,383	
	KBrB045I01	AC241036	102,572	3,675,292	3,777,864	
	KBrH143G12	AC241199	111,007	3,776,357	3,887,385	
	KBrH005A08	AC189548	60,322	3,857,469	3,917,819	
	KBrH123I10	AC241192	163,028	3,900,029	4,050,342	
	KBrB039C02	AC241021	147,438	4,034,284	4,168,714	
	KBrB053E08	AC232496	134,757	4,115,594	4,250,364	12.9
	KBrB091M11	AC232535	137,380	4,237,461	4,374,844	14.4
	KBrB011H03	AC240990	151,155	4,301,312	4,485,496	
	KBrS011B08	AC189650	96,047	4,410,053	4,494,470	
	KBrB042H15	AC241029	97,957	4,449,061	4,564,126	
	KBrH125B10	AC241195	60,300	4,522,682	4,582,987	
	KBrB080F13	AC241070	137,790	4,564,133	4,701,922	15.7
	KBrB024N20	AC241001	155,195	4,682,241	4,837,435	
	KBrH129J18	AC241197	177,500	4,847,436	5,024,735	17.0
	KBrB045E11	AC241034	104,600	5,034,736	5,139,335	
	KBrH102F05	AC241171	135,015	5,149,336	5,284,350	
	KBrH059N21	AC229605	131,311	5,294,351	5,422,734	17.0
	KBrB009M08	AC240987	141,319	5,404,184	5,545,793	17.1
	KBrB039B11	AC241020	146,213	5,422,463	5,568,655	
	KBrH097C05	AC241168	104,187	5,567,080	5,671,233	
	KBrE041C10	AC241087	37,587	5,681,234	5,718,820	
	KBrH039L17	AC241126	101,813	5,710,981	5,812,794	
	KBrB085J21	AC232529	128,825	5,748,690	5,878,558	17.1
	KBrB058C06	AC241047	95,878	5,810,897	5,883,462	
	KBrB035K06	AC241013	106,334	5,844,121	5,950,459	
	KBrB030D12	AC241009	124,873	5,960,460	6,085,332	
	KBrB069I14	AC241058	155,562	6,079,913	6,229,330	
	KBrH006A08	AC189553	151,968	6,177,589	6,329,563	18.4
	KBrH022L17	AC241107	114,221	6,326,661	6,440,410	
	KBrB077H09	AC241066	168,977	6,393,622	6,586,617	
	KBrH045E23	AC232561	123,468	6,581,483	6,704,953	
	KBrH112A03	AC241185	135,376	6,658,645	6,793,701	
	KBrB018I17	AC240995	141,126	6,751,093	6,892,437	
	KBrB084K23	AC189490	147,448	6,831,386	6,978,833	
	KBrS003O10	AC189632	118,826	6,971,789	7,090,614	18.4
	KBrB047I09	AC232489	142,766	7,063,350	7,224,564	
	KBrB020F24	AC240996	101,920	7,112,934	7,210,551	19.8
	KBrH121N24	AC229606	76,187	7,207,430	7,283,606	
	KBrB089H07	AC189511	140,025	7,257,144	7,392,609	22.0
	KBrH003A15	AC241088	96,138	7,382,497	7,478,626	
	KBrB084F01	AC189486	136,504	7,397,042	7,547,758	22.9
	KBrS012D09	AC189652	147,147	7,531,290	7,678,393	26.4
	KBrS001M03	AC189627	115,604	7,645,275	7,760,878	27.0
	KBrB132O08	AC241086	134,286	7,747,940	7,864,852	
	KBrB002E24	AC189189	121,528	7,782,161	7,903,691	
	KBrB043L22	AC189354	101,703	7,913,692	8,015,394	
	KBrB023M09	AC240999	100,134	8,002,202	8,100,994	
	KBrH010M06	AC237305	121,580	8,020,922	8,135,699	
	KBrH108E21	AC241178	105,693	8,134,907	8,240,599	
	KBrB034G03	AC190051	121,855	8,239,861	8,341,847	34.5
	KBrH036K21	AC241123	106,189	8,302,057	8,408,383	
Contig 5	KBrB056J14	AC241046	113,469	1	113,472	38.1

	KBrB039F21	AC241022	139,040	102,025	240,187	
	KBrB091E13	AC232534	128,903	197,165	316,827	
	KBrH052P11	AC241140	110,012	286,425	396,457	
	KBrH069M03	AC241145	100,985	371,727	473,023	
	KBrB037E22	AC232481	103,670	459,209	557,840	
	KBrB011D06	AC232452	119,869	516,548	636,127	
	KBrS008C11	AC189641	108,677	609,834	718,535	
	KBrB058B22	AC189403	110,640	671,323	781,966	
	KBrH004I22	AC237303	110,934	759,592	859,344	
	KBrB001J17	AC229603	112,361	845,302	968,426	
	KBrB072K21	AC232519	101,717	927,094	1,028,807	
	KBrH091C07	AC241157	49,001	1,007,654	1,057,963	
	KBrH105A19	AC241173	102,823	1,067,964	1,170,786	
	KBrB118O08	AC241085	136,190	1,180,787	1,316,976	
	KBrH089O12	AC241153	109,754	1,312,789	1,422,542	
	KBrB037A01	AC189327	101,150	1,346,164	1,447,316	
	KBrB030G22	AC241010	144,183	1,427,451	1,570,034	
	KBrB059A03	AC189406	139,749	1,580,035	1,719,783	
	KBrH124C09	AC241193	146,084	1,701,460	1,848,720	
	KBrB009B09	AC189213	143,244	1,841,188	1,984,431	50.5
	KBrH113B15	AC241186	124,258	1,994,432	2,118,689	
	KBrH048J07	AC241136	103,805	2,115,163	2,222,093	
	KBrB044L16	AC189359	133,781	2,170,724	2,304,509	
	KBrH046K16	AC241133	103,031	2,227,378	2,330,411	
	KBrB069K06	AC241059	135,012	2,281,199	2,394,070	
	KBrH040N18	AC241128	121,949	2,389,745	2,511,695	52.0
	KBrH028K05	AC241114	130,755	2,479,853	2,610,769	
	KBrB070K15	AC232517	144,897	2,561,392	2,713,468	
	KBrH023I01	AC241110	136,087	2,723,469	2,859,547	
	KBrH034I10	AC241119	97,103	2,847,518	2,944,621	
Contig 6	KBrB056E07	AC241045	119,868	1	119,618	
	KBrB036B21	AC189317	110,411	98,896	209,306	
	KBrB052N08	AC189387	129,570	154,567	261,447	52.4
	KBrB010F13	AC189218	128,973	241,358	370,332	
	KBrB073B03	AC241061	134,630	358,635	491,156	
	KBrB044L24	AC241032	99,951	458,482	558,766	
	KBrB021F10	AC189256	109,476	485,177	592,656	
	KBrH121N04	AC241190	148,715	485,907	634,503	
	KBrB059A02	AC241050	101,237	605,367	706,087	
	KBrB042O05	AC189349	119,891	632,050	751,952	53.4
	KBrB019N06	AC189253	143,759	737,233	880,991	
	KBrH001D20	AC189532	87,166	879,299	1,027,168	
	KBrB091E23	AC241077	157,344	833,009	1,039,050	
	KBrB047D06	AC189369	99,481	1,026,833	1,125,056	
	KBrB088F12	AC241073	165,753	1,099,777	1,255,404	
	KBrH119A16	AC241187	109,430	1,247,751	1,357,205	
	KBrB041O21	AC241027	92,255	1,345,754	1,432,916	
	KBrB035O14	AC241014	94,857	1,409,463	1,500,203	
	KBrH122D17	AC232569	142,455	1,477,834	1,620,286	54.7
	KBrH005J04	AC241092	105,493	1,590,649	1,696,129	
	KBrB068B07	AC189431	145,501	1,665,374	1,810,876	
	KBrB026A13	AC241003	120,236	1,803,313	1,923,548	
	KBrB037L23	AC241017	130,937	1,956,024	2,094,443	
	KBrB055G10	AC189394	109,674	2,017,220	2,126,898	58.0
	KBrH107M15	AC241176	157,430	2,074,326	2,231,679	
	KBrB005K09	AC240981	132,713	2,220,520	2,352,979	
	KBrH034E17	AC241117	113,974	2,252,411	2,367,596	59.3

	KBrH011G10	AC189577	121,618	2,377,597	2,499,216	
	KBrH005H18	AC189550	85,248	2,498,436	2,583,683	61.3
	KBrB013O20	AC189233	128,372	2,581,575	2,709,946	
	KBrH013F17	AC232553	110,242	2,700,187	2,810,428	
	KBrB003A10	AC189193	134,395	2,807,938	2,942,332	
	KBrH012A23	AC189582	84,749	2,928,502	3,012,180	
	KBrB004M08	AC240980	128,849	3,002,403	3,131,028	
	KBrB037C07	AC189329	103,109	3,099,844	3,202,957	
	KBrB043L02	AC189352	89,756	3,183,064	3,272,831	
	KBrB016C20	AC240994	142,258	3,240,634	3,383,310	
	KBrB001H24	AC189185	110,488	3,371,637	3,482,135	
	KBrB003I01	AC238677	153,820	3,407,681	3,558,499	
	KBrH065D07	AC241143	127,153	3,516,125	3,643,277	
	KBrB049D17	AC189377	91,521	3,640,776	3,732,296	62.9
	KBrB055E21	AC232500	114,197	3,742,297	3,862,972	
	KBrB045L13	AC241037	158,495	3,842,250	4,002,086	
	KBrB010G10	AC240989	118,036	3,960,559	4,078,594	
	KBrH110N21	AC241183	81,233	4,075,288	4,156,522	
	KBrH085M22	AC241150	118,521	4,147,989	4,266,511	69.9
	KBrB007K04	AC240984	141,102	4,192,316	4,333,419	
	KBrB027P21	AC232474	145,178	4,343,420	4,488,597	
	KBrB048D13	AC232395	140,461	4,464,947	4,617,629	
	KBrB027L04	AC241005	116,970	4,498,598	4,619,230	
	KBrH045I10	AC241131	95,774	4,593,155	4,689,207	
	KBrB002P01	AC232438	132,867	4,644,228	4,777,097	
	KBrB040A20	AC241024	110,548	4,748,620	4,858,370	
	KBrH090C12	AC241155	119,484	4,813,604	4,933,090	
	KBrH011C16	AC241098	106,511	4,909,648	5,017,929	75.2
Contig 7	KBrH090L06	AC241156	125,048	1	125,049	
	KBrH009K01	AC232547	130,486	67,850	198,361	
	KBrH100N23	AC241169	156,456	196,177	352,300	
	KBrB051E01	AC232493	142,706	362,301	505,006	78.0
	KBrH022O14	AC241108	86,592	515,007	601,600	
	KBrB042K13	AC241031	122,402	588,997	711,399	76.6
	KBrB061C02	AC232509	134,601	721,400	856,015	76.6
	KBrB036E23	AC241015	159,534	812,912	972,548	
	KBrB078E04	AC241068	139,118	947,815	1,086,550	76.6
Contig 8	KBrH034P23	AC241121	100,610	1	100,610	
	KBrB086B23	AC232530	151,225	56,695	207,919	
	KBrH110M01	AC237306	146,796	174,299	320,692	
	KBrB094E24	AC241079	136,178	316,600	458,162	
	KBrH110B04	AC241182	94,811	322,381	418,566	
	KBrB025P12	AC241002	103,077	369,322	476,324	
	KBrB001M21	AC240977	94,983	472,364	567,316	
	KBrB038M13	AC189333	124,756	545,743	670,498	
	KBrB002E01	AC240978	132,441	656,265	788,546	81.8
	KBrB008O16	AC240985	161,656	670,505	832,179	
	KBrB068E07	AC189432	105,441	789,782	895,217	
	KBrH010E23	AC241097	136,517	892,077	1,028,379	82.5
	KBrB027C16	AC190050	227,428	1,038,380	1,280,770	
	KBrH036P09	AC241125	96,312	1,161,821	1,257,016	
	KBrB050C17	AC232492	155,235	1,173,804	1,329,344	
	KBrB045E05	AC241033	101,382	1,248,021	1,349,409	
	KBrH081F04	AC241148	138,522	1,257,150	1,394,933	
	KBrH011O17	AC189580	100,254	1,326,095	1,426,361	
	KBrH003H20	AC241089	105,517	1,412,126	1,506,626	
	KBrH021E08	AC241105	154,477	1,460,087	1,623,186	

	KBrB056I08	AC189399	119,637	1,548,725	1,668,373	
	KBrH004M22	AC241090	152,532	1,645,300	1,797,211	
	KBrH006E03	AC241095	103,904	1,749,482	1,837,543	
	KBrH095M04	AC241165	121,416	1,775,711	1,897,352	
	KBrB029K16	AC241007	125,298	1,886,865	2,016,751	
	KBrB030D06	AC241008	159,991	1,972,741	2,131,974	
	KBrH017L09	AC241104	121,007	2,124,991	2,245,999	
	KBrB081A21	AC241072	110,102	2,256,000	2,366,101	
	KBrB086M08	AC232531	122,820	2,356,109	2,478,928	84.0
	KBrB073C04	AC241062	122,756	2,488,929	2,611,684	87.5
	KBrH008D11	AC241096	110,572	2,591,892	2,702,463	
	KBrB032C14	AC189306	104,954	2,701,736	2,806,689	
	KBrH095B11	AC241164	102,453	2,751,701	2,856,436	
	KBrB059E18	AC241051	66,493	2,866,437	2,932,916	
	KBrH007P05	AC189561	116,270	2,907,369	3,023,636	
	KBrF191K20	AC240940	34,253	2,994,910	3,028,241	
	KBrH092E03	AC241159	121,752	3,038,242	3,159,993	
	KBrB031G07	AC189304	105,399	3,121,374	3,226,792	95.6
	KBrH027D03	AC241113	98,879	3,208,445	3,307,570	
	KBrH047I20	AC241135	106,356	3,297,711	3,404,066	
	KBrB003I04	AC240979	113,542	3,391,017	3,502,851	
	KBrH059M15	AC241142	146,896	3,394,515	3,598,268	
	KBrS012M03	AC189655	107,479	3,583,508	3,690,987	
	KBrB063O08	AC241053	141,838	3,679,150	3,830,456	
	KBrB047H21	AC232488	151,751	3,706,223	3,857,976	
	KBrH072P15	AC241146	122,358	3,867,977	3,990,334	
	KBrH047C14	AC241134	118,354	3,980,921	4,133,213	
	KBrB080O07	AC189477	189,477	4,058,684	4,208,087	
	KBrB082C18	AC232525	106,010	4,148,599	4,283,677	
	KBrH003D18	AC189536	131,847	4,271,448	4,403,296	105.3
	KBrB074K06	AC189463	104,457	4,361,947	4,464,403	
	KBrB068H20	AC189433	143,250	4,463,225	4,606,474	106.7
	KBrH108K15	AC241179	118,741	4,595,201	4,707,469	
	KBrH005J02	AC189551	120,265	4,601,677	4,721,941	
	KBrB071A06	AC232518	140,628	4,715,336	4,889,422	
	KBrB026F03	AC189293	100,568	4,792,055	4,892,691	
	KBrH038M21	AC232559	118,451	4,884,410	5,002,886	
	KBrH139O06	AC241198	128,488	5,012,887	5,141,375	
	KBrH013B15	AC241099	109,060	5,082,601	5,189,166	
	KBrS012H21	AC232577	125,891	5,199,167	5,325,057	
Contig 9	KBrF153M15	AC240938	31,870	1	31,871	
	KBrH120G08	AC241188	76,373	26,542	102,914	
	KBrS010N24	AC241200	124,586	112,915	243,208	
	KBrB003D07	AC232439	124,893	203,516	328,404	
	KBrH089O13	AC241154	128,676	305,579	459,469	
	KBrB059C07	AC232505	144,556	350,661	532,318	
	KBrB006G23	AC232446	123,630	472,108	595,798	
	KBrH094M21	AC241162	174,053	605,799	779,851	
	KBrH104A12	AC241172	101,441	776,787	878,255	
	KBrB094O14	AC241081	133,256	817,207	962,140	
	KBrB007C08	AC240983	142,238	868,087	1,004,331	
	KBrB054N05	AC189392	108,621	962,147	1,070,683	
	KBrH034L18	AC241120	92,597	1,047,825	1,140,437	
	KBrH089I14	AC241152	135,219	1,086,762	1,222,097	
	KBrB030F12	AC189303	128,174	1,200,293	1,328,466	108.1
	KBrH004B20	AC189542	121,034	1,326,103	1,447,139	
	KBrB091A17	AC241076	113,119	1,423,907	1,540,329	

KBrH124P11	AC241194	119,416	1,494,524	1,613,947	
KBrB027E01	AC232472	131,162	1,623,948	1,759,677	111.0
KBrB065L02	AC241055	149,241	1,711,376	1,860,937	
KBrB071P24	AC189451	121,155	1,835,972	1,957,146	
KBrH084J08	AC241149	150,721	1,867,442	2,021,955	111.9
KBrH015L02	AC241103	110,831	1,983,700	2,094,789	
KBrB024J23	AC241000	119,854	2,003,826	2,123,752	
KBrH091F08	AC241158	133,057	2,133,753	2,266,809	
KBrB011L12	AC240991	140,483	2,276,810	2,417,376	
KBrH006B04	AC241094	129,541	2,295,651	2,430,730	
KBrB042E12	AC241028	108,548	2,419,925	2,533,163	
KBrB020N18	AC240997	110,835	2,493,160	2,608,685	116.0
KBrH040A03	AC241127	100,904	2,602,047	2,702,975	
KBrB058P11	AC241049	101,393	2,665,920	2,767,363	
KBrH048N09	AC241137	45,565	2,759,406	2,804,970	117.5
KBrB053E22	AC241042	149,657	2,814,971	2,964,627	
KBrH034F10	AC241118	95,786	2,945,694	3,041,442	
KBrH046C14	AC241132	114,337	3,014,216	3,128,522	
KBrH015D02	AC241102	121,332	3,120,847	3,242,484	
KBrB004L02	AC189197	121,695	3,174,983	3,296,677	
KBrH036K23	AC241124	138,346	3,306,678	3,445,057	
KBrH029F06	AC241115	110,945	3,418,914	3,529,166	
KBrB033I17	AC241011	93,083	3,539,167	3,646,481	
KBrB091G21	AC241078	160,847	3,630,559	3,785,564	
KBrH042D10	AC241129	102,741	3,777,759	3,880,499	
KBrB018H04	AC232456	70,825	3,890,500	3,956,964	125.7
KBrH013I08	AC232554	99,567	3,943,565	4,043,132	127.5
KBrH003M07	AC232539	127,712	4,041,048	4,144,963	
KBrH054I24	AC241141	125,115	4,110,200	4,237,009	
KBrB050H15	AC241040	104,885	4,177,892	4,284,212	
KBrB038I20	AC241018	158,219	4,284,211	4,431,686	
KBrB008I08	AC189212	126,175	4,413,272	4,539,446	
KBrB080H21	AC241071	171,160	4,525,673	4,697,194	
KBrH014H10	AC189599	118,513	4,621,696	4,740,213	
KBrB037I10	AC241016	110,818	4,728,304	4,836,523	
KBrB029G12	AC241006	147,829	4,751,539	4,900,944	
KBrB033I20	AC241012	124,938	4,815,554	4,940,040	
KBrB010H02	AC232450	132,912	4,916,768	5,049,685	
KBrH096C04	AC241166	117,631	5,059,686	5,177,317	
KBrH006A23	AC241093	126,030	5,165,625	5,295,146	
KBrH052O08	AC155342	151,550	5,305,147	5,412,516	
KBrB009G06	AC240986	138,032	5,332,940	5,475,975	
KBrB070G10	AC241060	128,681	5,368,032	5,499,714	
KBrB022F09	AC240998	110,025	5,425,406	5,535,432	
KBrH006C14	AC189554	138,600	5,469,070	5,607,669	134.8
KBrB041G12	AC241025	84,915	5,556,463	5,646,568	
KBrB026J02	AC241004	119,180	5,629,553	5,748,740	
KBrB001B07	AC189184	106,945	5,706,600	5,813,400	134.8
KBrH126E06	AC241196	103,665	5,779,193	5,882,860	
KBrB046M04	AC189367	126,335	5,867,249	5,993,583	
KBrB055N13	AC189396	139,461	5,990,671	6,130,293	134.8
KBrH076J22	AC241147	107,326	6,072,161	6,181,524	134.9
KBrB041M20	AC241026	100,854	6,130,289	6,228,249	
KBrB058I10	AC241048	109,015	6,198,030	6,307,072	
KBrH051B19	AC241139	119,941	6,290,842	6,410,803	140.7
KBrB051F06	AC241041	121,569	6,347,578	6,430,950	
KBrH088G21	AC241151	123,487	6,381,704	7,311,575	

KBrB092L14	AC189528	133,095	6,496,313	6,629,406
KBrH027C06	AC241112	106,138	7,262,800	6,644,129
KBrB076A06	AC241064	104,813	6,618,650	6,722,710
KBrB094L22	AC241080	124,521	6,700,996	6,825,518
KBrB039N20	AC241023	105,695	6,796,826	6,902,517
KBrB065G03	AC241054	152,372	6,894,158	7,046,529
KBrB045I17	AC189362	105,566	7,044,548	7,150,113
KBrH108D17	AC241177	83,741	7,089,242	7,171,976
KBrF203I22	AC240941	36,849	7,149,257	7,186,102

Table S2. Comparison of repetitive sequences identified on chromosome A3 and seed BAC sequences of *B. rapa*.

	Genome coverage (%)[1]	
Family	A3	Seed BACs
SINEs	0.1	0.1
LINEs	1.0	1.3
LTRs	2.6	2.4
DNA transposon	1.9	2.2
Satellites	0.0	0.4
Low complexity repetitive sequences	4.5	4.4
Others[2]	0.8	0.4
Total	11.0	11.2

[1]Genomic coverage was calculated using 31.9 Mb for chromosome A3 and 68.5 Mb for seed BAC sequences that we reported previously [6]. [2]This refers to simple sequence repeats and short tandem repeats. LINES, long interspersed element; SINE, short interspersed element.

Table S3. miRNAs identified on chromosome A3.

miRNA	Start	End	Strand	miRNA sequence	Potential precursor structure	Genomic loci
miR170	230,121	230,141	+	TGATTGAGCCGTGCCAATATC	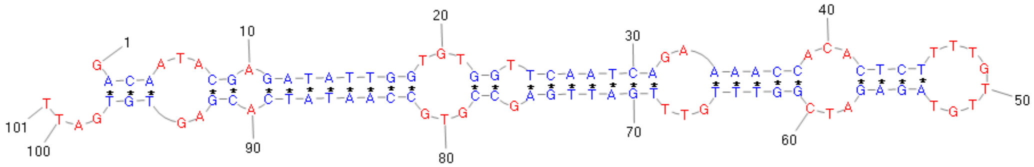	IG
miR171	230,124	230,144	+	TTGAGCCGTGCCAATATCACG	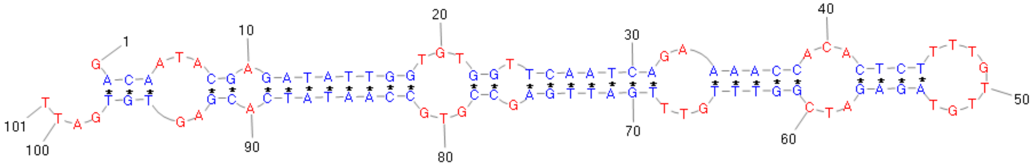	IG
miR156	3,260,299	3,260,318	+	TGACAGAAGAGAGTGAGCAC	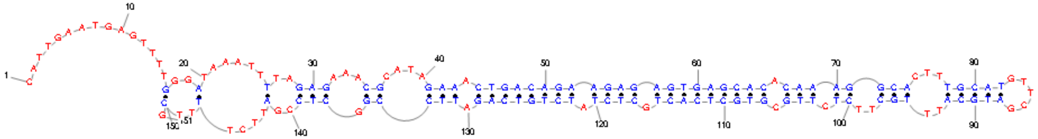	IG
miR156	3,652,352	3,652,333	-	TGACAGAAGAGAGTGAGCAC	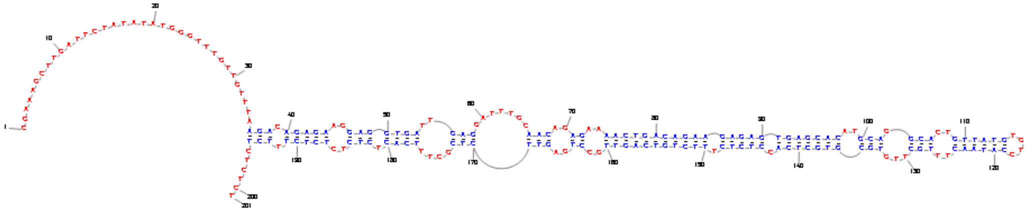	IG
miR828	5,190,496	5,190,475	-	TCTTGCTTAAATGGGTATTCCA	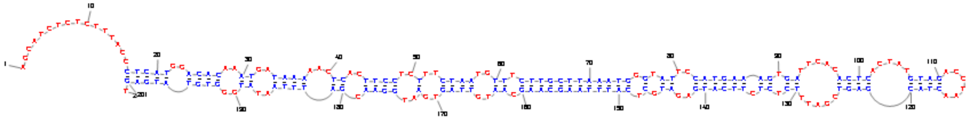	IG
miR168	8,363,644	8,363,625	-	TCGCTTGGTGCAGGTCGGGA	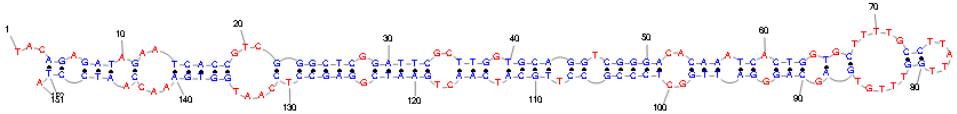	IG
miR160	9,051,143	9,051,123	-	TGCCTGGCTCCCTGTATGCCA	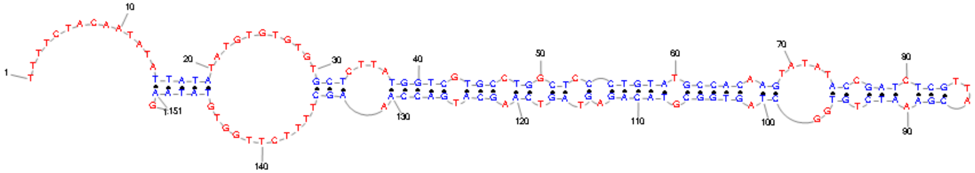	IG

miR167	12,087,682	12,087,662	-	TGAAGCTGCCAGCATGATCTA	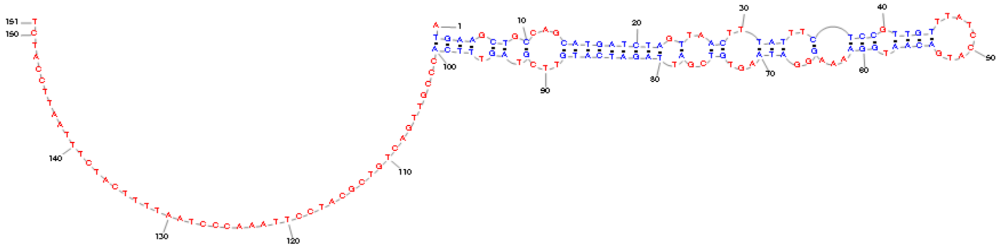	IG
miR2111	15,826,673	15,826,693	+	TAATCTGCATCCTGAGGTTTA	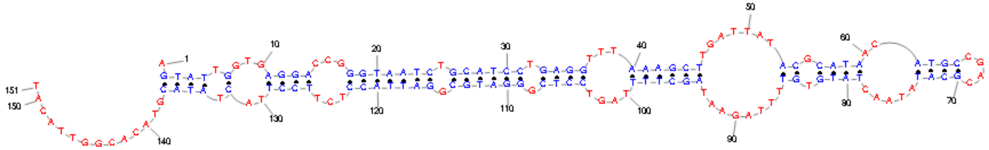	IG
miR2111	15,826,755	15,826,775	+	GTCCTCGGGATGCGGATTACC	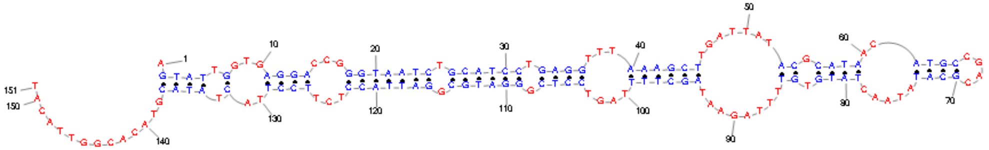	IG
miR408	21,188,065	21,188,045	-	ATGCACTGCCTCTTCCCTGGC	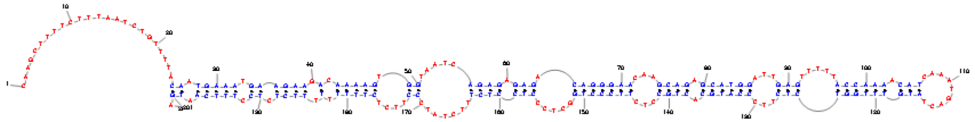	IG
miR399	23,831,675	23,831,655	-	TGCCAAAGGAGATTTGTCCGG	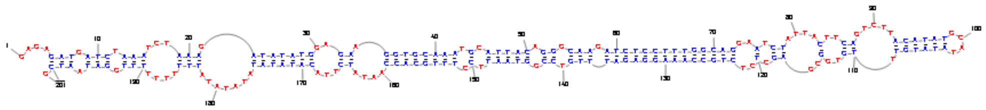	IG
miR399	23,833,469	23,833,449	-	TGCCAAAGGAGATTTGCCTCG	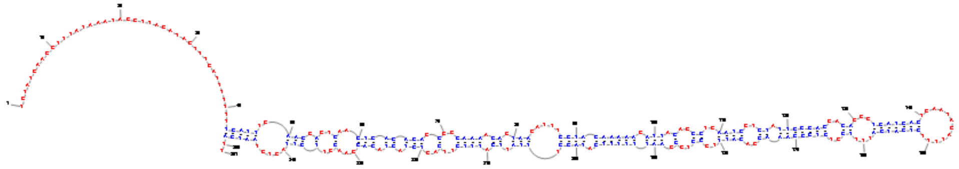	IG
miR399	23,834,082	23,834,102	+	TGCCAAAGGAGATTTGCCCCG	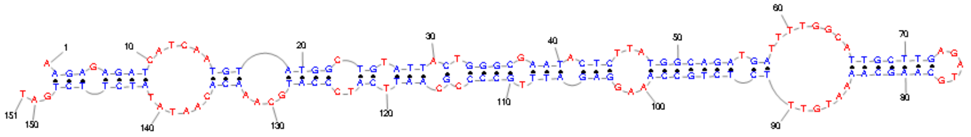	IG

miR399	25,795,156	25,795,137	-	TGCCAAAGGAGAGTTGCCCT	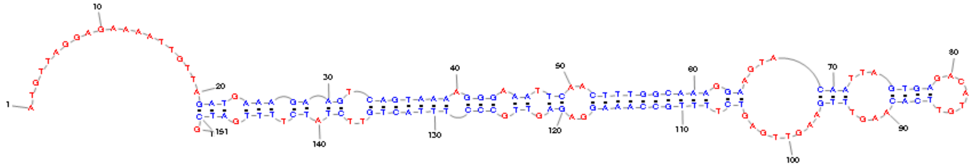	3'-UTR
miR390	27,535,097	27,535,117	+	AAGCTCAGGAGGGATAGCGCC	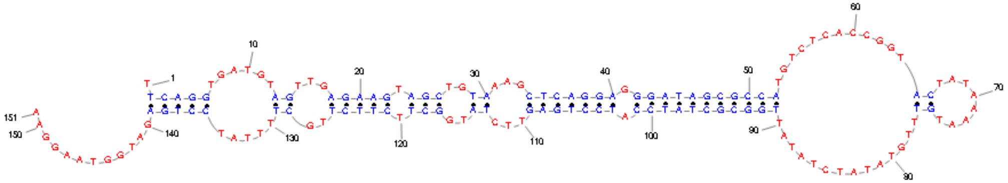	EX
miR172	27,749,476	27,749,457	-	GAATCTTGATGATGCTGCAT	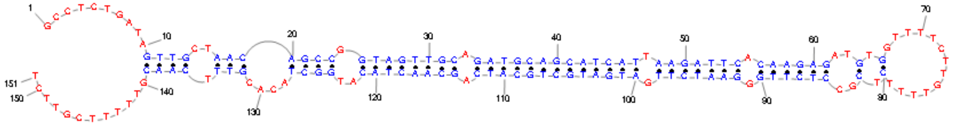	IG
miR172	27,749,514	27,749,533	+	GAATCTTAATGATGCTGCAT	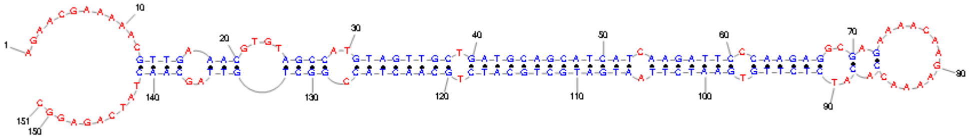	IG
miR172	27,749,531	27,749,512	-	GCAGCATCATTAAGATTCAC	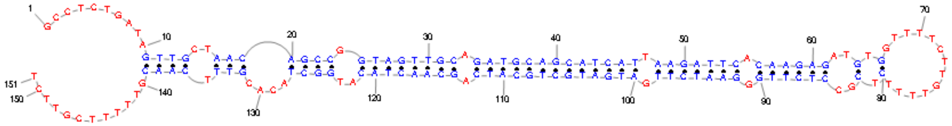	IG
miR391	28,025,453	28,025,473	+	TTCGCAGGAGAGATAGCGCCA	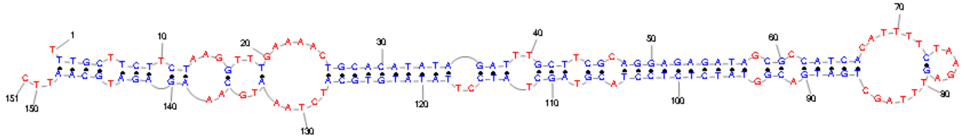	IG

miR156	30,390,534	30,390,515	-	TGACAGAAGAGAGTGAGCAC	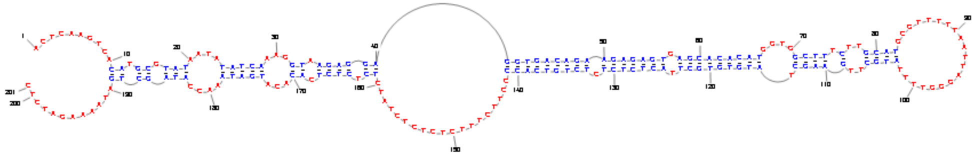	IG
miR156	30,732,505	30,732,486	-	TGACAGAAGAGAGTGAGCAC	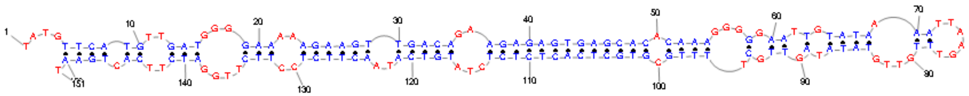	IG
miR172	31,932,285	31,932,304	+	GCAGCACCATCAAGATTCAC	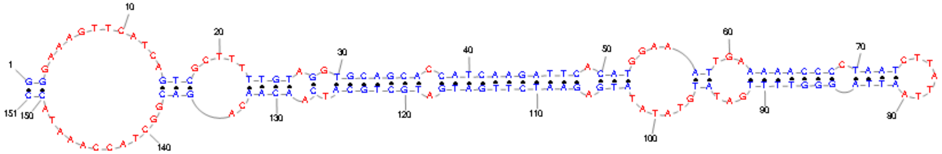	IG
miR172	31,932,360	31,932,380	+	AGAATCTTGATGATGCTGCAT	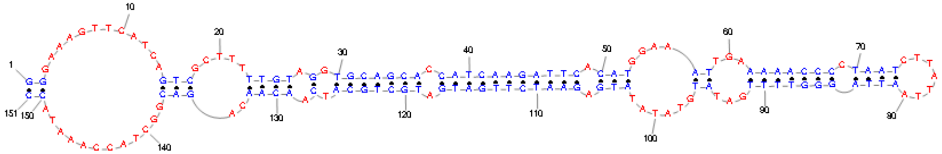	IG
miR2111*	32,495,142	32,495,122	-	ATCCTCGGGATACAGATTACC	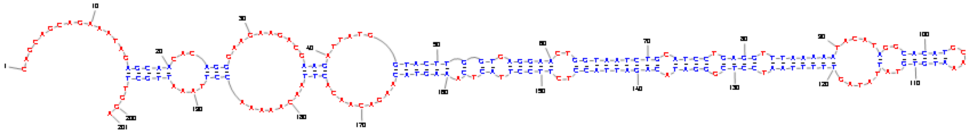	IG
miR2111	32,495,203	32,495,183	-	TAATCTGCATCCTGAGGTTTA	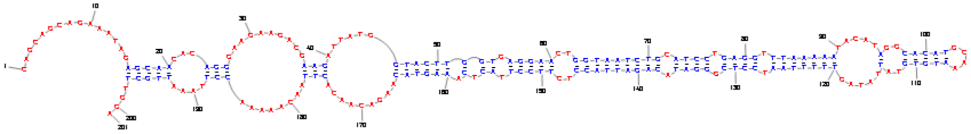	IG

The name, sequence, predicted precursor structure, and locus annotation are given. IG, intergenic; EX, exon; UTR, untranslated region; + or -, sense or antisense strand.

Table S4. Synteny alignment between *B. rapa* chromosome A3 and the *A. thaliana* genome along with mapping of AK genome blocks.

B. rapa gene	*A. thaliana* gene	BLASTP E-value	Coverage (%)	AK Block
BraA03g70580				R
BraA03g70570				
BraA03g70560	AT5G02000	1.0E-38	97%	
BraA03g70550	AT5G16900	7.0E-61	35%	
BraA03g70540	AT5G16000	6.0E-64	38%	
BraA03g70530	AT5G20920	4.0E-30	57%	
BraA03g70520				
BraA03g70510	AT5G04510	1.0E-08	40%	
BraA03g70500				
BraA03g70490	AT5G14010	7.0E-11	25%	
BraA03g70480				
BraA03g70470	AT5G16880	2.0E-27	46%	
BraA03g70460	AT5G20640	6.0E-13	84%	
BraA03g70450	AT5G07670	1.0E-09	54%	
BraA03g70440	AT5G03190	3.0E-26	84%	
BraA03g70430	AT5G22900	5.0E-83	95%	
BraA03g70420				
BraA03g70410				
BraA03g70400	AT5G05380	3.0E-52	87%	
BraA03g70390				
BraA03g70380	AT5G06700	8.0E-67	75%	
BraA03g70370	AT5G16380	8.0E-10	62%	
BraA03g70360				
BraA03g70350				
BraA03g70340				
BraA03g70330				
BraA03g70320	AT5G06740	9.0E-89	88%	
BraA03g70310	AT5G06740	5.0E-94	90%	
BraA03g70300	AT5G05280	4.0E-05	19%	
BraA03g70290				
BraA03g70280	AT5G07320	2.0E-33	64%	
BraA03g70270	AT5G04460	1.0E-05	12%	
BraA03g70260				
BraA03g70250				
BraA03g70240	AT5G03680	2.0E-08	42%	
BraA03g70230	AT5G20590	2.0E-67	71%	
BraA03g70220				
BraA03g70210	AT5G19760	3.0E-25	90%	
BraA03g70200				
BraA03g70190				
BraA03g70180				
BraA03g70170	AT5G20830	1.0E-05	18%	
BraA03g70160	AT5G12100	9.0E-65	76%	
BraA03g70150	AT5G03140	3.0E-15	57%	
BraA03g70140	AT5G05830	1.0E-35	64%	
BraA03g70130	AT5G05390	3.0E-49	57%	
BraA03g70120	AT5G05390	1.0E-144	96%	
BraA03g70110				
BraA03g70100	AT5G02020	1.0E-48	99%	
BraA03g70090	AT5G08420	6.0E-05	95%	
BraA03g70080	AT5G08420	5.0E-45	87%	
BraA03g70070	AT5G02030	0.0E+00	99%	
BraA03g70060	AT5G02040	7.0E-99	100%	

BraA03g70050	AT5G02050	8.0E-106	99%
BraA03g70040			
BraA03g70030	AT5G02060	2.0E-30	92%
BraA03g70020	AT5G02090	6.0E-22	92%
BraA03g70010	AT5G02110	7.0E-121	100%
BraA03g70000	AT5G02120	1.0E-44	87%
BraA03g69990	AT5G02130	0.0E+00	100%
BraA03g69980	AT5G02170	0.0E+00	62%
BraA03g69970	AT5G02170	0.0E+00	100%
BraA03g69960	AT5G20510	1.0E-09	22%
BraA03g69950	AT3G20650	5.0E-32	86%
BraA03g69940	AT5G02260	9.0E-139	100%
BraA03g69930	AT5G02270	1.0E-123	100%
BraA03g69920	AT5G02280	5.0E-82	99%
BraA03g69910	AT5G02290	0.0E+00	100%
BraA03g69900	AT5G02310	0.0E+00	100%
BraA03g69890			
BraA03g69880			
BraA03g69870	AT5G02370	9.0E-149	66%
BraA03g69860	AT5G02380	7.0E-22	69%
BraA03g69850	AT5G02390	0.0E+00	100%
BraA03g69840	AT5G02410	0.0E+00	100%
BraA03g69830	AT5G02420	9.0E-44	99%
BraA03g69820	AT5G02430	0.0E+00	100%
BraA03g69810	AT5G02460	2.0E-126	96%
BraA03g69800	AT5G02470	5.0E-137	99%
BraA03g69790	AT5G02480	0.0E+00	98%
BraA03g69780	AT5G02530	4.0E-90	73%
BraA03g69770	AT5G02540	2.0E-176	99%
BraA03g69760	AT5G02550	7.0E-17	99%
BraA03g69750	AT5G02580	4.0E-47	99%
BraA03g69740			
BraA03g69730	AT5G03370	7.0E-48	98%
BraA03g69720	AT5G03340	0.0E+00	100%
BraA03g69710	AT5G03330	4.0E-169	100%
BraA03g69700	AT5G03300	0.0E+00	100%
BraA03g69690	AT5G03290	0.0E+00	100%
BraA03g69680	AT5G03250	0.0E+00	100%
BraA03g69670	AT5G03230	8.0E-67	99%
BraA03g69660			
BraA03g69650			
BraA03g69640	AT5G03210	1.0E-15	49%
BraA03g69630			
BraA03g69620	AT5G03170	3.0E-108	100%
BraA03g69610	AT5G03150	0.0E+00	100%
BraA03g69600	AT5G03140	0.0E+00	100%
BraA03g69590	AT5G03120	4.0E-26	87%
BraA03g69580	AT5G03780	4.0E-21	69%
BraA03g69570	AT5G03040	3.0E-104	67%
BraA03g69560	AT5G02960	2.0E-78	99%
BraA03g69550	AT5G02910	2.0E-86	84%
BraA03g69540	AT5G02890	4.0E-138	99%
BraA03g69530	AT5G02880	0.0E+00	100%
BraA03g69520	AT5G02870	0.0E+00	71%
BraA03g69510	AT5G02760	8.0E-179	100%
BraA03g69500	AT5G02840	2.0E-29	91%
BraA03g69490	AT5G02700	5.0E-120	91%

BraA03g69480			
BraA03g69470	AT5G02660	1.0E-19	65%
BraA03g69460	AT5G02840	6.0E-128	96%
BraA03g69450	AT5G04280	2.0E-09	44%
BraA03g69440	AT5G02640	5.0E-60	99%
BraA03g69430	AT5G02630	2.0E-126	97%
BraA03g69420	AT5G02590	4.0E-139	100%
BraA03g69410			
BraA03g69400			
BraA03g69390	AT5G03380	3.0E-128	99%
BraA03g69380	AT5G03415	1.0E-134	93%
BraA03g69370	AT5G03430	0.0E+00	100%
BraA03g69360	AT5G03455	2.0E-72	98%
BraA03g69350			
BraA03g69340	AT5G03510	7.0E-114	100%
BraA03g69330	AT5G03520	1.0E-116	100%
BraA03g69320	AT5G03555	0.0E+00	62%
BraA03g69310	AT5G03560	1.0E-104	100%
BraA03g69300	AT5G03570	0.0E+00	98%
BraA03g69290	AT5G03580	4.0E-34	94%
BraA03g69280	AT5G04350	1.0E-21	90%
BraA03g69270	AT5G04350	1.0E-20	90%
BraA03g69260	AT5G03630	0.0E+00	100%
BraA03g69250			
BraA03g69240	AT5G03670	0.0E+00	100%
BraA03g69230			
BraA03g69220			
BraA03g69210	AT5G03680	0.0E+00	100%
BraA03g69200	AT5G03690	5.0E-29	81%
BraA03g69190	AT5G03730	0.0E+00	100%
BraA03g69180	AT5G03760	0.0E+00	100%
BraA03g69170	AT5G03790	1.0E-99	100%
BraA03g69160	AT5G03795	0.0E+00	100%
BraA03g69150	AT5G03810	3.0E-68	97%
BraA03g69140	AT5G03810	2.0E-170	99%
BraA03g69130	AT5G03840	4.0E-98	99%
BraA03g69120	AT5G03850	4.0E-32	98%
BraA03g69110	AT5G17470	4.0E-50	99%
BraA03g69100	AT5G03870	4.0E-142	84%
BraA03g69090	AT5G03880	1.0E-153	100%
BraA03g69080	AT5G03890	3.0E-58	99%
BraA03g69070	AT5G04040	0.0E+00	99%
BraA03g69060			
BraA03g69050	AT5G04040	5.0E-68	56%
BraA03g69040	AT5G04080	1.0E-06	75%
BraA03g69030			
BraA03g69020			
BraA03g69010	AT5G04130	2.0E-43	98%
BraA03g69000	AT5G04130	3.0E-138	100%
BraA03g68990	AT5G04110	1.0E-164	69%
BraA03g68980	AT5G04150	6.0E-81	84%
BraA03g68970			
BraA03g68960	AT5G04160	2.0E-145	99%
BraA03g68950	AT5G04170	6.0E-135	100%
BraA03g68940	AT5G03880	1.0E-153	100%
BraA03g68930	AT5G04045	6.0E-18	97%
BraA03g68920	AT5G03890	3.0E-58	99%

BraA03g68910	AT5G04040	0.0E+00	99%
BraA03g68900	AT5G04040	5.0E-68	56%
BraA03g68890	AT5G04045	3.0E-23	99%
BraA03g68880	AT5G04080	5.0E-15	75%
BraA03g68870			
BraA03g68860			
BraA03g68850	AT5G04110	6.0E-156	97%
BraA03g68840	AT5G04130	0.0E+00	100%
BraA03g68830			
BraA03g68820			
BraA03g68810	AT5G04150	9.0E-81	92%
BraA03g68800			
BraA03g68790	AT5G04160	2.0E-145	99%
BraA03g68780	AT5G04170	6.0E-135	100%
BraA03g68770	AT5G04250	5.0E-158	100%
BraA03g68760	AT5G18880	2.0E-08	91%
BraA03g68750	AT5G04280	5.0E-125	100%
BraA03g68740	AT5G04310	6.0E-23	91%
BraA03g68730	AT5G04320	2.0E-117	82%
BraA03g68720	AT5G04347	1.0E-41	96%
BraA03g68710	AT5G04370	0.0E+00	96%
BraA03g68700	AT5G04347	2.0E-35	96%
BraA03g68690	AT5G04390	5.0E-152	100%
BraA03g68680	AT5G04410	0.0E+00	100%
BraA03g68670	AT5G04420	0.0E+00	100%
BraA03g68660	AT5G04430	9.0E-162	100%
BraA03g68650	AT5G04440	2.0E-39	99%
BraA03g68640			
BraA03g68630	AT5G04470	4.0E-29	63%
BraA03g68620	AT5G04490	1.0E-111	96%
BraA03g68610	AT5G04520	1.0E-157	100%
BraA03g68600	AT5G04660	0.0E+00	99%
BraA03g68590	AT5G04670	0.0E+00	98%
BraA03g68580	AT5G02600	2.0E-13	83%
BraA03g68570	AT5G04700	0.0E+00	100%
BraA03g68560	AT5G04730	0.0E+00	94%
BraA03g68550	AT5G04740	6.0E-147	100%
BraA03g68540	AT5G04750	1.0E-30	99%
BraA03g68530	AT5G04760	4.0E-103	100%
BraA03g68520	AT5G04770	0.0E+00	100%
BraA03g68510	AT5G04800	2.0E-66	99%
BraA03g68500	AT5G04780	0.0E+00	100%
BraA03g68490	AT5G04830	2.0E-92	91%
BraA03g68480			
BraA03g68470			
BraA03g68460			
BraA03g68450	AT5G04850	5.0E-33	97%
BraA03g68440	AT5G04870	4.0E-172	94%
BraA03g68430	AT5G04885	0.0E+00	99%
BraA03g68420	AT5G04890	2.0E-97	97%
BraA03g68410	AT5G04900	0.0E+00	100%
BraA03g68400			
BraA03g68390	AT5G04930	0.0E+00	100%
BraA03g68380	AT5G04940	0.0E+00	100%
BraA03g68370			
BraA03g68360	AT5G04950	9.0E-179	100%
BraA03g68350	AT5G26770	5.0E-05	83%

BraA03g68340	AT5G04960	0.0E+00	100%
BraA03g68330	AT5G04970	0.0E+00	91%
BraA03g68320	AT5G04980	0.0E+00	93%
BraA03g68310	AT5G05000	3.0E-165	100%
BraA03g68300	AT5G05010	0.0E+00	100%
BraA03g68290	AT5G05070	0.0E+00	98%
BraA03g68280			
BraA03g68270	AT5G05080	1.0E-131	100%
BraA03g68260	AT5G05090	3.0E-80	94%
BraA03g68250	AT5G05100	3.0E-132	100%
BraA03g68240	AT5G05110	9.0E-108	100%
BraA03g68230	AT5G05120	1.0E-42	91%
BraA03g68220	AT5G05030	2.0E-107	100%
BraA03g68210	AT5G05140	8.0E-163	99%
BraA03g68200			
BraA03g68190	AT5G05690	8.0E-34	88%
BraA03g68180	AT5G05150	8.0E-54	79%
BraA03g68170	AT5G05170	0.0E+00	100%
BraA03g68160	AT5G05180	2.0E-121	93%
BraA03g68150	AT5G05190	9.0E-167	74%
BraA03g68140	AT5G05200	0.0E+00	100%
BraA03g68130	AT5G05210	3.0E-148	99%
BraA03g68120	AT5G05340	1.0E-135	100%
BraA03g68110	AT5G05370	4.0E-36	99%
BraA03g68100			
BraA03g68090	AT5G05410	1.0E-110	99%
BraA03g68080	AT5G05440	2.0E-109	100%
BraA03g68070	AT5G05460	0.0E+00	97%
BraA03g68060	AT5G05480	0.0E+00	97%
BraA03g68050	AT5G08420	5.0E-05	29%
BraA03g68040	AT5G05560	0.0E+00	100%
BraA03g68030	AT5G10340	3.0E-05	48%
BraA03g68020	AT5G05530	8.0E-39	98%
BraA03g68010	AT5G05600	6.0E-91	96%
BraA03g68000	AT5G05600	1.0E-86	99%
BraA03g67990	AT5G05610	4.0E-30	73%
BraA03g67980	AT5G05670	3.0E-140	86%
BraA03g67970	AT5G05690	0.0E+00	98%
BraA03g67960	AT5G05730	0.0E+00	100%
BraA03g67950	AT5G05740	0.0E+00	100%
BraA03g67940	AT5G05760	7.0E-128	98%
BraA03g67930	AT5G05780	4.0E-178	100%
BraA03g67920	AT5G05820	3.0E-164	100%
BraA03g67910	AT5G05900	7.0E-133	88%
BraA03g67900	AT5G07990	7.0E-72	98%
BraA03g67890	AT5G05980	0.0E+00	89%
BraA03g67880	AT5G05987	2.0E-114	100%
BraA03g67870	AT5G05990	2.0E-109	99%
BraA03g67860			
BraA03g67850	AT5G06070	3.0E-98	100%
BraA03g67840	AT5G06110	2.0E-173	97%
BraA03g67830	AT5G07010	7.0E-75	98%
BraA03g67820	AT5G06110	7.0E-85	77%
BraA03g67810	AT5G07010	4.0E-72	98%
BraA03g67800	AT5G07010	1.0E-47	99%
BraA03g67790	AT5G06150	0.0E+00	99%
BraA03g67780	AT5G08390	5.0E-14	31%

BraA03g67770	AT5G06160	0.0E+00	100%
BraA03g67760	AT5G06170	0.0E+00	98%
BraA03g67750			
BraA03g67740	AT5G06220	0.0E+00	97%
BraA03g67730	AT5G06240	2.0E-56	60%
BraA03g67720			
BraA03g67710	AT5G06250	8.0E-84	92%
BraA03g67700	AT5G04800	1.0E-66	98%
BraA03g67690	AT5G06270	7.0E-42	99%
BraA03g67680			
BraA03g67670	AT5G06280	2.0E-46	99%
BraA03g67660	AT5G06320	6.0E-99	97%
BraA03g67650	AT5G06330	2.0E-59	100%
BraA03g67640	AT5G06370	6.0E-144	100%
BraA03g67630	AT5G06390	0.0E+00	100%
BraA03g67620	AT5G06410	4.0E-108	100%
BraA03g67610	AT5G06440	4.0E-33	51%
BraA03g67600	AT5G06450	3.0E-94	99%
BraA03g67590	AT5G06470	6.0E-115	100%
BraA03g67580	AT5G06570	3.0E-155	99%
BraA03g67570	AT5G06600	0.0E+00	100%
BraA03g67560	AT5G06610	0.0E+00	98%
BraA03g67550			
BraA03g67540			
BraA03g67530	AT5G03770	1.0E-48	52%
BraA03g67520	AT5G03480	6.0E-27	68%
BraA03g67510	AT5G03495	3.0E-07	56%
BraA03g67500	AT5G06700	0.0E+00	100%
BraA03g67490	AT5G06710	4.0E-131	100%
BraA03g67480	AT5G03480	3.0E-27	76%
BraA03g67470	AT5G03480	6.0E-28	65%
BraA03g67460			
BraA03g67450	AT5G06760	1.0E-55	90%
BraA03g67440	AT5G06800	3.0E-154	99%
BraA03g67430			
BraA03g67420	AT5G06820	0.0E+00	100%
BraA03g67410	AT5G06830	3.0E-12	39%
BraA03g67400	AT5G06839	0.0E+00	97%
BraA03g67390	AT5G06850	0.0E+00	84%
BraA03g67380	AT5G06860	1.0E-132	89%
BraA03g67370			
BraA03g67360	AT5G06860	2.0E-63	89%
BraA03g67350	AT5G06860	3.0E-74	86%
BraA03g67340			
BraA03g67330	AT5G06860	4.0E-62	88%
BraA03g67320	AT5G06860	3.0E-127	94%
BraA03g67310	AT5G06520	1.0E-09	56%
BraA03g67300	AT5G06920	4.0E-147	100%
BraA03g67290			
BraA03g67280	AT5G07010	2.0E-175	99%
BraA03g67270			
BraA03g67260	AT5G07030	0.0E+00	100%
BraA03g67250	AT5G07090	5.0E-150	100%
BraA03g67240	AT5G07110	5.0E-81	88%
BraA03g67230	AT5G07120	0.0E+00	100%
BraA03g67220			
BraA03g67210	AT5G07170	6.0E-136	99%

BraA03g67200	AT5G07190	6.0E-34	95%
BraA03g67190	AT5G07200	1.0E-170	98%
BraA03g67180	AT5G07210	1.0E-165	57%
BraA03g67170	AT5G07240	4.0E-115	97%
BraA03g67160	AT5G07250	1.0E-132	100%
BraA03g67150	AT5G07300	0.0E+00	98%
BraA03g67140	AT5G07310	7.0E-30	45%
BraA03g67130	AT5G07350	0.0E+00	100%
BraA03g67120	AT5G07430	0.0E+00	100%
BraA03g67110	AT5G07440	0.0E+00	100%
BraA03g67100	AT5G07460	8.0E-108	100%
BraA03g67090	AT5G07470	1.0E-80	97%
BraA03g67080	AT5G07470	5.0E-83	97%
BraA03g67070			
BraA03g67060			
BraA03g67050			
BraA03g67040	AT5G07470	1.0E-81	97%
BraA03g67030	AT5G07470	2.0E-83	97%
BraA03g67020	AT5G07475	4.0E-88	97%
BraA03g67010	AT5G07490	2.0E-74	99%
BraA03g67000	AT5G07530	2.0E-37	39%
BraA03g66990	AT5G07530	8.0E-31	46%
BraA03g66980			
BraA03g66970	AT5G07550	3.0E-25	66%
BraA03g66960	AT5G07560	3.0E-28	92%
BraA03g66950	AT5G07560	1.0E-23	75%
BraA03g66940	AT5G07610	9.0E-52	96%
BraA03g66930	AT5G07610	5.0E-150	100%
BraA03g66920	AT5G07620	1.0E-145	92%
BraA03g66910	AT5G03480	2.0E-25	90%
BraA03g66900	AT5G07690	1.0E-46	92%
BraA03g66890	AT5G07690	8.0E-50	99%
BraA03g66880	AT5G03495	1.0E-27	75%
BraA03g66870	AT5G07730	9.0E-67	100%
BraA03g66860	AT5G07770	2.0E-141	26%
BraA03g66850	AT5G07790	4.0E-111	100%
BraA03g66840	AT5G07810	0.0E+00	99%
BraA03g66830	AT5G07820	2.0E-113	100%
BraA03g66820			
BraA03g66810			
BraA03g66800	AT5G07870	0.0E+00	98%
BraA03g66790	AT5G07880	4.0E-93	95%
BraA03g66780	AT5G07890	2.0E-161	94%
BraA03g66770	AT5G07910	3.0E-127	100%
BraA03g66760	AT5G07920	0.0E+00	100%
BraA03g66750	AT5G08020	0.0E+00	98%
BraA03g66740	AT5G08040	3.0E-19	98%
BraA03g66730	AT5G08050	2.0E-68	99%
BraA03g66720	AT5G08060	7.0E-64	99%
BraA03g66710			
BraA03g66700	AT5G08070	4.0E-75	98%
BraA03g66690	AT5G08080	8.0E-144	100%
BraA03g66680	AT5G08130	7.0E-145	77%
BraA03g66670	AT5G08139	2.0E-72	98%
BraA03g66660	AT5G08141	3.0E-18	77%
BraA03g66650	AT5G08160	0.0E+00	100%
BraA03g66640	AT5G08170	0.0E+00	100%

BraA03g66630	AT5G08180	2.0E-78	99%
BraA03g66620	AT5G08190	4.0E-56	88%
BraA03g66610	AT5G08200	1.0E-172	100%
BraA03g66600	AT5G08240	4.0E-73	98%
BraA03g66590	AT5G08260	2.0E-07	44%
BraA03g66580	AT5G08260	0.0E+00	100%
BraA03g66570	AT5G08280	0.0E+00	100%
BraA03g66560	AT5G08290	1.0E-20	88%
BraA03g66550	AT5G21150	1.0E-54	92%
BraA03g66540	AT5G08290	3.0E-23	98%
BraA03g66530	AT5G21150	6.0E-54	62%
BraA03g66520	AT5G08300	0.0E+00	100%
BraA03g66510	AT5G08310	0.0E+00	90%
BraA03g66500	AT5G08330	2.0E-88	100%
BraA03g66490	AT5G08335	1.0E-100	99%
BraA03g66480	AT5G08340	4.0E-174	100%
BraA03g66470	AT5G08350	7.0E-100	100%
BraA03g66460	AT5G08400	3.0E-95	100%
BraA03g66450	AT5G08410	2.0E-74	98%
BraA03g66440	AT5G08415	0.0E+00	100%
BraA03g66430	AT5G05800	8.0E-06	37%
BraA03g66420	AT5G08450	0.0E+00	100%
BraA03g66410	AT5G08480	1.0E-57	99%
BraA03g66400	AT5G08490	0.0E+00	95%
BraA03g66390	AT5G08520	4.0E-142	100%
BraA03g66380	AT5G08530	0.0E+00	100%
BraA03g66370			
BraA03g66360			
BraA03g66350			
BraA03g66340			
BraA03g66330	AT5G08540	2.0E-134	99%
BraA03g66320			
BraA03g66310			
BraA03g66300			
BraA03g66290			
BraA03g66280			
BraA03g66270	AT5G08550	1.0E-156	99%
BraA03g66260	AT5G08550	3.0E-130	96%
BraA03g66250	AT5G08560	0.0E+00	68%
BraA03g66240	AT5G16750	9.0E-08	53%
BraA03g66230	AT5G08565	7.0E-21	5%
BraA03g66220	AT5G08565	2.0E-59	99%
BraA03g66210	AT5G08570	0.0E+00	100%
BraA03g66200	AT5G08580	0.0E+00	100%
BraA03g66190	AT5G08590	0.0E+00	98%
BraA03g66180	AT5G02700	3.0E-103	99%
BraA03g66170	AT5G08630	0.0E+00	100%
BraA03g66160	AT5G08650	1.0E-57	41%
BraA03g66150	AT5G08680	0.0E+00	96%
BraA03g66140	AT5G08680	0.0E+00	96%
BraA03g66130	AT5G08790	2.0E-146	100%
BraA03g66120	AT5G09220	0.0E+00	100%
BraA03g66110	AT5G09300	0.0E+00	100%
BraA03g66100	AT5G09310	1.0E-62	98%
BraA03g66090	AT5G09330	3.0E-152	91%
BraA03g66080	AT5G09400	0.0E+00	100%
BraA03g66070	AT5G09430	3.0E-180	100%

BraA03g66060	AT5G09470	7.0E-80	99%
BraA03g66050	AT5G09480	7.0E-17	89%
BraA03g66040	AT5G09500	4.0E-77	99%
BraA03g66030	AT5G09530	2.0E-74	98%
BraA03g66020	AT5G09550	0.0E+00	100%
BraA03g66010	AT5G09590	0.0E+00	100%
BraA03g66000	AT5G09630	5.0E-104	99%
BraA03g65990	AT5G09650	8.0E-159	100%
BraA03g65980	AT5G19270	6.0E-07	27%
BraA03g65970			
BraA03g65960	AT5G09760	0.0E+00	94%
BraA03g65950	AT5G09770	9.0E-81	97%
BraA03g65940	AT5G06820	1.0E-06	88%
BraA03g65930	AT5G06820	2.0E-05	87%
BraA03g65920			
BraA03g65910	AT5G09805	4.0E-32	94%
BraA03g65900			
BraA03g65890	AT5G09810	0.0E+00	100%
BraA03g65880	AT5G09820	3.0E-94	100%
BraA03g65870	AT5G09850	3.0E-163	100%
BraA03g65860	AT5G09870	0.0E+00	91%
BraA03g65850	AT5G09876	5.0E-22	97%
BraA03g65840	AT5G09900	0.0E+00	100%
BraA03g65830	AT5G09920	2.0E-76	99%
BraA03g65820	AT5G09940	8.0E-54	99%
BraA03g65810	AT5G09980	3.0E-13	90%
BraA03g65800	AT5G10010	2.0E-157	100%
BraA03g65790	AT5G10020	0.0E+00	97%
BraA03g65780	AT5G10050	3.0E-153	99%
BraA03g65770	AT5G10060	0.0E+00	100%
BraA03g65760	AT5G10090	0.0E+00	98%
BraA03g65750	AT5G10100	2.0E-129	75%
BraA03g65740	AT5G10110	1.0E-86	100%
BraA03g65730	AT5G10120	2.0E-180	100%
BraA03g65720	AT5G10140	1.0E-79	99%
BraA03g65710	AT5G10160	2.0E-112	99%
BraA03g65700	AT5G10240	0.0E+00	99%
BraA03g65690	AT5G10260	2.0E-112	100%
BraA03g65680	AT5G10280	4.0E-68	99%
BraA03g65670	AT5G10360	4.0E-138	100%
BraA03g65660	AT5G10370	0.0E+00	100%
BraA03g65650	AT5G10400	5.0E-75	99%
BraA03g65640	AT5G10420	1.0E-80	100%
BraA03g65630	AT5G10420	1.0E-100	100%
BraA03g65620	AT5G10420	9.0E-12	76%
BraA03g65610	AT5G10420	4.0E-16	42%
BraA03g65600	AT5G10420	0.0E+00	99%
BraA03g65590			
BraA03g65580	AT5G10450	1.0E-136	99%
BraA03g65570	AT5G10470	0.0E+00	100%
BraA03g65560	AT5G10720	0.0E+00	100%
BraA03g65550	AT5G10720	7.0E-169	91%
BraA03g65540			
BraA03g65530	AT5G10710	2.0E-144	99%
BraA03g65520			
BraA03g65510	AT5G10700	1.0E-84	99%
BraA03g65500	AT5G10660	3.0E-104	100%

BraA03g65490			
BraA03g65480	AT5G10650	0.0E+00	100%
BraA03g65470	AT5G10630	2.0E-155	100%
BraA03g65460	AT5G11200	3.0E-87	99%
BraA03g65450	AT5G11110	3.0E-70	98%
BraA03g65440	AT5G11110	0.0E+00	100%
BraA03g65430			
BraA03g65420			
BraA03g65410	AT5G11090	8.0E-81	97%
BraA03g65400			
BraA03g65390	AT5G11070	2.0E-67	99%
BraA03g65380	AT5G11060	9.0E-159	96%
BraA03g65370	AT5G10980	1.0E-75	99%
BraA03g65360	AT5G10960	5.0E-152	100%
BraA03g65350	AT5G10950	5.0E-33	87%
BraA03g65340	AT5G05810	1.0E-07	14%
BraA03g65330	AT5G10840	0.0E+00	100%
BraA03g65320	AT5G10840	0.0E+00	99%
BraA03g65310	AT5G10780	1.0E-71	99%
BraA03g65300	AT5G10750	5.0E-162	100%
BraA03g65290	AT5G10745	9.0E-24	98%
BraA03g65280	AT5G10550	0.0E+00	100%
BraA03g65270	AT5G10540	0.0E+00	100%
BraA03g65260			
BraA03g65250	AT5G11200	7.0E-25	87%
BraA03g65240			
BraA03g65230	AT5G11230	6.0E-123	99%
BraA03g65220	AT5G11250	4.0E-32	98%
BraA03g65210	AT5G11250	0.0E+00	100%
BraA03g65200	AT5G11250	7.0E-154	88%
BraA03g65190			
BraA03g65180	AT5G11410	7.0E-120	100%
BraA03g65170	AT5G11440	7.0E-59	99%
BraA03g65160	AT5G11470	0.0E+00	99%
BraA03g65150			
BraA03g65140			
BraA03g65130	AT5G11480	2.0E-145	100%
BraA03g65120	AT5G11490	0.0E+00	100%
BraA03g65110	AT5G11500	3.0E-84	92%
BraA03g65100	AT5G11510	0.0E+00	97%
BraA03g65090	AT5G11520	0.0E+00	100%
BraA03g65080			
BraA03g65070	AT5G11530	0.0E+00	99%
BraA03g65060	AT5G05170	4.0E-05	20%
BraA03g65050	AT5G11550	4.0E-29	96%
BraA03g65040	AT5G11550	7.0E-76	99%
BraA03g65030	AT5G11560	6.0E-29	37%
BraA03g65020	AT5G11670	0.0E+00	100%
BraA03g65010	AT5G11680	2.0E-112	100%
BraA03g65000	AT5G11690	7.0E-46	97%
BraA03g64990	AT5G11700	0.0E+00	100%
BraA03g64980	AT5G11720	0.0E+00	100%
BraA03g64970			
BraA03g64960	AT5G11480	2.0E-145	100%
BraA03g64950	AT5G11490	0.0E+00	100%
BraA03g64940	AT5G11500	2.0E-124	100%
BraA03g64930	AT5G11510	0.0E+00	97%

BraA03g64920	AT5G11520	0.0E+00	100%
BraA03g64910			
BraA03g64900	AT5G11530	0.0E+00	99%
BraA03g64890	AT5G05170	4.0E-05	20%
BraA03g64880	AT5G11550	4.0E-29	96%
BraA03g64870	AT5G11550	4.0E-75	99%
BraA03g64860	AT5G11560	6.0E-29	37%
BraA03g64850	AT5G11670	0.0E+00	100%
BraA03g64840	AT5G11680	2.0E-112	100%
BraA03g64830	AT5G11690	7.0E-46	97%
BraA03g64820	AT5G11700	0.0E+00	100%
BraA03g64810	AT5G11720	0.0E+00	100%
BraA03g64800	AT5G08730	4.0E-51	87%
BraA03g64790	AT5G11730	0.0E+00	100%
BraA03g64780	AT5G11730	3.0E-75	98%
BraA03g64770	AT5G11730	3.0E-45	85%
BraA03g64760	AT5G11730	4.0E-42	99%
BraA03g64750	AT5G11740	6.0E-12	98%
BraA03g64740	AT5G11750	2.0E-96	100%
BraA03g64730	AT5G04250	3.0E-36	73%
BraA03g64720	AT5G11770	2.0E-113	100%
BraA03g64710	AT5G11790	0.0E+00	100%
BraA03g64700	AT5G11810	5.0E-147	100%
BraA03g64690	AT5G11830	1.0E-67	97%
BraA03g64680	AT5G11840	6.0E-138	96%
BraA03g64670	AT5G11850	0.0E+00	96%
BraA03g64660			
BraA03g64650	AT5G03480	2.0E-14	85%
BraA03g64640	AT5G11860	1.0E-156	100%
BraA03g64630	AT5G11880	0.0E+00	100%
BraA03g64620	AT5G11890	1.0E-111	100%
BraA03g64610	AT5G11900	5.0E-102	100%
BraA03g64600	AT5G11910	4.0E-142	100%
BraA03g64590			
BraA03g64580	AT5G11920	5.0E-138	98%
BraA03g64570	AT5G11920	1.0E-76	86%
BraA03g64560	AT5G11920	1.0E-81	89%
BraA03g64550	AT5G11930	2.0E-53	99%
BraA03g64540	AT5G11940	2.0E-34	62%
BraA03g64530			
BraA03g64520			
BraA03g64510	AT5G11970	4.0E-47	99%
BraA03g64500	AT5G11980	0.0E+00	99%
BraA03g64490	AT5G12020	2.0E-70	99%
BraA03g64480	AT5G12040	0.0E+00	100%
BraA03g64470	AT5G12050	2.0E-110	100%
BraA03g64460			
BraA03g64450			
BraA03g64440	AT5G12070	9.0E-50	99%
BraA03g64430	AT5G12080	0.0E+00	100%
BraA03g64420	AT5G12120	0.0E+00	100%
BraA03g64410	AT5G12180	9.0E-178	88%
BraA03g64400	AT5G12190	3.0E-67	99%
BraA03g64390			
BraA03g64380	AT5G11250	4.0E-109	96%
BraA03g64370	AT5G12220	0.0E+00	100%
BraA03g64360	AT5G12230	5.0E-65	100%

BraA03g64350	AT5G12235	9.0E-27	99%
BraA03g64340	AT5G12240	8.0E-22	42%
BraA03g64330	AT5G12260	0.0E+00	100%
BraA03g64320	AT5G12290	0.0E+00	100%
BraA03g64310	AT5G18880	4.0E-22	81%
BraA03g64300			
BraA03g64290			
BraA03g64280			
BraA03g64270	AT5G14160	1.0E-37	97%
BraA03g64260			
BraA03g64250	AT5G12310	3.0E-99	100%
BraA03g64240	AT5G12370	0.0E+00	99%
BraA03g64230	AT5G12400	0.0E+00	97%
BraA03g64220	AT5G12410	8.0E-164	99%
BraA03g64210	AT5G16350	0.0E+00	99%
BraA03g64200	AT5G12420	0.0E+00	99%
BraA03g64190	AT5G12420	3.0E-94	95%
BraA03g64180	AT5G12440	0.0E+00	99%
BraA03g64170	AT5G12460	7.0E-42	99%
BraA03g64160	AT5G12470	1.0E-177	100%
BraA03g64150	AT5G12840	2.0E-103	98%
BraA03g64140	AT5G12870	4.0E-74	91%
BraA03g64130	AT5G12870	4.0E-48	88%
BraA03g64120	AT5G12900	0.0E+00	100%
BraA03g64110	AT5G12920	0.0E+00	94%
BraA03g64100	AT5G12930	7.0E-172	100%
BraA03g64090	AT5G12950	0.0E+00	84%
BraA03g64080	AT5G12970	0.0E+00	100%
BraA03g64070	AT5G12980	6.0E-120	100%
BraA03g64060	AT5G13020	0.0E+00	100%
BraA03g64050	AT5G15690	5.0E-04	69%
BraA03g64040	AT5G13030	0.0E+00	100%
BraA03g64030			
BraA03g64020	AT5G13080	3.0E-63	99%
BraA03g64010	AT5G13090	1.0E-60	100%
BraA03g64000			
BraA03g63990	AT5G13100	4.0E-168	100%
BraA03g63980	AT5G13110	0.0E+00	100%
BraA03g63970	AT5G13120	2.0E-126	100%
BraA03g63960	AT5G13150	0.0E+00	100%
BraA03g63950	AT5G13160	0.0E+00	99%
BraA03g63940	AT5G13170	5.0E-105	96%
BraA03g63930	AT5G13180	7.0E-114	98%
BraA03g63920	AT5G16640	8.0E-123	100%
BraA03g63910			
BraA03g63900			
BraA03g63890	AT5G13220	7.0E-54	99%
BraA03g63880			
BraA03g63870			
BraA03g63860	AT5G13250	2.0E-103	68%
BraA03g63850	AT5G13300	0.0E+00	61%
BraA03g63840	AT5G13320	0.0E+00	100%
BraA03g63830	AT5G13330	2.0E-12	33%
BraA03g63820	AT5G13370	0.0E+00	100%
BraA03g63810	AT5G13420	0.0E+00	100%
BraA03g63800	AT5G13430	4.0E-148	100%
BraA03g63790	AT5G13440	1.0E-149	100%

BraA03g63780	AT5G13450	7.0E-120	99%
BraA03g63770	AT5G13460	0.0E+00	100%
BraA03g63760	AT5G13480	4.0E-12	61%
BraA03g63750	AT5G13480	0.0E+00	100%
BraA03g63740	AT5G13490	0.0E+00	99%
BraA03g63730	AT5G13500	0.0E+00	100%
BraA03g63720	AT5G13530	0.0E+00	100%
BraA03g63710	AT5G13550	0.0E+00	100%
BraA03g63700	AT5G13560	4.0E-18	50%
BraA03g63690			
BraA03g63680	AT5G13630	0.0E+00	100%
BraA03g63670	AT5G13660	3.0E-138	100%
BraA03g63660	AT5G13700	0.0E+00	100%
BraA03g63650	AT5G13710	0.0E+00	98%
BraA03g63640	AT5G13740	0.0E+00	100%
BraA03g63630	AT5G13750	0.0E+00	100%
BraA03g63620	AT5G13790	4.0E-72	94%
BraA03g63610	AT5G13800	0.0E+00	100%
BraA03g63600	AT5G13810	2.0E-119	100%
BraA03g63590	AT5G13820	0.0E+00	95%
BraA03g63580	AT5G13840	0.0E+00	100%
BraA03g63570	AT5G13870	7.0E-168	100%
BraA03g63560	AT5G13880	5.0E-58	99%
BraA03g63550	AT5G13910	1.0E-78	98%
BraA03g63540	AT5G13920	1.0E-131	89%
BraA03g63530	AT5G13930	0.0E+00	100%
BraA03g63520	AT5G13950	7.0E-81	100%
BraA03g63510	AT5G13950	2.0E-45	59%
BraA03g63500			
BraA03g63490	AT5G13960	0.0E+00	100%
BraA03g63480	AT5G13980	0.0E+00	100%
BraA03g63470	AT5G13990	0.0E+00	100%
BraA03g63460	AT5G14000	9.0E-32	100%
BraA03g63450	AT5G14240	1.0E-58	57%
BraA03g63440	AT5G14230	0.0E+00	100%
BraA03g63430	AT5G14210	0.0E+00	100%
BraA03g63420	AT5G14170	0.0E+00	100%
BraA03g63410	AT5G14150	0.0E+00	100%
BraA03g63400	AT5G14140	2.0E-131	93%
BraA03g63390			
BraA03g63380	AT5G14120	0.0E+00	100%
BraA03g63370	AT5G14110	6.0E-33	83%
BraA03g63360			
BraA03g63350	AT5G14090	2.0E-144	100%
BraA03g63340			
BraA03g63330	AT5G14070	6.0E-67	99%
BraA03g63320	AT5G14060	0.0E+00	99%
BraA03g63310			
BraA03g63300	AT5G14040	0.0E+00	100%
BraA03g63290	AT5G14030	4.0E-93	99%
BraA03g63280	AT5G14020	0.0E+00	93%
BraA03g63270			
BraA03g63260	AT5G14010	8.0E-26	99%
BraA03g63250	AT5G14330	7.0E-23	70%
BraA03g63240	AT5G14320	1.0E-86	99%
BraA03g63230	AT5G14310	0.0E+00	100%
BraA03g63220	AT5G14250	0.0E+00	100%

BraA03g63210	AT5G14490	2.0E-18	86%
BraA03g63200	AT5G14480	0.0E+00	100%
BraA03g63190	AT5G14440	1.0E-88	95%
BraA03g63180	AT5G14430	0.0E+00	100%
BraA03g63170	AT5G14420	0.0E+00	100%
BraA03g63160	AT5G14380	2.0E-34	99%
BraA03g63150	AT5G14370	5.0E-105	99%
BraA03g63140	AT5G14360	6.0E-77	99%
BraA03g63130	AT5G14510	3.0E-163	100%
BraA03g63120	AT5G03470	7.0E-122	100%
BraA03g63110			
BraA03g63100	AT5G14580	0.0E+00	100%
BraA03g63090	AT5G14590	0.0E+00	100%
BraA03g63080	AT5G14640	0.0E+00	100%
BraA03g63070	AT5G14710	3.0E-65	73%
BraA03g63060	AT5G14720	1.0E-113	38%
BraA03g63050			
BraA03g63040	AT5G14640	2.0E-17	55%
BraA03g63030	AT5G14720	1.0E-15	57%
BraA03g63020			
BraA03g63010	AT5G21160	1.0E-45	79%
BraA03g63000			
BraA03g62990			
BraA03g62980			
BraA03g62970	AT5G14790	3.0E-147	100%
BraA03g62960	AT5G14800	3.0E-137	63%
BraA03g62950	AT5G14860	0.0E+00	100%
BraA03g62940	AT5G14910	5.0E-81	99%
BraA03g62930	AT5G14920	1.0E-34	24%
BraA03g62920	AT5G14930	3.0E-20	22%
BraA03g62910	AT5G14950	0.0E+00	98%
BraA03g62900	AT5G15020	0.0E+00	100%
BraA03g62890	AT5G15070	0.0E+00	69%
BraA03g62880	AT5G15080	0.0E+00	100%
BraA03g62870	AT5G15110	3.0E-40	92%
BraA03g62860			
BraA03g62850	AT5G15120	8.0E-133	100%
BraA03g62840			
BraA03g62830	AT5G15690	1.0E-05	28%
BraA03g62820			
BraA03g62810			
BraA03g62800	AT5G15130	0.0E+00	100%
BraA03g62790	AT5G15140	3.0E-148	68%
BraA03g62780	AT5G15150	8.0E-126	94%
BraA03g62770	AT5G15160	2.0E-37	80%
BraA03g62760	AT5G15170	0.0E+00	100%
BraA03g62750	AT5G15190	2.0E-40	97%
BraA03g62740	AT5G15200	3.0E-106	94%
BraA03g62730	AT5G15220	8.0E-73	99%
BraA03g62720			
BraA03g62710	AT5G15230	2.0E-51	99%
BraA03g62700	AT5G15260	2.0E-72	87%
BraA03g62690	AT5G15265	2.0E-19	93%
BraA03g62680	AT5G15270	0.0E+00	96%
BraA03g62670	AT5G15290	2.0E-83	99%
BraA03g62660	AT5G15310	9.0E-69	82%
BraA03g62650	AT5G15320	7.0E-17	98%

BraA03g62640	AT5G15350	1.0E-84	99%
BraA03g62630	AT5G15430	5.0E-147	100%
BraA03g62620	AT5G15440	2.0E-165	98%
BraA03g62610	AT5G15460	2.0E-53	98%
BraA03g62600	AT5G15470	0.0E+00	100%
BraA03g62590	AT5G15480	5.0E-76	98%
BraA03g62580	AT5G15490	0.0E+00	100%
BraA03g62570	AT5G15500	4.0E-147	77%
BraA03g62560	AT5G15530	6.0E-109	100%
BraA03g62550	AT5G15550	0.0E+00	100%
BraA03g62540			
BraA03g62530	AT5G15610	0.0E+00	46%
BraA03g62520	AT5G15660	1.0E-09	70%
BraA03g62510	AT5G18160	6.0E-11	79%
BraA03g62500	AT5G15660	9.0E-13	34%
BraA03g62490	AT5G18160	6.0E-10	73%
BraA03g62480	AT5G18160	1.0E-09	73%
BraA03g62470	AT5G15630	0.0E+00	96%
BraA03g62460	AT5G15640	0.0E+00	100%
BraA03g62450	AT5G15650	0.0E+00	100%
BraA03g62440	AT5G15680	0.0E+00	100%
BraA03g62430	AT5G15710	0.0E+00	99%
BraA03g62420	AT5G15720	0.0E+00	100%
BraA03g62410	AT5G15740	0.0E+00	100%
BraA03g62400	AT5G15750	2.0E-96	99%
BraA03g62390	AT5G15790	6.0E-100	100%
BraA03g62380	AT5G15800	2.0E-87	99%
BraA03g62370	AT5G15810	0.0E+00	100%
BraA03g62360	AT5G15830	2.0E-68	99%
BraA03g62350			
BraA03g62340			
BraA03g62330			
BraA03g62320	AT5G14250	7.0E-79	71%
BraA03g62310			
BraA03g62300	AT5G08490	0.0E+00	99%
BraA03g62290	AT5G08490	1.0E-151	100%
BraA03g62280	AT5G14640	2.0E-172	57%
BraA03g62270			
BraA03g62260	AT5G14790	9.0E-19	64%
BraA03g62250	AT5G20300	7.0E-70	91%
BraA03g62240			
BraA03g62230	AT5G10340	4.0E-18	76%
BraA03g62220	AT5G15880	1.0E-165	100%
BraA03g62210	AT5G15920	0.0E+00	100%
BraA03g62200	AT5G15948	2.0E-17	70%
BraA03g62190	AT5G15950	0.0E+00	99%
BraA03g62180	AT5G16000	0.0E+00	65%
BraA03g62170	AT5G16010	2.0E-131	100%
BraA03g62160	AT5G16030	3.0E-142	100%
BraA03g62150	AT5G16070	0.0E+00	100%
BraA03g62140	AT5G16110	6.0E-68	99%
BraA03g62130	AT5G16130	3.0E-105	99%
BraA03g62120	AT5G16150	0.0E+00	100%
BraA03g62110	AT5G16170	2.0E-178	100%
BraA03g62100	AT5G16190	0.0E+00	85%
BraA03g62090	AT5G16220	1.0E-155	96%
BraA03g62080			

BraA03g62070	AT5G16270	0.0E+00	100%
BraA03g62060	AT5G16280	0.0E+00	100%
BraA03g62050	AT5G16300	0.0E+00	95%
BraA03g62040	AT5G16280	0.0E+00	96%
BraA03g62030	AT5G16300	0.0E+00	100%
BraA03g62020	AT5G16310	1.0E-173	99%
BraA03g62010	AT5G16330	3.0E-91	95%
BraA03g62000	AT5G16360	7.0E-82	81%
BraA03g61990			
BraA03g61980	AT5G16270	2.0E-132	98%
BraA03g61970	AT5G16280	1.0E-88	61%
BraA03g61960			
BraA03g61950			
BraA03g61940	AT5G16370	0.0E+00	100%
BraA03g61930	AT5G16730	1.0E-165	98%
BraA03g61920	AT5G16760	6.0E-167	99%
BraA03g61910	AT5G05780	9.0E-55	48%
BraA03g61900	AT5G16770	2.0E-94	100%
BraA03g61890	AT5G16800	3.0E-108	76%
BraA03g61880			
BraA03g61870	AT5G16830	9.0E-132	95%
BraA03g61860	AT5G16840	3.0E-124	99%
BraA03g61850	AT5G08740	3.0E-09	59%
BraA03g61840			
BraA03g61830	AT5G16870	1.0E-28	74%
BraA03g61820			
BraA03g61810			
BraA03g61800	AT5G16880	0.0E+00	99%
BraA03g61790	AT5G16890	0.0E+00	97%
BraA03g61780	AT5G16940	6.0E-70	99%
BraA03g61770	AT5G16950	4.0E-43	99%
BraA03g61760	AT5G16990	1.0E-174	78%
BraA03g61750	AT5G17020	0.0E+00	100%
BraA03g61740	AT5G17070	2.0E-21	89%
BraA03g61730			
BraA03g61720			
BraA03g61710	AT5G17090	2.0E-10	36%
BraA03g61700			
BraA03g61690	AT5G17120	3.0E-07	41%
BraA03g61680	AT5G17090	5.0E-44	69%
BraA03g61670	AT5G17120	9.0E-48	48%
BraA03g61660	AT5G17120	4.0E-49	72%
BraA03g61650	AT5G17150	4.0E-42	70%
BraA03g61640	AT5G17150	9.0E-08	41%
BraA03g61630	AT5G17160	2.0E-51	88%
BraA03g61620	AT5G17230	0.0E+00	100%
BraA03g61610	AT5G17260	0.0E+00	100%
BraA03g61600	AT5G17270	3.0E-168	95%
BraA03g61590	AT5G17270	0.0E+00	99%
BraA03g61580	AT5G17300	6.0E-149	97%
BraA03g61570	AT5G17310	0.0E+00	79%
BraA03g61560	AT5G17330	0.0E+00	97%
BraA03g61550	AT5G17330	0.0E+00	100%
BraA03g61540	AT5G17340	2.0E-09	84%
BraA03g61530	AT5G17340	3.0E-15	98%
BraA03g61520	AT5G17330	7.0E-44	99%
BraA03g61510	AT5G17340	2.0E-13	99%

BraA03g61500	AT5G17350	1.0E-68	85%
BraA03g61490	AT5G17340	8.0E-15	99%
BraA03g61480	AT5G17350	3.0E-68	85%
BraA03g61470	AT5G17330	8.0E-65	95%
BraA03g61460	AT5G17340	1.0E-08	66%
BraA03g61450	AT5G17340	8.0E-15	99%
BraA03g61440	AT5G17370	8.0E-36	93%
BraA03g61430	AT5G17410	0.0E+00	100%
BraA03g61420	AT5G17420	0.0E+00	95%
BraA03g61410	AT5G17440	7.0E-48	90%
BraA03g61400			
BraA03g61390	AT5G15660	7.0E-32	94%
BraA03g61380	AT5G18160	2.0E-33	77%
BraA03g61370			
BraA03g61360	AT5G17540	0.0E+00	98%
BraA03g61350	AT5G17560	1.0E-79	99%
BraA03g61340	AT5G17590	1.0E-38	95%
BraA03g61330	AT5G17600	9.0E-105	98%
BraA03g61320	AT5G17650	2.0E-15	91%
BraA03g61310	AT5G17690	2.0E-59	97%
BraA03g61300	AT5G17760	0.0E+00	100%
BraA03g61290	AT5G17770	1.0E-145	100%
BraA03g61280	AT5G17790	0.0E+00	100%
BraA03g61270	AT1G20480	5.0E-16	86%
BraA03g61260			
BraA03g61250			
BraA03g61240			
BraA03g61230	AT5G17910	0.0E+00	98%
BraA03g61220	AT5G17920	0.0E+00	100%
BraA03g61210	AT5G18070	0.0E+00	100%
BraA03g61200	AT5G18110	8.0E-115	100%
BraA03g61190	AT5G18120	1.0E-126	100%
BraA03g61180	AT5G18130	1.0E-97	100%
BraA03g61170	AT5G18150	1.0E-30	98%
BraA03g61160	AT5G18180	2.0E-49	54%
BraA03g61150			
BraA03g61140	AT5G18190	0.0E+00	100%
BraA03g61130	AT5G18230	0.0E+00	100%
BraA03g61120	AT5G18230	0.0E+00	100%
BraA03g61110	AT5G18250	4.0E-67	99%
BraA03g61100	AT5G20810	8.0E-07	52%
BraA03g61090			
BraA03g61080			
BraA03g61070			
BraA03g61060			
BraA03g61050	AT5G17800	1.0E-129	100%
BraA03g61040	AT5G17820	1.0E-163	99%
BraA03g61030	AT5G17840	8.0E-59	99%
BraA03g61020	AT5G17870	2.0E-49	97%
BraA03g61010	AT4G15096	2.0E-06	9%
BraA03g61000	AT5G17900	2.0E-55	79%
BraA03g60990	AT5G18270	8.0E-180	100%
BraA03g60980			
BraA03g60970	AT5G18280	0.0E+00	98%
BraA03g60960	AT5G18320	2.0E-102	97%
BraA03g60950	AT5G18320	2.0E-101	93%
BraA03g60940			

BraA03g60930	AT5G18320	3.0E-142	99%
BraA03g60920			
BraA03g60910	AT5G18380	7.0E-76	99%
BraA03g60900	AT5G18390	2.0E-164	100%
BraA03g60890	AT5G18390	1.0E-31	98%
BraA03g60880	AT5G18400	2.0E-118	99%
BraA03g60870	AT5G18410	0.0E+00	100%
BraA03g60860			
BraA03g60850	AT5G18420	1.0E-160	94%
BraA03g60840	AT5G18450	2.0E-119	98%
BraA03g60830	AT5G18470	1.0E-162	100%
BraA03g60820	AT5G18480	0.0E+00	99%
BraA03g60810	AT5G18500	0.0E+00	100%
BraA03g60800	AT5G18520	0.0E+00	100%
BraA03g60790	AT5G18550	7.0E-169	99%
BraA03g60780	AT5G18560	1.0E-141	100%
BraA03g60770			
BraA03g60760	AT5G18580	0.0E+00	98%
BraA03g60750	AT5G18600	8.0E-54	99%
BraA03g60740			
BraA03g60730			
BraA03g60720	AT5G18610	0.0E+00	99%
BraA03g60710	AT5G18650	1.0E-149	100%
BraA03g60700	AT5G18660	3.0E-41	60%
BraA03g60690	AT5G18670	0.0E+00	100%
BraA03g60680	AT5G18730	2.0E-154	71%
BraA03g60670			
BraA03g60660	AT5G18700	0.0E+00	95%
BraA03g60650	AT5G18700	0.0E+00	97%
BraA03g60640	AT5G18730	1.0E-115	81%
BraA03g60630	AT5G18730	8.0E-158	38%
BraA03g60620	AT5G18730	6.0E-148	88%
BraA03g60610	AT5G18730	1.0E-151	89%
BraA03g60600	AT5G18930	9.0E-180	100%
BraA03g60590	AT5G18920	3.0E-31	99%
BraA03g60580			
BraA03g60570			
BraA03g60560	AT5G18910	0.0E+00	95%
BraA03g60550	AT5G18850	1.0E-42	99%
BraA03g60540	AT5G18800	8.0E-60	99%
BraA03g60530	AT5G18790	1.0E-28	98%
BraA03g60520	AT5G18780	2.0E-55	98%
BraA03g60510	AT5G18780	3.0E-105	97%
BraA03g60500	AT5G18770	1.0E-140	99%
BraA03g60490			
BraA03g60480	AT5G13920	9.0E-04	29%
BraA03g60470	AT5G18770	5.0E-142	27%
BraA03g60460	AT5G18770	2.0E-125	85%
BraA03g60450	AT5G18770	4.0E-134	100%
BraA03g60440	AT5G18960	2.0E-62	100%
BraA03g60430	AT5G18950	9.0E-12	43%
BraA03g60420			
BraA03g60410	AT5G18770	3.0E-125	88%
BraA03g60400	AT5G18770	1.0E-141	29%
BraA03g60390	AT5G18780	1.0E-159	99%
BraA03g60380	AT5G18800	8.0E-60	99%
BraA03g60370	AT5G18850	3.0E-42	99%

BraA03g60360	AT5G18910	0.0E+00	95%
BraA03g60350	AT5G18920	3.0E-31	99%
BraA03g60340	AT5G18930	4.0E-179	100%
BraA03g60330			
BraA03g60320			
BraA03g60310	AT5G18950	9.0E-12	43%
BraA03g60300	AT5G18960	0.0E+00	100%
BraA03g60290	AT5G17970	1.0E-114	94%
BraA03g60280	AT5G18970	7.0E-87	99%
BraA03g60270	AT5G19000	0.0E+00	93%
BraA03g60260	AT5G19010	0.0E+00	100%
BraA03g60250	AT5G19030	2.0E-34	91%
BraA03g60240	AT5G19050	7.0E-180	90%
BraA03g60230	AT5G19070	1.0E-118	97%
BraA03g60220	AT5G10340	1.0E-70	92%
BraA03g60210			
BraA03g60200			
BraA03g60190	AT5G08450	1.0E-67	34%
BraA03g60180	AT5G19140	8.0E-84	56%
BraA03g60170	AT5G19150	0.0E+00	100%
BraA03g60160	AT5G19180	0.0E+00	96%
BraA03g60150	AT5G19190	8.0E-52	99%
BraA03g60140			
BraA03g60130			
BraA03g60120	AT5G19330	0.0E+00	100%
BraA03g60110	AT5G19340	4.0E-68	99%
BraA03g60100			
BraA03g60090	AT5G19350	0.0E+00	100%
BraA03g60080	AT5G19370	5.0E-131	100%
BraA03g60070	AT5G19420	0.0E+00	100%
BraA03g60060	AT5G19430	5.0E-117	100%
BraA03g60050	AT5G19473	8.0E-39	90%
BraA03g60040	AT5G12110	1.0E-92	100%
BraA03g60030	AT5G19520	0.0E+00	98%
BraA03g60020	AT5G19520	0.0E+00	79%
BraA03g60010			
BraA03g60000	AT5G19560	0.0E+00	100%
BraA03g59990	AT5G19600	0.0E+00	99%
BraA03g59980	AT5G19630	2.0E-116	98%
BraA03g59970	AT5G19660	0.0E+00	100%
BraA03g59960	AT5G19690	0.0E+00	100%
BraA03g59950	AT5G19750	2.0E-112	100%
BraA03g59940	AT5G19760	1.0E-168	100%
BraA03g59930	AT5G19780	0.0E+00	100%
BraA03g59920	AT5G19790	9.0E-101	98%
BraA03g59910	AT5G19820	0.0E+00	100%
BraA03g59900	AT5G19840	1.0E-158	98%
BraA03g59890	AT5G19840	1.0E-77	93%
BraA03g59880			
BraA03g59870	AT5G19855	8.0E-99	98%
BraA03g59860			
BraA03g59850	AT5G19875	1.0E-50	99%
BraA03g59840	AT5G19900	4.0E-138	100%
BraA03g59830	AT5G19940	5.0E-118	100%
BraA03g59820	AT5G19960	1.0E-19	98%
BraA03g59810			
BraA03g59800	AT4G29720	2.0E-30	80%

BraA03g59790	AT5G19960	4.0E-53	88%
BraA03g59780	AT5G19980	1.0E-31	94%
BraA03g59770			
BraA03g59760			
BraA03g59750	AT5G19980	7.0E-153	99%
BraA03g59740	AT5G19990	0.0E+00	100%
BraA03g59730	AT5G20010	3.0E-129	100%
BraA03g59720	AT5G20030	9.0E-120	100%
BraA03g59710			
BraA03g59700	AT5G20040	0.0E+00	99%
BraA03g59690	AT5G20050	0.0E+00	97%
BraA03g59680	AT5G20060	4.0E-135	100%
BraA03g59670	AT5G20070	0.0E+00	100%
BraA03g59660	AT5G20080	0.0E+00	100%
BraA03g59650	AT5G20090	3.0E-59	99%
BraA03g59640	AT5G20100	2.0E-39	86%
BraA03g59630	AT5G20110	1.0E-95	98%
BraA03g59620			
BraA03g59610	AT5G20130	2.0E-73	100%
BraA03g59600	AT5G20150	4.0E-119	100%
BraA03g59590	AT5G20160	1.0E-31	49%
BraA03g59580	AT5G20170	0.0E+00	100%
BraA03g59570	AT5G20180	3.0E-48	99%
BraA03g59560			
BraA03g59550	AT5G20190	6.0E-50	79%
BraA03g59540	AT5G15660	8.0E-08	65%
BraA03g59530	AT5G20240	1.0E-80	99%
BraA03g59520	AT5G20260	6.0E-167	99%
BraA03g59510			
BraA03g59500	AT5G20260	2.0E-19	54%
BraA03g59490	AT5G20270	2.0E-172	100%
BraA03g59480	AT5G20290	1.0E-94	94%
BraA03g59470	AT5G20310	1.0E-152	82%
BraA03g59460	AT5G20350	0.0E+00	99%
BraA03g59450	AT5G11250	1.0E-152	93%
BraA03g59440	AT5G20360	0.0E+00	97%
BraA03g59430	AT5G20370	3.0E-39	96%
BraA03g59420	AT5G20400	4.0E-170	99%
BraA03g59410	AT5G20480	0.0E+00	100%
BraA03g59400	AT5G20490	0.0E+00	100%
BraA03g59390	AT5G20510	2.0E-31	69%
BraA03g59380	AT5G20540	0.0E+00	100%
BraA03g59370			
BraA03g59360	AT5G20570	8.0E-60	99%
BraA03g59350	AT5G20580	0.0E+00	99%
BraA03g59340	AT5G20630	3.0E-112	100%
BraA03g59330	AT5G20650	4.0E-29	48%
BraA03g59320	AT5G20670	2.0E-70	94%
BraA03g59310	AT5G15460	2.0E-12	56%
BraA03g59300	AT5G20680	0.0E+00	100%
BraA03g59290	AT5G20720	1.0E-134	100%
BraA03g59280	AT5G20740	5.0E-95	99%
BraA03g59270			
BraA03g59260	AT5G20790	1.0E-61	98%
BraA03g59250	AT5G20810	7.0E-83	96%
BraA03g59240	AT5G20820	7.0E-61	99%
BraA03g59230	AT5G20830	0.0E+00	99%

BraA03g59220	AT5G20840	0.0E+00	99%
BraA03g59210	AT5G20850	0.0E+00	100%
BraA03g59200	AT5G20870	0.0E+00	99%
BraA03g59190			
BraA03g59180	AT5G20885	3.0E-77	98%
BraA03g59170	AT5G20890	0.0E+00	92%
BraA03g59160	AT5G20720	1.0E-54	53%
BraA03g59150	AT5G08420	5.0E-68	77%
BraA03g59140	AT5G20910	1.0E-154	96%
BraA03g59130	AT5G20935	4.0E-49	87%
BraA03g59120	AT5G20940	0.0E+00	97%
BraA03g59110	AT5G20940	4.0E-63	93%
BraA03g59100	AT5G20970	9.0E-99	100%
BraA03g59090	AT5G20980	0.0E+00	100%
BraA03g59080	AT5G20990	0.0E+00	100%
BraA03g59070			
BraA03g59060	AT5G20885	3.0E-77	98%
BraA03g59050	AT5G20890	0.0E+00	100%
BraA03g59040	AT5G20720	1.0E-54	60%
BraA03g59030	AT5G08420	3.0E-57	79%
BraA03g59020	AT5G22000	5.0E-159	100%
BraA03g59010	AT5G22791	2.0E-44	53%
BraA03g59000	AT5G22030	0.0E+00	99%
BraA03g58990	AT5G22040	9.0E-128	80%
BraA03g58980	AT5G22050	5.0E-151	99%
BraA03g58970	AT5G22060	0.0E+00	100%
BraA03g58960	AT5G22070	0.0E+00	100%
BraA03g58950	AT5G22080	2.0E-26	68%
BraA03g58940	AT5G22090	2.0E-171	100%
BraA03g58930	AT5G22140	4.0E-77	88%
BraA03g58920	AT5G22120	3.0E-154	100%
BraA03g58910	AT5G22120	2.0E-23	62%
BraA03g58900	AT5G22140	8.0E-107	96%
BraA03g58890	AT5G22160	7.0E-51	90%
BraA03g58880	AT5G22150	5.0E-32	71%
BraA03g58870	AT5G14960	3.0E-12	14%
BraA03g58860	AT5G22250	6.0E-132	99%
BraA03g58850	AT5G22270	7.0E-19	95%
BraA03g58840	AT5G22280	5.0E-52	99%
BraA03g58830	AT5G22290	1.0E-158	85%
BraA03g58820	AT5G22310	0.0E+00	99%
BraA03g58810			
BraA03g58800	AT5G22330	0.0E+00	100%
BraA03g58790	AT5G22340	8.0E-160	99%
BraA03g58780	AT5G22360	8.0E-124	100%
BraA03g58770			
BraA03g58760	AT5G22380	4.0E-106	94%
BraA03g58750	AT5G22500	2.0E-177	100%
BraA03g58740			
BraA03g58730	AT5G22440	9.0E-113	100%
BraA03g58720	AT5G22450	0.0E+00	100%
BraA03g58710	AT5G22460	5.0E-168	99%
BraA03g58700	AT5G22480	0.0E+00	100%
BraA03g58690			
BraA03g58680	AT5G22650	5.0E-60	95%
BraA03g58670	AT5G22670	4.0E-68	97%
BraA03g58660	AT5G22670	3.0E-45	51%

BraA03g58650	AT5G22670	1.0E-131	96%	
BraA03g58640	AT5G22670	5.0E-77	96%	
BraA03g58630	AT5G22720	6.0E-24	92%	
BraA03g58620	AT5G22730	1.0E-159	94%	
BraA03g58610				
BraA03g58600	AT5G22740	0.0E+00	100%	
BraA03g58590	AT5G22750	0.0E+00	100%	
BraA03g58580	AT5G22760	0.0E+00	100%	
BraA03g58570	AT5G06010	5.0E-10	35%	
BraA03g58560	AT5G22790	7.0E-179	100%	
BraA03g58550	AT5G15660	5.0E-19	79%	
BraA03g58540	AT5G22791	1.0E-79	53%	
BraA03g58530	AT5G22880	3.0E-57	99%	
BraA03g58520	AT5G22910	0.0E+00	100%	
BraA03g58510	AT5G22920	7.0E-56	100%	
BraA03g58500				
BraA03g58490	AT5G22970	2.0E-07	75%	
BraA03g58480	AT5G22940	0.0E+00	90%	
BraA03g58470	AT5G22950	3.0E-116	100%	
BraA03g58460	AT5G22970	7.0E-24	90%	
BraA03g58450				CD1
BraA03g58440				
BraA03g58430				
BraA03g58420	AT5G60790	0.0E+00	94%	W
BraA03g58410	AT5G52740	1.0E-04	22%	
BraA03g58400	AT5G53890	3.0E-11	97%	
BraA03g58390	AT5G60660	1.0E-153	99%	
BraA03g58380	AT5G60650	8.0E-43	37%	
BraA03g58370	AT5G60630	2.0E-25	99%	
BraA03g58360	AT5G60615	7.0E-33	99%	
BraA03g58350				
BraA03g58340				
BraA03g58330	AT5G60530	2.0E-176	100%	
BraA03g58320	AT5G60490	4.0E-91	100%	
BraA03g58310	AT5G60480	3.0E-70	97%	
BraA03g58300				
BraA03g58290	AT5G60460	8.0E-37	99%	
BraA03g58280				
BraA03g58270	AT5G60400	1.0E-10	71%	
BraA03g58260	AT5G60390	0.0E+00	100%	
BraA03g58250				
BraA03g58240				
BraA03g58230				
BraA03g58220	AT5G60360	1.0E-56	86%	
BraA03g58210	AT5G57490	1.0E-63	100%	
BraA03g58200	AT5G60340	5.0E-50	65%	
BraA03g58190	AT5G60340	6.0E-18	53%	
BraA03g58180	AT5G60360	1.0E-19	83%	
BraA03g58170	AT5G60360	2.0E-113	95%	
BraA03g58160				
BraA03g58150	AT5G60340	1.0E-78	91%	
BraA03g58140	AT5G60320	0.0E+00	100%	
BraA03g58130	AT5G60300	0.0E+00	100%	
BraA03g58120	AT5G60270	0.0E+00	99%	
BraA03g58110	AT5G60250	0.0E+00	99%	
BraA03g58100	AT5G60230	1.0E-126	99%	
BraA03g58090	AT5G60220	7.0E-127	97%	

BraA03g58080	AT5G60210	0.0E+00	100%
BraA03g58070	AT5G60200	9.0E-119	100%
BraA03g58060	AT5G60190	4.0E-111	98%
BraA03g58050			
BraA03g58040	AT5G60110	1.0E-97	96%
BraA03g58030	AT5G60170	0.0E+00	100%
BraA03g58020	AT5G60180	7.0E-106	97%
BraA03g58010	AT5G60170	0.0E+00	100%
BraA03g58000	AT5G60070	0.0E+00	100%
BraA03g57990	AT5G60060	8.0E-119	86%
BraA03g57980	AT5G60030	5.0E-24	96%
BraA03g57970	AT5G60020	0.0E+00	100%
BraA03g57960	AT5G59960	1.0E-112	94%
BraA03g57950	AT5G59910	2.0E-59	96%
BraA03g57940	AT5G59890	4.0E-78	99%
BraA03g57930	AT5G59880	1.0E-71	87%
BraA03g57920	AT5G59870	4.0E-73	99%
BraA03g57910	AT5G54580	4.0E-13	87%
BraA03g57900	AT5G59850	3.0E-73	99%
BraA03g57890	AT5G59845	3.0E-36	99%
BraA03g57880	AT5G59840	2.0E-117	95%
BraA03g57870	AT5G59820	2.0E-74	99%
BraA03g57860	AT5G59820	9.0E-74	99%
BraA03g57850	AT5G59800	6.0E-71	99%
BraA03g57840			
BraA03g57830	AT5G59780	2.0E-81	69%
BraA03g57820	AT5G59740	0.0E+00	99%
BraA03g57810	AT5G59730	0.0E+00	100%
BraA03g57800	AT5G59720	2.0E-77	99%
BraA03g57790	AT5G59710	0.0E+00	100%
BraA03g57780	AT5G59970	8.0E-54	99%
BraA03g57770	AT5G59670	0.0E+00	98%
BraA03g57760	AT5G59680	0.0E+00	98%
BraA03g57750	AT5G59613	8.0E-29	36%
BraA03g57740			
BraA03g57730	AT5G60180	3.0E-68	93%
BraA03g57720	AT5G60180	8.0E-61	82%
BraA03g57710	AT5G59600	8.0E-47	77%
BraA03g57700	AT5G59590	0.0E+00	98%
BraA03g57690			
BraA03g57680	AT5G59550	2.0E-144	94%
BraA03g57670	AT5G59540	1.0E-79	98%
BraA03g57660	AT5G59540	4.0E-76	91%
BraA03g57650			
BraA03g57640	AT5G59290	7.0E-36	99%
BraA03g57630	AT5G59290	6.0E-40	74%
BraA03g57620	AT5G59320	7.0E-53	99%
BraA03g57610	AT5G59350	1.0E-91	97%
BraA03g57600	AT5G59370	0.0E+00	100%
BraA03g57590	AT5G59380	4.0E-66	99%
BraA03g57580	AT5G56040	2.0E-99	96%
BraA03g57570	AT5G59480	2.0E-129	100%
BraA03g57560	AT5G60560	2.0E-23	96%
BraA03g57550			
BraA03g57540			
BraA03g57530	AT5G59540	2.0E-82	91%
BraA03g57520			

BraA03g57510	AT5G59540	6.0E-87	99%
BraA03g57500	AT5G59540	2.0E-82	92%
BraA03g57490	AT5G59540	3.0E-79	83%
BraA03g57480	AT5G59540	1.0E-79	95%
BraA03g57470	AT5G59550	1.0E-144	94%
BraA03g57460	AT5G59590	0.0E+00	98%
BraA03g57450	AT5G59600	1.0E-24	63%
BraA03g57440	AT5G60180	2.0E-61	82%
BraA03g57430	AT5G60180	1.0E-67	93%
BraA03g57420	AT5G60180	1.0E-61	84%
BraA03g57410	AT5G59613	4.0E-28	98%
BraA03g57400	AT5G59680	2.0E-180	97%
BraA03g57390	AT5G59670	8.0E-146	100%
BraA03g57380	AT5G59670	0.0E+00	98%
BraA03g57370	AT5G59970	8.0E-54	99%
BraA03g57360	AT5G59710	0.0E+00	100%
BraA03g57350	AT5G59720	1.0E-76	99%
BraA03g57340	AT5G59730	0.0E+00	100%
BraA03g57330	AT5G59740	0.0E+00	99%
BraA03g57320	AT5G59780	1.0E-79	69%
BraA03g57310			
BraA03g57300	AT5G59800	2.0E-68	90%
BraA03g57290	AT5G59820	3.0E-75	99%
BraA03g57280	AT5G59840	1.0E-116	95%
BraA03g57270	AT5G59845	3.0E-36	97%
BraA03g57260	AT5G59850	3.0E-73	99%
BraA03g57250	AT5G54580	4.0E-13	87%
BraA03g57240	AT5G59870	4.0E-73	99%
BraA03g57230	AT5G59880	4.0E-72	99%
BraA03g57220	AT5G59890	4.0E-78	99%
BraA03g57210	AT5G59910	3.0E-14	92%
BraA03g57200	AT5G58930	3.0E-97	100%
BraA03g57190	AT5G58920	2.0E-90	99%
BraA03g57180	AT5G58900	5.0E-138	100%
BraA03g57170	AT5G58880	0.0E+00	100%
BraA03g57160	AT5G58860	0.0E+00	100%
BraA03g57150	AT5G58850	0.0E+00	100%
BraA03g57140	AT5G58787	8.0E-118	98%
BraA03g57130	AT5G58730	8.0E-49	98%
BraA03g57120	AT5G58720	0.0E+00	96%
BraA03g57110	AT5G58710	7.0E-98	90%
BraA03g57100	AT5G58670	0.0E+00	99%
BraA03g57090	AT5G58650	2.0E-10	59%
BraA03g57080	AT5G58640	8.0E-102	99%
BraA03g57070			
BraA03g57060			
BraA03g57050	AT5G58580	8.0E-78	98%
BraA03g57040	AT5G58575	2.0E-77	94%
BraA03g57030	AT5G58560	5.0E-76	99%
BraA03g57020	AT5G58560	2.0E-130	97%
BraA03g57010	AT5G54570	6.0E-86	81%
BraA03g57000	AT5G54570	7.0E-87	91%
BraA03g56990			
BraA03g56980			
BraA03g56970			
BraA03g56960	AT5G58530	4.0E-111	100%
BraA03g56950			

BraA03g56940	AT5G58520	0.0E+00	100%
BraA03g56930	AT5G58510	0.0E+00	100%
BraA03g56920	AT5G58470	3.0E-87	75%
BraA03g56910			
BraA03g56900	AT5G58460	0.0E+00	100%
BraA03g56890	AT5G53840	3.0E-36	84%
BraA03g56880			
BraA03g56870			
BraA03g56860	AT5G58430	0.0E+00	100%
BraA03g56850	AT5G58420	8.0E-151	100%
BraA03g56840	AT5G58410	0.0E+00	100%
BraA03g56830	AT5G58400	1.0E-142	99%
BraA03g56820	AT5G58375	2.0E-16	90%
BraA03g56810	AT5G58350	0.0E+00	100%
BraA03g56800			
BraA03g56790	AT5G58330	0.0E+00	100%
BraA03g56780	AT5G58320	0.0E+00	100%
BraA03g56770			
BraA03g56760	AT5G58300	0.0E+00	99%
BraA03g56750	AT5G58290	0.0E+00	99%
BraA03g56740	AT5G58270	0.0E+00	100%
BraA03g56730	AT5G58260	3.0E-104	100%
BraA03g56720	AT5G58250	1.0E-84	99%
BraA03g56710	AT5G58230	0.0E+00	100%
BraA03g56700	AT5G58210	1.0E-58	99%
BraA03g56690	AT5G58190	0.0E+00	97%
BraA03g56680	AT5G58160	1.0E-06	44%
BraA03g56670	AT5G58150	0.0E+00	100%
BraA03g56660	AT5G58140	0.0E+00	100%
BraA03g56650	AT5G58110	3.0E-95	99%
BraA03g56640	AT5G58070	1.0E-96	98%
BraA03g56630	AT5G58170	0.0E+00	95%
BraA03g56620	AT5G58040	0.0E+00	97%
BraA03g56610	AT5G58030	2.0E-113	99%
BraA03g56600	AT5G58010	2.0E-86	85%
BraA03g56590	AT5G58005	6.0E-61	99%
BraA03g56580	AT5G58003	0.0E+00	95%
BraA03g56570	AT5G57990	0.0E+00	99%
BraA03g56560	AT5G57980	5.0E-102	100%
BraA03g56550	AT5G57920	1.0E-59	88%
BraA03g56540	AT5G57910	2.0E-58	100%
BraA03g56530	AT5G57860	2.0E-45	99%
BraA03g56520	AT5G57850	2.0E-171	95%
BraA03g56510	AT5G57840	0.0E+00	81%
BraA03g56500	AT5G57840	0.0E+00	100%
BraA03g56490	AT5G57830	5.0E-85	96%
BraA03g56480	AT5G57830	1.0E-27	99%
BraA03g56470	AT5G57800	2.0E-139	31%
BraA03g56460	AT5G57830	2.0E-35	65%
BraA03g56450	AT5G57800	0.0E+00	99%
BraA03g56440	AT5G57800	0.0E+00	100%
BraA03g56430	AT5G57790	1.0E-43	99%
BraA03g56420	AT5G57760	7.0E-24	74%
BraA03g56410	AT5G57700	5.0E-178	88%
BraA03g56400	AT5G57690	0.0E+00	100%
BraA03g56390	AT5G59060	2.0E-05	26%
BraA03g56380	AT5G57685	7.0E-67	99%

BraA03g56370	AT5G57630	3.0E-103	57%
BraA03g56360	AT5G57625	1.0E-99	100%
BraA03g56350	AT5G57620	3.0E-90	100%
BraA03g56340	AT5G57610	0.0E+00	98%
BraA03g56330	AT5G57590	0.0E+00	97%
BraA03g56320	AT5G57530	5.0E-153	99%
BraA03g56310	AT5G57520	2.0E-71	99%
BraA03g56300	AT5G57500	9.0E-172	100%
BraA03g56290			
BraA03g56280	AT5G57490	1.0E-141	100%
BraA03g56270	AT5G57480	0.0E+00	100%
BraA03g56260	AT5G57460	0.0E+00	100%
BraA03g56250	AT5G57450	3.0E-139	100%
BraA03g56240	AT5G57410	0.0E+00	98%
BraA03g56230			
BraA03g56220			
BraA03g56210	AT5G57380	0.0E+00	100%
BraA03g56200	AT5G57330	2.0E-173	100%
BraA03g56190	AT5G57320	0.0E+00	98%
BraA03g56180	AT5G57300	6.0E-156	100%
BraA03g56170	AT5G57280	3.0E-141	100%
BraA03g56160	AT5G57270	1.0E-170	90%
BraA03g56150	AT5G57260	0.0E+00	99%
BraA03g56140	AT5G57220	0.0E+00	100%
BraA03g56130	AT5G57150	2.0E-114	96%
BraA03g56120	AT5G57123	7.0E-28	56%
BraA03g56110			
BraA03g56100	AT5G57100	4.0E-149	95%
BraA03g56090			
BraA03g56080	AT5G57090	0.0E+00	100%
BraA03g56070	AT5G57040	4.0E-99	99%
BraA03g56060	AT5G57035	0.0E+00	100%
BraA03g56050	AT5G56560	2.0E-88	100%
BraA03g56040			
BraA03g56030	AT5G57030	0.0E+00	100%
BraA03g56020	AT5G57015	4.0E-144	100%
BraA03g56010	AT5G57000	9.0E-62	94%
BraA03g56000	AT5G56940	1.0E-21	40%
BraA03g55990	AT5G60360	2.0E-13	68%
BraA03g55980	AT5G56920	4.0E-68	100%
BraA03g55970	AT5G56920	4.0E-52	76%
BraA03g55960	AT5G56920	3.0E-05	56%
BraA03g55950	AT5G56920	3.0E-07	63%
BraA03g55940	AT5G56920	2.0E-05	56%
BraA03g55930			
BraA03g55920	AT5G56880	1.0E-55	99%
BraA03g55910	AT5G56870	0.0E+00	100%
BraA03g55900	AT5G56860	4.0E-131	100%
BraA03g55890	AT5G56500	0.0E+00	100%
BraA03g55880	AT5G56420	1.0E-77	97%
BraA03g55870	AT5G56000	0.0E+00	100%
BraA03g55860	AT5G56440	2.0E-89	100%
BraA03g55850	AT5G56440	5.0E-136	100%
BraA03g55840	AT5G56420	3.0E-99	87%
BraA03g55830	AT5G56390	2.0E-22	90%
BraA03g55820	AT5G56420	3.0E-91	81%
BraA03g55810	AT5G56380	1.0E-110	94%

BraA03g55800	AT5G56370	8.0E-115	98%
BraA03g55790			
BraA03g55780	AT5G56360	0.0E+00	96%
BraA03g55770	AT5G56350	0.0E+00	100%
BraA03g55760	AT5G56340	2.0E-143	90%
BraA03g55750			
BraA03g55740	AT5G56390	3.0E-58	99%
BraA03g55730	AT5G56310	0.0E+00	99%
BraA03g55720	AT5G56300	0.0E+00	99%
BraA03g55710	AT5G56290	0.0E+00	100%
BraA03g55700	AT5G56270	7.0E-157	100%
BraA03g55690	AT5G56260	3.0E-92	99%
BraA03g55680			
BraA03g55670	AT5G56250	0.0E+00	95%
BraA03g55660	AT5G56240	0.0E+00	64%
BraA03g55650	AT5G56200	1.0E-102	96%
BraA03g55640	AT5G56170	6.0E-63	82%
BraA03g55630	AT5G56160	4.0E-161	73%
BraA03g55620	AT5G60740	0.0E+00	100%
BraA03g55610	AT5G56150	2.0E-82	99%
BraA03g55600	AT5G56140	5.0E-149	93%
BraA03g55590	AT5G56000	0.0E+00	100%
BraA03g55580	AT5G55990	2.0E-94	81%
BraA03g55570	AT5G55970	2.0E-75	98%
BraA03g55560			
BraA03g55550	AT5G56000	0.0E+00	100%
BraA03g55540	AT5G55940	2.0E-77	99%
BraA03g55530	AT5G55910	0.0E+00	100%
BraA03g55520	AT5G55860	0.0E+00	100%
BraA03g55510	AT5G55850	4.0E-32	93%
BraA03g55500			
BraA03g55490	AT5G55820	0.0E+00	59%
BraA03g55480	AT5G55810	6.0E-119	100%
BraA03g55470			
BraA03g55460	AT5G55760	0.0E+00	94%
BraA03g55450			
BraA03g55440	AT5G55730	2.0E-67	87%
BraA03g55430	AT5G55700	0.0E+00	68%
BraA03g55420	AT5G55690	2.0E-111	100%
BraA03g55410	AT5G55670	0.0E+00	76%
BraA03g55400	AT5G55630	1.0E-176	100%
BraA03g55390	AT5G55620	4.0E-41	99%
BraA03g55380			
BraA03g55370	AT5G55600	0.0E+00	98%
BraA03g55360	AT5G55580	0.0E+00	100%
BraA03g55350	AT5G56270	3.0E-39	49%
BraA03g55340	AT5G55560	1.0E-150	92%
BraA03g55330			
BraA03g55320			
BraA03g55310	AT5G55550	0.0E+00	99%
BraA03g55300	AT5G55530	0.0E+00	100%
BraA03g55290	AT5G55510	1.0E-92	98%
BraA03g55280	AT5G55500	0.0E+00	100%
BraA03g55270	AT5G55490	5.0E-139	68%
BraA03g55260			
BraA03g55250			
BraA03g55240	AT5G55480	0.0E+00	100%

BraA03g55230	AT5G55450	1.0E-26	98%
BraA03g55220	AT5G55410	2.0E-43	94%
BraA03g55210	AT5G55400	0.0E+00	100%
BraA03g55200			
BraA03g55190	AT5G55370	4.0E-75	51%
BraA03g55180	AT5G55350	6.0E-149	97%
BraA03g55170	AT5G55340	4.0E-148	94%
BraA03g55160	AT5G55360	2.0E-113	100%
BraA03g55150	AT5G55350	2.0E-116	99%
BraA03g55140	AT5G55290	5.0E-30	99%
BraA03g55130	AT5G55230	0.0E+00	100%
BraA03g55120	AT5G55210	1.0E-59	99%
BraA03g55110	AT5G55200	1.0E-25	78%
BraA03g55100	AT5G55180	0.0E+00	100%
BraA03g55090	AT5G55150	3.0E-130	98%
BraA03g55080	AT5G55110	5.0E-38	62%
BraA03g55070	AT5G55110	9.0E-46	73%
BraA03g55060	AT5G55090	0.0E+00	96%
BraA03g55050	AT5G55070	0.0E+00	100%
BraA03g55040	AT5G55060	0.0E+00	99%
BraA03g55030	AT5G55050	1.0E-149	94%
BraA03g55020	AT5G55050	2.0E-166	100%
BraA03g55010	AT5G55050	3.0E-179	100%
BraA03g55000	AT5G55040	6.0E-12	66%
BraA03g54990	AT5G55020	8.0E-126	82%
BraA03g54980	AT5G55000	1.0E-151	94%
BraA03g54970	AT5G54980	2.0E-82	99%
BraA03g54960	AT5G54970	1.0E-36	99%
BraA03g54950	AT5G54940	2.0E-56	99%
BraA03g54940	AT5G54930	5.0E-84	99%
BraA03g54930			
BraA03g54920	AT5G54820	5.0E-115	98%
BraA03g54910			
BraA03g54900	AT5G51570	6.0E-07	33%
BraA03g54890	AT5G54800	0.0E+00	100%
BraA03g54880	AT5G54780	0.0E+00	97%
BraA03g54870	AT5G54770	0.0E+00	100%
BraA03g54860			
BraA03g54850	AT5G54730	0.0E+00	100%
BraA03g54840	AT5G54710	2.0E-80	89%
BraA03g54830	AT5G54690	1.0E-68	85%
BraA03g54820	AT5G54690	1.0E-80	99%
BraA03g54810	AT5G54690	1.0E-29	69%
BraA03g54800	AT5G54680	1.0E-120	100%
BraA03g54790	AT5G54670	4.0E-15	72%
BraA03g54780	AT5G54650	0.0E+00	100%
BraA03g54770			
BraA03g54760	AT5G54620	4.0E-91	93%
BraA03g54750	AT5G54620	4.0E-78	99%
BraA03g54740	AT5G54600	8.0E-65	99%
BraA03g54730	AT5G54590	6.0E-124	53%
BraA03g54720	AT5G54585	1.0E-44	99%
BraA03g54710	AT5G54580	5.0E-76	99%
BraA03g54700	AT5G54520	0.0E+00	100%
BraA03g54690			
BraA03g54680	AT5G54510	0.0E+00	100%
BraA03g54670	AT5G54500	1.0E-112	99%

BraA03g54660	AT5G54430	6.0E-82	97%
BraA03g54650	AT5G54380	2.0E-32	82%
BraA03g54640	AT5G54380	3.0E-50	98%
BraA03g54630	AT5G53670	8.0E-04	85%
BraA03g54620	AT5G54430	5.0E-11	90%
BraA03g54610			
BraA03g54600	AT5G54380	0.0E+00	99%
BraA03g54590	AT5G54370	0.0E+00	100%
BraA03g54580	AT5G54340	5.0E-10	19%
BraA03g54570	AT5G54310	0.0E+00	100%
BraA03g54560	AT5G54300	8.0E-113	99%
BraA03g54550			
BraA03g54540	AT5G54230	3.0E-48	99%
BraA03g54530	AT5G54220	9.0E-27	97%
BraA03g54520	AT5G54100	8.0E-23	46%
BraA03g54510	AT5G54100	4.0E-24	75%
BraA03g54500	AT5G54095	5.0E-18	99%
BraA03g54490	AT5G54010	0.0E+00	100%
BraA03g54480	AT5G54010	0.0E+00	100%
BraA03g54470	AT5G54010	2.0E-64	100%
BraA03g54460	AT5G57220	1.0E-36	56%
BraA03g54450			
BraA03g54440	AT5G55320	7.0E-28	87%
BraA03g54430	AT5G53620	1.0E-68	62%
BraA03g54420	AT5G53520	4.0E-99	87%
BraA03g54410	AT5G56460	1.0E-09	75%
BraA03g54400			
BraA03g54390	AT5G53770	5.0E-05	32%
BraA03g54380	AT5G54600	6.0E-12	29%
BraA03g54370	AT5G53870	3.0E-72	88%
BraA03g54360	AT5G53750	2.0E-176	100%
BraA03g54350			
BraA03g54340	AT5G54062	1.0E-75	100%
BraA03g54330	AT5G53650	2.0E-34	96%
BraA03g54320	AT5G53620	0.0E+00	99%
BraA03g54310	AT5G53840	1.0E-76	100%
BraA03g54300	AT5G53620	7.0E-10	48%
BraA03g54290	AT5G53840	5.0E-84	98%
BraA03g54280	AT5G53560	6.0E-18	37%
BraA03g54270	AT5G53560	8.0E-58	99%
BraA03g54260	AT5G53540	8.0E-174	100%
BraA03g54250	AT5G53520	0.0E+00	100%
BraA03g54240	AT5G53510	0.0E+00	97%
BraA03g54230	AT5G53500	0.0E+00	100%
BraA03g54220	AT5G53460	0.0E+00	100%
BraA03g54210	AT5G53440	0.0E+00	64%
BraA03g54200	AT5G53420	2.0E-126	99%
BraA03g54190	AT5G53400	6.0E-123	100%
BraA03g54180			
BraA03g54170	AT5G53390	0.0E+00	100%
BraA03g54160	AT5G53350	0.0E+00	100%
BraA03g54150	AT5G53330	2.0E-85	100%
BraA03g54140	AT5G53320	0.0E+00	79%
BraA03g54130	AT5G53290	2.0E-42	46%
BraA03g54120	AT5G58230	6.0E-22	20%
BraA03g54110	AT5G02050	2.0E-48	96%
BraA03g54100	AT5G53280	2.0E-101	83%

BraA03g54090	AT5G53260	2.0E-72	99%
BraA03g54080	AT5G53250	2.0E-27	98%
BraA03g54070	AT5G53220	3.0E-94	74%
BraA03g54060	AT5G53210	1.0E-135	99%
BraA03g54050	AT5G53200	3.0E-23	82%
BraA03g54040	AT5G53180	0.0E+00	100%
BraA03g54030	AT5G53170	3.0E-16	25%
BraA03g54020	AT5G53160	1.0E-58	62%
BraA03g54010			
BraA03g54000	AT5G02050	6.0E-23	93%
BraA03g53990			
BraA03g53980	AT5G53290	7.0E-110	98%
BraA03g53970	AT5G52840	2.0E-31	46%
BraA03g53960	AT5G52870	1.0E-94	99%
BraA03g53950	AT5G52882	0.0E+00	100%
BraA03g53940			
BraA03g53930			
BraA03g53920	AT5G52890	1.0E-34	94%
BraA03g53910	AT5G52900	4.0E-98	95%
BraA03g53900	AT5G53045	9.0E-45	98%
BraA03g53890	AT5G53050	6.0E-145	76%
BraA03g53880	AT5G53045	4.0E-45	98%
BraA03g53870	AT5G53640	8.0E-38	96%
BraA03g53860	AT5G51250	2.0E-14	90%
BraA03g53850	AT5G53100	2.0E-164	94%
BraA03g53840	AT5G53140	7.0E-153	100%
BraA03g53830	AT5G52670	7.0E-34	18%
BraA03g53820	AT5G52760	3.0E-31	99%
BraA03g53810	AT5G52840	9.0E-55	97%
BraA03g53800	AT5G52660	2.0E-22	88%
BraA03g53790	AT5G53210	1.0E-135	99%
BraA03g53780	AT5G53200	3.0E-23	82%
BraA03g53770	AT5G53180	0.0E+00	100%
BraA03g53760	AT5G53170	3.0E-16	25%
BraA03g53750	AT5G53160	1.0E-58	62%
BraA03g53740			
BraA03g53730	AT5G53140	2.0E-138	94%
BraA03g53720	AT5G53100	2.0E-164	94%
BraA03g53710	AT5G51250	2.0E-14	90%
BraA03g53700	AT5G53640	2.0E-77	90%
BraA03g53690	AT5G53045	4.0E-45	98%
BraA03g53680	AT5G53050	1.0E-45	47%
BraA03g53670	AT5G53045	9.0E-45	98%
BraA03g53660	AT5G52900	4.0E-98	95%
BraA03g53650	AT5G52890	1.0E-34	94%
BraA03g53640			
BraA03g53630			
BraA03g53620	AT5G52882	0.0E+00	100%
BraA03g53610	AT5G52870	5.0E-81	99%
BraA03g53600	AT5G52840	1.0E-90	99%
BraA03g53590	AT5G52760	3.0E-31	99%
BraA03g53580			
BraA03g53570	AT5G52670	4.0E-36	99%
BraA03g53560	AT5G52660	1.0E-144	100%
BraA03g53550	AT5G52650	5.0E-63	73%
BraA03g53540	AT5G52600	6.0E-47	99%
BraA03g53530	AT5G59105	1.0E-08	89%

BraA03g53520			
BraA03g53510	AT5G52540	0.0E+00	100%
BraA03g53500	AT5G59105	1.0E-08	89%
BraA03g53490			
BraA03g53480	AT5G52610	3.0E-14	72%
BraA03g53470	AT5G52290	0.0E+00	100%
BraA03g53460	AT5G52270	1.0E-80	73%
BraA03g53450	AT5G52260	4.0E-86	98%
BraA03g53440			
BraA03g53430	AT5G52250	4.0E-155	97%
BraA03g53420	AT5G52210	7.0E-114	94%
BraA03g53410	AT5G52190	4.0E-68	99%
BraA03g53400	AT5G52160	7.0E-31	67%
BraA03g53390	AT5G52120	2.0E-51	96%
BraA03g53380	AT5G52110	5.0E-27	53%
BraA03g53370	AT5G52210	1.0E-107	99%
BraA03g53360	AT5G52250	4.0E-155	97%
BraA03g53350			
BraA03g53340	AT5G52260	4.0E-86	98%
BraA03g53330	AT5G52270	2.0E-13	37%
BraA03g53320	AT5G52290	0.0E+00	100%
BraA03g53310	AT5G52610	3.0E-14	72%
BraA03g53300			
BraA03g53290	AT5G51280	5.0E-48	91%
BraA03g53280	AT5G53080	1.0E-17	47%
BraA03g53270	AT5G52120	1.0E-62	45%
BraA03g53260	AT5G52110	2.0E-142	100%
BraA03g53250	AT5G52100	5.0E-169	100%
BraA03g53240			
BraA03g53230			
BraA03g53220	AT5G52070	8.0E-57	86%
BraA03g53210	AT5G52060	9.0E-141	100%
BraA03g53200			
BraA03g53190	AT5G56810	2.0E-103	88%
BraA03g53180	AT5G56810	9.0E-86	42%
BraA03g53170	AT5G52050	0.0E+00	100%
BraA03g53160	AT5G52040	9.0E-59	100%
BraA03g53150	AT5G52040	5.0E-16	45%
BraA03g53140	AT5G52020	5.0E-23	96%
BraA03g53130	AT5G52010	0.0E+00	96%
BraA03g53120	AT5G51980	0.0E+00	99%
BraA03g53110	AT5G51970	1.0E-32	65%
BraA03g53100	AT5G51950	0.0E+00	100%
BraA03g53090	AT5G51950	0.0E+00	98%
BraA03g53080	AT5G51950	0.0E+00	100%
BraA03g53070			
BraA03g53060	AT5G51860	2.0E-69	100%
BraA03g53050	AT5G51860	4.0E-75	95%
BraA03g53040			
BraA03g53030			
BraA03g53020			
BraA03g53010	AT5G51840	4.0E-120	100%
BraA03g53000	AT5G51830	0.0E+00	100%
BraA03g52990	AT5G51770	0.0E+00	100%
BraA03g52980	AT5G51760	0.0E+00	99%
BraA03g52970			
BraA03g52960			

BraA03g52950	AT5G51600	1.0E-98	83%	
BraA03g52940	AT5G51590	1.0E-134	100%	
BraA03g52930				
BraA03g52920	AT5G51570	2.0E-163	99%	
BraA03g52910				
BraA03g52900	AT5G51540	0.0E+00	82%	
BraA03g52890	AT5G51490	0.0E+00	97%	
BraA03g52880	AT5G51480	0.0E+00	97%	
BraA03g52870	AT5G59320	7.0E-47	99%	
BraA03g52860	AT5G51470	0.0E+00	100%	
BraA03g52850				
BraA03g52840	AT5G51451	3.0E-27	99%	
BraA03g52830	AT5G51440	3.0E-78	99%	
BraA03g52820	AT5G51410	1.0E-158	99%	
BraA03g52810	AT5G51380	0.0E+00	100%	
BraA03g52800	AT5G51300	0.0E+00	100%	
BraA03g52790	AT5G51290	0.0E+00	95%	
BraA03g52780	AT5G51230	5.0E-49	75%	
BraA03g52770	AT5G51230	1.0E-82	78%	
BraA03g52760	AT5G51230	5.0E-122	79%	
BraA03g52750				
BraA03g52740				
BraA03g52730	AT5G51451	3.0E-27	99%	
BraA03g52720	AT5G51440	1.0E-75	99%	
BraA03g52710	AT5G51410	2.0E-147	82%	
BraA03g52700	AT5G51380	0.0E+00	100%	
BraA03g52690	AT5G51300	0.0E+00	100%	
BraA03g52680	AT5G51290	0.0E+00	98%	
BraA03g52670	AT5G51230	1.0E-141	75%	
BraA03g52660	AT5G51230	1.0E-143	80%	
BraA03g52650				
BraA03g52640				
BraA03g52630	AT5G51160	0.0E+00	99%	
BraA03g52620	AT5G51150	0.0E+00	90%	
BraA03g52610	AT5G51140	0.0E+00	100%	
BraA03g52600	AT5G51130	2.0E-129	100%	
BraA03g52590				
BraA03g52580	AT5G51120	2.0E-100	100%	
BraA03g52570	AT5G51110	4.0E-102	100%	
BraA03g52560	AT5G51105	6.0E-35	92%	
BraA03g52550	AT5G51105	5.0E-40	98%	
BraA03g52540	AT5G51100	1.0E-150	93%	
BraA03g52530	AT5G53890	1.0E-100	96%	
BraA03g52520	AT5G51100	5.0E-38	46%	
BraA03g52510				CD2
BraA03g52500	AT5G57710	1.0E-09	14%	
BraA03g52490				
BraA03g52480	AT5G60010	0.0E+00	91%	
BraA03g52470				
BraA03g52460				
BraA03g52450				
BraA03g52440				
BraA03g52430	AT2G29900	3.0E-166	100%	
BraA03g52420	AT2G37050	1.0E-13	9%	
BraA03g52410	AT2G33790	7.0E-25	97%	
BraA03g52400	AT2G29890	0.0E+00	99%	
BraA03g52390	AT2G29890	3.0E-61	96%	

BraA03g52380	AT2G29760	0.0E+00	100%	J
BraA03g52370	AT1G52950	1.0E-15	80%	
BraA03g52360				
BraA03g52350	AT2G44300	1.0E-05	43%	
BraA03g52340				
BraA03g52330				
BraA03g52320	AT2G29770	2.0E-30	95%	
BraA03g52310	AT2G30150	8.0E-41	95%	
BraA03g52300	AT2G36750	2.0E-43	98%	
BraA03g52290				
BraA03g52280				
BraA03g52270	AT2G35280	5.0E-10	43%	
BraA03g52260				
BraA03g52250				
BraA03g52240				
BraA03g52230				
BraA03g52220				
BraA03g52210				
BraA03g52200	AT2G38060	6.0E-94	97%	
BraA03g52190				
BraA03g52180				
BraA03g52170				
BraA03g52160				
BraA03g52150				
BraA03g52140				
BraA03g52130				
BraA03g52120	AT2G39060	6.0E-57	77%	
BraA03g52110				
BraA03g52100				
BraA03g52090	AT2G46690	2.0E-10	35%	
BraA03g52080	AT2G36950	2.0E-21	100%	
BraA03g52070	AT2G42820	6.0E-25	82%	
BraA03g52060	AT2G42200	2.0E-29	27%	
BraA03g52050	AT2G29980	0.0E+00	100%	
BraA03g52040	AT2G29990	0.0E+00	100%	
BraA03g52030				
BraA03g52020	AT2G30000	5.0E-60	99%	
BraA03g52010				
BraA03g52000	AT2G44940	7.0E-31	53%	
BraA03g51990				
BraA03g51980	AT2G45660	6.0E-26	81%	
BraA03g51970	AT2G30020	1.0E-171	100%	
BraA03g51960				
BraA03g51950				
BraA03g51940	AT2G30040	1.0E-168	97%	
BraA03g51930	AT2G30050	1.0E-169	100%	
BraA03g51920	AT2G30060	1.0E-104	100%	
BraA03g51910				
BraA03g51900	AT2G30070	0.0E+00	90%	
BraA03g51890	AT2G29830	2.0E-36	93%	
BraA03g51880	AT2G29800	1.0E-34	91%	
BraA03g51870	AT2G30110	0.0E+00	94%	
BraA03g51860	AT2G30130	2.0E-86	75%	
BraA03g51850	AT2G30140	0.0E+00	96%	
BraA03g51840	AT2G41910	3.0E-101	98%	
BraA03g51830				
BraA03g51820	AT2G30200	2.0E-86	93%	

BraA03g51810	AT2G29990	2.0E-71	92%
BraA03g51800	AT2G30000	5.0E-60	99%
BraA03g51790	AT2G44940	8.0E-31	53%
BraA03g51780			
BraA03g51770	AT2G45660	6.0E-26	81%
BraA03g51760			
BraA03g51750	AT2G30020	6.0E-171	100%
BraA03g51740			
BraA03g51730			
BraA03g51720	AT2G30040	5.0E-156	86%
BraA03g51710	AT2G30050	1.0E-169	100%
BraA03g51700	AT2G30060	1.0E-104	100%
BraA03g51690			
BraA03g51680	AT2G30070	0.0E+00	90%
BraA03g51670			
BraA03g51660	AT2G29830	2.0E-36	93%
BraA03g51650	AT2G29800	4.0E-35	91%
BraA03g51640	AT2G30110	0.0E+00	100%
BraA03g51630	AT2G30130	2.0E-86	75%
BraA03g51620	AT2G30140	0.0E+00	99%
BraA03g51610	AT2G41910	1.0E-102	92%
BraA03g51600	AT2G30200	2.0E-81	60%
BraA03g51590	AT2G30230	1.0E-47	75%
BraA03g51580	AT2G30240	0.0E+00	100%
BraA03g51570	AT2G30250	1.0E-170	99%
BraA03g51560	AT2G30270	1.0E-78	95%
BraA03g51550	AT2G30300	0.0E+00	100%
BraA03g51540	AT2G30320	0.0E+00	99%
BraA03g51530	AT2G29800	5.0E-31	95%
BraA03g51520	AT2G29800	3.0E-40	93%
BraA03g51510	AT2G30330	2.0E-68	99%
BraA03g51500	AT2G30350	1.0E-87	72%
BraA03g51490	AT2G30360	2.0E-21	90%
BraA03g51480	AT2G30370	8.0E-43	99%
BraA03g51470	AT2G30380	2.0E-38	80%
BraA03g51460	AT2G30400	8.0E-105	100%
BraA03g51450	AT2G43590	1.0E-128	100%
BraA03g51440	AT2G30410	1.0E-33	99%
BraA03g51430	AT2G30440	2.0E-49	84%
BraA03g51420	AT2G41910	5.0E-99	92%
BraA03g51410	AT2G30470	0.0E+00	99%
BraA03g51400	AT2G30490	0.0E+00	48%
BraA03g51390	AT2G30500	1.0E-151	100%
BraA03g51380	AT2G30500	2.0E-105	92%
BraA03g51370	AT2G30520	0.0E+00	100%
BraA03g51360	AT2G30520	6.0E-93	99%
BraA03g51350	AT2G30530	2.0E-116	92%
BraA03g51340	AT2G30540	1.0E-52	99%
BraA03g51330	AT2G30580	1.0E-41	73%
BraA03g51320	AT2G30590	9.0E-106	94%
BraA03g51310			
BraA03g51300	AT2G30410	1.0E-33	99%
BraA03g51290	AT2G43590	1.0E-128	100%
BraA03g51280	AT2G30330	4.0E-58	99%
BraA03g51270	AT2G30350	1.0E-54	99%
BraA03g51260			
BraA03g51250	AT2G30350	1.0E-87	72%

BraA03g51240	AT2G30360	2.0E-21	90%
BraA03g51230	AT2G30370	8.0E-43	99%
BraA03g51220	AT2G30620	6.0E-55	100%
BraA03g51210	AT2G30630	2.0E-24	80%
BraA03g51200	AT2G30700	0.0E+00	44%
BraA03g51190	AT2G30740	0.0E+00	97%
BraA03g51180			
BraA03g51170	AT2G30766	1.0E-09	98%
BraA03g51160	AT2G30770	0.0E+00	100%
BraA03g51150			
BraA03g51140	AT2G30820	3.0E-47	37%
BraA03g51130	AT2G30860	1.0E-121	100%
BraA03g51120	AT2G30870	5.0E-120	100%
BraA03g51110	AT2G30880	1.0E-79	68%
BraA03g51100	AT2G30890	8.0E-70	99%
BraA03g51090			
BraA03g51080	AT2G30910	0.0E+00	100%
BraA03g51070			
BraA03g51060	AT2G32120	3.0E-67	74%
BraA03g51050	AT2G31010	0.0E+00	100%
BraA03g51040	AT2G31030	1.0E-31	79%
BraA03g51030	AT2G31035	9.0E-31	42%
BraA03g51020			
BraA03g51010	AT2G31050	8.0E-30	92%
BraA03g51000	AT2G31060	0.0E+00	100%
BraA03g50990	AT2G31060	6.0E-33	28%
BraA03g50980	AT2G31100	0.0E+00	96%
BraA03g50970	AT2G42570	1.0E-141	87%
BraA03g50960	AT2G31130	1.0E-49	58%
BraA03g50950	AT2G31140	1.0E-111	99%
BraA03g50940	AT2G31150	3.0E-81	70%
BraA03g50930	AT2G31180	6.0E-93	100%
BraA03g50920	AT2G31230	7.0E-22	67%
BraA03g50910	AT2G30950	4.0E-42	35%
BraA03g50900	AT2G31270	0.0E+00	100%
BraA03g50890	AT2G31345	3.0E-21	97%
BraA03g50880	AT2G31350	2.0E-178	99%
BraA03g50870	AT2G31360	4.0E-153	100%
BraA03g50860	AT2G31370	2.0E-44	93%
BraA03g50850	AT2G31380	2.0E-54	85%
BraA03g50840	AT2G31390	0.0E+00	100%
BraA03g50830	AT2G31400	4.0E-32	89%
BraA03g50820	AT2G31410	3.0E-71	97%
BraA03g50810			
BraA03g50800	AT2G31440	1.0E-134	99%
BraA03g50790	AT2G31450	7.0E-164	95%
BraA03g50780	AT2G31490	3.0E-38	99%
BraA03g50770	AT2G31500	0.0E+00	99%
BraA03g50760	AT2G31510	0.0E+00	100%
BraA03g50750	AT2G31530	0.0E+00	100%
BraA03g50740			
BraA03g50730	AT2G31540	2.0E-148	100%
BraA03g50720			
BraA03g50710			
BraA03g50700	AT2G46495	9.0E-07	37%
BraA03g50690	AT2G42800	7.0E-05	27%
BraA03g50680	AT2G31610	3.0E-133	99%

BraA03g50670	AT2G31560	2.0E-73	99%
BraA03g50660	AT2G31570	6.0E-91	99%
BraA03g50650			
BraA03g50640			
BraA03g50630	AT2G31580	0.0E+00	100%
BraA03g50620	AT2G31600	5.0E-128	95%
BraA03g50610			
BraA03g50600	AT2G31610	6.0E-133	99%
BraA03g50590	AT2G31660	0.0E+00	95%
BraA03g50580	AT2G31680	5.0E-117	100%
BraA03g50570	AT2G31690	0.0E+00	99%
BraA03g50560	AT2G31710	8.0E-48	99%
BraA03g50550	AT2G31730	2.0E-55	96%
BraA03g50540	AT2G31750	0.0E+00	100%
BraA03g50530	AT2G31770	0.0E+00	96%
BraA03g50520	AT2G31800	0.0E+00	100%
BraA03g50510	AT2G31810	0.0E+00	100%
BraA03g50500	AT2G31840	2.0E-141	64%
BraA03g50490	AT2G31880	0.0E+00	100%
BraA03g50480	AT2G31890	0.0E+00	100%
BraA03g50470	AT2G31900	0.0E+00	100%
BraA03g50460			
BraA03g50450	AT2G31955	0.0E+00	100%
BraA03g50440	AT2G31970	0.0E+00	100%
BraA03g50430	AT2G31980	6.0E-53	92%
BraA03g50420	AT2G31990	0.0E+00	99%
BraA03g50410	AT2G32000	0.0E+00	100%
BraA03g50400	AT2G31470	4.0E-78	87%
BraA03g50390	AT2G32060	3.0E-61	99%
BraA03g50380			
BraA03g50370	AT2G32235	9.0E-81	99%
BraA03g50360	AT2G32240	1.0E-127	90%
BraA03g50350			
BraA03g50340	AT2G32260	3.0E-164	99%
BraA03g50330	AT2G32280	1.0E-84	98%
BraA03g50320	AT2G42730	9.0E-33	89%
BraA03g50310	AT2G42730	1.0E-09	51%
BraA03g50300	AT2G32300	1.0E-98	100%
BraA03g50290	AT2G32295	0.0E+00	98%
BraA03g50280	AT2G32340	1.0E-116	92%
BraA03g50270	AT2G32360	4.0E-44	81%
BraA03g50260	AT2G32390	0.0E+00	89%
BraA03g50250	AT2G32400	0.0E+00	100%
BraA03g50240	AT2G32460	0.0E+00	100%
BraA03g50230	AT2G32487	2.0E-06	33%
BraA03g50220	AT2G32500	4.0E-99	100%
BraA03g50210	AT2G32550	2.0E-71	96%
BraA03g50200	AT2G32630	0.0E+00	100%
BraA03g50190	AT2G32640	0.0E+00	80%
BraA03g50180	AT2G32700	5.0E-44	37%
BraA03g50170	AT2G32700	0.0E+00	100%
BraA03g50160	AT2G32710	1.0E-71	100%
BraA03g50150	AT2G32720	2.0E-71	99%
BraA03g50140	AT2G32730	0.0E+00	100%
BraA03g50130	AT2G32750	0.0E+00	100%
BraA03g50120	AT2G32760	0.0E+00	99%
BraA03g50110	AT2G32765	2.0E-23	92%

BraA03g50100	AT2G32770	0.0E+00	98%
BraA03g50090			
BraA03g50080	AT2G32790	7.0E-44	99%
BraA03g50070	AT2G32800	0.0E+00	100%
BraA03g50060	AT2G32820	2.0E-42	99%
BraA03g50050	AT2G33040	1.0E-12	100%
BraA03g50040			
BraA03g50030	AT2G32930	5.0E-127	99%
BraA03g50020	AT2G32940	0.0E+00	99%
BraA03g50010	AT2G32970	0.0E+00	78%
BraA03g50000	AT2G32980	2.0E-143	100%
BraA03g49990			
BraA03g49980			
BraA03g49970			
BraA03g49960			
BraA03g49950			
BraA03g49940	AT2G33040	3.0E-28	78%
BraA03g49930			
BraA03g49920	AT2G31400	1.0E-05	10%
BraA03g49910			
BraA03g49900	AT2G31470	2.0E-14	90%
BraA03g49890	AT2G31470	1.0E-29	84%
BraA03g49880	AT2G33040	0.0E+00	100%
BraA03g49870	AT2G32820	2.0E-42	99%
BraA03g49860	AT2G32800	0.0E+00	100%
BraA03g49850	AT2G33150	0.0E+00	100%
BraA03g49840	AT2G32830	0.0E+00	100%
BraA03g49830	AT2G32850	0.0E+00	100%
BraA03g49820	AT2G32870	2.0E-84	97%
BraA03g49810	AT2G32890	1.0E-16	99%
BraA03g49800	AT2G32900	0.0E+00	100%
BraA03g49790			
BraA03g49780	AT2G32970	0.0E+00	99%
BraA03g49770	AT2G32940	0.0E+00	99%
BraA03g49760	AT2G32930	5.0E-127	99%
BraA03g49750	AT2G30830	2.0E-66	62%
BraA03g49740			
BraA03g49730			
BraA03g49720			
BraA03g49710			
BraA03g49700	AT2G32900	0.0E+00	100%
BraA03g49690	AT2G32890	1.0E-16	99%
BraA03g49680	AT2G32870	2.0E-84	97%
BraA03g49670	AT2G32850	0.0E+00	100%
BraA03g49660	AT2G32830	0.0E+00	100%
BraA03g49650	AT2G33150	0.0E+00	100%
BraA03g49640	AT2G33160	0.0E+00	100%
BraA03g49630	AT2G33200	6.0E-38	92%
BraA03g49620	AT2G33200	1.0E-129	100%
BraA03g49610	AT2G33210	0.0E+00	96%
BraA03g49600	AT2G33240	0.0E+00	100%
BraA03g49590	AT2G33255	9.0E-134	100%
BraA03g49580	AT2G33310	5.0E-91	100%
BraA03g49570			
BraA03g49560	AT2G33360	2.0E-134	63%
BraA03g49550	AT2G33380	4.0E-125	100%
BraA03g49540			

BraA03g49530			
BraA03g49520	AT2G33410	2.0E-146	100%
BraA03g49510	AT2G33420	0.0E+00	100%
BraA03g49500			
BraA03g49490	AT2G33435	0.0E+00	60%
BraA03g49480	AT2G33460	2.0E-15	97%
BraA03g49470	AT2G33510	3.0E-67	99%
BraA03g49460	AT2G33520	9.0E-29	51%
BraA03g49450	AT2G33570	0.0E+00	100%
BraA03g49440	AT2G33585	2.0E-45	99%
BraA03g49430	AT2G33620	3.0E-113	100%
BraA03g49420	AT2G33630	0.0E+00	100%
BraA03g49410			
BraA03g49400	AT2G41690	2.0E-12	71%
BraA03g49390	AT2G33690	1.0E-29	99%
BraA03g49380	AT2G33720	1.0E-43	60%
BraA03g49370	AT2G33730	0.0E+00	86%
BraA03g49360	AT2G44030	2.0E-35	90%
BraA03g49350			
BraA03g49340	AT2G33810	3.0E-47	99%
BraA03g49330	AT2G33830	4.0E-54	99%
BraA03g49320	AT2G33835	4.0E-158	93%
BraA03g49310	AT2G38600	3.0E-54	99%
BraA03g49300	AT2G33870	9.0E-119	100%
BraA03g49290	AT2G33880	3.0E-52	43%
BraA03g49280			
BraA03g49270	AT2G34010	2.0E-65	100%
BraA03g49260	AT2G34020	6.0E-39	99%
BraA03g49250	AT2G33570	0.0E+00	100%
BraA03g49240			
BraA03g49230	AT2G33585	1.0E-45	99%
BraA03g49220	AT2G33620	3.0E-112	100%
BraA03g49210	AT2G33630	0.0E+00	100%
BraA03g49200			
BraA03g49190	AT2G41690	2.0E-11	71%
BraA03g49180	AT2G33690	1.0E-29	99%
BraA03g49170			
BraA03g49160	AT2G33720	5.0E-43	66%
BraA03g49150	AT2G33730	0.0E+00	86%
BraA03g49140	AT2G44030	2.0E-35	90%
BraA03g49130	AT2G33810	3.0E-47	99%
BraA03g49120	AT2G33830	4.0E-54	99%
BraA03g49110	AT2G33835	2.0E-162	95%
BraA03g49100	AT2G38600	5.0E-59	98%
BraA03g49090	AT2G33870	9.0E-119	100%
BraA03g49080	AT2G33880	6.0E-150	95%
BraA03g49070	AT2G33980	1.0E-07	34%
BraA03g49060	AT2G34010	2.0E-66	100%
BraA03g49050	AT2G34020	0.0E+00	100%
BraA03g49040	AT2G34040	0.0E+00	86%
BraA03g49030	AT2G34070	0.0E+00	94%
BraA03g49020	AT2G39850	2.0E-84	91%
BraA03g49010	AT2G34200	1.0E-75	91%
BraA03g49000	AT2G34210	0.0E+00	100%
BraA03g48990	AT2G34250	0.0E+00	100%
BraA03g48980			
BraA03g48970	AT2G34310	2.0E-15	66%

BraA03g48960	AT2G34330	2.0E-54	99%
BraA03g48950	AT2G34357	0.0E+00	99%
BraA03g48940	AT2G34360	0.0E+00	100%
BraA03g48930	AT2G34370	2.0E-170	100%
BraA03g48920	AT2G34410	0.0E+00	100%
BraA03g48910	AT2G34430	5.0E-150	100%
BraA03g48900	AT2G34480	8.0E-103	99%
BraA03g48890	AT2G34555	3.0E-157	99%
BraA03g48880	AT2G34560	0.0E+00	100%
BraA03g48870	AT2G34570	6.0E-126	100%
BraA03g48860	AT2G34600	3.0E-30	94%
BraA03g48850	AT2G34630	3.0E-172	75%
BraA03g48840	AT2G34670	0.0E+00	78%
BraA03g48830	AT2G34680	0.0E+00	99%
BraA03g48820	AT2G34680	0.0E+00	97%
BraA03g48810	AT2G34690	3.0E-101	100%
BraA03g48800			
BraA03g48790	AT2G34730	0.0E+00	99%
BraA03g48780	AT2G34740	6.0E-140	99%
BraA03g48770	AT2G34750	0.0E+00	100%
BraA03g48760	AT2G34770	1.0E-125	100%
BraA03g48750	AT2G34780	3.0E-114	63%
BraA03g48740			
BraA03g48730	AT2G34810	0.0E+00	100%
BraA03g48720			
BraA03g48710	AT2G34820	3.0E-110	99%
BraA03g48700	AT2G34840	7.0E-162	100%
BraA03g48690	AT2G34870	2.0E-07	33%
BraA03g48680	AT2G34910	5.0E-98	99%
BraA03g48670	AT2G34930	0.0E+00	99%
BraA03g48660			
BraA03g48650	AT2G35050	0.0E+00	100%
BraA03g48640	AT2G35060	1.0E-22	87%
BraA03g48630	AT2G44220	1.0E-22	80%
BraA03g48620	AT2G35100	0.0E+00	100%
BraA03g48610	AT2G35120	7.0E-81	99%
BraA03g48600			
BraA03g48590			
BraA03g48580	AT2G35155	0.0E+00	99%
BraA03g48570	AT2G35190	4.0E-137	99%
BraA03g48560			
BraA03g48550	AT2G35230	6.0E-109	100%
BraA03g48540	AT2G35240	1.0E-17	69%
BraA03g48530	AT2G35260	1.0E-169	100%
BraA03g48520	AT2G35270	5.0E-13	27%
BraA03g48510	AT2G35270	5.0E-97	96%
BraA03g48500	AT2G35320	3.0E-106	99%
BraA03g48490	AT2G43590	1.0E-105	97%
BraA03g48480			
BraA03g48470			
BraA03g48460			
BraA03g48450	AT2G35260	1.0E-169	100%
BraA03g48440	AT2G35240	1.0E-17	69%
BraA03g48430	AT2G35230	3.0E-113	100%
BraA03g48420			
BraA03g48410	AT2G35190	4.0E-137	99%
BraA03g48400	AT2G35155	0.0E+00	99%

BraA03g48390			
BraA03g48380			
BraA03g48370	AT2G35120	7.0E-81	99%
BraA03g48360	AT2G35100	3.0E-46	100%
BraA03g48350			
BraA03g48340	AT2G35270	5.0E-13	27%
BraA03g48330	AT2G35270	5.0E-97	96%
BraA03g48320	AT2G35320	4.0E-158	100%
BraA03g48310	AT2G43590	1.0E-105	97%
BraA03g48300			
BraA03g48290			
BraA03g48280	AT2G35530	5.0E-160	99%
BraA03g48270	AT2G35550	4.0E-90	100%
BraA03g48260	AT2G35580	2.0E-129	100%
BraA03g48250			
BraA03g48240	AT2G35612	3.0E-39	97%
BraA03g48230	AT2G35615	0.0E+00	99%
BraA03g48220	AT2G35620	0.0E+00	100%
BraA03g48210	AT2G35630	0.0E+00	100%
BraA03g48200	AT2G40050	0.0E+00	97%
BraA03g48190	AT2G35650	0.0E+00	100%
BraA03g48180	AT2G35840	0.0E+00	100%
BraA03g48170			
BraA03g48160	AT2G41890	5.0E-11	61%
BraA03g48150	AT2G35800	0.0E+00	100%
BraA03g48140	AT2G35795	4.0E-58	99%
BraA03g48130	AT2G35800	2.0E-35	63%
BraA03g48120	AT2G35790	2.0E-111	100%
BraA03g48110	AT2G47160	1.0E-18	47%
BraA03g48100	AT2G35765	7.0E-28	99%
BraA03g48090	AT2G35760	2.0E-87	100%
BraA03g48080			
BraA03g48070	AT2G35700	2.0E-20	53%
BraA03g48060			
BraA03g48050			
BraA03g48040	AT2G35690	0.0E+00	100%
BraA03g48030	AT2G35680	1.0E-165	100%
BraA03g48020			
BraA03g48010	AT2G38995	2.0E-78	100%
BraA03g48000	AT2G38995	7.0E-29	89%
BraA03g47990	AT2G35658	1.0E-31	99%
BraA03g47980	AT2G35650	3.0E-38	98%
BraA03g47970	AT2G35860	0.0E+00	100%
BraA03g47960	AT2G35880	5.0E-142	100%
BraA03g47950			
BraA03g47940	AT2G38590	4.0E-11	95%
BraA03g47930	AT2G35890	2.0E-14	48%
BraA03g47920	AT2G38590	5.0E-09	94%
BraA03g47910	AT2G35900	1.0E-74	99%
BraA03g47900	AT2G35910	3.0E-72	99%
BraA03g47890	AT2G35910	1.0E-62	99%
BraA03g47880	AT2G35920	0.0E+00	95%
BraA03g47870			
BraA03g47860	AT2G35930	0.0E+00	100%
BraA03g47850	AT2G35940	0.0E+00	100%
BraA03g47840			
BraA03g47830	AT2G36100	7.0E-89	100%

BraA03g47820	AT2G36020	7.0E-130	100%
BraA03g47810	AT2G36010	0.0E+00	96%
BraA03g47800	AT2G36000	4.0E-33	84%
BraA03g47790	AT2G36190	0.0E+00	58%
BraA03g47780			
BraA03g47770	AT2G36210	2.0E-62	99%
BraA03g47760	AT2G36230	2.0E-161	97%
BraA03g47750	AT2G36305	4.0E-148	100%
BraA03g47740			
BraA03g47730	AT2G36310	1.0E-179	100%
BraA03g47720	AT2G36320	2.0E-68	99%
BraA03g47710	AT2G36325	1.0E-143	100%
BraA03g47700	AT2G42960	2.0E-67	52%
BraA03g47690	AT2G36380	0.0E+00	100%
BraA03g47680	AT2G36400	6.0E-148	100%
BraA03g47670	AT2G36410	2.0E-56	99%
BraA03g47660	AT2G36410	3.0E-86	99%
BraA03g47650	AT2G36420	6.0E-142	99%
BraA03g47640	AT2G36450	6.0E-23	99%
BraA03g47630	AT2G36470	3.0E-131	100%
BraA03g47620	AT2G36530	0.0E+00	100%
BraA03g47610	AT2G36560	3.0E-50	75%
BraA03g47600	AT2G36570	0.0E+00	96%
BraA03g47590	AT2G36570	0.0E+00	97%
BraA03g47580			
BraA03g47570	AT2G32560	9.0E-05	38%
BraA03g47560	AT2G36890	9.0E-22	100%
BraA03g47550	AT2G36880	0.0E+00	100%
BraA03g47540	AT2G36870	2.0E-97	81%
BraA03g47530	AT2G34320	5.0E-04	85%
BraA03g47520	AT2G36800	0.0E+00	99%
BraA03g47510	AT2G36770	0.0E+00	100%
BraA03g47500	AT2G36780	0.0E+00	100%
BraA03g47490	AT2G36650	2.0E-43	81%
BraA03g47480	AT2G36650	1.0E-14	99%
BraA03g47470			
BraA03g47460			
BraA03g47450	AT2G36895	2.0E-12	100%
BraA03g47440			
BraA03g47430	AT2G36910	0.0E+00	98%
BraA03g47420	AT2G36930	2.0E-60	98%
BraA03g47410	AT2G36950	4.0E-127	100%
BraA03g47400	AT2G36985	9.0E-25	98%
BraA03g47390	AT2G36990	3.0E-35	99%
BraA03g47380	AT2G36990	1.0E-59	97%
BraA03g47370	AT2G37020	8.0E-146	99%
BraA03g47360	AT2G37025	4.0E-128	95%
BraA03g47350	AT2G37030	1.0E-56	99%
BraA03g47340	AT2G37050	0.0E+00	100%
BraA03g47330	AT2G37060	4.0E-84	99%
BraA03g47320	AT2G37080	0.0E+00	100%
BraA03g47310			
BraA03g47300	AT2G37100	1.0E-10	21%
BraA03g47290	AT2G37110	8.0E-121	100%
BraA03g47280	AT2G37130	3.0E-77	97%
BraA03g47270	AT2G37130	5.0E-90	99%
BraA03g47260	AT2G37150	0.0E+00	99%

BraA03g47250	AT2G37170	3.0E-157	100%
BraA03g47240	AT2G37170	5.0E-158	100%
BraA03g47230	AT2G37190	2.0E-91	99%
BraA03g47220	AT2G37435	2.0E-04	40%
BraA03g47210	AT2G37195	2.0E-59	92%
BraA03g47200	AT2G37210	4.0E-115	100%
BraA03g47190	AT2G37230	0.0E+00	74%
BraA03g47180	AT2G32120	1.0E-70	78%
BraA03g47170	AT2G37250	1.0E-154	100%
BraA03g47160	AT2G37260	0.0E+00	100%
BraA03g47150	AT2G37270	2.0E-118	100%
BraA03g47140	AT2G37300	2.0E-19	52%
BraA03g47130	AT2G37340	4.0E-117	100%
BraA03g47120	AT2G37380	2.0E-84	100%
BraA03g47110	AT2G37400	4.0E-129	99%
BraA03g47100	AT2G37410	1.0E-23	99%
BraA03g47090	AT2G39310	0.0E+00	100%
BraA03g47080	AT2G39390	4.0E-62	99%
BraA03g47070	AT2G32250	8.0E-100	88%
BraA03g47060	AT2G39360	0.0E+00	99%
BraA03g47050	AT2G39340	2.0E-19	99%
BraA03g47040			
BraA03g47030	AT2G39480	0.0E+00	100%
BraA03g47020	AT2G39460	8.0E-48	45%
BraA03g47010	AT2G39435	6.0E-136	100%
BraA03g47000	AT2G39430	4.0E-149	100%
BraA03g46990	AT2G39420	7.0E-42	66%
BraA03g46980	AT2G39620	0.0E+00	100%
BraA03g46970	AT2G39630	2.0E-179	99%
BraA03g46960	AT2G39650	9.0E-143	95%
BraA03g46950	AT2G39660	0.0E+00	100%
BraA03g46940	AT2G39700	6.0E-146	98%
BraA03g46930			
BraA03g46920	AT2G39720	3.0E-134	89%
BraA03g46910	AT2G43400	1.0E-12	26%
BraA03g46900	AT2G39730	0.0E+00	100%
BraA03g46890	AT2G39750	0.0E+00	100%
BraA03g46880			
BraA03g46870	AT2G39760	0.0E+00	92%
BraA03g46860	AT2G46870	2.0E-04	50%
BraA03g46850	AT2G39770	8.0E-172	100%
BraA03g46840	AT2G39800	0.0E+00	100%
BraA03g46830	AT2G39805	2.0E-144	100%
BraA03g46820	AT2G39830	0.0E+00	98%
BraA03g46810			
BraA03g46800	AT2G39830	6.0E-26	85%
BraA03g46790	AT2G39840	0.0E+00	99%
BraA03g46780	AT2G39890	0.0E+00	62%
BraA03g46770	AT2G39910	8.0E-180	95%
BraA03g46760	AT2G39940	0.0E+00	100%
BraA03g46750	AT2G39960	1.0E-55	91%
BraA03g46740	AT2G39960	2.0E-42	97%
BraA03g46730	AT2G39990	6.0E-152	100%
BraA03g46720	AT2G40000	0.0E+00	100%
BraA03g46710	AT2G40010	2.0E-164	100%
BraA03g46700	AT2G38120	0.0E+00	91%
BraA03g46690	AT2G40020	6.0E-35	72%

BraA03g46680	AT2G40030	0.0E+00	98%
BraA03g46670	AT2G40060	6.0E-119	100%
BraA03g46660	AT2G40080	2.0E-48	98%
BraA03g46650	AT2G40085	6.0E-22	69%
BraA03g46640	AT2G40090	0.0E+00	100%
BraA03g46630			
BraA03g46620	AT2G40095	8.0E-112	100%
BraA03g46610	AT2G40116	0.0E+00	100%
BraA03g46600	AT2G40130	0.0E+00	50%
BraA03g46590	AT2G40140	0.0E+00	100%
BraA03g46580	AT2G40150	0.0E+00	99%
BraA03g46570	AT2G40160	0.0E+00	100%
BraA03g46560	AT2G40170	1.0E-32	99%
BraA03g46550	AT2G40180	5.0E-173	99%
BraA03g46540	AT2G39415	4.0E-21	13%
BraA03g46530	AT2G40190	2.0E-42	82%
BraA03g46520			
BraA03g46510			
BraA03g46500			
BraA03g46490	AT2G40200	6.0E-94	100%
BraA03g46480	AT2G40220	2.0E-108	100%
BraA03g46470	AT2G40230	0.0E+00	100%
BraA03g46460	AT2G40260	4.0E-63	96%
BraA03g46450	AT2G40270	2.0E-21	47%
BraA03g46440			
BraA03g46430	AT2G40280	0.0E+00	100%
BraA03g46420	AT2G40290	2.0E-176	99%
BraA03g46410	AT2G40310	0.0E+00	100%
BraA03g46400	AT2G40330	2.0E-103	100%
BraA03g46390			
BraA03g46380	AT2G40450	8.0E-68	99%
BraA03g46370	AT2G40450	5.0E-70	99%
BraA03g46360	AT2G40460	0.0E+00	99%
BraA03g46350	AT2G40470	2.0E-100	100%
BraA03g46340	AT2G40475	4.0E-55	99%
BraA03g46330	AT2G40480	0.0E+00	100%
BraA03g46320	AT2G34320	1.0E-08	70%
BraA03g46310	AT2G40530	2.0E-26	99%
BraA03g46300	AT2G43000	7.0E-04	62%
BraA03g46290	AT2G40590	4.0E-63	89%
BraA03g46280	AT2G40610	4.0E-137	100%
BraA03g46270	AT2G40620	2.0E-116	98%
BraA03g46260	AT2G40630	5.0E-171	100%
BraA03g46250	AT2G40640	4.0E-85	87%
BraA03g46240	AT2G41140	0.0E+00	100%
BraA03g46230			
BraA03g46220	AT2G41180	3.0E-54	99%
BraA03g46210	AT2G41190	0.0E+00	96%
BraA03g46200	AT2G41200	3.0E-65	99%
BraA03g46190	AT2G41210	0.0E+00	100%
BraA03g46180	AT2G41220	0.0E+00	88%
BraA03g46170	AT2G41220	0.0E+00	100%
BraA03g46160	AT2G41225	1.0E-09	43%
BraA03g46150	AT2G41290	0.0E+00	100%
BraA03g46140	AT2G41300	1.0E-171	86%
BraA03g46130	AT2G41310	2.0E-96	100%
BraA03g46120	AT2G41330	2.0E-147	95%

BraA03g46110	AT2G41342	8.0E-29	83%
BraA03g46100	AT2G41350	3.0E-165	100%
BraA03g46090	AT2G41420	4.0E-09	57%
BraA03g46080			
BraA03g46070	AT2G41420	9.0E-07	49%
BraA03g46060	AT2G41430	2.0E-58	99%
BraA03g46050	AT2G41460	5.0E-156	71%
BraA03g46040	AT2G41480	1.0E-173	99%
BraA03g46030	AT2G41510	0.0E+00	100%
BraA03g46020	AT2G41530	8.0E-162	100%
BraA03g46010	AT2G41540	0.0E+00	100%
BraA03g46000	AT2G41630	2.0E-131	89%
BraA03g45990	AT2G41660	4.0E-118	100%
BraA03g45980	AT2G41680	0.0E+00	99%
BraA03g45970			
BraA03g45960	AT2G41690	3.0E-103	100%
BraA03g45950	AT2G41700	0.0E+00	100%
BraA03g45940	AT2G41710	0.0E+00	100%
BraA03g45930	AT2G41740	0.0E+00	100%
BraA03g45920	AT2G41760	8.0E-118	99%
BraA03g45910	AT2G41770	0.0E+00	100%
BraA03g45900			
BraA03g45890	AT2G41790	1.0E-49	88%
BraA03g45880	AT2G41840	4.0E-123	84%
BraA03g45870	AT2G41900	0.0E+00	100%
BraA03g45860	AT2G41905	2.0E-26	98%
BraA03g45850	AT2G41940	2.0E-92	100%
BraA03g45840	AT2G41945	5.0E-78	98%
BraA03g45830	AT2G41960	0.0E+00	81%
BraA03g45820	AT2G41970	0.0E+00	100%
BraA03g45810	AT2G41940	2.0E-92	100%
BraA03g45800	AT2G41945	1.0E-75	98%
BraA03g45790	AT2G41960	0.0E+00	81%
BraA03g45780	AT2G41970	0.0E+00	100%
BraA03g45770	AT2G41980	5.0E-166	100%
BraA03g45760	AT2G42030	4.0E-69	96%
BraA03g45750	AT2G42040	4.0E-62	99%
BraA03g45740	AT2G42040	2.0E-11	80%
BraA03g45730	AT2G42060	3.0E-125	100%
BraA03g45720	AT2G42070	2.0E-141	100%
BraA03g45710			
BraA03g45700	AT5G10630	2.0E-10	76%
BraA03g45690			
BraA03g45680			
BraA03g45670			
BraA03g45660			
BraA03g45650	AT2G42330	2.0E-14	26%
BraA03g45640	AT2G42380	3.0E-114	100%
BraA03g45630	AT2G42430	9.0E-122	100%
BraA03g45620	AT2G42460	5.0E-39	91%
BraA03g45610	AT2G42460	3.0E-44	88%
BraA03g45600	AT2G42490	0.0E+00	100%
BraA03g45590	AT2G41890	6.0E-14	89%
BraA03g45580			
BraA03g45570	AT2G42500	0.0E+00	100%
BraA03g45560	AT2G42530	7.0E-52	99%
BraA03g45550	AT2G42520	7.0E-10	34%

BraA03g45540	AT2G42530	1.0E-47	93%
BraA03g45530	AT2G42560	1.0E-24	58%
BraA03g45520	AT2G42580	0.0E+00	100%
BraA03g45510	AT2G42590	7.0E-146	100%
BraA03g45500	AT2G42610	7.0E-90	93%
BraA03g45490	AT2G42660	1.0E-78	93%
BraA03g45480	AT2G42670	7.0E-76	99%
BraA03g45470	AT2G42680	1.0E-74	99%
BraA03g45460	AT2G42700	1.0E-35	66%
BraA03g45450	AT2G42780	8.0E-111	69%
BraA03g45440	AT2G42860	4.0E-04	44%
BraA03g45430	AT2G42870	5.0E-38	99%
BraA03g45420	AT2G42880	0.0E+00	99%
BraA03g45410	AT2G44220	3.0E-78	87%
BraA03g45400	AT2G44250	1.0E-22	71%
BraA03g45390	AT2G42890	5.0E-120	99%
BraA03g45380	AT2G42890	0.0E+00	100%
BraA03g45370	AT2G42910	0.0E+00	100%
BraA03g45360	AT2G42940	2.0E-125	100%
BraA03g45350	AT2G43000	9.0E-137	98%
BraA03g45340	AT2G43010	2.0E-157	100%
BraA03g45330	AT2G43020	0.0E+00	100%
BraA03g45320	AT2G43030	7.0E-125	100%
BraA03g45310	AT2G43040	0.0E+00	100%
BraA03g45300	AT2G45180	3.0E-21	73%
BraA03g45290	AT2G43090	3.0E-119	99%
BraA03g45280	AT2G43120	1.0E-173	100%
BraA03g45270	AT2G43130	1.0E-118	98%
BraA03g45260	AT2G43140	4.0E-71	90%
BraA03g45250	AT2G43150	5.0E-10	16%
BraA03g45240	AT2G43160	0.0E+00	100%
BraA03g45230	AT2G43180	0.0E+00	100%
BraA03g45220	AT2G39660	3.0E-109	77%
BraA03g45210			
BraA03g45200	AT2G43200	0.0E+00	87%
BraA03g45190	AT2G43235	0.0E+00	99%
BraA03g45180	AT2G43240	0.0E+00	100%
BraA03g45170	AT2G43290	3.0E-100	100%
BraA03g45160	AT2G43320	0.0E+00	100%
BraA03g45150			
BraA03g45140			
BraA03g45130			
BraA03g45120	AT2G43340	3.0E-65	99%
BraA03g45110	AT2G43350	3.0E-87	99%
BraA03g45100	AT2G43360	0.0E+00	100%
BraA03g45090	AT2G43370	9.0E-144	100%
BraA03g45080	AT2G43445	6.0E-14	76%
BraA03g45070	AT2G43430	0.0E+00	100%
BraA03g45060	AT2G43460	2.0E-33	19%
BraA03g45050			
BraA03g45040	AT2G43500	0.0E+00	99%
BraA03g45030	AT2G43520	9.0E-33	97%
BraA03g45020	AT2G43500	0.0E+00	100%
BraA03g45010	AT2G43520	2.0E-37	97%
BraA03g45000	AT2G43520	3.0E-32	97%
BraA03g44990	AT2G43560	8.0E-16	97%
BraA03g44980	AT2G43570	2.0E-110	98%

BraA03g44970	AT2G43580	5.0E-124	100%
BraA03g44960			
BraA03g44950	AT2G43590	1.0E-131	100%
BraA03g44940	AT2G43600	1.0E-112	92%
BraA03g44930	AT2G43600	2.0E-142	99%
BraA03g44920	AT2G43610	9.0E-151	98%
BraA03g44910			
BraA03g44900	AT2G43620	3.0E-142	97%
BraA03g44890	AT2G43640	5.0E-54	95%
BraA03g44880	AT2G43650	0.0E+00	100%
BraA03g44870	AT2G43680	0.0E+00	100%
BraA03g44860	AT2G43700	0.0E+00	100%
BraA03g44850	AT2G43700	0.0E+00	100%
BraA03g44840	AT2G43710	0.0E+00	100%
BraA03g44830	AT2G43730	2.0E-18	78%
BraA03g44820	AT2G43730	4.0E-32	78%
BraA03g44810	AT2G43730	4.0E-41	100%
BraA03g44800	AT2G43780	4.0E-28	99%
BraA03g44790	AT2G43790	0.0E+00	99%
BraA03g44780	AT2G40310	0.0E+00	100%
BraA03g44770	AT2G43790	1.0E-53	62%
BraA03g44760	AT2G43800	0.0E+00	93%
BraA03g44750	AT2G43820	0.0E+00	99%
BraA03g44740	AT2G43820	0.0E+00	91%
BraA03g44730			
BraA03g44720			
BraA03g44710	AT2G38255	3.0E-149	94%
BraA03g44700			
BraA03g44690	AT2G43820	0.0E+00	100%
BraA03g44680	AT2G43820	0.0E+00	100%
BraA03g44670	AT2G43860	0.0E+00	100%
BraA03g44660	AT2G43870	0.0E+00	100%
BraA03g44650	AT2G43900	0.0E+00	86%
BraA03g44640	AT2G43945	2.0E-123	91%
BraA03g44630	AT2G43950	3.0E-163	65%
BraA03g44620			
BraA03g44610	AT2G44000	5.0E-62	99%
BraA03g44600	AT2G44010	1.0E-33	99%
BraA03g44590	AT2G44040	0.0E+00	99%
BraA03g44580	AT2G44050	5.0E-112	100%
BraA03g44570	AT2G44060	4.0E-157	97%
BraA03g44560	AT2G44065	1.0E-110	100%
BraA03g44550	AT2G44080	6.0E-27	91%
BraA03g44540	AT2G44120	8.0E-132	100%
BraA03g44530			
BraA03g44520	AT2G44130	1.0E-161	97%
BraA03g44510	AT2G44150	9.0E-146	99%
BraA03g44500	AT2G44180	0.0E+00	99%
BraA03g44490	AT2G44190	7.0E-158	97%
BraA03g44480	AT2G44200	1.0E-180	100%
BraA03g44470	AT2G44210	0.0E+00	100%
BraA03g44460	AT2G44260	0.0E+00	56%
BraA03g44450	AT2G44310	3.0E-70	99%
BraA03g44440	AT2G44330	6.0E-59	98%
BraA03g44430			
BraA03g44420	AT2G44340	1.0E-64	98%
BraA03g44410	AT2G44350	0.0E+00	84%

BraA03g44400	AT2G43410	9.0E-43	57%
BraA03g44390	AT2G44380	2.0E-99	98%
BraA03g44380	AT2G32470	2.0E-05	49%
BraA03g44370	AT2G44440	3.0E-168	92%
BraA03g44360	AT2G44570	0.0E+00	100%
BraA03g44350	AT2G44610	2.0E-121	100%
BraA03g44340	AT2G44620	2.0E-57	99%
BraA03g44330	AT2G44640	0.0E+00	100%
BraA03g44320	AT2G44670	1.0E-41	99%
BraA03g44310	AT2G44680	2.0E-136	100%
BraA03g44300	AT2G44730	9.0E-125	98%
BraA03g44290	AT2G44740	2.0E-100	96%
BraA03g44280	AT2G44745	6.0E-28	91%
BraA03g44270	AT2G44770	2.0E-101	92%
BraA03g44260	AT2G44790	2.0E-51	99%
BraA03g44250	AT2G34670	2.0E-37	39%
BraA03g44240	AT2G44940	8.0E-33	95%
BraA03g44230	AT2G45060	6.0E-92	99%
BraA03g44220	AT2G45070	2.0E-29	16%
BraA03g44210	AT2G45080	1.0E-121	97%
BraA03g44200	AT2G45110	4.0E-120	99%
BraA03g44190	AT2G40360	0.0E+00	100%
BraA03g44180	AT2G45120	5.0E-15	59%
BraA03g44170	AT2G45120	4.0E-75	100%
BraA03g44160	AT2G45130	9.0E-118	99%
BraA03g44150	AT2G45140	7.0E-72	100%
BraA03g44140	AT2G45150	1.0E-180	100%
BraA03g44130	AT2G45160	0.0E+00	100%
BraA03g44120			
BraA03g44110	AT2G45170	1.0E-63	99%
BraA03g44100	AT2G45180	5.0E-49	99%
BraA03g44090	AT2G45190	1.0E-105	95%
BraA03g44080			
BraA03g44070	AT2G45200	1.0E-130	93%
BraA03g44060	AT2G45210	6.0E-55	98%
BraA03g44050	AT3G22290	3.0E-04	27%
BraA03g44040	AT2G45220	0.0E+00	100%
BraA03g44030	AT2G45640	6.0E-41	91%
BraA03g44020	AT2G45510	0.0E+00	99%
BraA03g44010	AT2G45510	0.0E+00	99%
BraA03g44000	AT2G45460	1.0E-07	37%
BraA03g43990	AT2G45450	7.0E-20	91%
BraA03g43980	AT2G32630	1.0E-12	52%
BraA03g43970			
BraA03g43960			
BraA03g43950			
BraA03g43940	AT2G45320	0.0E+00	100%
BraA03g43930	AT2G45310	0.0E+00	100%
BraA03g43920	AT2G45300	0.0E+00	100%
BraA03g43910	AT2G45260	0.0E+00	100%
BraA03g43900	AT2G45220	0.0E+00	100%
BraA03g43890	AT3G22290	3.0E-04	27%
BraA03g43880	AT2G45210	6.0E-55	98%
BraA03g43870	AT2G45200	3.0E-44	84%
BraA03g43860	AT2G45650	7.0E-85	99%
BraA03g43850	AT2G45660	2.0E-74	99%
BraA03g43840	AT2G45670	0.0E+00	100%

BraA03g43830	AT2G45680	9.0E-112	100%
BraA03g43820			
BraA03g43810			
BraA03g43800			
BraA03g43790	AT2G45690	9.0E-175	100%
BraA03g43780	AT2G45695	1.0E-47	99%
BraA03g43770	AT2G45710	4.0E-44	99%
BraA03g43760	AT2G45720	0.0E+00	93%
BraA03g43750	AT2G45730	0.0E+00	100%
BraA03g43740	AT2G45740	6.0E-126	79%
BraA03g43730	AT2G45760	2.0E-82	100%
BraA03g43720	AT2G45790	9.0E-139	98%
BraA03g43710	AT2G45800	6.0E-100	90%
BraA03g43700	AT2G45940	2.0E-131	50%
BraA03g43690	AT2G45950	4.0E-125	85%
BraA03g43680	AT2G45960	9.0E-166	100%
BraA03g43670	AT2G46000	7.0E-79	100%
BraA03g43660	AT2G46020	0.0E+00	100%
BraA03g43650	AT2G46030	2.0E-85	81%
BraA03g43640			
BraA03g43630	AT2G45910	6.0E-29	56%
BraA03g43620			
BraA03g43610	AT2G46140	9.0E-84	99%
BraA03g43600	AT2G46225	2.0E-145	100%
BraA03g43590	AT2G46260	3.0E-128	100%
BraA03g43580	AT2G46260	9.0E-150	99%
BraA03g43570	AT2G46300	3.0E-104	100%
BraA03g43560	AT2G46330	1.0E-32	99%
BraA03g43550	AT2G46340	0.0E+00	99%
BraA03g43540	AT2G46370	0.0E+00	100%
BraA03g43530	AT2G46375	4.0E-21	65%
BraA03g43520	AT2G46390	6.0E-19	98%
BraA03g43510	AT2G46400	3.0E-109	100%
BraA03g43500	AT2G46410	1.0E-41	99%
BraA03g43490	AT2G46470	8.0E-117	92%
BraA03g43480	AT2G46590	3.0E-147	96%
BraA03g43470	AT2G46800	8.0E-177	100%
BraA03g43460	AT2G46850	0.0E+00	95%
BraA03g43450	AT2G46870	3.0E-128	100%
BraA03g43440			
BraA03g43430	AT2G46940	4.0E-96	100%
BraA03g43420	AT2G46970	6.0E-162	98%
BraA03g43410			
BraA03g43400	AT2G47040	0.0E+00	100%
BraA03g43390	AT2G47050	1.0E-108	99%
BraA03g43380	AT2G47060	2.0E-37	65%
BraA03g43370	AT2G47110	2.0E-88	99%
BraA03g43360	AT2G47115	1.0E-126	98%
BraA03g43350	AT2G47130	5.0E-96	99%
BraA03g43340	AT2G47130	2.0E-121	97%
BraA03g43330	AT2G47160	0.0E+00	100%
BraA03g43320	AT2G47170	8.0E-105	68%
BraA03g43310	AT2G35030	5.0E-80	97%
BraA03g43300	AT2G47250	0.0E+00	100%
BraA03g43290	AT2G36410	1.0E-38	89%
BraA03g43280	AT2G47310	0.0E+00	99%
BraA03g43270	AT2G47320	1.0E-62	100%

BraA03g43260	AT2G46940	3.0E-91	100%
BraA03g43250			
BraA03g43240	AT2G46950	2.0E-59	67%
BraA03g43230	AT2G46950	0.0E+00	100%
BraA03g43220			
BraA03g43210	AT2G46970	4.0E-160	100%
BraA03g43200	AT2G35030	9.0E-07	20%
BraA03g43190			
BraA03g43180	AT2G46980	0.0E+00	66%
BraA03g43170			
BraA03g43160			
BraA03g43150	AT2G47000	0.0E+00	99%
BraA03g43140	AT2G47010	0.0E+00	90%
BraA03g43130	AT2G47020	0.0E+00	100%
BraA03g43120	AT2G46780	2.0E-122	100%
BraA03g43110	AT2G46790	2.0E-163	99%
BraA03g43100	AT2G46800	1.0E-169	100%
BraA03g43090	AT2G46810	6.0E-115	98%
BraA03g43080	AT2G46820	4.0E-56	99%
BraA03g43070	AT2G46830	0.0E+00	100%
BraA03g43060	AT2G46860	3.0E-116	99%
BraA03g43050	AT2G46870	4.0E-118	99%
BraA03g43040	AT2G46880	4.0E-178	99%
BraA03g43030	AT2G46890	6.0E-162	100%
BraA03g43020	AT2G46900	0.0E+00	100%
BraA03g43010	AT2G46910	1.0E-149	100%
BraA03g43000	AT2G46915	0.0E+00	100%
BraA03g42990	AT2G46920	0.0E+00	100%
BraA03g42980	AT2G46930	0.0E+00	99%
BraA03g42970	AT2G35030	5.0E-80	97%
BraA03g42960	AT2G47250	0.0E+00	100%
BraA03g42950	AT2G36410	1.0E-38	89%
BraA03g42940	AT2G47310	0.0E+00	87%
BraA03g42930	AT2G47320	1.0E-104	100%
BraA03g42920			
BraA03g42910	AT2G47310	4.0E-88	61%
BraA03g42900	AT2G47320	2.0E-28	64%
BraA03g42890	AT2G47330	3.0E-27	99%
BraA03g42880	AT2G47330	0.0E+00	98%
BraA03g42870	AT2G47350	5.0E-154	90%
BraA03g42860	AT2G47360	4.0E-119	99%
BraA03g42850	AT2G47380	2.0E-24	98%
BraA03g42840	AT2G47400	2.0E-54	99%
BraA03g42830	AT2G47420	1.0E-172	100%
BraA03g42820	AT2G47460	4.0E-120	100%
BraA03g42810	AT2G47470	0.0E+00	100%
BraA03g42800	AT2G47610	1.0E-137	100%
BraA03g42790	AT2G47620	0.0E+00	100%
BraA03g42780	AT2G47710	2.0E-85	99%
BraA03g42770			
BraA03g42760	AT2G47730	2.0E-107	96%
BraA03g42750	AT2G44480	3.0E-93	85%
BraA03g42740	AT2G47760	7.0E-47	78%
BraA03g42730			
BraA03g42720	AT2G47810	4.0E-77	99%
BraA03g42710			
BraA03g42700	AT2G39450	2.0E-05	58%

BraA03g42690	AT2G44020	1.0E-05	64%	
BraA03g42680	AT2G32930	2.0E-54	85%	
BraA03g42670				
BraA03g42660	AT2G29950	7.0E-37	83%	
BraA03g42650	AT2G29940	0.0E+00	100%	
BraA03g42640	AT2G29930	3.0E-34	21%	
BraA03g42630	AT2G30140	3.0E-13	86%	
BraA03g42620				
BraA03g42610	AT2G28880	2.0E-52	65%	
BraA03g42600				
BraA03g42590				
BraA03g42580	AT2G29400	0.0E+00	100%	I
BraA03g42570	AT2G29370	2.0E-70	99%	
BraA03g42560				
BraA03g42550	AT2G29320	7.0E-122	98%	
BraA03g42540	AT2G29310	6.0E-138	100%	
BraA03g42530	AT2G28930	8.0E-175	100%	
BraA03g42520	AT2G28870	4.0E-42	99%	
BraA03g42510	AT2G28840	0.0E+00	90%	
BraA03g42500	AT2G28740	3.0E-54	99%	
BraA03g42490	AT2G28725	7.0E-31	90%	
BraA03g42480	AT2G28720	1.0E-55	92%	
BraA03g42470	AT2G28610	2.0E-86	100%	
BraA03g42460				
BraA03g42450	AT2G28605	2.0E-96	100%	
BraA03g42440	AT2G28590	8.0E-77	66%	
BraA03g42430	AT2G28550	2.0E-157	81%	
BraA03g42420	AT2G28520	0.0E+00	99%	
BraA03g42410				
BraA03g42400				
BraA03g42390	AT2G28500	2.0E-96	100%	
BraA03g42380	AT2G28480	4.0E-158	94%	
BraA03g42370	AT2G28470	0.0E+00	100%	
BraA03g42360	AT2G28470	1.0E-57	99%	
BraA03g42350	AT2G28390	0.0E+00	100%	
BraA03g42340				
BraA03g42330	AT2G28160	2.0E-153	100%	
BraA03g42320				
BraA03g42310	AT2G28105	4.0E-75	97%	
BraA03g42300	AT2G28085	5.0E-65	99%	
BraA03g42290				
BraA03g42280				
BraA03g42270				
BraA03g42260				
BraA03g42250	AT2G28000	0.0E+00	98%	
BraA03g42240	AT2G27900	0.0E+00	100%	
BraA03g42230	AT2G27720	1.0E-30	99%	
BraA03g42220	AT2G27035	5.0E-08	31%	
BraA03g42210	AT2G27610	2.0E-24	62%	
BraA03g42200	AT2G27600	0.0E+00	100%	
BraA03g42190				
BraA03g42180				
BraA03g42170	AT2G27580	2.0E-65	99%	
BraA03g42160	AT2G27550	1.0E-99	99%	
BraA03g42150				
BraA03g42140	AT2G27510	1.0E-76	99%	
BraA03g42130				

BraA03g42120				
BraA03g42110	AT2G27500	0.0E+00	95%	
BraA03g42100	AT2G27490	8.0E-117	100%	
BraA03g42090	AT2G27480	4.0E-103	99%	
BraA03g42080	AT2G27430	3.0E-59	90%	
BraA03g42070	AT2G27430	6.0E-139	100%	
BraA03g42060				
BraA03g42050	AT4G19670	2.0E-15	45%	
BraA03g42040	AT2G27520	7.0E-09	59%	
BraA03g42030	AT2G27370	2.0E-112	100%	
BraA03g42020	AT2G27330	3.0E-45	90%	
BraA03g42010	AT2G27310	1.0E-145	81%	
BraA03g42000				
BraA03g41990	AT2G27260	9.0E-78	96%	
BraA03g41980				
BraA03g41970	AT2G27180	9.0E-27	68%	
BraA03g41960	AT2G27180	7.0E-20	69%	
BraA03g41950	AT2G27170	0.0E+00	99%	
BraA03g41940	AT2G27140	6.0E-83	100%	
BraA03g41930	AT2G27610	2.0E-58	82%	
BraA03g41920	AT2G27080	3.0E-124	100%	
BraA03g41910	AT2G27035	5.0E-41	36%	
BraA03g41900	AT2G27050	0.0E+00	100%	
BraA03g41890	AT2G26990	0.0E+00	100%	
BraA03g41880				
BraA03g41870	AT2G26960	3.0E-121	90%	
BraA03g41860	AT2G26840	5.0E-109	97%	
BraA03g41850	AT2G26810	1.0E-123	99%	
BraA03g41840	AT2G26800	0.0E+00	93%	
BraA03g41830				CD3
BraA03g41820				
BraA03g41810				
BraA03g41800				
BraA03g41790				
BraA03g41780				
BraA03g41770				
BraA03g41760				
BraA03g41750				
BraA03g41740				
BraA03g41730				
BraA03g41720				
BraA03g41710				
BraA03g41700				
BraA03g41690				
BraA03g41680				
BraA03g41670				
BraA03g41660				
BraA03g41650				
BraA03g41640				
BraA03g41630				
BraA03g41620				
BraA03g41610				
BraA03g41600				
BraA03g41590				
BraA03g41580				
BraA03g41570				
BraA03g41560				

BraA03g41550				
BraA03g41540				
BraA03g41530				
BraA03g41520				
BraA03g41510				
BraA03g41500				
BraA03g41490				
BraA03g41480				
BraA03g41470				
BraA03g41460				
BraA03g41450				
BraA03g41440				
BraA03g41430				
BraA03g41420				
BraA03g41410				
BraA03g41400				
BraA03g41390				
BraA03g41380				
BraA03g41370				
BraA03g41360				
BraA03g41350				
BraA03g41340				
BraA03g41330				
BraA03g41320				
BraA03g41310				
BraA03g41300				
BraA03g41290				
BraA03g41280				
BraA03g41270				
BraA03g41260				
BraA03g41250				
BraA03g41240				
BraA03g41230				
BraA03g41220				
BraA03g41210				
BraA03g41200				
BraA03g41190				
BraA03g41180				
BraA03g41170				
BraA03g41160				
BraA03g41150				
BraA03g41140				
BraA03g41130	AT5G49770	0.0E+00	99%	W
BraA03g41120	AT5G49700	1.0E-119	100%	
BraA03g41110				
BraA03g41100				
BraA03g41090				
BraA03g41080	AT5G49890	0.0E+00	100%	
BraA03g41070				
BraA03g41060	AT5G49900	0.0E+00	100%	
BraA03g41050	AT5G49920	4.0E-101	97%	
BraA03g41040	AT5G49930	0.0E+00	98%	
BraA03g41030	AT5G49940	9.0E-99	100%	
BraA03g41020	AT5G49945	0.0E+00	95%	
BraA03g41010	AT5G49970	0.0E+00	100%	
BraA03g41000	AT5G49980	0.0E+00	100%	
BraA03g40990	AT5G50000	0.0E+00	100%	

BraA03g40980	AT5G50020	0.0E+00	100%	
BraA03g40970	AT5G50230	1.0E-15	60%	
BraA03g40960				
BraA03g40950	AT5G50090	2.0E-71	99%	
BraA03g40940	AT5G50110	1.0E-121	73%	
BraA03g40930	AT5G50120	0.0E+00	99%	
BraA03g40920	AT5G50175	8.0E-36	99%	
BraA03g40910				
BraA03g40900	AT5G50180	0.0E+00	100%	
BraA03g40890				
BraA03g40880	AT5G50200	3.0E-91	100%	
BraA03g40870	AT5G50240	1.0E-131	99%	
BraA03g40860	AT5G50260	0.0E+00	100%	
BraA03g40850	AT5G50280	0.0E+00	99%	
BraA03g40840	AT5G50290	3.0E-159	100%	
BraA03g40830	AT5G50310	0.0E+00	98%	
BraA03g40820				
BraA03g40810	AT5G49770	5.0E-43	33%	
BraA03g40800	AT5G50320	0.0E+00	100%	
BraA03g40790	AT5G50340	0.0E+00	100%	
BraA03g40780	AT5G50350	0.0E+00	100%	
BraA03g40770				
BraA03g40760	AT5G50370	2.0E-141	99%	
BraA03g40750				
BraA03g40740	AT5G50375	1.0E-153	100%	
BraA03g40730	AT5G50700	2.0E-172	99%	
BraA03g40720	AT5G50460	7.0E-36	99%	
BraA03g40710	AT5G50750	0.0E+00	100%	
BraA03g40700				
BraA03g40690	AT5G50760	1.0E-39	84%	
BraA03g40680				CD4
BraA03g40670				
BraA03g40660				
BraA03g40650				
BraA03g40640				
BraA03g40630				
BraA03g40620				
BraA03g40610				
BraA03g40600				
BraA03g40590	AT4G12410	1.0E-08	55%	
BraA03g40580	AT4G09900	4.0E-38	98%	
BraA03g40570	AT4G09900	8.0E-37	97%	
BraA03g40560				
BraA03g40550				
BraA03g40540				
BraA03g40530				
BraA03g40520				
BraA03g40510				
BraA03g40500	AT4G11280	1.0E-144	92%	
BraA03g40490				
BraA03g40480				
BraA03g40470				
BraA03g40460	AT4G12460	0.0E+00	90%	
BraA03g40450	AT4G09770	5.0E-45	93%	
BraA03g40440	AT4G10613	9.0E-18	48%	
BraA03g40430				
BraA03g40420				

BraA03g40410				
BraA03g40400	AT4G11690	7.0E-09	68%	
BraA03g40390	AT4G11690	1.0E-15	82%	
BraA03g40380				
BraA03g40370				
BraA03g40360				
BraA03g40350				
BraA03g40340				
BraA03g40330				
BraA03g40320	AT4G12020	2.0E-96	58%	
BraA03g40310				
BraA03g40300				
BraA03g40290				
BraA03g40280				
BraA03g40270				
BraA03g40260				
BraA03g40250				
BraA03g40240				
BraA03g40230				
BraA03g40220				
BraA03g40210				
BraA03g40200				
BraA03g40190				
BraA03g40180				
BraA03g40170	AT4G09160	9.0E-15	55%	
BraA03g40160				
BraA03g40150	AT4G11900	8.0E-51	32%	
BraA03g40140	AT4G10820	5.0E-08	27%	
BraA03g40130	AT4G08900	0.0E+00	99%	P
BraA03g40120	AT4G08950	6.0E-154	95%	
BraA03g40110	AT4G08960	0.0E+00	100%	
BraA03g40100	AT4G08980	3.0E-173	97%	
BraA03g40090	AT4G09010	0.0E+00	100%	
BraA03g40080	AT4G11770	3.0E-34	92%	
BraA03g40070				
BraA03g40060	AT4G11770	3.0E-33	96%	
BraA03g40050	AT4G09012	4.0E-42	62%	
BraA03g40040	AT4G09020	0.0E+00	100%	
BraA03g40030	AT4G11750	2.0E-24	87%	
BraA03g40020	AT4G11770	7.0E-34	96%	
BraA03g40010	AT4G09030	3.0E-18	99%	
BraA03g40000				
BraA03g39990	AT4G09060	5.0E-147	99%	
BraA03g39980	AT4G09545	3.0E-18	66%	
BraA03g39970	AT4G09130	8.0E-132	99%	
BraA03g39960	AT4G09140	0.0E+00	99%	
BraA03g39950	AT4G09160	8.0E-62	92%	
BraA03g39940	AT4G09170	2.0E-27	99%	
BraA03g39930	AT4G11460	7.0E-31	82%	
BraA03g39920	AT4G09190	9.0E-96	98%	
BraA03g39910	AT4G09340	0.0E+00	99%	
BraA03g39900				
BraA03g39890				
BraA03g39880	AT4G09460	3.0E-47	92%	
BraA03g39870				
BraA03g39860	AT4G09510	0.0E+00	100%	
BraA03g39850	AT4G09530	1.0E-39	97%	

BraA03g39840	AT4G09560	2.0E-119	99%
BraA03g39830	AT4G09570	0.0E+00	99%
BraA03g39820	AT4G09490	8.0E-05	83%
BraA03g39810	AT4G09600	1.0E-41	99%
BraA03g39800	AT4G09490	6.0E-06	45%
BraA03g39790	AT4G09620	2.0E-105	100%
BraA03g39780	AT4G11900	7.0E-16	45%
BraA03g39770	AT4G09090	3.0E-13	89%
BraA03g39760	AT4G09650	3.0E-115	99%
BraA03g39750	AT4G09462	9.0E-16	84%
BraA03g39740	AT4G09670	0.0E+00	100%
BraA03g39730	AT4G09720	1.0E-112	100%
BraA03g39720	AT4G09730	0.0E+00	100%
BraA03g39710			
BraA03g39700	AT4G09800	3.0E-87	99%
BraA03g39690	AT4G09810	0.0E+00	98%
BraA03g39680			
BraA03g39670			
BraA03g39660			
BraA03g39650			
BraA03g39640			
BraA03g39630	AT4G10090	2.0E-78	98%
BraA03g39620	AT4G11900	8.0E-39	67%
BraA03g39610			
BraA03g39600	AT4G10010	0.0E+00	99%
BraA03g39590	AT4G10010	0.0E+00	72%
BraA03g39580			
BraA03g39570			
BraA03g39560			
BraA03g39550	AT4G10040	9.0E-53	99%
BraA03g39540	AT4G10090	1.0E-111	100%
BraA03g39530			
BraA03g39520			
BraA03g39510			
BraA03g39500	AT4G09980	0.0E+00	100%
BraA03g39490	AT4G09960	4.0E-76	99%
BraA03g39480	AT4G09950	8.0E-153	100%
BraA03g39470	AT4G09900	0.0E+00	208%
BraA03g39460			
BraA03g39450			
BraA03g39440			
BraA03g39430			
BraA03g39420			
BraA03g39410			
BraA03g39400			
BraA03g39390	AT4G11730	1.0E-53	63%
BraA03g39380			
BraA03g39370			
BraA03g39360			
BraA03g39350			
BraA03g39340			
BraA03g39330			
BraA03g39320			
BraA03g39310	AT4G09900	0.0E+00	100%
BraA03g39300			
BraA03g39290			
BraA03g39280			

BraA03g39270			
BraA03g39260			
BraA03g39250	AT4G09890	1.0E-46	99%
BraA03g39240	AT4G09830	9.0E-57	90%
BraA03g39230	AT4G10030	1.0E-158	100%
BraA03g39220	AT4G10040	8.0E-63	99%
BraA03g39210	AT4G10050	0.0E+00	99%
BraA03g39200	AT4G10170	2.0E-115	100%
BraA03g39190	AT4G10180	0.0E+00	100%
BraA03g39180	AT4G10250	9.0E-98	99%
BraA03g39170	AT4G10260	0.0E+00	99%
BraA03g39160	AT4G10280	1.0E-59	99%
BraA03g39150			
BraA03g39140	AT4G10300	4.0E-43	94%
BraA03g39130	AT4G10300	2.0E-62	99%
BraA03g39120			
BraA03g39110	AT4G10310	0.0E+00	100%
BraA03g39100	AT4G10340	1.0E-137	100%
BraA03g39090	AT4G10350	0.0E+00	100%
BraA03g39080	AT4G10380	6.0E-155	100%
BraA03g39070	AT4G10480	9.0E-77	99%
BraA03g39060	AT4G10490	4.0E-89	72%
BraA03g39050	AT4G10500	6.0E-179	100%
BraA03g39040	AT4G10570	0.0E+00	100%
BraA03g39030	AT4G10610	1.0E-156	100%
BraA03g39020	AT4G10630	2.0E-131	100%
BraA03g39010	AT4G10640	0.0E+00	100%
BraA03g39000			
BraA03g38990			
BraA03g38980	AT4G10710	0.0E+00	100%
BraA03g38970			
BraA03g38960			
BraA03g38950	AT4G10790	0.0E+00	100%
BraA03g38940	AT4G10810	3.0E-34	99%
BraA03g38930	AT4G08980	2.0E-05	40%
BraA03g38920	AT4G10840	1.0E-79	100%
BraA03g38910	AT4G10710	0.0E+00	100%
BraA03g38900	AT4G10930	4.0E-176	97%
BraA03g38890	AT4G10920	2.0E-63	92%
BraA03g38880			
BraA03g38870			
BraA03g38860			
BraA03g38850	AT4G10850	2.0E-103	100%
BraA03g38840	AT4G10840	0.0E+00	100%
BraA03g38830	AT4G08980	2.0E-05	40%
BraA03g38820	AT4G10810	3.0E-34	99%
BraA03g38810	AT4G10790	0.0E+00	100%
BraA03g38800			
BraA03g38790			
BraA03g38780	AT4G11040	1.0E-63	100%
BraA03g38770			
BraA03g38760	AT4G10970	5.0E-78	100%
BraA03g38750	AT4G10930	0.0E+00	74%
BraA03g38740	AT4G11360	2.0E-59	97%
BraA03g38730	AT4G11260	5.0E-176	100%
BraA03g38720	AT4G11240	3.0E-174	99%
BraA03g38710	AT4G11220	6.0E-131	100%

BraA03g38700	AT4G11210	3.0E-72	99%
BraA03g38690	AT4G11160	0.0E+00	99%
BraA03g38680			
BraA03g38670			
BraA03g38660			
BraA03g38650	AT4G11150	6.0E-125	100%
BraA03g38640	AT4G11140	1.0E-84	94%
BraA03g38630			
BraA03g38620	AT4G11120	2.0E-29	99%
BraA03g38610	AT4G11120	4.0E-142	100%
BraA03g38600			
BraA03g38590			
BraA03g38580	AT4G11050	0.0E+00	97%
BraA03g38570	AT4G11040	1.0E-59	20%
BraA03g38560			
BraA03g38550	AT4G11380	3.0E-63	78%
BraA03g38540	AT4G11420	0.0E+00	100%
BraA03g38530	AT4G11470	8.0E-128	76%
BraA03g38520			
BraA03g38510	AT4G11480	1.0E-115	79%
BraA03g38500			
BraA03g38490	AT4G11480	7.0E-85	82%
BraA03g38480	AT4G11560	0.0E+00	100%
BraA03g38470	AT4G11580	1.0E-74	100%
BraA03g38460	AT4G11660	1.0E-113	90%
BraA03g38450	AT4G10660	2.0E-69	75%
BraA03g38440	AT4G12370	1.0E-69	89%
BraA03g38430	AT4G08455	6.0E-130	249%
BraA03g38420			
BraA03g38410	AT4G11850	0.0E+00	99%
BraA03g38400	AT4G11850	0.0E+00	98%
BraA03g38390	AT4G11890	8.0E-151	100%
BraA03g38380	AT4G11910	8.0E-130	99%
BraA03g38370			
BraA03g38360	AT4G11920	0.0E+00	95%
BraA03g38350	AT4G11960	6.0E-158	98%
BraA03g38340	AT4G12020	0.0E+00	64%
BraA03g38330	AT4G12010	0.0E+00	100%
BraA03g38320	AT4G12030	1.0E-147	86%
BraA03g38310			
BraA03g38300	AT4G12120	0.0E+00	98%
BraA03g38290	AT4G12130	0.0E+00	100%
BraA03g38280	AT4G11770	2.0E-49	96%
BraA03g38270	AT4G12130	7.0E-18	96%
BraA03g38260	AT4G12210	7.0E-15	48%
BraA03g38250	AT4G09570	7.0E-06	83%
BraA03g38240	AT4G12230	0.0E+00	100%
BraA03g38230	AT4G12320	0.0E+00	100%
BraA03g38220	AT4G12320	0.0E+00	100%
BraA03g38210	AT4G10660	2.0E-108	92%
BraA03g38200	AT4G10660	2.0E-107	82%
BraA03g38190	AT4G12410	3.0E-73	99%
BraA03g38180	AT4G12420	0.0E+00	98%
BraA03g38170	AT4G12460	4.0E-53	88%
BraA03g38160			
BraA03g38150	AT4G02730	2.0E-09	56%
BraA03g38140	AT4G02730	5.0E-04	87%

BraA03g38130	AT4G04340	0.0E+00	100%	
BraA03g38120	AT3G24690	3.0E-38	98%	
BraA03g38110				
BraA03g38100				
BraA03g38090	AT4G01720	1.0E-59	91%	
BraA03g38080				
BraA03g38070				
BraA03g38060	AT4G00970	1.0E-83	49%	
BraA03g38050				
BraA03g38040	AT4G00960	5.0E-13	49%	
BraA03g38030				
BraA03g38020	AT4G00970	3.0E-50	33%	
BraA03g38010	AT4G00970	4.0E-90	93%	
BraA03g38000	AT4G00970	2.0E-54	80%	
BraA03g37990	AT4G00960	4.0E-15	82%	
BraA03g37980	AT4G00970	2.0E-42	76%	
BraA03g37970	AT4G00970	1.0E-61	77%	
BraA03g37960				
BraA03g37950	AT4G03500	7.0E-06	78%	
BraA03g37940				
BraA03g37930	AT4G03080	2.0E-06	14%	
BraA03g37920				
BraA03g37910				
BraA03g37900				
BraA03g37890	AT4G02050	4.0E-29	83%	
BraA03g37880	AT4G00160	2.0E-09	15%	
BraA03g37870				
BraA03g37860				
BraA03g37850	AT4G00730	0.0E+00	95%	
BraA03g37840	AT4G02480	5.0E-20	30%	
BraA03g37830				
BraA03g37820	AT4G02730	5.0E-07	11%	
BraA03g37810	AT4G03520	2.0E-10	19%	
BraA03g37800				
BraA03g37790				
BraA03g37780	AT4G02890	3.0E-130	75%	
BraA03g37770	AT4G00700	8.0E-06	27%	
BraA03g37760	AT4G00090	6.0E-07	13%	
BraA03g37750				
BraA03g37740				
BraA03g37730	AT4G03630	1.0E-61	59%	O
BraA03g37720	AT4G03630	4.0E-33	95%	
BraA03g37710	AT4G03610	1.0E-151	100%	
BraA03g37700	AT4G03565	2.0E-78	92%	
BraA03g37690	AT4G03560	0.0E+00	100%	
BraA03g37680	AT4G03520	3.0E-76	99%	
BraA03g37670				
BraA03g37660	AT4G03470	3.0E-111	100%	
BraA03g37650	AT4G03440	7.0E-38	93%	
BraA03g37640	AT4G03430	0.0E+00	85%	
BraA03g37630				
BraA03g37620	AT4G03420	3.0E-164	100%	
BraA03g37610	AT4G03410	4.0E-155	99%	
BraA03g37600	AT4G03390	0.0E+00	98%	
BraA03g37590				
BraA03g37580				
BraA03g37570				

BraA03g37560	AT5G48240	6.0E-05	15%
BraA03g37550	AT4G00315	1.0E-13	53%
BraA03g37540			
BraA03g37530			
BraA03g37520	AT4G03340	0.0E+00	100%
BraA03g37510	AT4G03290	4.0E-69	99%
BraA03g37500	AT4G03280	1.0E-110	98%
BraA03g37490	AT4G03260	0.0E+00	100%
BraA03g37480	AT4G03240	3.0E-82	98%
BraA03g37470	AT4G03210	1.0E-162	100%
BraA03g37460	AT4G03200	2.0E-12	42%
BraA03g37450	AT4G03200	2.0E-16	97%
BraA03g37440	AT4G03190	0.0E+00	100%
BraA03g37430	AT4G00360	2.0E-09	49%
BraA03g37420	AT4G03120	6.0E-59	99%
BraA03g37410	AT4G03100	0.0E+00	100%
BraA03g37400	AT4G03080	0.0E+00	99%
BraA03g37390	AT4G03070	6.0E-137	98%
BraA03g37380	AT4G03050	9.0E-77	71%
BraA03g37370			
BraA03g37360	AT4G03020	0.0E+00	100%
BraA03g37350	AT4G02980	7.0E-104	99%
BraA03g37340			
BraA03g37330			
BraA03g37320	AT4G02930	0.0E+00	100%
BraA03g37310	AT4G02900	0.0E+00	99%
BraA03g37300	AT4G00620	9.0E-144	100%
BraA03g37290	AT4G02880	0.0E+00	100%
BraA03g37280			
BraA03g37270	AT4G02850	4.0E-126	98%
BraA03g37260	AT4G02850	4.0E-144	100%
BraA03g37250	AT4G02840	1.0E-51	84%
BraA03g37240	AT4G02800	1.0E-133	99%
BraA03g37230	AT4G02790	0.0E+00	100%
BraA03g37220	AT4G02780	0.0E+00	92%
BraA03g37210	AT4G02740	2.0E-116	94%
BraA03g37200	AT4G02740	1.0E-136	90%
BraA03g37190			
BraA03g37180	AT4G02715	1.0E-45	98%
BraA03g37170			
BraA03g37160	AT4G02680	0.0E+00	100%
BraA03g37150	AT4G02660	0.0E+00	97%
BraA03g37140	AT4G02310	2.0E-11	89%
BraA03g37130	AT4G02620	1.0E-70	99%
BraA03g37120	AT4G02580	1.0E-126	98%
BraA03g37110	AT4G02570	0.0E+00	100%
BraA03g37100	AT4G02570	1.0E-109	100%
BraA03g37090	AT4G02520	8.0E-105	99%
BraA03g37080	AT4G02520	3.0E-109	99%
BraA03g37070	AT4G02500	0.0E+00	100%
BraA03g37060	AT4G02480	0.0E+00	100%
BraA03g37050	AT4G02480	8.0E-38	97%
BraA03g37040	AT4G02450	9.0E-66	59%
BraA03g37030			
BraA03g37020	AT4G02420	0.0E+00	100%
BraA03g37010	AT4G02420	2.0E-145	97%
BraA03g37000			

BraA03g36990	AT4G02405	4.0E-126	99%
BraA03g36980	AT4G02390	0.0E+00	100%
BraA03g36970	AT4G02380	6.0E-41	99%
BraA03g36960			
BraA03g36950	AT4G02370	3.0E-73	99%
BraA03g36940	AT4G02250	1.0E-58	65%
BraA03g36930	AT4G02230	8.0E-88	96%
BraA03g36920			
BraA03g36910			
BraA03g36900	AT4G01010	4.0E-92	85%
BraA03g36890	AT4G02170	9.0E-66	99%
BraA03g36880	AT4G02150	0.0E+00	100%
BraA03g36870	AT4G02130	6.0E-171	100%
BraA03g36860			
BraA03g36850			
BraA03g36840			
BraA03g36830			
BraA03g36820	AT4G02110	7.0E-171	92%
BraA03g36810			
BraA03g36800	AT4G02130	6.0E-171	100%
BraA03g36790	AT4G02150	9.0E-23	98%
BraA03g36780	AT4G02110	0.0E+00	66%
BraA03g36770			
BraA03g36760			
BraA03g36750			
BraA03g36740			
BraA03g36730	AT4G02100	0.0E+00	100%
BraA03g36720	AT4G03430	2.0E-37	13%
BraA03g36710	AT4G02080	1.0E-111	99%
BraA03g36700	AT4G02070	0.0E+00	100%
BraA03g36690	AT4G02060	0.0E+00	100%
BraA03g36680	AT4G02050	0.0E+00	99%
BraA03g36670	AT4G02040	3.0E-26	99%
BraA03g36660	AT4G02010	0.0E+00	100%
BraA03g36650	AT4G01995	5.0E-118	100%
BraA03g36640	AT4G01990	0.0E+00	99%
BraA03g36630			
BraA03g36620	AT4G01950	0.0E+00	100%
BraA03g36610			
BraA03g36600			
BraA03g36590			
BraA03g36580	AT4G01940	1.0E-92	37%
BraA03g36570	AT4G01770	6.0E-69	99%
BraA03g36560			
BraA03g36550			
BraA03g36540	AT4G01220	3.0E-81	83%
BraA03g36530	AT4G01220	1.0E-127	88%
BraA03g36520	AT4G01900	9.0E-90	99%
BraA03g36510	AT4G01897	1.0E-56	98%
BraA03g36500	AT4G01895	1.0E-24	69%
BraA03g36490	AT4G01890	2.0E-53	98%
BraA03g36480	AT4G01890	7.0E-126	99%
BraA03g36470	AT4G01880	0.0E+00	100%
BraA03g36460	AT4G01870	0.0E+00	100%
BraA03g36450	AT4G01850	0.0E+00	100%
BraA03g36440	AT4G01800	0.0E+00	100%
BraA03g36430	AT4G01790	3.0E-73	92%

BraA03g36420	AT4G01735	2.0E-12	30%
BraA03g36410	AT4G01670	1.0E-87	100%
BraA03g36400	AT5G18880	4.0E-66	87%
BraA03g36390	AT4G01650	4.0E-127	99%
BraA03g36380	AT4G01630	7.0E-142	100%
BraA03g36370	AT4G01575	5.0E-52	99%
BraA03g36360			
BraA03g36350	AT4G01440	2.0E-28	99%
BraA03g36340			
BraA03g36330	AT4G00810	3.0E-43	99%
BraA03g36320	AT4G01400	0.0E+00	98%
BraA03g36310	AT4G01410	1.0E-102	100%
BraA03g36300	AT4G01450	2.0E-39	100%
BraA03g36290	AT4G00910	0.0E+00	100%
BraA03g36280	AT4G00860	6.0E-30	99%
BraA03g36270	AT4G00840	9.0E-149	100%
BraA03g36260	AT4G00830	0.0E+00	100%
BraA03g36250	AT4G00820	0.0E+00	98%
BraA03g36240	AT4G00990	0.0E+00	97%
BraA03g36230			
BraA03g36220	AT4G00990	1.0E-14	32%
BraA03g36210	AT4G00990	3.0E-51	88%
BraA03g36200	AT4G01000	1.0E-140	96%
BraA03g36190	AT4G01010	0.0E+00	99%
BraA03g36180	AT4G01026	8.0E-98	94%
BraA03g36170	AT4G01040	0.0E+00	44%
BraA03g36160	AT4G01060	2.0E-32	96%
BraA03g36150	AT4G01070	0.0E+00	100%
BraA03g36140	AT4G01090	0.0E+00	100%
BraA03g36130	AT4G01100	0.0E+00	100%
BraA03g36120	AT4G01110	3.0E-108	100%
BraA03g36110	AT4G01130	0.0E+00	99%
BraA03g36100	AT4G01150	2.0E-82	99%
BraA03g36090	AT4G01160	8.0E-143	98%
BraA03g36080	AT4G01210	0.0E+00	100%
BraA03g36070	AT4G01220	0.0E+00	100%
BraA03g36060	AT4G01270	0.0E+00	100%
BraA03g36050	AT4G01290	0.0E+00	100%
BraA03g36040	AT4G01310	9.0E-144	98%
BraA03g36030	AT4G01320	0.0E+00	100%
BraA03g36020	AT4G01370	0.0E+00	99%
BraA03g36010	AT4G00770	7.0E-157	99%
BraA03g36000	AT4G00755	2.0E-98	99%
BraA03g35990	AT4G00752	0.0E+00	100%
BraA03g35980	AT4G00740	0.0E+00	100%
BraA03g35970	AT4G00730	0.0E+00	99%
BraA03g35960	AT4G00730	8.0E-156	94%
BraA03g35950	AT4G00720	0.0E+00	100%
BraA03g35940	AT4G00710	0.0E+00	100%
BraA03g35930	AT4G00700	0.0E+00	100%
BraA03g35920	AT4G00670	1.0E-43	99%
BraA03g35910	AT4G00660	0.0E+00	98%
BraA03g35900	AT4G00585	9.0E-41	99%
BraA03g35890	AT4G00570	0.0E+00	100%
BraA03g35880	AT4G00525	2.0E-43	97%
BraA03g35870			
BraA03g35860	AT4G00500	0.0E+00	100%

BraA03g35850	AT4G00490	5.0E-04	56%	
BraA03g35840	AT4G00480	0.0E+00	99%	
BraA03g35830	AT4G00467	1.0E-43	81%	
BraA03g35820	AT4G00460	2.0E-175	68%	
BraA03g35810	AT4G00450	0.0E+00	98%	
BraA03g35800	AT4G00440	0.0E+00	100%	
BraA03g35790	AT4G00430	2.0E-164	99%	
BraA03g35780	AT4G00370	0.0E+00	100%	
BraA03g35770	AT4G00355	2.0E-75	67%	
BraA03g35760				
BraA03g35750	AT4G00170	6.0E-110	98%	
BraA03g35740	AT4G00120	1.0E-43	88%	
BraA03g35730	AT4G00050	2.0E-149	100%	
BraA03g35720				CD5
BraA03g35710				
BraA03g35700	AT3G02000	2.0E-61	99%	
BraA03g35690				
BraA03g35680				
BraA03g35670	AT3G04350	4.0E-110	90%	
BraA03g35660				
BraA03g35650	AT3G22930	5.0E-11	91%	
BraA03g35640				
BraA03g35630				
BraA03g35620				
BraA03g35610				
BraA03g35600	AT3G04140	2.0E-130	67%	
BraA03g35590				
BraA03g35580				
BraA03g35570				
BraA03g35560	AT3G23330	9.0E-94	90%	
BraA03g35550	AT3G18570	8.0E-07	64%	
BraA03g35540	AT3G10290	9.0E-20	76%	
BraA03g35530	AT3G06480	0.0E+00	65%	
BraA03g35520	AT3G06490	8.0E-11	25%	
BraA03g35510				
BraA03g35500				
BraA03g35490				
BraA03g35480				
BraA03g35470	AT3G23280	3.0E-04	21%	
BraA03g35460				
BraA03g35450	AT3G09840	2.0E-140	72%	
BraA03g35440	AT3G18400	6.0E-12	40%	
BraA03g35430				
BraA03g35420	AT3G06480	0.0E+00	79%	
BraA03g35410				
BraA03g35400	AT3G06490	8.0E-11	25%	
BraA03g35390	AT3G10940	4.0E-26	30%	
BraA03g35380				
BraA03g35370				
BraA03g35360	AT3G07360	3.0E-33	75%	
BraA03g35350				
BraA03g35340				
BraA03g35330				
BraA03g35320				
BraA03g35310				
BraA03g35300				
BraA03g35290				

BraA03g35280	AT3G07010	5.0E-47	57%	
BraA03g35270	AT3G17940	2.0E-77	87%	
BraA03g35260				
BraA03g35250	AT3G16510	5.0E-44	69%	
BraA03g35240				
BraA03g35230				
BraA03g35220				
BraA03g35210				
BraA03g35200				
BraA03g35190				
BraA03g35180				
BraA03g35170	AT3G21620	1.0E-117	98%	
BraA03g35160	AT3G17510	5.0E-37	37%	
BraA03g35150	AT3G25490	7.0E-85	86%	
BraA03g35140	AT3G05050	5.0E-122	60%	
BraA03g35130				
BraA03g35120				
BraA03g35110	AT3G25140	2.0E-100	78%	
BraA03g35100	AT3G19580	2.0E-05	9%	
BraA03g35090				
BraA03g35080	AT3G02080	3.0E-79	99%	
BraA03g35070	AT3G10510	4.0E-51	98%	
BraA03g35060	AT3G02130	0.0E+00	98%	F
BraA03g35050	AT3G02140	1.0E-105	100%	
BraA03g35040	AT3G02150	1.0E-113	82%	
BraA03g35030				
BraA03g35020	AT3G02160	5.0E-138	97%	
BraA03g35010	AT3G02170	0.0E+00	100%	
BraA03g35000	AT3G02180	1.0E-36	73%	
BraA03g34990	AT3G02200	0.0E+00	86%	
BraA03g34980	AT3G02230	0.0E+00	100%	
BraA03g34970				
BraA03g34960	AT3G02250	0.0E+00	100%	
BraA03g34950	AT3G02280	0.0E+00	100%	
BraA03g34940				
BraA03g34930	AT3G02350	0.0E+00	100%	
BraA03g34920				
BraA03g34910	AT3G02350	0.0E+00	100%	
BraA03g34900				
BraA03g34890	AT3G02380	2.0E-86	93%	
BraA03g34880	AT3G02400	2.0E-95	98%	
BraA03g34870	AT3G02460	0.0E+00	100%	
BraA03g34860	AT3G02470	0.0E+00	100%	
BraA03g34850	AT3G02480	6.0E-20	94%	
BraA03g34840	AT3G02490	0.0E+00	100%	
BraA03g34830				
BraA03g34820	AT3G02500	3.0E-91	98%	
BraA03g34810	AT3G02520	4.0E-143	100%	
BraA03g34800	AT3G02540	0.0E+00	100%	
BraA03g34790	AT3G02550	2.0E-122	100%	
BraA03g34780	AT3G02555	9.0E-48	99%	
BraA03g34770	AT3G02560	9.0E-100	99%	
BraA03g34760	AT3G02580	5.0E-146	100%	
BraA03g34750	AT3G02590	4.0E-100	95%	
BraA03g34740	AT3G02590	8.0E-144	96%	
BraA03g34730	AT3G02600	2.0E-166	98%	
BraA03g34720	AT3G02630	0.0E+00	100%	

BraA03g34710	AT3G02650	0.0E+00	98%
BraA03g34700	AT3G02660	0.0E+00	99%
BraA03g34690	AT3G02750	0.0E+00	100%
BraA03g34680	AT3G02760	1.0E-172	100%
BraA03g34670	AT3G02760	0.0E+00	100%
BraA03g34660	AT3G02770	3.0E-90	99%
BraA03g34650	AT3G02780	3.0E-145	100%
BraA03g34640	AT3G02800	1.0E-104	98%
BraA03g34630	AT3G02810	0.0E+00	98%
BraA03g34620	AT3G02830	1.0E-176	100%
BraA03g34610	AT3G02840	9.0E-159	100%
BraA03g34600	AT3G02850	0.0E+00	98%
BraA03g34590	AT3G02870	5.0E-119	99%
BraA03g34580			
BraA03g34570			
BraA03g34560	AT3G02970	4.0E-153	96%
BraA03g34550	AT3G02990	0.0E+00	100%
BraA03g34540	AT4G11580	1.0E-04	44%
BraA03g34530			
BraA03g34520	AT3G03090	0.0E+00	100%
BraA03g34510	AT3G03150	1.0E-47	97%
BraA03g34500	AT3G03160	4.0E-71	99%
BraA03g34490	AT3G03170	7.0E-61	99%
BraA03g34480	AT3G03272	4.0E-39	75%
BraA03g34470	AT3G03350	1.0E-15	71%
BraA03g34460	AT3G03430	1.0E-41	99%
BraA03g34450	AT3G03470	0.0E+00	100%
BraA03g34440	AT3G03490	2.0E-111	100%
BraA03g34430	AT3G03550	5.0E-109	99%
BraA03g34420	AT3G03560	0.0E+00	88%
BraA03g34410	AT3G03570	0.0E+00	100%
BraA03g34400	AT3G03600	3.0E-125	100%
BraA03g34390	AT3G03670	1.0E-151	100%
BraA03g34380	AT3G03710	0.0E+00	100%
BraA03g34370			
BraA03g34360			
BraA03g34350	AT3G03760	8.0E-52	98%
BraA03g34340	AT3G03760	3.0E-24	70%
BraA03g34330	AT3G03770	0.0E+00	100%
BraA03g34320			
BraA03g34310	AT3G03780	0.0E+00	100%
BraA03g34300	AT3G03810	0.0E+00	100%
BraA03g34290			
BraA03g34280			
BraA03g34270	AT3G03826	1.0E-15	93%
BraA03g34260	AT3G04290	0.0E+00	100%
BraA03g34250	AT3G04280	9.0E-61	99%
BraA03g34240	AT3G04230	9.0E-72	99%
BraA03g34230	AT3G04150	4.0E-106	97%
BraA03g34220	AT3G04160	0.0E+00	100%
BraA03g34210	AT3G04120	0.0E+00	100%
BraA03g34200	AT3G04050	0.0E+00	100%
BraA03g34190	AT3G03960	0.0E+00	99%
BraA03g34180	AT3G05650	3.0E-90	97%
BraA03g34170	AT3G03860	3.0E-149	100%
BraA03g34160			
BraA03g34150	AT3G04460	5.0E-105	100%

BraA03g34140	AT3G04400	8.0E-78	99%
BraA03g34130	AT3G04380	0.0E+00	68%
BraA03g34120	AT3G04360	3.0E-140	99%
BraA03g34110	AT3G04310	3.0E-83	99%
BraA03g34100	AT3G04300	1.0E-48	98%
BraA03g34090	AT3G04480	0.0E+00	100%
BraA03g34080	AT3G13510	4.0E-10	46%
BraA03g34070	AT3G04500	7.0E-73	78%
BraA03g34060	AT3G04510	4.0E-72	75%
BraA03g34050			
BraA03g34040	AT3G04620	1.0E-69	99%
BraA03g34030	AT3G04610	0.0E+00	100%
BraA03g34020	AT3G09550	7.0E-19	67%
BraA03g34010	AT3G18670	4.0E-12	84%
BraA03g34000	AT3G07000	2.0E-45	59%
BraA03g33990	AT3G04630	4.0E-109	98%
BraA03g33980	AT3G04650	0.0E+00	100%
BraA03g33970	AT3G04670	7.0E-99	96%
BraA03g33960	AT3G04680	2.0E-148	90%
BraA03g33950	AT3G04700	4.0E-80	99%
BraA03g33940	AT3G04710	0.0E+00	100%
BraA03g33930	AT3G04720	1.0E-47	85%
BraA03g33920	AT3G04720	4.0E-50	84%
BraA03g33910	AT3G04720	4.0E-103	100%
BraA03g33900	AT3G04810	6.0E-111	96%
BraA03g33890	AT3G04810	0.0E+00	100%
BraA03g33880	AT3G04820	0.0E+00	100%
BraA03g33870	AT3G04830	7.0E-168	100%
BraA03g33860	AT3G04840	2.0E-138	100%
BraA03g33850	AT3G04850	2.0E-159	99%
BraA03g33840	AT3G04903	5.0E-29	99%
BraA03g33830	AT3G04910	0.0E+00	99%
BraA03g33820			
BraA03g33810	AT3G04940	3.0E-160	75%
BraA03g33800	AT3G22560	4.0E-38	71%
BraA03g33790	AT3G04945	1.0E-24	99%
BraA03g33780	AT3G04950	1.0E-102	75%
BraA03g33770	AT3G05010	4.0E-143	91%
BraA03g33760	AT3G05020	3.0E-55	99%
BraA03g33750	AT3G05070	5.0E-45	99%
BraA03g33740	AT3G05090	0.0E+00	100%
BraA03g33730	AT3G05100	3.0E-161	99%
BraA03g33720	AT3G05140	0.0E+00	93%
BraA03g33710	AT3G05200	1.0E-178	100%
BraA03g33700	AT3G05220	3.0E-42	35%
BraA03g33690	AT3G05230	1.0E-89	99%
BraA03g33680	AT3G16740	1.0E-107	97%
BraA03g33670			
BraA03g33660	AT3G16750	4.0E-08	28%
BraA03g33650	AT3G05270	5.0E-34	99%
BraA03g33640	AT3G05280	6.0E-152	100%
BraA03g33630	AT3G05290	5.0E-148	100%
BraA03g33620	AT3G05300	5.0E-50	59%
BraA03g33610	AT3G05310	0.0E+00	99%
BraA03g33600	AT3G05420	0.0E+00	100%
BraA03g33590	AT3G05470	0.0E+00	100%
BraA03g33580	AT3G05670	0.0E+00	94%

BraA03g33570	AT3G05625	4.0E-138	100%
BraA03g33560	AT3G05610	0.0E+00	94%
BraA03g33550	AT3G05590	5.0E-104	99%
BraA03g33540	AT3G05580	7.0E-180	100%
BraA03g33530	AT3G05560	2.0E-59	99%
BraA03g33520	AT3G05545	2.0E-169	100%
BraA03g33510	AT3G05530	0.0E+00	100%
BraA03g33500	AT3G05520	8.0E-155	95%
BraA03g33490	AT3G05510	0.0E+00	98%
BraA03g33480	AT3G05490	2.0E-50	98%
BraA03g33470	AT3G05770	4.0E-16	72%
BraA03g33460	AT3G05690	2.0E-129	100%
BraA03g33450	AT3G05680	0.0E+00	100%
BraA03g33440	AT3G05675	0.0E+00	98%
BraA03g33430			
BraA03g33420	AT3G05675	1.0E-64	99%
BraA03g33410	AT3G25510	2.0E-31	54%
BraA03g33400			
BraA03g33390			
BraA03g33380	AT3G05220	3.0E-37	36%
BraA03g33370	AT3G05230	1.0E-89	99%
BraA03g33360	AT3G16740	4.0E-125	97%
BraA03g33350	AT3G05280	3.0E-159	48%
BraA03g33340	AT3G05290	2.0E-151	100%
BraA03g33330	AT3G05300	1.0E-48	59%
BraA03g33320	AT3G05310	0.0E+00	99%
BraA03g33310	AT3G05420	0.0E+00	100%
BraA03g33300	AT3G05470	0.0E+00	100%
BraA03g33290	AT3G05490	2.0E-50	98%
BraA03g33280	AT3G05510	0.0E+00	98%
BraA03g33270	AT3G05520	8.0E-131	100%
BraA03g33260	AT3G05530	0.0E+00	100%
BraA03g33250	AT3G05545	5.0E-180	100%
BraA03g33240	AT3G05560	2.0E-59	99%
BraA03g33230	AT3G05580	7.0E-180	100%
BraA03g33220	AT3G05590	5.0E-104	99%
BraA03g33210	AT3G05610	0.0E+00	94%
BraA03g33200	AT3G05625	4.0E-138	100%
BraA03g33190	AT3G05670	0.0E+00	94%
BraA03g33180	AT3G25510	2.0E-22	55%
BraA03g33170	AT3G25510	2.0E-31	54%
BraA03g33160	AT3G05675	1.0E-64	99%
BraA03g33150			
BraA03g33140	AT3G05675	0.0E+00	98%
BraA03g33130	AT3G05680	0.0E+00	98%
BraA03g33120	AT3G05680	0.0E+00	95%
BraA03g33110	AT3G05690	2.0E-129	100%
BraA03g33100	AT3G05770	4.0E-16	72%
BraA03g33090			
BraA03g33080	AT3G05800	8.0E-70	99%
BraA03g33070			
BraA03g33060	AT3G05840	0.0E+00	100%
BraA03g33050	AT3G05858	8.0E-43	98%
BraA03g33040			
BraA03g33030	AT3G05880	3.0E-22	98%
BraA03g33020	AT3G05880	5.0E-20	96%
BraA03g33010	AT3G05900	8.0E-72	74%

BraA03g33000	AT3G05910	0.0E+00	100%
BraA03g32990	AT3G05930	2.0E-98	100%
BraA03g32980	AT3G05936	8.0E-32	80%
BraA03g32970	AT3G05937	3.0E-13	48%
BraA03g32960	AT3G11830	3.0E-81	96%
BraA03g32950	AT3G05960	4.0E-106	99%
BraA03g32940	AT3G04220	3.0E-136	71%
BraA03g32930	AT3G05960	0.0E+00	100%
BraA03g32920	AT3G05970	0.0E+00	98%
BraA03g32910	AT3G24255	1.0E-14	21%
BraA03g32900			
BraA03g32890	AT3G06020	2.0E-81	100%
BraA03g32880	AT3G06035	5.0E-78	88%
BraA03g32870	AT3G06040	5.0E-30	99%
BraA03g32860	AT3G06040	6.0E-27	97%
BraA03g32850			
BraA03g32840	AT3G06070	5.0E-55	99%
BraA03g32830	AT3G06080	5.0E-164	73%
BraA03g32820	AT3G06120	1.0E-106	100%
BraA03g32810	AT3G06130	3.0E-28	23%
BraA03g32800	AT3G06150	0.0E+00	100%
BraA03g32790			
BraA03g32780	AT3G06190	0.0E+00	100%
BraA03g32770	AT3G06240	2.0E-20	63%
BraA03g32760	AT3G06240	5.0E-41	99%
BraA03g32750	AT3G06240	6.0E-151	99%
BraA03g32740	AT3G20015	3.0E-04	46%
BraA03g32730	AT3G10116	2.0E-07	7%
BraA03g32720	AT3G06260	0.0E+00	100%
BraA03g32710	AT3G06270	8.0E-177	100%
BraA03g32700	AT3G06290	0.0E+00	100%
BraA03g32690	AT3G06300	9.0E-154	100%
BraA03g32680	AT3G06330	0.0E+00	96%
BraA03g32670	AT3G06340	0.0E+00	100%
BraA03g32660	AT3G06350	0.0E+00	100%
BraA03g32650	AT3G06410	0.0E+00	100%
BraA03g32640	AT3G06420	5.0E-63	99%
BraA03g32630	AT3G06450	0.0E+00	100%
BraA03g32620	AT3G06470	3.0E-116	96%
BraA03g32610	AT3G06480	0.0E+00	84%
BraA03g32600	AT3G06483	0.0E+00	100%
BraA03g32590	AT3G06490	1.0E-100	100%
BraA03g32580	AT3G06530	0.0E+00	100%
BraA03g32570	AT3G06580	0.0E+00	82%
BraA03g32560	AT3G06620	9.0E-89	99%
BraA03g32550	AT3G06640	7.0E-67	97%
BraA03g32540			
BraA03g32530	AT3G06620	2.0E-74	82%
BraA03g32520	AT3G06620	8.0E-09	36%
BraA03g32510			
BraA03g32500			
BraA03g32490			
BraA03g32480			
BraA03g32470	AT3G06710	1.0E-37	23%
BraA03g32460	AT3G06660	2.0E-103	97%
BraA03g32450	AT3G06670	0.0E+00	100%
BraA03g32440			

BraA03g32430	AT3G06760	1.0E-67	99%
BraA03g32420	AT3G06770	0.0E+00	84%
BraA03g32410			
BraA03g32400			
BraA03g32390	AT3G06778	1.0E-104	100%
BraA03g32380	AT3G06780	2.0E-20	100%
BraA03g32370	AT3G06790	2.0E-101	91%
BraA03g32360	AT3G06830	0.0E+00	100%
BraA03g32350	AT3G06850	0.0E+00	100%
BraA03g32340	AT3G06860	0.0E+00	100%
BraA03g32330	AT3G06868	3.0E-92	100%
BraA03g32320	AT3G06880	0.0E+00	99%
BraA03g32310	AT3G06890	6.0E-30	99%
BraA03g32300	AT3G06920	0.0E+00	97%
BraA03g32290	AT3G06950	2.0E-134	100%
BraA03g32280	AT3G06970	2.0E-46	87%
BraA03g32270			
BraA03g32260	AT3G07020	0.0E+00	100%
BraA03g32250	AT3G07030	9.0E-68	43%
BraA03g32240			
BraA03g32230	AT3G07050	0.0E+00	97%
BraA03g32220	AT3G07070	0.0E+00	94%
BraA03g32210	AT3G07080	0.0E+00	99%
BraA03g32200	AT3G07100	0.0E+00	100%
BraA03g32190	AT3G07120	2.0E-119	100%
BraA03g32180			
BraA03g32170	AT3G07160	2.0E-26	66%
BraA03g32160			
BraA03g32150			
BraA03g32140			
BraA03g32130			
BraA03g32120	AT3G07160	0.0E+00	100%
BraA03g32110	AT3G07170	5.0E-88	99%
BraA03g32100	AT3G07195	1.0E-57	100%
BraA03g32090	AT3G07250	6.0E-87	90%
BraA03g32080			
BraA03g32070			
BraA03g32060	AT3G07320	0.0E+00	97%
BraA03g32050	AT3G07330	0.0E+00	100%
BraA03g32040	AT3G07360	0.0E+00	100%
BraA03g32030			
BraA03g32020	AT3G07390	8.0E-82	95%
BraA03g32010	AT3G07420	0.0E+00	100%
BraA03g32000	AT3G07425	2.0E-31	93%
BraA03g31990	AT3G07450	6.0E-40	99%
BraA03g31980	AT3G07470	4.0E-62	97%
BraA03g31970	AT3G07490	6.0E-72	99%
BraA03g31960	AT3G07510	3.0E-79	99%
BraA03g31950	AT3G02880	1.0E-18	82%
BraA03g31940	AT3G25500	5.0E-12	27%
BraA03g31930	AT3G07540	0.0E+00	100%
BraA03g31920	AT3G07560	8.0E-69	42%
BraA03g31910	AT3G07565	3.0E-127	100%
BraA03g31900	AT3G07570	2.0E-82	82%
BraA03g31890	AT3G07590	3.0E-49	58%
BraA03g31880	AT3G07600	1.0E-34	94%
BraA03g31870	AT3G07630	6.0E-146	99%

BraA03g31860	AT3G07630	1.0E-14	97%
BraA03g31850	AT3G07640	2.0E-25	100%
BraA03g31840	AT3G07330	0.0E+00	100%
BraA03g31830	AT3G07360	0.0E+00	100%
BraA03g31820			
BraA03g31810	AT3G07390	8.0E-82	95%
BraA03g31800	AT3G07420	0.0E+00	100%
BraA03g31790	AT3G07425	2.0E-31	93%
BraA03g31780	AT3G07450	6.0E-40	99%
BraA03g31770	AT3G07470	1.0E-67	99%
BraA03g31760	AT3G07490	6.0E-72	99%
BraA03g31750	AT3G07510	3.0E-79	99%
BraA03g31740	AT3G02880	2.0E-18	78%
BraA03g31730	AT3G25500	1.0E-11	40%
BraA03g31720	AT3G07540	0.0E+00	100%
BraA03g31710	AT3G07560	2.0E-68	42%
BraA03g31700	AT3G07565	2.0E-127	100%
BraA03g31690	AT3G07570	2.0E-82	82%
BraA03g31680	AT3G07590	3.0E-49	58%
BraA03g31670	AT3G07600	2.0E-49	99%
BraA03g31660	AT3G07630	0.0E+00	100%
BraA03g31650	AT3G07640	1.0E-112	100%
BraA03g31640	AT3G07650	8.0E-173	100%
BraA03g31630	AT3G07680	8.0E-99	79%
BraA03g31620	AT3G07760	2.0E-66	99%
BraA03g31610	AT3G07800	8.0E-109	95%
BraA03g31600	AT3G07810	0.0E+00	100%
BraA03g31590	AT3G07820	0.0E+00	98%
BraA03g31580	AT3G07840	4.0E-163	99%
BraA03g31570	AT3G14040	2.0E-165	94%
BraA03g31560	AT3G07860	6.0E-79	94%
BraA03g31550			
BraA03g31540	AT3G07870	0.0E+00	100%
BraA03g31530	AT3G07880	5.0E-115	100%
BraA03g31520	AT3G07890	0.0E+00	100%
BraA03g31510			
BraA03g31500	AT3G07910	1.0E-19	99%
BraA03g31490	AT3G07940	0.0E+00	100%
BraA03g31480	AT3G07960	0.0E+00	100%
BraA03g31470	AT3G07970	3.0E-48	100%
BraA03g31460	AT3G07970	1.0E-120	96%
BraA03g31450	AT3G13530	3.0E-96	98%
BraA03g31440	AT3G07980	0.0E+00	100%
BraA03g31430	AT3G08010	0.0E+00	100%
BraA03g31420	AT3G08040	0.0E+00	99%
BraA03g31410	AT3G10040	5.0E-07	31%
BraA03g31400	AT3G08040	6.0E-127	74%
BraA03g31390	AT3G08530	0.0E+00	93%
BraA03g31380	AT3G11120	1.0E-05	96%
BraA03g31370	AT3G08505	2.0E-147	100%
BraA03g31360	AT3G08500	1.0E-55	95%
BraA03g31350	AT3G08500	1.0E-68	99%
BraA03g31340	AT3G11130	0.0E+00	100%
BraA03g31330	AT3G10195	2.0E-25	97%
BraA03g31320	AT3G10180	0.0E+00	98%
BraA03g31310	AT3G10140	2.0E-178	70%
BraA03g31300	AT3G10060	2.0E-20	68%

BraA03g31290	AT3G10050	0.0E+00	100%
BraA03g31280	AT3G10040	4.0E-157	100%
BraA03g31270	AT3G10020	2.0E-66	97%
BraA03g31260	AT3G09980	2.0E-75	93%
BraA03g31250	AT3G09940	1.0E-148	95%
BraA03g31240	AT3G09925	1.0E-81	99%
BraA03g31230	AT3G09890	3.0E-88	100%
BraA03g31220	AT3G09880	0.0E+00	100%
BraA03g31210	AT3G09840	0.0E+00	100%
BraA03g31200	AT3G09800	4.0E-98	96%
BraA03g31190	AT3G09760	0.0E+00	99%
BraA03g31180	AT3G09735	2.0E-13	99%
BraA03g31170	AT3G09710	2.0E-134	99%
BraA03g31160	AT3G09680	2.0E-75	99%
BraA03g31150	AT3G09670	0.0E+00	86%
BraA03g31140	AT3G09630	0.0E+00	100%
BraA03g31130	AT3G09590	2.0E-78	96%
BraA03g31120	AT3G09550	0.0E+00	100%
BraA03g31110	AT3G09540	7.0E-18	43%
BraA03g31100	AT3G09530	0.0E+00	100%
BraA03g31090	AT3G09500	4.0E-62	99%
BraA03g31080	AT3G09410	0.0E+00	100%
BraA03g31070	AT3G09400	0.0E+00	100%
BraA03g31060	AT3G09390	4.0E-22	65%
BraA03g31050	AT3G09370	8.0E-140	53%
BraA03g31040	AT3G09360	0.0E+00	95%
BraA03g31030	AT3G09350	2.0E-24	89%
BraA03g31020	AT3G09350	2.0E-20	94%
BraA03g31010	AT3G09360	0.0E+00	100%
BraA03g31000	AT3G09350	4.0E-175	100%
BraA03g30990	AT3G09330	0.0E+00	99%
BraA03g30980	AT3G09320	6.0E-147	99%
BraA03g30970	AT3G09300	0.0E+00	99%
BraA03g30960	AT3G09290	3.0E-66	99%
BraA03g30950	AT3G09280	8.0E-46	99%
BraA03g30940	AT3G09260	0.0E+00	100%
BraA03g30930	AT3G09250	3.0E-34	50%
BraA03g30920	AT3G09240	0.0E+00	96%
BraA03g30910	AT3G09230	2.0E-139	99%
BraA03g30900	AT3G09200	2.0E-155	85%
BraA03g30890	AT3G09150	8.0E-132	79%
BraA03g30880	AT3G09070	0.0E+00	100%
BraA03g30870	AT3G09060	0.0E+00	99%
BraA03g30860	AT3G04250	4.0E-31	94%
BraA03g30850	AT3G20950	1.0E-96	90%
BraA03g30840	AT3G22940	6.0E-87	88%
BraA03g30830	AT3G09040	0.0E+00	100%
BraA03g30820	AT3G20140	6.0E-28	57%
BraA03g30810	AT3G09180	0.0E+00	100%
BraA03g30800	AT3G09190	7.0E-127	99%
BraA03g30790	AT3G09010	2.0E-158	100%
BraA03g30780	AT3G08960	0.0E+00	100%
BraA03g30770	AT3G08950	8.0E-134	100%
BraA03g30760	AT3G08930	0.0E+00	100%
BraA03g30750	AT3G08910	2.0E-166	100%
BraA03g30740	AT3G08900	2.0E-111	97%
BraA03g30730	AT3G08900	5.0E-93	98%

BraA03g30720	AT3G08890	1.0E-80	99%
BraA03g30710	AT3G08880	1.0E-30	98%
BraA03g30700	AT3G08860	0.0E+00	100%
BraA03g30690	AT3G08850	0.0E+00	100%
BraA03g30680	AT3G08840	0.0E+00	55%
BraA03g30670	AT3G08770	1.0E-34	86%
BraA03g30660	AT3G08710	1.0E-70	99%
BraA03g30650	AT3G08690	2.0E-84	99%
BraA03g30640	AT3G08670	0.0E+00	100%
BraA03g30630	AT3G08660	0.0E+00	96%
BraA03g30620	AT3G08650	0.0E+00	100%
BraA03g30610	AT3G08610	5.0E-33	98%
BraA03g30600	AT3G08600	2.0E-107	89%
BraA03g30590	AT3G10310	5.0E-27	91%
BraA03g30580			
BraA03g30570	AT3G06545	6.0E-47	88%
BraA03g30560	AT3G10330	0.0E+00	100%
BraA03g30550	AT3G10360	0.0E+00	100%
BraA03g30540	AT3G10380	0.0E+00	95%
BraA03g30530	AT3G10390	0.0E+00	100%
BraA03g30520	AT3G10405	4.0E-83	86%
BraA03g30510	AT3G10410	0.0E+00	100%
BraA03g30500	AT3G08750	4.0E-76	98%
BraA03g30490	AT3G10430	2.0E-77	70%
BraA03g30480	AT3G08750	1.0E-75	96%
BraA03g30470	AT3G08750	1.0E-15	62%
BraA03g30460	AT3G08750	9.0E-34	99%
BraA03g30450	AT3G10480	7.0E-180	100%
BraA03g30440	AT3G10480	1.0E-76	69%
BraA03g30430	AT3G10525	6.0E-35	99%
BraA03g30420			
BraA03g30410	AT3G10572	4.0E-143	100%
BraA03g30400			
BraA03g30390	AT3G10640	1.0E-122	100%
BraA03g30380	AT3G10650	0.0E+00	100%
BraA03g30370	AT3G10660	4.0E-178	67%
BraA03g30360	AT3G10670	4.0E-162	100%
BraA03g30350	AT3G10690	0.0E+00	100%
BraA03g30340	AT3G10710	0.0E+00	100%
BraA03g30330	AT3G10740	0.0E+00	100%
BraA03g30320	AT3G10760	1.0E-31	85%
BraA03g30310			
BraA03g30300			
BraA03g30290	AT3G10760	2.0E-23	70%
BraA03g30280	AT3G10770	4.0E-139	99%
BraA03g30270	AT3G10780	5.0E-62	99%
BraA03g30260	AT3G11060	5.0E-47	79%
BraA03g30250	AT3G10815	8.0E-78	99%
BraA03g30240	AT3G10820	2.0E-59	55%
BraA03g30230	AT3G10890	0.0E+00	98%
BraA03g30220			
BraA03g30210	AT3G10986	3.0E-56	99%
BraA03g30200			
BraA03g30190	AT3G10986	2.0E-34	97%
BraA03g30180			
BraA03g30170	AT3G11020	4.0E-74	84%
BraA03g30160	AT3G18990	4.0E-05	21%

BraA03g30150	AT3G18960	7.0E-04	30%
BraA03g30140	AT3G11080	1.0E-128	97%
BraA03g30130	AT3G11070	0.0E+00	100%
BraA03g30120	AT3G11110	2.0E-58	98%
BraA03g30110			
BraA03g30100	AT3G11130	0.0E+00	97%
BraA03g30090			
BraA03g30080	AT3G11170	0.0E+00	100%
BraA03g30070	AT3G11200	8.0E-115	99%
BraA03g30060			
BraA03g30050			
BraA03g30040			
BraA03g30030	AT3G09840	7.0E-06	61%
BraA03g30020	AT3G13590	1.0E-166	97%
BraA03g30010	AT3G11230	1.0E-69	99%
BraA03g30000	AT3G11320	6.0E-173	100%
BraA03g29990	AT3G11330	0.0E+00	100%
BraA03g29980	AT3G11380	8.0E-164	68%
BraA03g29970			
BraA03g29960	AT3G11400	2.0E-163	100%
BraA03g29950	AT3G11420	0.0E+00	100%
BraA03g29940	AT3G11450	0.0E+00	100%
BraA03g29930	AT3G11480	3.0E-178	100%
BraA03g29920			
BraA03g29910	AT3G11530	3.0E-59	83%
BraA03g29900	AT3G11510	5.0E-76	92%
BraA03g29890	AT3G11060	3.0E-07	42%
BraA03g29880	AT3G11540	0.0E+00	100%
BraA03g29870	AT3G11550	3.0E-84	100%
BraA03g29860	AT3G11570	0.0E+00	100%
BraA03g29850	AT3G11580	3.0E-80	82%
BraA03g29840	AT3G11590	1.0E-132	99%
BraA03g29830	AT3G11600	3.0E-45	97%
BraA03g29820	AT3G11620	2.0E-103	94%
BraA03g29810	AT3G11630	1.0E-121	100%
BraA03g29800	AT3G11710	0.0E+00	100%
BraA03g29790	AT3G11720	0.0E+00	99%
BraA03g29780	AT3G11730	2.0E-116	100%
BraA03g29770	AT3G11740	5.0E-104	99%
BraA03g29760	AT3G11780	2.0E-67	97%
BraA03g29750	AT3G11800	5.0E-126	100%
BraA03g29740	AT3G11820	6.0E-147	99%
BraA03g29730	AT3G11830	0.0E+00	96%
BraA03g29720	AT3G11840	0.0E+00	97%
BraA03g29710	AT3G11850	4.0E-21	74%
BraA03g29700	AT3G11900	0.0E+00	100%
BraA03g29690	AT3G11910	0.0E+00	100%
BraA03g29680			
BraA03g29670	AT3G11930	5.0E-93	99%
BraA03g29660	AT3G11940	2.0E-115	100%
BraA03g29650	AT3G11945	9.0E-124	96%
BraA03g29640	AT3G11950	0.0E+00	89%
BraA03g29630	AT3G11960	0.0E+00	100%
BraA03g29620	AT3G12000	2.0E-144	94%
BraA03g29610	AT3G25050	1.0E-72	97%
BraA03g29600	AT3G25050	5.0E-97	96%
BraA03g29590	AT3G12012	5.0E-28	82%

BraA03g29580	AT3G12010	0.0E+00	99%
BraA03g29570	AT3G12130	7.0E-109	99%
BraA03g29560	AT3G12140	5.0E-158	100%
BraA03g29550	AT3G12150	0.0E+00	100%
BraA03g29540	AT3G12160	6.0E-127	100%
BraA03g29530	AT3G12240	3.0E-170	94%
BraA03g29520	AT3G12240	1.0E-167	97%
BraA03g29510	AT3G12240	8.0E-121	100%
BraA03g29500			
BraA03g29490	AT3G12250	1.0E-163	87%
BraA03g29480	AT3G12260	2.0E-74	99%
BraA03g29470	AT3G12270	3.0E-151	96%
BraA03g29460	AT3G12280	0.0E+00	100%
BraA03g29450	AT3G12290	7.0E-158	100%
BraA03g29440	AT3G12300	4.0E-108	99%
BraA03g29430	AT3G12320	2.0E-101	100%
BraA03g29420	AT3G12390	2.0E-77	100%
BraA03g29410	AT3G12400	3.0E-175	100%
BraA03g29400	AT3G12410	2.0E-77	99%
BraA03g29390	AT3G12480	9.0E-114	96%
BraA03g29380	AT3G12500	3.0E-37	66%
BraA03g29370	AT3G12500	9.0E-65	99%
BraA03g29360	AT3G12500	1.0E-115	100%
BraA03g29350	AT3G12520	0.0E+00	100%
BraA03g29340	AT3G12530	3.0E-110	100%
BraA03g29330	AT3G12540	0.0E+00	88%
BraA03g29320	AT3G12580	0.0E+00	100%
BraA03g29310	AT3G12590	0.0E+00	100%
BraA03g29300	AT3G12600	1.0E-80	98%
BraA03g29290	AT3G12610	0.0E+00	100%
BraA03g29280	AT3G12630	9.0E-61	99%
BraA03g29270	AT3G12650	1.0E-93	100%
BraA03g29260	AT3G12640	0.0E+00	100%
BraA03g29250	AT3G12660	2.0E-63	94%
BraA03g29240	AT3G12670	2.0E-80	28%
BraA03g29230	AT3G12700	0.0E+00	100%
BraA03g29220	AT3G12740	8.0E-168	51%
BraA03g29210	AT3G12775	2.0E-92	79%
BraA03g29200	AT3G12780	0.0E+00	98%
BraA03g29190	AT3G12800	1.0E-145	100%
BraA03g29180	AT3G12820	2.0E-27	72%
BraA03g29170	AT3G12830	2.0E-60	99%
BraA03g29160	AT3G12860	0.0E+00	87%
BraA03g29150	AT3G12890	8.0E-112	100%
BraA03g29140			
BraA03g29130	AT3G12900	0.0E+00	100%
BraA03g29120	AT3G12910	3.0E-27	83%
BraA03g29110			
BraA03g29100			
BraA03g29090	AT3G12910	1.0E-86	98%
BraA03g29080			
BraA03g29070	AT3G12920	9.0E-147	100%
BraA03g29060			
BraA03g29050			
BraA03g29040	AT3G12970	1.0E-103	94%
BraA03g29030			
BraA03g29020	AT3G13470	8.0E-05	10%

BraA03g29010	AT3G12977	1.0E-133	100%
BraA03g29000	AT3G12980	0.0E+00	100%
BraA03g28990	AT3G13000	0.0E+00	94%
BraA03g28980	AT3G13040	3.0E-11	23%
BraA03g28970	AT3G13040	0.0E+00	99%
BraA03g28960			
BraA03g28950	AT3G13050	0.0E+00	67%
BraA03g28940	AT3G13062	0.0E+00	100%
BraA03g28930	AT3G13070	0.0E+00	100%
BraA03g28920	AT3G13080	0.0E+00	100%
BraA03g28910	AT3G13120	7.0E-83	99%
BraA03g28900	AT3G13080	0.0E+00	100%
BraA03g28890			
BraA03g28880	AT3G13130	3.0E-57	100%
BraA03g28870	AT3G13160	0.0E+00	100%
BraA03g28860	AT3G13200	2.0E-38	94%
BraA03g28850	AT3G13227	9.0E-21	62%
BraA03g28840	AT3G13229	3.0E-123	79%
BraA03g28830	AT3G13235	0.0E+00	100%
BraA03g28820	AT3G13310	4.0E-62	99%
BraA03g28810			
BraA03g28800	AT3G13320	0.0E+00	95%
BraA03g28790	AT3G13330	0.0E+00	99%
BraA03g28780	AT3G13330	0.0E+00	99%
BraA03g28770	AT3G13340	0.0E+00	100%
BraA03g28760	AT3G13360	1.0E-141	94%
BraA03g28750	AT3G13390	0.0E+00	100%
BraA03g28740	AT3G13400	0.0E+00	100%
BraA03g28730			
BraA03g28720	AT3G13410	3.0E-164	100%
BraA03g28710			
BraA03g28700	AT3G13433	7.0E-04	81%
BraA03g28690			
BraA03g28680	AT3G13433	5.0E-06	81%
BraA03g28670			
BraA03g28660	AT3G13460	0.0E+00	100%
BraA03g28650	AT3G13470	0.0E+00	100%
BraA03g28640			
BraA03g28630	AT3G13480	4.0E-33	99%
BraA03g28620	AT3G13490	3.0E-82	88%
BraA03g28610	AT3G13530	0.0E+00	100%
BraA03g28600	AT3G09510	6.0E-08	84%
BraA03g28590	AT3G13600	0.0E+00	99%
BraA03g28580	AT3G13610	0.0E+00	100%
BraA03g28570	AT3G13620	0.0E+00	100%
BraA03g28560	AT3G13640	0.0E+00	100%
BraA03g28550	AT3G13650	9.0E-91	99%
BraA03g28540			
BraA03g28530	AT3G13670	0.0E+00	98%
BraA03g28520	AT3G13672	2.0E-100	85%
BraA03g28510	AT3G13690	0.0E+00	100%
BraA03g28500			
BraA03g28490	AT3G13760	9.0E-176	92%
BraA03g28480	AT3G13760	2.0E-25	59%
BraA03g28470	AT3G13772	0.0E+00	100%
BraA03g28460	AT3G13800	0.0E+00	100%
BraA03g28450			

BraA03g28440	AT3G13810	2.0E-164	100%
BraA03g28430	AT3G13860	0.0E+00	100%
BraA03g28420			
BraA03g28410	AT3G13870	2.0E-150	100%
BraA03g28400	AT3G13870	0.0E+00	97%
BraA03g28390	AT3G13882	3.0E-35	89%
BraA03g28380	AT3G22820	1.0E-06	71%
BraA03g28370	AT3G13910	7.0E-35	98%
BraA03g28360	AT3G13920	0.0E+00	100%
BraA03g28350	AT3G13930	0.0E+00	99%
BraA03g28340	AT3G13960	2.0E-134	98%
BraA03g28330			
BraA03g28320	AT3G14010	3.0E-33	31%
BraA03g28310	AT3G14010	2.0E-167	96%
BraA03g28300	AT3G14080	3.0E-69	99%
BraA03g28290	AT3G14110	1.0E-143	100%
BraA03g28280	AT3G14150	0.0E+00	100%
BraA03g28270	AT3G11090	2.0E-25	95%
BraA03g28260	AT3G14172	0.0E+00	100%
BraA03g28250	AT3G14190	2.0E-84	99%
BraA03g28240			
BraA03g28230	AT3G14200	8.0E-103	100%
BraA03g28220			
BraA03g28210	AT3G13870	0.0E+00	100%
BraA03g28200	AT3G13882	2.0E-68	99%
BraA03g28190			
BraA03g28180	AT3G13882	4.0E-68	99%
BraA03g28170	AT3G22820	1.0E-06	71%
BraA03g28160	AT3G13910	7.0E-35	98%
BraA03g28150	AT3G13920	0.0E+00	100%
BraA03g28140	AT3G13930	0.0E+00	99%
BraA03g28130	AT3G13960	3.0E-135	100%
BraA03g28120			
BraA03g28110	AT3G14010	4.0E-33	32%
BraA03g28100	AT3G14010	2.0E-168	100%
BraA03g28090	AT3G14080	3.0E-69	99%
BraA03g28080	AT3G14110	1.0E-139	100%
BraA03g28070	AT3G14150	0.0E+00	100%
BraA03g28060	AT3G11090	2.0E-25	95%
BraA03g28050	AT3G14172	0.0E+00	100%
BraA03g28040	AT3G14190	2.0E-84	99%
BraA03g28030			
BraA03g28020	AT3G14200	1.0E-80	92%
BraA03g28010	AT3G13430	2.0E-25	96%
BraA03g28000	AT3G14230	5.0E-120	100%
BraA03g27990	AT3G14240	0.0E+00	97%
BraA03g27980	AT3G14250	3.0E-110	93%
BraA03g27970	AT3G14260	7.0E-100	98%
BraA03g27960	AT3G11100	2.0E-33	96%
BraA03g27950	AT3G14270	0.0E+00	100%
BraA03g27940	AT3G14300	0.0E+00	90%
BraA03g27930	AT3G14310	0.0E+00	100%
BraA03g27920			
BraA03g27910	AT3G14395	7.0E-26	99%
BraA03g27900	AT3G14400	5.0E-24	80%
BraA03g27890			
BraA03g27880	AT3G06040	5.0E-09	34%

BraA03g27870	AT3G19120	2.0E-23	58%
BraA03g27860	AT3G14415	0.0E+00	100%
BraA03g27850	AT3G14420	0.0E+00	100%
BraA03g27840	AT3G14440	0.0E+00	100%
BraA03g27830			
BraA03g27820	AT3G14450	6.0E-162	100%
BraA03g27810	AT3G14490	0.0E+00	100%
BraA03g27800	AT3G14550	1.0E-158	99%
BraA03g27790	AT3G14490	0.0E+00	100%
BraA03g27780	AT3G14595	7.0E-41	99%
BraA03g27770	AT3G14600	2.0E-96	71%
BraA03g27760	AT3G14630	0.0E+00	100%
BraA03g27750	AT3G14690	0.0E+00	100%
BraA03g27740	AT3G14680	7.0E-137	100%
BraA03g27730	AT3G14690	2.0E-120	99%
BraA03g27720	AT3G14680	2.0E-73	99%
BraA03g27710	AT3G14690	5.0E-39	53%
BraA03g27700	AT3G14690	0.0E+00	97%
BraA03g27690	AT3G14810	0.0E+00	100%
BraA03g27680			
BraA03g27670	AT3G14840	2.0E-32	14%
BraA03g27660	AT3G08870	1.0E-07	38%
BraA03g27650	AT3G24240	3.0E-36	39%
BraA03g27640	AT3G05140	1.0E-07	39%
BraA03g27630	AT3G24240	3.0E-36	37%
BraA03g27620	AT3G14850	2.0E-133	78%
BraA03g27610	AT3G14860	0.0E+00	100%
BraA03g27600	AT3G14870	0.0E+00	100%
BraA03g27590	AT3G14890	0.0E+00	99%
BraA03g27580	AT3G14920	0.0E+00	99%
BraA03g27570	AT3G14930	4.0E-50	96%
BraA03g27560	AT3G14940	0.0E+00	100%
BraA03g27550	AT3G14960	0.0E+00	99%
BraA03g27540	AT3G14990	0.0E+00	100%
BraA03g27530	AT3G15000	3.0E-145	100%
BraA03g27520	AT3G15030	2.0E-155	99%
BraA03g27510			
BraA03g27500	AT3G15040	3.0E-77	100%
BraA03g27490			
BraA03g27480			
BraA03g27470	AT3G15060	7.0E-118	100%
BraA03g27460	AT3G15070	2.0E-114	99%
BraA03g27450			
BraA03g27440	AT3G15090	0.0E+00	100%
BraA03g27430	AT3G15120	0.0E+00	100%
BraA03g27420	AT3G15130	0.0E+00	100%
BraA03g27410	AT3G15160	0.0E+00	100%
BraA03g27400			
BraA03g27390	AT3G15170	1.0E-122	95%
BraA03g27380	AT3G15190	4.0E-75	100%
BraA03g27370	AT3G15210	5.0E-80	100%
BraA03g27360			
BraA03g27350			
BraA03g27340	AT3G15260	1.0E-157	100%
BraA03g27330	AT3G15270	7.0E-31	47%
BraA03g27320	AT3G15340	0.0E+00	97%
BraA03g27310	AT3G15355	3.0E-176	98%

BraA03g27300	AT3G15360	2.0E-60	85%
BraA03g27290			
BraA03g27280			
BraA03g27270	AT3G15370	5.0E-133	99%
BraA03g27260	AT3G15380	0.0E+00	80%
BraA03g27250	AT3G15460	2.0E-168	99%
BraA03g27240	AT3G15500	1.0E-41	65%
BraA03g27230	AT3G15510	3.0E-153	100%
BraA03g27220	AT3G15540	1.0E-73	85%
BraA03g27210	AT3G15550	2.0E-21	65%
BraA03g27200	AT3G15570	0.0E+00	100%
BraA03g27190	AT3G15630	2.0E-35	96%
BraA03g27180	AT3G15640	8.0E-30	63%
BraA03g27170	AT3G15640	1.0E-27	63%
BraA03g27160			
BraA03g27150			
BraA03g27140	AT3G15640	7.0E-28	72%
BraA03g27130			
BraA03g27120	AT3G15650	2.0E-135	99%
BraA03g27110	AT3G15670	7.0E-84	100%
BraA03g27100			
BraA03g27090			
BraA03g27080	AT3G06910	6.0E-07	22%
BraA03g27070	AT3G24255	7.0E-55	89%
BraA03g27060	AT3G15680	2.0E-74	99%
BraA03g27050	AT3G15720	0.0E+00	100%
BraA03g27040	AT3G15730	1.0E-43	65%
BraA03g27030	AT3G15810	1.0E-112	100%
BraA03g27020	AT3G15820	8.0E-132	99%
BraA03g27010	AT3G15840	4.0E-148	100%
BraA03g27000	AT3G15850	2.0E-180	99%
BraA03g26990	AT3G15870	3.0E-152	100%
BraA03g26980	AT3G15870	4.0E-159	99%
BraA03g26970	AT3G15870	2.0E-158	99%
BraA03g26960	AT3G15880	0.0E+00	98%
BraA03g26950	AT3G15890	3.0E-143	98%
BraA03g26940			
BraA03g26930	AT3G15970	1.0E-31	29%
BraA03g26920	AT3G15980	0.0E+00	99%
BraA03g26910	AT3G15990	0.0E+00	100%
BraA03g26900	AT3G16000	0.0E+00	100%
BraA03g26890	AT3G16010	0.0E+00	100%
BraA03g26880	AT3G11780	8.0E-05	63%
BraA03g26870	AT3G16010	7.0E-53	98%
BraA03g26860	AT3G11780	2.0E-07	79%
BraA03g26850			
BraA03g26840			
BraA03g26830	AT3G11780	1.0E-09	68%
BraA03g26820	AT3G16060	9.0E-23	95%
BraA03g26810			
BraA03g26800			
BraA03g26790	AT3G16210	8.0E-15	97%
BraA03g26780	AT3G16080	5.0E-48	99%
BraA03g26770	AT3G16210	1.0E-147	100%
BraA03g26760	AT3G16230	2.0E-155	73%
BraA03g26750	AT3G16240	4.0E-131	100%
BraA03g26740	AT3G16300	7.0E-71	98%

BraA03g26730	AT3G16330	4.0E-96	99%
BraA03g26720	AT3G16350	2.0E-17	53%
BraA03g26710			
BraA03g26700	AT3G16350	4.0E-120	100%
BraA03g26690	AT3G16360	3.0E-61	89%
BraA03g26680	AT3G12000	3.0E-29	97%
BraA03g26670	AT3G12000	7.0E-21	36%
BraA03g26660			
BraA03g26650			
BraA03g26640	AT3G16430	5.0E-138	100%
BraA03g26630	AT3G16430	3.0E-91	86%
BraA03g26620	AT3G16490	7.0E-163	100%
BraA03g26610			
BraA03g26600	AT3G16500	8.0E-109	100%
BraA03g26590	AT3G16510	4.0E-88	100%
BraA03g26580			
BraA03g26570	AT3G16570	2.0E-53	99%
BraA03g26560	AT3G16630	5.0E-20	88%
BraA03g26550			
BraA03g26540			
BraA03g26530	AT3G16640	1.0E-86	99%
BraA03g26520	AT3G16690	3.0E-110	100%
BraA03g26510	AT3G16720	7.0E-101	99%
BraA03g26500	AT3G16760	6.0E-14	42%
BraA03g26490	AT3G16770	9.0E-35	49%
BraA03g26480	AT3G16857	2.0E-70	98%
BraA03g26470	AT3G16850	0.0E+00	100%
BraA03g26460	AT3G16857	0.0E+00	94%
BraA03g26450			
BraA03g26440			
BraA03g26430			
BraA03g26420	AT3G16870	2.0E-61	99%
BraA03g26410	AT3G16950	0.0E+00	91%
BraA03g26400			
BraA03g26390	AT3G16990	1.0E-108	98%
BraA03g26380	AT3G17010	3.0E-55	96%
BraA03g26370	AT3G17010	2.0E-89	97%
BraA03g26360	AT3G17020	1.0E-84	99%
BraA03g26350			
BraA03g26340	AT3G17060	0.0E+00	99%
BraA03g26330	AT3G16660	4.0E-24	99%
BraA03g26320	AT3G17120	3.0E-88	97%
BraA03g26310			
BraA03g26300			
BraA03g26290	AT3G17130	3.0E-76	99%
BraA03g26280			
BraA03g26270	AT3G17620	3.0E-46	90%
BraA03g26260	AT3G17230	8.0E-40	98%
BraA03g26250			
BraA03g26240	AT3G17300	3.0E-47	99%
BraA03g26230	AT3G17365	4.0E-118	100%
BraA03g26220	AT3G17380	3.0E-162	100%
BraA03g26210	AT3G17390	0.0E+00	100%
BraA03g26200			
BraA03g26190			
BraA03g26180	AT3G17410	0.0E+00	97%
BraA03g26170	AT3G17440	4.0E-147	99%

BraA03g26160	AT3G17470	0.0E+00	100%
BraA03g26150	AT3G17510	0.0E+00	100%
BraA03g26140			
BraA03g26130	AT3G17520	4.0E-86	100%
BraA03g26120	AT3G17570	5.0E-79	99%
BraA03g26110	AT3G05858	3.0E-09	95%
BraA03g26100	AT3G17580	1.0E-34	88%
BraA03g26090	AT3G18550	2.0E-158	92%
BraA03g26080	AT3G18524	0.0E+00	100%
BraA03g26070	AT3G18520	3.0E-128	65%
BraA03g26060			
BraA03g26050	AT3G18490	0.0E+00	100%
BraA03g26040	AT3G18410	1.0E-57	99%
BraA03g26030	AT3G18400	3.0E-143	100%
BraA03g26020	AT3G18390	0.0E+00	97%
BraA03g26010	AT3G18300	6.0E-89	99%
BraA03g26000	AT3G18295	1.0E-41	99%
BraA03g25990	AT3G18280	2.0E-41	99%
BraA03g25980	AT3G18260	4.0E-105	100%
BraA03g25970	AT3G18210	0.0E+00	98%
BraA03g25960	AT3G18200	0.0E+00	100%
BraA03g25950	AT3G18160	3.0E-174	99%
BraA03g25940	AT3G18050	5.0E-149	100%
BraA03g25930	AT3G18035	5.0E-42	79%
BraA03g25920	AT3G18030	7.0E-116	98%
BraA03g25910	AT3G18010	7.0E-154	100%
BraA03g25900	AT3G17980	2.0E-88	96%
BraA03g25890	AT3G17970	0.0E+00	95%
BraA03g25880	AT3G17950	8.0E-78	99%
BraA03g25870	AT3G17010	7.0E-11	56%
BraA03g25860	AT3G17560	2.0E-61	98%
BraA03g25850	AT3G17900	0.0E+00	100%
BraA03g25840			
BraA03g25830	AT3G17560	8.0E-58	85%
BraA03g25820	AT3G17820	0.0E+00	99%
BraA03g25810	AT3G25270	2.0E-26	98%
BraA03g25800			
BraA03g25790	AT3G17810	9.0E-45	99%
BraA03g25780	AT3G17800	0.0E+00	100%
BraA03g25770	AT3G17780	1.0E-57	99%
BraA03g25760	AT3G17710	7.0E-130	98%
BraA03g25750	AT3G17710	6.0E-135	98%
BraA03g25740			
BraA03g25730	AT3G17700	0.0E+00	99%
BraA03g25720	AT3G17650	3.0E-06	23%
BraA03g25710	AT3G17650	7.0E-47	99%
BraA03g25700	AT3G17700	0.0E+00	99%
BraA03g25690			
BraA03g25680	AT3G17650	1.0E-134	63%
BraA03g25670	AT3G17640	2.0E-178	100%
BraA03g25660	AT3G17630	0.0E+00	100%
BraA03g25650	AT3G17630	2.0E-39	96%
BraA03g25640	AT3G17980	5.0E-97	99%
BraA03g25630	AT3G17970	0.0E+00	95%
BraA03g25620	AT3G17950	4.0E-79	99%
BraA03g25610	AT3G17010	7.0E-11	56%
BraA03g25600	AT3G17560	6.0E-61	98%

BraA03g25590	AT3G17900	0.0E+00	100%
BraA03g25580	AT3G18210	0.0E+00	98%
BraA03g25570	AT3G18200	0.0E+00	100%
BraA03g25560	AT3G18160	3.0E-174	99%
BraA03g25550	AT3G18050	4.0E-149	99%
BraA03g25540	AT3G18035	5.0E-42	79%
BraA03g25530	AT3G18030	7.0E-116	98%
BraA03g25520	AT3G18010	2.0E-152	100%
BraA03g25510			
BraA03g25500	AT3G18490	0.0E+00	100%
BraA03g25490	AT3G18410	1.0E-57	99%
BraA03g25480	AT3G18400	3.0E-143	100%
BraA03g25470	AT3G18390	0.0E+00	100%
BraA03g25460	AT3G18300	6.0E-89	99%
BraA03g25450	AT3G18295	1.0E-41	99%
BraA03g25440	AT3G18280	2.0E-41	99%
BraA03g25430	AT3G18260	4.0E-105	100%
BraA03g25420			
BraA03g25410			
BraA03g25400	AT3G18520	5.0E-129	66%
BraA03g25390	AT3G18524	0.0E+00	100%
BraA03g25380	AT3G18550	9.0E-158	92%
BraA03g25370	AT3G18570	8.0E-65	99%
BraA03g25360	AT3G18590	5.0E-73	99%
BraA03g25350	AT3G18600	0.0E+00	100%
BraA03g25340	AT3G18620	2.0E-180	100%
BraA03g25330	AT3G18680	2.0E-156	100%
BraA03g25320	AT3G18690	6.0E-84	100%
BraA03g25310	AT3G18710	4.0E-170	100%
BraA03g25300			
BraA03g25290			
BraA03g25280	AT3G18715	1.0E-27	99%
BraA03g25270	AT3G18740	2.0E-59	99%
BraA03g25260	AT3G18750	0.0E+00	100%
BraA03g25250	AT3G18773	4.0E-95	99%
BraA03g25240	AT3G12110	0.0E+00	100%
BraA03g25230	AT3G18810	4.0E-55	99%
BraA03g25220	AT3G18810	9.0E-142	68%
BraA03g25210	AT3G18820	3.0E-117	100%
BraA03g25200	AT3G18830	0.0E+00	100%
BraA03g25190	AT3G18830	1.0E-82	93%
BraA03g25180	AT3G18940	7.0E-152	100%
BraA03g25170	AT3G18990	9.0E-170	100%
BraA03g25160	AT3G19000	4.0E-132	100%
BraA03g25150	AT3G19010	1.0E-07	41%
BraA03g25140	AT3G19000	9.0E-171	99%
BraA03g25130			
BraA03g25120	AT3G19020	0.0E+00	50%
BraA03g25110	AT3G24255	1.0E-12	82%
BraA03g25100	AT3G19030	4.0E-19	63%
BraA03g25090			
BraA03g25080	AT3G19090	0.0E+00	98%
BraA03g25070	AT3G19100	0.0E+00	100%
BraA03g25060	AT3G19120	0.0E+00	100%
BraA03g25050			
BraA03g25040	AT3G19130	1.0E-176	100%
BraA03g25030	AT3G03040	2.0E-13	51%

BraA03g25020	AT3G19150	6.0E-26	61%
BraA03g25010	AT3G19160	4.0E-136	100%
BraA03g25000	AT3G19170	0.0E+00	100%
BraA03g24990	AT3G06570	5.0E-07	73%
BraA03g24980	AT3G19260	7.0E-142	81%
BraA03g24970			
BraA03g24960	AT3G19270	0.0E+00	98%
BraA03g24950	AT3G19290	8.0E-131	100%
BraA03g24940	AT3G19300	1.0E-26	62%
BraA03g24930	AT3G19350	3.0E-15	56%
BraA03g24920	AT3G19350	1.0E-15	67%
BraA03g24910	AT3G19350	1.0E-15	61%
BraA03g24900	AT3G19360	5.0E-138	100%
BraA03g24890	AT3G10185	6.0E-09	60%
BraA03g24880			
BraA03g24870	AT3G19490	0.0E+00	100%
BraA03g24860	AT3G19480	0.0E+00	97%
BraA03g24850	AT3G19460	7.0E-86	88%
BraA03g24840	AT3G16740	3.0E-88	96%
BraA03g24830	AT3G16750	4.0E-07	77%
BraA03g24820			
BraA03g24810	AT3G16740	2.0E-84	89%
BraA03g24800	AT3G19580	1.0E-87	100%
BraA03g24790	AT3G19595	2.0E-118	96%
BraA03g24780	AT3G19595	7.0E-90	92%
BraA03g24770	AT3G19610	0.0E+00	99%
BraA03g24760	AT3G19615	4.0E-35	93%
BraA03g24750	AT3G19630	0.0E+00	100%
BraA03g24740			
BraA03g24730	AT3G19680	6.0E-51	99%
BraA03g24720	AT3G19690	3.0E-76	96%
BraA03g24710			
BraA03g24700	AT3G19710	9.0E-174	97%
BraA03g24690			
BraA03g24680	AT3G19760	0.0E+00	100%
BraA03g24670	AT3G19850	0.0E+00	100%
BraA03g24660			
BraA03g24650	AT3G19920	0.0E+00	99%
BraA03g24640	AT3G20030	4.0E-98	98%
BraA03g24630			
BraA03g24620	AT3G19930	4.0E-27	78%
BraA03g24610	AT3G19930	2.0E-38	89%
BraA03g24600	AT3G19930	0.0E+00	100%
BraA03g24590	AT3G19950	3.0E-119	100%
BraA03g24580	AT3G19960	0.0E+00	100%
BraA03g24570	AT3G19970	0.0E+00	59%
BraA03g24560	AT3G20000	4.0E-160	100%
BraA03g24550	AT3G20010	0.0E+00	100%
BraA03g24540	AT3G20020	0.0E+00	100%
BraA03g24530	AT3G20050	0.0E+00	100%
BraA03g24520	AT3G20080	3.0E-33	75%
BraA03g24510	AT3G20080	6.0E-42	44%
BraA03g24500	AT3G20150	0.0E+00	100%
BraA03g24490	AT3G20160	4.0E-147	100%
BraA03g24480	AT3G20170	0.0E+00	100%
BraA03g24470	AT3G20190	0.0E+00	100%
BraA03g24460	AT3G20240	8.0E-178	99%

BraA03g24450	AT3G20250	0.0E+00	77%
BraA03g24440	AT3G20260	1.0E-175	99%
BraA03g24430	AT3G20300	0.0E+00	100%
BraA03g24420			
BraA03g24410	AT3G20310	2.0E-79	100%
BraA03g24400	AT3G11070	1.0E-04	18%
BraA03g24390	AT3G20370	2.0E-37	94%
BraA03g24380	AT3G20360	3.0E-152	93%
BraA03g24370	AT3G20370	2.0E-74	100%
BraA03g24360	AT3G20370	2.0E-140	100%
BraA03g24350	AT3G20370	2.0E-102	98%
BraA03g24340	AT3G20370	1.0E-73	48%
BraA03g24330	AT3G20370	4.0E-47	76%
BraA03g24320	AT3G20390	7.0E-72	99%
BraA03g24310	AT3G20410	0.0E+00	100%
BraA03g24300	AT3G20510	2.0E-56	98%
BraA03g24290	AT3G20520	0.0E+00	100%
BraA03g24280	AT3G20530	0.0E+00	99%
BraA03g24270	AT3G20550	6.0E-117	94%
BraA03g24260	AT3G20560	0.0E+00	98%
BraA03g24250	AT3G20570	2.0E-80	100%
BraA03g24240	AT3G20580	0.0E+00	99%
BraA03g24230	AT3G20600	8.0E-93	99%
BraA03g24220	AT3G20630	0.0E+00	100%
BraA03g24210	AT3G20640	3.0E-162	100%
BraA03g24200			
BraA03g24190	AT3G20030	8.0E-123	99%
BraA03g24180	AT3G20750	2.0E-37	99%
BraA03g24170			
BraA03g24160	AT3G20770	0.0E+00	100%
BraA03g24150	AT3G20865	5.0E-14	75%
BraA03g24140	AT3G13680	4.0E-60	100%
BraA03g24130	AT3G20890	2.0E-112	95%
BraA03g24120			
BraA03g24110	AT3G20898	4.0E-39	99%
BraA03g24100	AT3G20910	1.0E-82	87%
BraA03g24090	AT3G21070	0.0E+00	99%
BraA03g24080	AT3G21055	4.0E-45	99%
BraA03g24070	AT3G20930	3.0E-09	71%
BraA03g24060	AT3G20920	2.0E-179	100%
BraA03g24050	AT3G20910	1.0E-107	100%
BraA03g24040	AT3G20898	4.0E-39	99%
BraA03g24030			
BraA03g24020	AT3G20890	2.0E-112	95%
BraA03g24010	AT3G13680	4.0E-60	100%
BraA03g24000	AT3G20865	5.0E-14	75%
BraA03g23990	AT3G20770	0.0E+00	100%
BraA03g23980			
BraA03g23970	AT3G20750	2.0E-37	99%
BraA03g23960	AT3G20030	8.0E-123	99%
BraA03g23950			
BraA03g23940	AT3G20640	2.0E-131	100%
BraA03g23930	AT3G20630	0.0E+00	100%
BraA03g23920	AT3G20600	8.0E-93	99%
BraA03g23910	AT3G20580	8.0E-168	98%
BraA03g23900	AT3G21110	0.0E+00	99%
BraA03g23890	AT3G16390	1.0E-05	66%

BraA03g23880	AT3G05420	4.0E-10	60%
BraA03g23870	AT3G21140	0.0E+00	99%
BraA03g23860	AT3G21150	1.0E-70	100%
BraA03g23850	AT3G21160	0.0E+00	100%
BraA03g23840	AT3G21180	0.0E+00	100%
BraA03g23830			
BraA03g23820	AT3G21220	3.0E-144	90%
BraA03g23810			
BraA03g23800			
BraA03g23790	AT3G21220	3.0E-166	99%
BraA03g23780			
BraA03g23770	AT3G21230	0.0E+00	99%
BraA03g23760	AT3G21230	0.0E+00	98%
BraA03g23750			
BraA03g23740	AT3G21270	1.0E-70	100%
BraA03g23730	AT3G21295	6.0E-22	58%
BraA03g23720	AT3G21320	0.0E+00	100%
BraA03g23710			
BraA03g23700	AT3G21330	4.0E-152	100%
BraA03g23690	AT3G21500	0.0E+00	88%
BraA03g23680	AT3G21490	4.0E-42	99%
BraA03g23670			
BraA03g23660	AT3G21460	5.0E-55	99%
BraA03g23650	AT3G21460	3.0E-48	99%
BraA03g23640			
BraA03g23630	AT3G21410	2.0E-107	99%
BraA03g23620	AT3G21400	1.0E-88	99%
BraA03g23610	AT3G21352	3.0E-27	94%
BraA03g23600	AT3G21340	0.0E+00	100%
BraA03g23590			
BraA03g23580			
BraA03g23570	AT3G21530	0.0E+00	54%
BraA03g23560	AT3G21600	7.0E-79	99%
BraA03g23550	AT3G21610	9.0E-89	99%
BraA03g23540	AT3G21620	0.0E+00	100%
BraA03g23530	AT3G21650	6.0E-20	79%
BraA03g23520	AT3G21680	3.0E-20	99%
BraA03g23510			
BraA03g23500	AT3G21690	0.0E+00	100%
BraA03g23490	AT3G21700	2.0E-128	100%
BraA03g23480	AT3G21710	1.0E-58	71%
BraA03g23470			
BraA03g23460			
BraA03g23450			
BraA03g23440	AT3G21720	0.0E+00	100%
BraA03g23430	AT3G21740	2.0E-18	94%
BraA03g23420	AT3G21760	0.0E+00	99%
BraA03g23410	AT3G21760	0.0E+00	99%
BraA03g23400	AT3G21790	0.0E+00	100%
BraA03g23390	AT3G21865	8.0E-128	99%
BraA03g23380	AT3G21970	2.0E-76	98%
BraA03g23370			
BraA03g23360	AT3G21970	2.0E-72	98%
BraA03g23350			
BraA03g23340	AT3G21970	3.0E-73	98%
BraA03g23330	AT3G22110	3.0E-60	96%
BraA03g23320	AT3G22142	9.0E-46	59%

BraA03g23310			
BraA03g23300	AT3G24255	1.0E-20	77%
BraA03g23290	AT3G22240	4.0E-12	65%
BraA03g23280			
BraA03g23270			
BraA03g23260			
BraA03g23250			
BraA03g23240			
BraA03g23230			
BraA03g23220			
BraA03g23210			
BraA03g23200			
BraA03g23190			
BraA03g23180			
BraA03g23170			
BraA03g23160			
BraA03g23150			
BraA03g23140	AT3G22290	0.0E+00	99%
BraA03g23130	AT3G22300	1.0E-80	81%
BraA03g23120	AT3G22320	9.0E-114	99%
BraA03g23110	AT3G22370	2.0E-74	96%
BraA03g23100	AT3G22380	4.0E-94	99%
BraA03g23090	AT3G22380	0.0E+00	100%
BraA03g23080	AT3G22420	0.0E+00	100%
BraA03g23070	AT3G22480	9.0E-69	99%
BraA03g23060	AT3G22490	9.0E-81	84%
BraA03g23050	AT3G22520	0.0E+00	99%
BraA03g23040			
BraA03g23030	AT3G22540	8.0E-52	99%
BraA03g23020	AT3G22550	2.0E-113	99%
BraA03g23010			
BraA03g23000	AT3G22600	6.0E-72	98%
BraA03g22990	AT3G22620	1.0E-84	100%
BraA03g22980	AT3G22650	8.0E-154	100%
BraA03g22970	AT3G22660	5.0E-103	100%
BraA03g22960	AT3G12490	8.0E-07	57%
BraA03g22950	AT3G22670	4.0E-56	99%
BraA03g22940	AT3G12490	4.0E-06	54%
BraA03g22930	AT3G22670	0.0E+00	100%
BraA03g22920	AT3G22680	4.0E-69	94%
BraA03g22910	AT3G22720	7.0E-84	88%
BraA03g22900	AT3G22760	5.0E-173	100%
BraA03g22890	AT3G22780	0.0E+00	99%
BraA03g22880	AT3G22830	1.0E-149	100%
BraA03g22870	AT3G22840	5.0E-83	99%
BraA03g22860	AT3G14225	6.0E-07	52%
BraA03g22850	AT3G22845	3.0E-118	100%
BraA03g22840	AT3G22850	5.0E-129	100%
BraA03g22830	AT3G22880	2.0E-34	99%
BraA03g22820	AT3G22890	0.0E+00	99%
BraA03g22810			
BraA03g22800	AT3G22930	1.0E-76	91%
BraA03g22790			
BraA03g22780			
BraA03g22770	AT3G22942	4.0E-45	98%
BraA03g22760			
BraA03g22750	AT3G22960	0.0E+00	100%

BraA03g22740	AT3G09510	5.0E-04	78%
BraA03g22730	AT3G22970	2.0E-142	100%
BraA03g22720	AT3G23030	6.0E-83	99%
BraA03g22710			
BraA03g22700	AT3G16030	0.0E+00	100%
BraA03g22690	AT3G23140	5.0E-38	89%
BraA03g22680	AT3G23170	7.0E-33	63%
BraA03g22670	AT3G23180	1.0E-99	100%
BraA03g22660			
BraA03g22650	AT3G23250	8.0E-68	72%
BraA03g22640			
BraA03g22630			
BraA03g22620	AT3G21130	8.0E-50	93%
BraA03g22610	AT3G23300	0.0E+00	100%
BraA03g22600	AT3G23380	1.0E-60	99%
BraA03g22590	AT3G23390	3.0E-54	99%
BraA03g22580	AT3G23410	0.0E+00	99%
BraA03g22570			
BraA03g22560	AT3G23050	4.0E-84	99%
BraA03g22550			
BraA03g22540			
BraA03g22530	AT3G24255	2.0E-08	79%
BraA03g22520	AT3G23060	9.0E-74	97%
BraA03g22510	AT3G22060	1.0E-23	86%
BraA03g22500	AT3G24130	1.0E-153	99%
BraA03g22490	AT3G24120	4.0E-143	100%
BraA03g22480	AT3G24090	0.0E+00	100%
BraA03g22470	AT3G06910	3.0E-04	24%
BraA03g22460	AT3G24050	3.0E-87	100%
BraA03g22450	AT3G23870	2.0E-152	100%
BraA03g22440	AT3G23820	0.0E+00	99%
BraA03g22430	AT3G23830	3.0E-32	52%
BraA03g22420	AT3G23810	0.0E+00	100%
BraA03g22410	AT3G05490	3.0E-09	58%
BraA03g22400	AT3G24140	5.0E-174	100%
BraA03g22390	AT3G24570	3.0E-120	100%
BraA03g22380	AT3G10460	5.0E-09	71%
BraA03g22370			
BraA03g22360	AT3G24620	0.0E+00	99%
BraA03g22350	AT3G24255	4.0E-05	46%
BraA03g22340	AT3G24650	0.0E+00	100%
BraA03g22330	AT3G24660	0.0E+00	77%
BraA03g22320			
BraA03g22310			
BraA03g22300	AT3G24715	0.0E+00	100%
BraA03g22290	AT3G24730	2.0E-69	98%
BraA03g22280			
BraA03g22270	AT3G24800	4.0E-160	100%
BraA03g22260	AT3G24810	5.0E-59	100%
BraA03g22250	AT3G24830	3.0E-115	100%
BraA03g22240	AT3G24880	0.0E+00	100%
BraA03g22230	AT3G25040	1.0E-119	100%
BraA03g22220	AT3G25060	0.0E+00	98%
BraA03g22210	AT3G09260	3.0E-50	82%
BraA03g22200	AT3G25070	4.0E-91	98%
BraA03g22190	AT3G25100	0.0E+00	100%
BraA03g22180	AT3G25110	2.0E-29	77%

BraA03g22170	AT3G25110	3.0E-176	99%	
BraA03g22160	AT3G25140	0.0E+00	100%	
BraA03g22150	AT3G25160	8.0E-147	100%	
BraA03g22140	AT3G25180	0.0E+00	99%	
BraA03g22130	AT3G25190	4.0E-92	100%	
BraA03g22120	AT3G25220	6.0E-73	97%	
BraA03g22110	AT3G25230	0.0E+00	100%	
BraA03g22100	AT3G25240	3.0E-115	99%	
BraA03g22090	AT3G25260	0.0E+00	100%	
BraA03g22080	AT3G25260	1.0E-120	99%	
BraA03g22070	AT3G25260	6.0E-119	100%	
BraA03g22060	AT3G25460	9.0E-85	95%	
BraA03g22050	AT3G25470	4.0E-150	99%	
BraA03g22040	AT3G25480	2.0E-115	100%	
BraA03g22030	AT3G25500	0.0E+00	98%	
BraA03g22020				
BraA03g22010	AT3G25520	2.0E-164	96%	
BraA03g22000	AT3G25520	2.0E-163	96%	
BraA03g21990	AT2G04050	0.0E+00	100%	G
BraA03g21980				
BraA03g21970	AT2G04160	0.0E+00	100%	
BraA03g21960	AT2G04240	4.0E-61	99%	
BraA03g21950	AT2G04350	0.0E+00	95%	
BraA03g21940	AT2G04400	0.0E+00	100%	
BraA03g21930	AT2G04410	3.0E-26	39%	
BraA03g21920				
BraA03g21910				
BraA03g21900	AT2G04515	1.0E-43	97%	
BraA03g21890	AT2G04520	9.0E-74	99%	
BraA03g21880	AT2G04530	0.0E+00	100%	
BraA03g21870	AT2G04540	0.0E+00	100%	
BraA03g21860	AT2G04550	1.0E-129	100%	
BraA03g21850	AT2G04570	0.0E+00	100%	
BraA03g21840	AT2G04300	6.0E-19	27%	
BraA03g21830	AT2G04740	0.0E+00	100%	
BraA03g21820	AT2G04780	9.0E-109	100%	
BraA03g21810	AT2G04790	2.0E-65	80%	
BraA03g21800				
BraA03g21790				
BraA03g21780				
BraA03g21770	AT2G04795	4.0E-40	99%	
BraA03g21760	AT2G04842	0.0E+00	97%	
BraA03g21750	AT2G04845	5.0E-50	98%	
BraA03g21740				
BraA03g21730				
BraA03g21720				
BraA03g21710	AT2G05070	1.0E-153	100%	
BraA03g21700				
BraA03g21690				
BraA03g21680				
BraA03g21670				
BraA03g21660				
BraA03g21650				
BraA03g21640				
BraA03g21630				
BraA03g21620				
BraA03g21610				

BraA03g21600				
BraA03g21590				
BraA03g21580				
BraA03g21570				
BraA03g21560				
BraA03g21550				
BraA03g21540				
BraA03g21530				
BraA03g21520				
BraA03g21510				
BraA03g21500				
BraA03g21490				
BraA03g21480				
BraA03g21470				
BraA03g21460				
BraA03g21450				
BraA03g21440				
BraA03g21430	AT2G14510	7.0E-58	60%	H
BraA03g21420				
BraA03g21410				
BraA03g21400				
BraA03g21390				
BraA03g21380	AT2G11910	8.0E-15	98%	
BraA03g21370				
BraA03g21360				
BraA03g21350				
BraA03g21340				
BraA03g21330				
BraA03g21320				
BraA03g21310				
BraA03g21300	AT2G13360	0.0E+00	100%	
BraA03g21290				
BraA03g21280				
BraA03g21270				
BraA03g21260	AT2G15680	2.0E-15	53%	
BraA03g21250				
BraA03g21240	AT2G15690	2.0E-31	30%	
BraA03g21230				
BraA03g21220				
BraA03g21210	AT2G14510	1.0E-15	41%	
BraA03g21200				
BraA03g21190				
BraA03g21180				
BraA03g21170	AT2G14510	2.0E-62	77%	
BraA03g21160				
BraA03g21150	AT2G15050	3.0E-04	46%	
BraA03g21140	AT2G15080	4.0E-10	18%	
BraA03g21130				
BraA03g21120				
BraA03g21110				
BraA03g21100	AT2G16440	2.0E-62	82%	
BraA03g21090				
BraA03g21080				
BraA03g21070				
BraA03g21060	AT2G14760	1.0E-05	23%	
BraA03g21050				
BraA03g21040	AT2G14520	0.0E+00	100%	

BraA03g21030			
BraA03g21020	AT2G14530	4.0E-99	71%
BraA03g21010	AT2G14580	2.0E-66	99%
BraA03g21000	AT2G14580	6.0E-74	99%
BraA03g20990			
BraA03g20980	AT2G14740	0.0E+00	96%
BraA03g20970	AT2G14750	8.0E-144	99%
BraA03g20960	AT2G15080	1.0E-33	80%
BraA03g20950	AT2G14835	9.0E-178	100%
BraA03g20940	AT2G14850	1.0E-96	97%
BraA03g20930			
BraA03g20920	AT2G14890	4.0E-06	11%
BraA03g20910	AT2G14900	5.0E-36	99%
BraA03g20900	AT2G15000	2.0E-40	57%
BraA03g20890	AT2G15020	0.0E+00	100%
BraA03g20880	AT2G15080	2.0E-180	93%
BraA03g20870	AT2G15220	6.0E-109	100%
BraA03g20860	AT2G15260	2.0E-25	25%
BraA03g20850			
BraA03g20840			
BraA03g20830			
BraA03g20820	AT2G15440	2.0E-114	100%
BraA03g20810			
BraA03g20800	AT2G15480	0.0E+00	100%
BraA03g20790	AT2G15500	1.0E-21	27%
BraA03g20780			
BraA03g20770			
BraA03g20760	AT2G15580	6.0E-44	88%
BraA03g20750			
BraA03g20740	AT2G15580	4.0E-43	88%
BraA03g20730			
BraA03g20720			
BraA03g20710	AT2G15690	0.0E+00	100%
BraA03g20700	AT2G15695	0.0E+00	100%
BraA03g20690			
BraA03g20680	AT2G15730	0.0E+00	100%
BraA03g20670			
BraA03g20660	AT2G15860	0.0E+00	99%
BraA03g20650	AT1G65140	6.0E-74	99%
BraA03g20640	AT2G15880	5.0E-170	79%
BraA03g20630	AT2G15890	8.0E-89	100%
BraA03g20620	AT2G15910	2.0E-160	100%
BraA03g20610	AT2G15960	2.0E-23	99%
BraA03g20600	AT2G15970	1.0E-87	99%
BraA03g20590	AT2G14960	4.0E-97	97%
BraA03g20580	AT2G16070	5.0E-89	67%
BraA03g20570			
BraA03g20560	AT2G14710	1.0E-04	33%
BraA03g20550	AT2G16250	0.0E+00	91%
BraA03g20540			
BraA03g20530	AT2G16280	0.0E+00	100%
BraA03g20520	AT2G16365	8.0E-153	98%
BraA03g20510	AT2G16360	4.0E-41	74%
BraA03g20500	AT2G16370	0.0E+00	100%
BraA03g20490			
BraA03g20480	AT2G16380	0.0E+00	96%
BraA03g20470	AT2G16390	0.0E+00	100%

BraA03g20460	AT2G16400	0.0E+00	100%	
BraA03g20450	AT2G16430	0.0E+00	83%	
BraA03g20440				
BraA03g20430	AT2G16430	1.0E-14	35%	
BraA03g20420	AT2G16430	0.0E+00	73%	
BraA03g20410	AT2G16440	0.0E+00	100%	
BraA03g20400				CD6
BraA03g20390				
BraA03g20380				
BraA03g20370				
BraA03g20360				
BraA03g20350				
BraA03g20340	AT3G51330	3.0E-130	91%	
BraA03g20330				
BraA03g20320				
BraA03g20310				
BraA03g20300	AT3G46020	1.0E-13	80%	
BraA03g20290	AT5G61040	0.0E+00	94%	
BraA03g20280				
BraA03g20270				
BraA03g20260				
BraA03g20250				
BraA03g20240				
BraA03g20230				
BraA03g20220	AT1G52950	1.0E-38	98%	
BraA03g20210				
BraA03g20200				
BraA03g20190	AT5G61120	7.0E-140	99%	
BraA03g20180				
BraA03g20170				
BraA03g20160				
BraA03g20150				
BraA03g20140				
BraA03g20130				
BraA03g20120				
BraA03g20110				
BraA03g20100				
BraA03g20090				
BraA03g20080	AT5G61120	5.0E-157	95%	
BraA03g20070				
BraA03g20060				
BraA03g20050				
BraA03g20040				
BraA03g20030				
BraA03g20020				
BraA03g20010				
BraA03g20000				
BraA03g19990				
BraA03g19980				
BraA03g19970				
BraA03g19960				
BraA03g19950				
BraA03g19940	AT3G49670	2.0E-31	76%	M
BraA03g19930				
BraA03g19920				
BraA03g19910				
BraA03g19900	AT3G48940	5.0E-05	42%	

BraA03g19890			
BraA03g19880			
BraA03g19870			
BraA03g19860			
BraA03g19850	AT5G61360	9.0E-37	91%
BraA03g19840	AT3G49730	2.0E-39	82%
BraA03g19830			
BraA03g19820	AT3G48100	4.0E-10	17%
BraA03g19810			
BraA03g19800	AT3G47600	4.0E-30	28%
BraA03g19790	AT3G49530	1.0E-43	46%
BraA03g19780			
BraA03g19770			
BraA03g19760			
BraA03g19750	AT3G47460	5.0E-06	14%
BraA03g19740			
BraA03g19730			
BraA03g19720	AT3G49930	1.0E-13	52%
BraA03g19710	AT3G49670	0.0E+00	93%
BraA03g19700	AT5G61490	9.0E-89	77%
BraA03g19690			
BraA03g19680			
BraA03g19670			
BraA03g19660			
BraA03g19650			
BraA03g19640			
BraA03g19630	AT3G49060	9.0E-77	44%
BraA03g19620			
BraA03g19610			
BraA03g19600	AT3G49060	6.0E-82	60%
BraA03g19590	AT3G49670	6.0E-30	79%
BraA03g19580	AT3G47840	6.0E-105	97%
BraA03g19570			
BraA03g19560			
BraA03g19550			
BraA03g19540			
BraA03g19530			
BraA03g19520			
BraA03g19510			
BraA03g19500			
BraA03g19490	AT5G61650	4.0E-54	59%
BraA03g19480			
BraA03g19470			
BraA03g19460	AT3G49220	2.0E-31	84%
BraA03g19450	AT3G47780	0.0E+00	99%
BraA03g19440	AT3G47730	0.0E+00	100%
BraA03g19430			
BraA03g19420			
BraA03g19410	AT3G47730	0.0E+00	100%
BraA03g19400	AT3G47780	0.0E+00	100%
BraA03g19390			
BraA03g19380			
BraA03g19370	AT3G49670	0.0E+00	82%
BraA03g19360			
BraA03g19350			
BraA03g19340	AT3G49700	0.0E+00	100%
BraA03g19330			

BraA03g19320				
BraA03g19310	AT3G49740	0.0E+00	100%	
BraA03g19300				
BraA03g19290	AT3G49790	5.0E-155	100%	
BraA03g19280				
BraA03g19270				
BraA03g19260	AT3G49850	4.0E-47	73%	
BraA03g19250	AT3G49870	2.0E-104	99%	
BraA03g19240	AT3G49890	1.0E-92	94%	
BraA03g19230	AT3G49910	3.0E-80	99%	
BraA03g19220	AT3G49920	1.0E-93	100%	
BraA03g19210				
BraA03g19200	AT3G50510	3.0E-07	36%	
BraA03g19190	AT3G49990	0.0E+00	100%	
BraA03g19180	AT3G50040	1.0E-84	99%	N
BraA03g19170	AT3G50060	6.0E-112	100%	
BraA03g19160	AT3G50070	8.0E-133	100%	
BraA03g19150	AT3G50110	0.0E+00	98%	
BraA03g19140	AT3G50120	0.0E+00	100%	
BraA03g19130	AT3G50130	0.0E+00	100%	
BraA03g19120	AT3G50130	0.0E+00	100%	
BraA03g19110	AT3G50170	0.0E+00	100%	
BraA03g19100				
BraA03g19090				
BraA03g19080				
BraA03g19070	AT3G50390	0.0E+00	100%	
BraA03g19060	AT3G50900	4.0E-41	99%	
BraA03g19050				
BraA03g19040	AT3G50890	3.0E-98	96%	
BraA03g19030	AT3G50870	1.0E-112	100%	
BraA03g19020				
BraA03g19010				
BraA03g19000				
BraA03g18990	AT3G50860	6.0E-92	98%	
BraA03g18980	AT3G50830	9.0E-105	99%	
BraA03g18970	AT3G50800	1.0E-58	99%	
BraA03g18960	AT3G50770	6.0E-98	100%	
BraA03g18950	AT3G50760	0.0E+00	100%	
BraA03g18940	AT3G50750	4.0E-116	100%	
BraA03g18930	AT3G50740	0.0E+00	99%	
BraA03g18920	AT3G52130	3.0E-49	81%	
BraA03g18910	AT3G52100	0.0E+00	100%	
BraA03g18900	AT3G52090	1.0E-27	68%	
BraA03g18890	AT3G52080	0.0E+00	100%	
BraA03g18880				
BraA03g18870	AT3G52040	5.0E-40	98%	
BraA03g18860	AT3G52030	0.0E+00	100%	
BraA03g18850	AT3G52010	3.0E-42	91%	
BraA03g18840				
BraA03g18830				
BraA03g18820	AT3G51950	0.0E+00	91%	
BraA03g18810				
BraA03g18800	AT3G51910	1.0E-111	99%	
BraA03g18790	AT3G51910	5.0E-47	98%	
BraA03g18780	AT3G52155	2.0E-108	100%	
BraA03g18770	AT3G52160	0.0E+00	100%	
BraA03g18760	AT3G52170	1.0E-130	100%	

BraA03g18750	AT3G52250	0.0E+00	83%	
BraA03g18740	AT3G52260	5.0E-136	75%	
BraA03g18730	AT3G51325	2.0E-08	40%	
BraA03g18720	AT3G52270	1.0E-99	96%	
BraA03g18710				
BraA03g18700	AT3G52270	7.0E-24	81%	
BraA03g18690	AT3G52280	5.0E-147	99%	
BraA03g18680	AT3G52300	6.0E-92	99%	
BraA03g18670	AT3G52320	8.0E-69	81%	
BraA03g18660				
BraA03g18650	AT3G52380	6.0E-132	99%	
BraA03g18640	AT3G52400	2.0E-125	100%	
BraA03g18630	AT3G52420	3.0E-22	73%	
BraA03g18620	AT3G52430	0.0E+00	100%	
BraA03g18610	AT3G52440	8.0E-113	100%	
BraA03g18600	AT3G52450	0.0E+00	100%	
BraA03g18590	AT3G52460	1.0E-111	100%	
BraA03g18580	AT3G52470	2.0E-111	100%	
BraA03g18570				CD7
BraA03g18560				
BraA03g18550				
BraA03g18540				
BraA03g18530	AT4G15233	6.0E-179	100%	T
BraA03g18520	AT4G15236	0.0E+00	100%	
BraA03g18510	AT4G15240	1.0E-17	51%	
BraA03g18500	AT4G15240	0.0E+00	100%	
BraA03g18490				
BraA03g18480	AT4G15280	0.0E+00	99%	
BraA03g18470	AT4G15400	6.0E-100	99%	
BraA03g18460	AT4G15310	6.0E-50	85%	
BraA03g18450	AT4G15310	2.0E-87	92%	
BraA03g18440	AT4G15396	0.0E+00	98%	
BraA03g18430				
BraA03g18420	AT4G15410	6.0E-157	98%	
BraA03g18410	AT4G15415	0.0E+00	100%	
BraA03g18400	AT4G15417	4.0E-89	99%	
BraA03g18390				
BraA03g18380	AT4G15440	0.0E+00	84%	
BraA03g18370	AT4G15470	1.0E-143	100%	
BraA03g18360	AT4G15475	0.0E+00	97%	
BraA03g18350				
BraA03g18340	AT4G15480	0.0E+00	100%	
BraA03g18330				
BraA03g18320	AT4G15520	1.0E-120	100%	
BraA03g18310	AT4G15530	0.0E+00	83%	
BraA03g18300	AT4G15550	6.0E-33	99%	
BraA03g18290	AT4G15550	6.0E-166	99%	
BraA03g18280	AT4G15560	0.0E+00	100%	
BraA03g18270				
BraA03g18260	AT2G06845	2.0E-05	25%	
BraA03g18250	AT4G15610	2.0E-66	90%	
BraA03g18240				
BraA03g18230				
BraA03g18220	AT4G15630	3.0E-94	99%	
BraA03g18210	AT4G15650	3.0E-55	95%	
BraA03g18200	AT4G15680	1.0E-55	99%	
BraA03g18190	AT4G15720	0.0E+00	100%	

BraA03g18180	AT4G15740	3.0E-47	89%	
BraA03g18170	AT4G15740	6.0E-173	95%	
BraA03g18160				
BraA03g18150	AT4G15755	2.0E-41	83%	
BraA03g18140	AT4G15760	0.0E+00	100%	
BraA03g18130				
BraA03g18120	AT4G15770	2.0E-104	99%	
BraA03g18110				
BraA03g18100	AT4G15800	2.0E-46	99%	
BraA03g18090				
BraA03g18080	AT4G15820	1.0E-152	100%	
BraA03g18070	AT4G15840	0.0E+00	100%	
BraA03g18060				
BraA03g18050				
BraA03g18040				
BraA03g18030	AT4G15890	1.0E-15	65%	
BraA03g18020	AT4G15880	0.0E+00	100%	
BraA03g18010	AT3G47350	7.0E-134	100%	
BraA03g18000				
BraA03g17990	AT4G16141	6.0E-08	31%	
BraA03g17980	AT4G15885	2.0E-53	14%	
BraA03g17970	AT4G15890	0.0E+00	100%	
BraA03g17960				
BraA03g17950	AT4G15900	0.0E+00	100%	
BraA03g17940	AT4G15920	1.0E-125	100%	
BraA03g17930	AT4G15930	4.0E-60	99%	
BraA03g17920	AT4G15960	2.0E-121	98%	
BraA03g17910	AT4G16060	9.0E-133	96%	
BraA03g17900	AT4G15990	3.0E-52	99%	
BraA03g17890	AT4G15960	5.0E-173	99%	
BraA03g17880	AT4G12720	3.0E-136	99%	
BraA03g17870				
BraA03g17860	AT4G16070	2.0E-160	100%	
BraA03g17850	AT4G16070	3.0E-33	100%	
BraA03g17840	AT4G15990	3.0E-52	99%	
BraA03g17830	AT4G15960	5.0E-173	99%	
BraA03g17820	AT4G12720	3.0E-136	99%	
BraA03g17810				
BraA03g17800	AT4G16070	0.0E+00	100%	
BraA03g17790	AT4G16100	4.0E-140	100%	
BraA03g17780				
BraA03g17770	AT4G16110	0.0E+00	99%	
BraA03g17760				
BraA03g17750	AT4G16141	4.0E-55	99%	
BraA03g17740	AT4G16143	2.0E-177	100%	
BraA03g17730	AT4G16143	1.0E-66	97%	
BraA03g17720	AT4G16144	0.0E+00	99%	U
BraA03g17710				
BraA03g17700	AT4G16146	2.0E-33	59%	
BraA03g17690				
BraA03g17680	AT4G16155	2.0E-70	100%	
BraA03g17670	AT4G16160	2.0E-78	99%	
BraA03g17660	AT4G30410	3.0E-04	62%	
BraA03g17650	AT4G16170	0.0E+00	56%	
BraA03g17640	AT4G16190	7.0E-178	99%	
BraA03g17630	AT4G16195	1.0E-59	99%	
BraA03g17620	AT4G16210	1.0E-143	99%	

BraA03g17610			
BraA03g17600			
BraA03g17590	AT4G16230	5.0E-124	67%
BraA03g17580			
BraA03g17570			
BraA03g17560	AT4G16360	3.0E-132	87%
BraA03g17550	AT4G16380	6.0E-38	99%
BraA03g17540	AT4G16400	3.0E-18	92%
BraA03g17530	AT4G16400	5.0E-39	99%
BraA03g17520	AT4G16410	2.0E-69	98%
BraA03g17510	AT4G16420	0.0E+00	100%
BraA03g17500	AT4G16430	0.0E+00	100%
BraA03g17490	AT4G16442	2.0E-73	87%
BraA03g17480	AT4G16444	1.0E-69	82%
BraA03g17470	AT4G16447	2.0E-43	99%
BraA03g17460	AT4G16450	9.0E-52	96%
BraA03g17450	AT4G16460	2.0E-45	99%
BraA03g17440	AT4G16480	0.0E+00	100%
BraA03g17430	AT4G16490	0.0E+00	100%
BraA03g17420			
BraA03g17410	AT4G16500	7.0E-16	74%
BraA03g17400			
BraA03g17390	AT4G16515	2.0E-16	73%
BraA03g17380	AT4G16520	8.0E-60	98%
BraA03g17370			
BraA03g17360			
BraA03g17350	AT4G16530	8.0E-63	97%
BraA03g17340	AT4G16550	2.0E-44	87%
BraA03g17330	AT4G16550	1.0E-38	95%
BraA03g17320	AT4G23210	3.0E-71	51%
BraA03g17310	AT4G16550	3.0E-103	95%
BraA03g17300	AT4G16560	2.0E-84	87%
BraA03g17290	AT4G16540	1.0E-36	99%
BraA03g17280	AT4G16540	8.0E-16	94%
BraA03g17270			
BraA03g17260	AT4G16590	0.0E+00	70%
BraA03g17250	AT4G16610	1.0E-77	99%
BraA03g17240	AT4G16620	3.0E-159	99%
BraA03g17230	AT4G26340	6.0E-17	81%
BraA03g17220	AT4G16640	2.0E-161	100%
BraA03g17210			
BraA03g17200	AT4G28010	2.0E-14	69%
BraA03g17190	AT4G16660	0.0E+00	59%
BraA03g17180			
BraA03g17170	AT4G16720	6.0E-116	100%
BraA03g17160	AT4G16745	7.0E-47	99%
BraA03g17150			
BraA03g17140	AT4G16765	4.0E-123	79%
BraA03g17130	AT4G16820	0.0E+00	100%
BraA03g17120	AT4G16890	0.0E+00	72%
BraA03g17110	AT4G16970	2.0E-13	44%
BraA03g17100	AT4G16890	0.0E+00	76%
BraA03g17090	AT4G16980	1.0E-13	22%
BraA03g17080	AT4G17030	4.0E-133	100%
BraA03g17070			
BraA03g17060			
BraA03g17050	AT4G17200	2.0E-30	95%

BraA03g17040	AT4G17030	2.0E-124	100%
BraA03g17030	AT4G17040	1.0E-158	100%
BraA03g17020	AT4G17050	7.0E-116	100%
BraA03g17010			
BraA03g17000	AT4G33150	4.0E-11	46%
BraA03g16990	AT4G21120	1.0E-127	95%
BraA03g16980			
BraA03g16970			
BraA03g16960	AT4G27780	5.0E-05	25%
BraA03g16950			
BraA03g16940	AT4G17090	0.0E+00	100%
BraA03g16930	AT4G17100	0.0E+00	100%
BraA03g16920	AT4G17170	5.0E-23	46%
BraA03g16910			
BraA03g16900			
BraA03g16890	AT4G17210	0.0E+00	100%
BraA03g16880			
BraA03g16870			
BraA03g16860	AT4G17215	1.0E-53	99%
BraA03g16850	AT4G17220	0.0E+00	100%
BraA03g16840	AT4G17250	0.0E+00	100%
BraA03g16830	AT4G17260	0.0E+00	100%
BraA03g16820	AT4G17270	0.0E+00	99%
BraA03g16810	AT4G17350	3.0E-97	89%
BraA03g16800	AT4G17370	0.0E+00	99%
BraA03g16790	AT4G16720	6.0E-116	100%
BraA03g16780			
BraA03g16770			
BraA03g16760			
BraA03g16750	AT4G17440	4.0E-51	72%
BraA03g16740			
BraA03g16730	AT4G17460	2.0E-141	100%
BraA03g16720	AT4G17483	1.0E-136	99%
BraA03g16710	AT4G17486	3.0E-106	97%
BraA03g16700	AT4G17510	2.0E-125	100%
BraA03g16690	AT4G17520	4.0E-120	100%
BraA03g16680	AT4G17530	6.0E-116	100%
BraA03g16670	AT4G17550	0.0E+00	82%
BraA03g16660	AT4G17600	3.0E-69	77%
BraA03g16650	AT4G17615	2.0E-118	100%
BraA03g16640	AT4G17616	0.0E+00	100%
BraA03g16630	AT4G17620	0.0E+00	98%
BraA03g16620	AT4G17640	1.0E-156	100%
BraA03g16610	AT4G17670	2.0E-57	99%
BraA03g16600			
BraA03g16590	AT4G17680	9.0E-106	99%
BraA03g16580	AT4G17690	2.0E-157	95%
BraA03g16570	AT4G17720	6.0E-71	98%
BraA03g16560	AT4G17718	1.0E-32	99%
BraA03g16550	AT4G17720	6.0E-71	99%
BraA03g16540	AT4G17718	1.0E-33	53%
BraA03g16530			
BraA03g16520	AT4G17720	5.0E-154	96%
BraA03g16510	AT4G17730	3.0E-113	88%
BraA03g16500	AT4G17740	0.0E+00	100%
BraA03g16490	AT4G17760	3.0E-169	100%
BraA03g16480	AT4G17770	0.0E+00	100%

BraA03g16470	AT4G17780	1.0E-105	81%
BraA03g16460	AT4G17785	8.0E-72	100%
BraA03g16450	AT4G17785	4.0E-45	99%
BraA03g16440	AT4G17790	4.0E-129	98%
BraA03g16430	AT4G17800	6.0E-124	100%
BraA03g16420	AT4G17810	3.0E-88	100%
BraA03g16410	AT4G17830	0.0E+00	100%
BraA03g16400	AT4G17840	0.0E+00	100%
BraA03g16390	AT4G17870	7.0E-104	99%
BraA03g16380	AT4G17890	0.0E+00	100%
BraA03g16370	AT4G17895	3.0E-178	100%
BraA03g16360			
BraA03g16350	AT4G17900	2.0E-116	100%
BraA03g16340	AT4G19900	4.0E-46	87%
BraA03g16330	AT4G17920	4.0E-127	96%
BraA03g16320	AT4G17950	3.0E-53	95%
BraA03g16310	AT4G20480	5.0E-19	28%
BraA03g16300	AT4G17970	0.0E+00	100%
BraA03g16290			
BraA03g16280	AT4G17980	7.0E-121	87%
BraA03g16270	AT4G35230	4.0E-31	92%
BraA03g16260	AT4G35230	6.0E-56	98%
BraA03g16250			
BraA03g16240	AT4G35230	7.0E-64	98%
BraA03g16230			
BraA03g16220	AT4G35230	2.0E-13	23%
BraA03g16210	AT4G35230	7.0E-64	98%
BraA03g16200			
BraA03g16190	AT4G35230	2.0E-132	88%
BraA03g16180			
BraA03g16170	AT4G18010	0.0E+00	100%
BraA03g16160	AT4G18020	0.0E+00	100%
BraA03g16150			
BraA03g16140			
BraA03g16130	AT4G18040	1.0E-71	96%
BraA03g16120			
BraA03g16110	AT4G18050	0.0E+00	98%
BraA03g16100	AT4G18060	0.0E+00	100%
BraA03g16090	AT4G18070	5.0E-33	28%
BraA03g16080	AT4G25960	9.0E-47	68%
BraA03g16070			
BraA03g16060	AT4G18100	5.0E-75	99%
BraA03g16050			
BraA03g16040	AT4G17713	4.0E-17	98%
BraA03g16030	AT4G18160	0.0E+00	98%
BraA03g16020	AT4G18170	2.0E-143	99%
BraA03g16010	AT4G18180	0.0E+00	93%
BraA03g16000	AT4G18205	8.0E-175	99%
BraA03g15990	AT4G18230	7.0E-103	100%
BraA03g15980			
BraA03g15970			
BraA03g15960	AT4G18250	6.0E-159	99%
BraA03g15950	AT4G18250	1.0E-87	92%
BraA03g15940	AT4G18260	0.0E+00	97%
BraA03g15930	AT4G18270	0.0E+00	84%
BraA03g15920			
BraA03g15910	AT4G18340	0.0E+00	99%

BraA03g15900			
BraA03g15890			
BraA03g15880	AT4G18350	0.0E+00	99%
BraA03g15870	AT4G18360	0.0E+00	100%
BraA03g15860	AT4G18372	2.0E-52	97%
BraA03g15850	AT4G18390	5.0E-151	96%
BraA03g15840	AT4G18400	2.0E-36	99%
BraA03g15830			
BraA03g15820			
BraA03g15810	AT4G18430	4.0E-125	99%
BraA03g15800	AT4G18440	0.0E+00	100%
BraA03g15790	AT4G18480	0.0E+00	100%
BraA03g15780	AT4G18490	0.0E+00	100%
BraA03g15770	AT4G18501	5.0E-32	99%
BraA03g15760	AT4G18510	7.0E-15	76%
BraA03g15750	AT4G18520	0.0E+00	100%
BraA03g15740	AT4G18530	8.0E-170	95%
BraA03g15730			
BraA03g15720	AT4G18540	0.0E+00	100%
BraA03g15710	AT4G18550	5.0E-109	61%
BraA03g15700	AT4G18570	5.0E-148	100%
BraA03g15690	AT4G18580	4.0E-04	33%
BraA03g15680	AT4G18596	1.0E-89	99%
BraA03g15670	AT4G18610	1.0E-83	97%
BraA03g15660			
BraA03g15650	AT4G18630	3.0E-150	100%
BraA03g15640			
BraA03g15630	AT4G18690	2.0E-101	99%
BraA03g15620			
BraA03g15610	AT4G18710	0.0E+00	100%
BraA03g15600	AT4G18730	2.0E-105	99%
BraA03g15590	AT4G18770	3.0E-144	99%
BraA03g15580	AT4G18780	0.0E+00	100%
BraA03g15570	AT4G18790	0.0E+00	100%
BraA03g15560	AT4G18800	1.0E-115	100%
BraA03g15550	AT4G18823	4.0E-22	99%
BraA03g15540	AT4G18830	8.0E-120	91%
BraA03g15530	AT4G19540	6.0E-33	84%
BraA03g15520			
BraA03g15510	AT4G20480	8.0E-17	52%
BraA03g15500	AT4G22820	3.0E-28	51%
BraA03g15490	AT4G20480	2.0E-16	24%
BraA03g15480	AT4G18880	5.0E-168	100%
BraA03g15470	AT4G18890	1.0E-134	100%
BraA03g15460			
BraA03g15450	AT4G18905	2.0E-12	46%
BraA03g15440	AT4G18910	1.0E-152	100%
BraA03g15430	AT4G18920	1.0E-113	96%
BraA03g15420			
BraA03g15410			
BraA03g15400	AT4G18960	2.0E-89	84%
BraA03g15390	AT4G18970	0.0E+00	100%
BraA03g15380			
BraA03g15370			
BraA03g15360			
BraA03g15350	AT4G18980	1.0E-37	99%
BraA03g15340	AT4G18990	6.0E-169	99%

BraA03g15330	AT4G19040	0.0E+00	95%
BraA03g15320	AT4G19070	1.0E-78	99%
BraA03g15310	AT4G19220	0.0E+00	98%
BraA03g15300			
BraA03g15290	AT4G19230	0.0E+00	100%
BraA03g15280			
BraA03g15270	AT4G19360	6.0E-09	75%
BraA03g15260	AT4G19360	6.0E-41	75%
BraA03g15250	AT4G19380	0.0E+00	94%
BraA03g15240	AT4G19430	1.0E-60	97%
BraA03g15230	AT4G23160	9.0E-68	90%
BraA03g15220	AT4G19450	0.0E+00	100%
BraA03g15210	AT4G19460	0.0E+00	92%
BraA03g15200	AT4G19510	0.0E+00	97%
BraA03g15190	AT4G19500	0.0E+00	61%
BraA03g15180	AT4G19490	0.0E+00	100%
BraA03g15170	AT4G19512	9.0E-26	67%
BraA03g15160			
BraA03g15150	AT4G19930	3.0E-124	100%
BraA03g15140	AT4G19670	9.0E-123	94%
BraA03g15130	AT4G19680	2.0E-119	70%
BraA03g15120	AT4G19680	7.0E-92	68%
BraA03g15110	AT4G19680	2.0E-92	82%
BraA03g15100	AT4G19700	4.0E-128	100%
BraA03g15090	AT4G19860	0.0E+00	100%
BraA03g15080	AT4G19880	0.0E+00	100%
BraA03g15070	AT4G19930	6.0E-118	100%
BraA03g15060	AT4G19960	6.0E-50	66%
BraA03g15050	AT4G29090	1.0E-38	65%
BraA03g15040	AT4G19930	6.0E-112	99%
BraA03g15030	AT4G19960	0.0E+00	100%
BraA03g15020	AT4G19960	0.0E+00	100%
BraA03g15010	AT4G19960	0.0E+00	100%
BraA03g15000	AT4G19970	2.0E-104	90%
BraA03g14990			
BraA03g14980	AT4G19970	8.0E-76	99%
BraA03g14970	AT4G20010	2.0E-153	99%
BraA03g14960	AT4G20020	4.0E-149	98%
BraA03g14950	AT4G20030	3.0E-33	80%
BraA03g14940	AT4G20040	0.0E+00	100%
BraA03g14930	AT4G20050	4.0E-57	28%
BraA03g14920			
BraA03g14910			
BraA03g14900			
BraA03g14890			
BraA03g14880	AT4G20270	2.0E-106	66%
BraA03g14870			
BraA03g14860			
BraA03g14850	AT4G20320	0.0E+00	97%
BraA03g14840			
BraA03g14830	AT4G20325	3.0E-149	100%
BraA03g14820	AT4G20360	0.0E+00	98%
BraA03g14810	AT4G20380	1.0E-70	99%
BraA03g14800	AT4G20420	8.0E-47	99%
BraA03g14790	AT4G20440	3.0E-40	91%
BraA03g14780	AT4G20780	8.0E-88	97%
BraA03g14770	AT4G20790	0.0E+00	100%

BraA03g14760	AT4G20800	2.0E-20	95%
BraA03g14750	AT4G20830	0.0E+00	84%
BraA03g14740			
BraA03g14730	AT4G20840	0.0E+00	100%
BraA03g14720			
BraA03g14710			
BraA03g14700			
BraA03g14690			
BraA03g14680			
BraA03g14670	AT4G20850	0.0E+00	100%
BraA03g14660	AT4G20880	1.0E-85	100%
BraA03g14650	AT4G20890	0.0E+00	97%
BraA03g14640	AT4G20900	0.0E+00	100%
BraA03g14630	AT4G20960	3.0E-61	95%
BraA03g14620	AT4G20960	6.0E-45	99%
BraA03g14610	AT4G20970	4.0E-65	98%
BraA03g14600			
BraA03g14590	AT4G20990	1.0E-118	99%
BraA03g14580	AT4G21050	5.0E-17	75%
BraA03g14570	AT4G21050	7.0E-59	84%
BraA03g14560			
BraA03g14550	AT4G21105	7.0E-36	29%
BraA03g14540	AT4G21110	5.0E-81	99%
BraA03g14530	AT4G21120	0.0E+00	99%
BraA03g14520	AT4G21150	0.0E+00	98%
BraA03g14510	AT4G21160	0.0E+00	99%
BraA03g14500	AT4G21190	6.0E-141	94%
BraA03g14490	AT4G21200	1.0E-87	82%
BraA03g14480	AT4G21215	7.0E-67	99%
BraA03g14470	AT4G21323	0.0E+00	70%
BraA03g14460	AT4G21326	0.0E+00	100%
BraA03g14450			
BraA03g14440	AT4G21410	4.0E-164	94%
BraA03g14430	AT4G21430	0.0E+00	100%
BraA03g14420			
BraA03g14410	AT4G21440	7.0E-99	96%
BraA03g14400	AT4G21445	1.0E-68	99%
BraA03g14390			
BraA03g14380	AT4G21540	0.0E+00	99%
BraA03g14370			
BraA03g14360	AT4G21650	0.0E+00	99%
BraA03g14350	AT4G21380	8.0E-06	57%
BraA03g14340	AT4G21380	1.0E-59	70%
BraA03g14330	AT4G21650	0.0E+00	99%
BraA03g14320	AT4G21670	0.0E+00	100%
BraA03g14310	AT4G21680	0.0E+00	98%
BraA03g14300			
BraA03g14290	AT4G21705	0.0E+00	100%
BraA03g14280	AT4G21710	0.0E+00	100%
BraA03g14270	AT4G21720	1.0E-50	99%
BraA03g14260	AT4G21740	3.0E-53	99%
BraA03g14250	AT4G21745	4.0E-54	99%
BraA03g14240	AT4G21750	0.0E+00	100%
BraA03g14230	AT4G21750	1.0E-125	100%
BraA03g14220	AT4G21760	0.0E+00	99%
BraA03g14210	AT4G21760	0.0E+00	98%
BraA03g14200	AT4G21790	9.0E-155	100%

BraA03g14190	AT4G21860	9.0E-88	56%
BraA03g14180	AT4G21895	3.0E-52	79%
BraA03g14170	AT4G21910	0.0E+00	100%
BraA03g14160	AT4G21903	3.0E-113	100%
BraA03g14150	AT4G16950	1.0E-39	53%
BraA03g14140			
BraA03g14130			
BraA03g14120	AT4G29090	1.0E-13	84%
BraA03g14110	AT4G16890	4.0E-49	96%
BraA03g14100			
BraA03g14090	AT4G16950	9.0E-63	70%
BraA03g14080	AT4G21970	3.0E-60	88%
BraA03g14070	AT4G21980	2.0E-64	95%
BraA03g14060	AT4G21990	0.0E+00	100%
BraA03g14050	AT4G22000	9.0E-08	57%
BraA03g14040	AT4G22180	2.0E-71	93%
BraA03g14030			
BraA03g14020			
BraA03g14010			
BraA03g14000	AT4G22165	5.0E-28	99%
BraA03g13990			
BraA03g13980			
BraA03g13970	AT4G22050	4.0E-48	90%
BraA03g13960	AT4G22030	3.0E-20	64%
BraA03g13950	AT4G22000	3.0E-08	30%
BraA03g13940	AT4G22060	4.0E-66	96%
BraA03g13930			
BraA03g13920			
BraA03g13910			
BraA03g13900	AT4G22115	2.0E-14	91%
BraA03g13890	AT4G29090	6.0E-18	87%
BraA03g13880			
BraA03g13870	AT4G22290	0.0E+00	100%
BraA03g13860			
BraA03g13850	AT4G22280	1.0E-25	54%
BraA03g13840			
BraA03g13830	AT4G22270	0.0E+00	98%
BraA03g13820	AT4G22250	8.0E-60	86%
BraA03g13810	AT4G29050	3.0E-23	76%
BraA03g13800	AT4G22190	2.0E-117	100%
BraA03g13790	AT4G22165	1.0E-32	71%
BraA03g13780	AT4G22180	2.0E-79	72%
BraA03g13770	AT4G22140	1.0E-128	72%
BraA03g13760	AT4G22115	1.0E-25	99%
BraA03g13750			
BraA03g13740			
BraA03g13730	AT4G23370	8.0E-10	72%
BraA03g13720			
BraA03g13710	AT4G29090	7.0E-21	88%
BraA03g13700	AT4G22115	2.0E-14	91%
BraA03g13690			
BraA03g13680			
BraA03g13670	AT4G22060	1.0E-67	47%
BraA03g13660	AT4G22000	2.0E-08	30%
BraA03g13650	AT4G22030	5.0E-14	89%
BraA03g13640	AT4G22050	1.0E-08	84%
BraA03g13630	AT4G22050	4.0E-33	66%

BraA03g13620			
BraA03g13610	AT4G22600	8.0E-37	69%
BraA03g13600	AT4G22600	1.0E-40	47%
BraA03g13590	AT4G22600	8.0E-44	66%
BraA03g13580	AT4G22590	0.0E+00	97%
BraA03g13570			
BraA03g13560	AT4G22620	5.0E-69	99%
BraA03g13550			
BraA03g13540	AT4G22680	4.0E-45	99%
BraA03g13530	AT4G22690	0.0E+00	100%
BraA03g13520	AT4G22700	6.0E-24	92%
BraA03g13510	AT4G22700	5.0E-07	79%
BraA03g13500	AT4G22700	2.0E-15	92%
BraA03g13490	AT4G22730	4.0E-41	38%
BraA03g13480	AT4G22700	2.0E-13	88%
BraA03g13470			
BraA03g13460	AT4G22740	1.0E-125	88%
BraA03g13450	AT4G22750	3.0E-138	100%
BraA03g13440			
BraA03g13430	AT4G23190	9.0E-103	69%
BraA03g13420	AT4G22756	8.0E-20	65%
BraA03g13410	AT4G22770	3.0E-143	100%
BraA03g13400			
BraA03g13390	AT4G22780	0.0E+00	100%
BraA03g13380	AT4G22790	0.0E+00	99%
BraA03g13370	AT4G22810	2.0E-102	100%
BraA03g13360	AT4G22820	2.0E-76	99%
BraA03g13350			
BraA03g13340			
BraA03g13330			
BraA03g13320	AT4G22840	0.0E+00	99%
BraA03g13310	AT4G22860	0.0E+00	100%
BraA03g13300	AT4G22880	0.0E+00	100%
BraA03g13290	AT4G22890	6.0E-170	100%
BraA03g13280	AT4G22900	1.0E-160	99%
BraA03g13270	AT4G22910	0.0E+00	100%
BraA03g13260	AT4G22920	6.0E-132	100%
BraA03g13250	AT4G23190	1.0E-102	100%
BraA03g13240	AT4G22756	3.0E-19	44%
BraA03g13230	AT4G22770	3.0E-143	100%
BraA03g13220			
BraA03g13210	AT4G22780	0.0E+00	100%
BraA03g13200	AT4G22790	0.0E+00	99%
BraA03g13190	AT4G22810	2.0E-102	100%
BraA03g13180	AT4G22820	2.0E-76	99%
BraA03g13170			
BraA03g13160			
BraA03g13150			
BraA03g13140	AT4G22840	0.0E+00	99%
BraA03g13130	AT4G22860	0.0E+00	100%
BraA03g13120	AT4G22880	0.0E+00	100%
BraA03g13110	AT4G22890	6.0E-170	100%
BraA03g13100	AT4G22900	1.0E-160	99%
BraA03g13090	AT4G22910	0.0E+00	100%
BraA03g13080	AT4G22920	6.0E-132	100%
BraA03g13070	AT4G22930	0.0E+00	70%
BraA03g13060	AT4G22950	1.0E-44	98%

BraA03g13050	AT4G22970	0.0E+00	96%
BraA03g13040	AT4G22970	0.0E+00	99%
BraA03g13030	AT4G23000	0.0E+00	49%
BraA03g13020	AT4G23010	0.0E+00	99%
BraA03g13010	AT4G23030	0.0E+00	98%
BraA03g13000	AT4G23050	0.0E+00	100%
BraA03g12990			
BraA03g12980	AT4G23060	0.0E+00	100%
BraA03g12970	AT4G23060	6.0E-45	59%
BraA03g12960			
BraA03g12950			
BraA03g12940			
BraA03g12930			
BraA03g12920			
BraA03g12910	AT4G23370	2.0E-15	56%
BraA03g12900	AT4G23080	2.0E-24	97%
BraA03g12890	AT4G23100	0.0E+00	98%
BraA03g12880	AT4G23100	0.0E+00	100%
BraA03g12870			
BraA03g12860			
BraA03g12850	AT4G23130	4.0E-111	99%
BraA03g12840			
BraA03g12830			
BraA03g12820	AT4G23130	3.0E-51	99%
BraA03g12810			
BraA03g12800	AT4G23160	3.0E-10	43%
BraA03g12790	AT4G23130	7.0E-26	78%
BraA03g12780	AT4G23130	1.0E-78	74%
BraA03g12770			
BraA03g12760			
BraA03g12750			
BraA03g12740	AT4G23160	6.0E-75	70%
BraA03g12730			
BraA03g12720			
BraA03g12710	AT4G23180	4.0E-100	93%
BraA03g12700	AT4G23160	2.0E-91	67%
BraA03g12690	AT4G23160	1.0E-101	72%
BraA03g12680	AT4G23290	3.0E-43	91%
BraA03g12670	AT4G23250	3.0E-45	90%
BraA03g12660	AT4G23210	6.0E-16	68%
BraA03g12650	AT4G23190	2.0E-97	58%
BraA03g12640	AT4G23190	2.0E-97	58%
BraA03g12630	AT4G23250	2.0E-104	76%
BraA03g12620	AT4G23250	2.0E-43	100%
BraA03g12610	AT4G23250	2.0E-94	100%
BraA03g12600	AT4G23190	2.0E-05	46%
BraA03g12590	AT4G23290	4.0E-77	99%
BraA03g12580	AT4G23220	3.0E-110	98%
BraA03g12570	AT4G23190	2.0E-167	96%
BraA03g12560	AT4G23330	8.0E-43	74%
BraA03g12550	AT4G23330	3.0E-04	7%
BraA03g12540	AT4G21240	3.0E-12	86%
BraA03g12530	AT4G23340	6.0E-165	100%
BraA03g12520	AT4G23370	5.0E-136	93%
BraA03g12510	AT4G23380	2.0E-169	95%
BraA03g12500	AT4G23390	1.0E-133	99%
BraA03g12490	AT4G23400	1.0E-156	100%

BraA03g12480	AT4G23410	3.0E-77	58%
BraA03g12470	AT4G23440	0.0E+00	100%
BraA03g12460	AT4G23450	4.0E-44	91%
BraA03g12450	AT4G22430	4.0E-10	63%
BraA03g12440			
BraA03g12430			
BraA03g12420	AT4G23460	0.0E+00	100%
BraA03g12410	AT4G23470	1.0E-121	93%
BraA03g12400	AT4G23490	0.0E+00	100%
BraA03g12390	AT4G23500	0.0E+00	99%
BraA03g12380	AT4G23515	3.0E-20	69%
BraA03g12370	AT4G23530	4.0E-138	100%
BraA03g12360	AT4G23550	3.0E-125	97%
BraA03g12350	AT4G23560	0.0E+00	99%
BraA03g12340			
BraA03g12330	AT4G23570	3.0E-164	100%
BraA03g12320	AT4G23530	4.0E-138	100%
BraA03g12310	AT4G23550	3.0E-125	97%
BraA03g12300	AT4G23560	0.0E+00	99%
BraA03g12290	AT4G23570	1.0E-165	100%
BraA03g12280	AT4G23590	0.0E+00	99%
BraA03g12270	AT4G23590	9.0E-151	97%
BraA03g12260			
BraA03g12250	AT4G23590	2.0E-160	99%
BraA03g12240			
BraA03g12230			
BraA03g12220			
BraA03g12210			
BraA03g12200	AT4G34460	2.0E-15	29%
BraA03g12190	AT4G23630	1.0E-130	100%
BraA03g12180	AT4G23650	3.0E-166	80%
BraA03g12170	AT4G23660	2.0E-161	100%
BraA03g12160	AT4G23680	2.0E-67	99%
BraA03g12150			
BraA03g12140			
BraA03g12130	AT4G23680	3.0E-19	64%
BraA03g12120	AT4G23690	2.0E-96	99%
BraA03g12110	AT4G23700	0.0E+00	100%
BraA03g12100	AT4G16890	3.0E-18	55%
BraA03g12090	AT4G23730	1.0E-147	99%
BraA03g12080	AT4G23740	0.0E+00	97%
BraA03g12070	AT4G23750	3.0E-111	99%
BraA03g12060	AT4G22756	3.0E-19	61%
BraA03g12050			
BraA03g12040			
BraA03g12030			
BraA03g12020	AT4G23790	0.0E+00	100%
BraA03g12010	AT4G23810	2.0E-128	100%
BraA03g12000	AT4G23820	0.0E+00	100%
BraA03g11990	AT4G23840	0.0E+00	97%
BraA03g11980	AT4G23870	2.0E-43	99%
BraA03g11970	AT4G23880	3.0E-77	100%
BraA03g11960	AT4G23885	7.0E-24	87%
BraA03g11950	AT4G23890	3.0E-113	100%
BraA03g11940	AT4G23895	2.0E-117	98%
BraA03g11930	AT4G23920	0.0E+00	97%
BraA03g11920	AT4G23930	7.0E-78	96%

BraA03g11910	AT4G23950	6.0E-22	75%
BraA03g11900	AT4G23980	0.0E+00	100%
BraA03g11890	AT4G23990	0.0E+00	100%
BraA03g11880	AT4G24015	3.0E-44	87%
BraA03g11870	AT4G24020	0.0E+00	100%
BraA03g11860	AT4G24026	4.0E-23	99%
BraA03g11850	AT4G24040	0.0E+00	99%
BraA03g11840			
BraA03g11830	AT4G24050	0.0E+00	100%
BraA03g11820	AT4G24060	5.0E-138	100%
BraA03g11810	AT4G24120	0.0E+00	100%
BraA03g11800	AT4G24130	2.0E-82	99%
BraA03g11790	AT4G24150	1.0E-153	95%
BraA03g11780	AT4G24160	0.0E+00	100%
BraA03g11770	AT4G24180	4.0E-126	100%
BraA03g11760	AT4G24190	0.0E+00	96%
BraA03g11750	AT4G24190	1.0E-77	95%
BraA03g11740	AT4G24204	2.0E-45	78%
BraA03g11730	AT4G24250	4.0E-51	88%
BraA03g11720	AT4G16500	1.0E-07	52%
BraA03g11710	AT4G24260	0.0E+00	98%
BraA03g11700			
BraA03g11690			
BraA03g11680			
BraA03g11670	AT4G24200	0.0E+00	97%
BraA03g11660	AT4G24270	3.0E-16	60%
BraA03g11650	AT4G24200	0.0E+00	87%
BraA03g11640	AT4G24280	0.0E+00	100%
BraA03g11630	AT4G20480	9.0E-16	41%
BraA03g11620	AT4G24330	0.0E+00	99%
BraA03g11610	AT4G24440	3.0E-58	99%
BraA03g11600	AT4G24470	2.0E-104	99%
BraA03g11590	AT4G26160	5.0E-10	54%
BraA03g11580	AT4G24490	2.0E-162	99%
BraA03g11570	AT4G24490	1.0E-150	98%
BraA03g11560	AT4G24500	2.0E-110	100%
BraA03g11550	AT4G24520	0.0E+00	100%
BraA03g11540	AT4G24530	4.0E-64	88%
BraA03g11530	AT4G24540	1.0E-66	99%
BraA03g11520	AT4G24550	7.0E-105	98%
BraA03g11510	AT4G24560	0.0E+00	100%
BraA03g11500	AT4G24570	1.0E-47	87%
BraA03g11490	AT4G24570	2.0E-30	99%
BraA03g11480	AT4G24580	0.0E+00	100%
BraA03g11470	AT4G24590	7.0E-97	100%
BraA03g11460	AT4G24610	0.0E+00	99%
BraA03g11450	AT4G24620	0.0E+00	100%
BraA03g11440	AT4G24680	0.0E+00	100%
BraA03g11430	AT4G24690	0.0E+00	100%
BraA03g11420	AT4G24700	2.0E-36	99%
BraA03g11410	AT4G24760	0.0E+00	100%
BraA03g11400	AT4G24770	9.0E-135	100%
BraA03g11390	AT4G24780	3.0E-35	68%
BraA03g11380	AT4G19130	6.0E-05	42%
BraA03g11370	AT4G24790	0.0E+00	100%
BraA03g11360	AT4G24800	3.0E-33	93%
BraA03g11350	AT4G24820	0.0E+00	100%

BraA03g11340	AT4G24830	0.0E+00	99%
BraA03g11330	AT4G24840	0.0E+00	100%
BraA03g11320	AT4G36070	1.0E-05	75%
BraA03g11310			
BraA03g11300	AT4G24880	0.0E+00	100%
BraA03g11290	AT4G24910	3.0E-132	99%
BraA03g11280	AT4G24920	2.0E-34	99%
BraA03g11270			
BraA03g11260	AT4G24960	1.0E-62	99%
BraA03g11250	AT4G24970	0.0E+00	99%
BraA03g11240	AT4G24990	3.0E-55	99%
BraA03g11230	AT4G25010	8.0E-138	97%
BraA03g11220			
BraA03g11210	AT4G25030	2.0E-165	100%
BraA03g11200	AT4G25030	5.0E-16	23%
BraA03g11190	AT4G25040	1.0E-74	99%
BraA03g11180	AT4G25050	2.0E-57	99%
BraA03g11170	AT4G25080	5.0E-166	100%
BraA03g11160	AT4G25090	0.0E+00	100%
BraA03g11150			
BraA03g11140	AT4G25080	8.0E-27	36%
BraA03g11130	AT4G25090	0.0E+00	99%
BraA03g11120	AT4G25120	5.0E-123	100%
BraA03g11110	AT4G25120	0.0E+00	100%
BraA03g11100	AT4G25130	2.0E-124	100%
BraA03g11090	AT4G25140	1.0E-74	98%
BraA03g11080	AT4G25160	0.0E+00	97%
BraA03g11070			
BraA03g11060	AT4G25190	1.0E-162	97%
BraA03g11050	AT4G25210	9.0E-87	76%
BraA03g11040	AT4G25225	7.0E-23	96%
BraA03g11030	AT4G25230	0.0E+00	100%
BraA03g11020			
BraA03g11010	AT4G25250	2.0E-87	100%
BraA03g11000	AT4G25260	1.0E-94	100%
BraA03g10990	AT4G25290	0.0E+00	98%
BraA03g10980	AT4G25300	8.0E-85	87%
BraA03g10970	AT4G25315	3.0E-50	98%
BraA03g10960			
BraA03g10950	AT4G25320	8.0E-161	90%
BraA03g10940			
BraA03g10930	AT4G25330	2.0E-47	91%
BraA03g10920	AT4G25340	1.0E-156	100%
BraA03g10910	AT4G25360	0.0E+00	98%
BraA03g10900	AT4G26340	2.0E-27	68%
BraA03g10890	AT4G25370	6.0E-107	99%
BraA03g10880	AT4G25380	2.0E-50	97%
BraA03g10870	AT4G25390	0.0E+00	100%
BraA03g10860	AT4G25400	1.0E-62	84%
BraA03g10850	AT4G25410	8.0E-82	99%
BraA03g10840			
BraA03g10830			
BraA03g10820			
BraA03g10810			
BraA03g10800			
BraA03g10790	AT4G25420	3.0E-180	99%
BraA03g10780	AT4G19990	6.0E-11	34%

BraA03g10770	AT4G29090	2.0E-05	24%
BraA03g10760	AT4G22950	2.0E-12	71%
BraA03g10750			
BraA03g10740	AT4G25470	2.0E-99	100%
BraA03g10730			
BraA03g10720	AT4G25520	0.0E+00	100%
BraA03g10710	AT4G25515	1.0E-130	73%
BraA03g10700			
BraA03g10690			
BraA03g10680	AT4G25540	0.0E+00	99%
BraA03g10670	AT4G25550	6.0E-116	100%
BraA03g10660	AT4G25560	6.0E-109	98%
BraA03g10650			
BraA03g10640			
BraA03g10630	AT4G25570	3.0E-112	100%
BraA03g10620	AT4G25580	6.0E-148	100%
BraA03g10610	AT4G25590	7.0E-71	99%
BraA03g10600	AT4G25600	4.0E-123	100%
BraA03g10590	AT4G25610	0.0E+00	88%
BraA03g10580	AT4G25620	8.0E-154	99%
BraA03g10570	AT4G25630	3.0E-138	80%
BraA03g10560	AT4G25640	0.0E+00	100%
BraA03g10550	AT4G25670	4.0E-78	99%
BraA03g10540	AT4G25820	8.0E-136	100%
BraA03g10530	AT4G25810	2.0E-28	94%
BraA03g10520			
BraA03g10510	AT4G25760	1.0E-53	90%
BraA03g10500			
BraA03g10490	AT4G25740	6.0E-75	99%
BraA03g10480	AT4G25730	0.0E+00	99%
BraA03g10470	AT4G25700	1.0E-154	99%
BraA03g10460			
BraA03g10450			
BraA03g10440	AT4G25990	3.0E-110	100%
BraA03g10430	AT4G25960	2.0E-162	82%
BraA03g10420	AT4G25960	0.0E+00	92%
BraA03g10410			
BraA03g10400	AT4G25950	2.0E-51	99%
BraA03g10390	AT4G25880	0.0E+00	99%
BraA03g10380	AT4G25835	0.0E+00	100%
BraA03g10370	AT4G25870	0.0E+00	100%
BraA03g10360	AT4G25820	9.0E-41	99%
BraA03g10350	AT4G25835	0.0E+00	100%
BraA03g10340	AT4G25840	6.0E-142	100%
BraA03g10330	AT4G25870	0.0E+00	100%
BraA03g10320	AT4G25880	0.0E+00	100%
BraA03g10310	AT4G25950	2.0E-51	99%
BraA03g10300			
BraA03g10290	AT4G25960	0.0E+00	92%
BraA03g10280	AT4G25990	3.0E-110	100%
BraA03g10270	AT4G26000	0.0E+00	100%
BraA03g10260	AT4G26010	4.0E-159	99%
BraA03g10250	AT4G26010	3.0E-140	99%
BraA03g10240	AT4G26020	1.0E-120	100%
BraA03g10230	AT4G26030	2.0E-13	72%
BraA03g10220	AT4G26050	6.0E-30	91%
BraA03g10210	AT4G26070	3.0E-159	86%

BraA03g10200	AT4G26080	0.0E+00	100%
BraA03g10190	AT4G26140	0.0E+00	100%
BraA03g10180			
BraA03g10170	AT4G26150	6.0E-105	100%
BraA03g10160	AT4G26200	0.0E+00	99%
BraA03g10150	AT4G26240	1.0E-96	100%
BraA03g10140	AT4G26210	1.0E-12	5%
BraA03g10130	AT4G26260	5.0E-154	100%
BraA03g10120	AT4G26280	2.0E-142	97%
BraA03g10110	AT4G26300	0.0E+00	94%
BraA03g10100	AT4G26320	2.0E-16	98%
BraA03g10090			
BraA03g10080	AT4G26330	0.0E+00	100%
BraA03g10070	AT4G26340	2.0E-28	70%
BraA03g10060	AT4G26370	3.0E-139	100%
BraA03g10050	AT4G26400	3.0E-111	92%
BraA03g10040	AT4G26410	3.0E-115	100%
BraA03g10030			
BraA03g10020	AT4G26430	5.0E-163	100%
BraA03g10010	AT4G26440	2.0E-113	62%
BraA03g10000			
BraA03g09990	AT4G26450	0.0E+00	100%
BraA03g09980	AT4G26466	5.0E-48	83%
BraA03g09970	AT4G26470	9.0E-111	93%
BraA03g09960	AT4G26480	6.0E-158	92%
BraA03g09950	AT4G26550	5.0E-110	50%
BraA03g09940	AT4G26555	8.0E-96	100%
BraA03g09930	AT4G26570	3.0E-128	100%
BraA03g09920			
BraA03g09910	AT4G26240	2.0E-76	83%
BraA03g09900	AT4G26200	0.0E+00	99%
BraA03g09890	AT4G26150	2.0E-95	98%
BraA03g09880	AT4G26370	7.0E-132	100%
BraA03g09870	AT4G26400	1.0E-112	92%
BraA03g09860	AT4G26410	7.0E-104	100%
BraA03g09850			
BraA03g09840	AT4G26430	4.0E-161	100%
BraA03g09830	AT4G26440	0.0E+00	100%
BraA03g09820			
BraA03g09810			
BraA03g09800	AT4G26450	0.0E+00	100%
BraA03g09790	AT4G26455	6.0E-11	91%
BraA03g09780	AT4G26466	5.0E-48	83%
BraA03g09770	AT4G26470	1.0E-110	93%
BraA03g09760	AT4G26480	7.0E-158	93%
BraA03g09750	AT4G26550	8.0E-117	100%
BraA03g09740	AT4G26555	1.0E-96	85%
BraA03g09730	AT4G26570	3.0E-128	100%
BraA03g09720	AT4G26590	0.0E+00	100%
BraA03g09710			
BraA03g09700	AT4G26640	2.0E-100	66%
BraA03g09690	AT4G26650	0.0E+00	100%
BraA03g09680	AT4G26690	0.0E+00	100%
BraA03g09670			
BraA03g09660	AT4G26730	4.0E-80	99%
BraA03g09650	AT4G26720	1.0E-178	100%
BraA03g09640	AT4G26710	2.0E-32	99%

BraA03g09630			
BraA03g09620	AT4G27500	3.0E-14	90%
BraA03g09610	AT4G27950	2.0E-12	35%
BraA03g09600	AT4G27950	2.0E-05	48%
BraA03g09590			
BraA03g09580	AT4G27950	5.0E-09	59%
BraA03g09570	AT5G15070	2.0E-18	94%
BraA03g09560	AT4G26780	6.0E-130	100%
BraA03g09550	AT4G26820	1.0E-72	81%
BraA03g09540	AT4G26830	0.0E+00	99%
BraA03g09530			
BraA03g09520			
BraA03g09510	AT4G26840	5.0E-49	89%
BraA03g09500	AT4G26850	0.0E+00	98%
BraA03g09490	AT4G26940	3.0E-161	100%
BraA03g09480	AT4G26950	3.0E-31	99%
BraA03g09470	AT4G26980	4.0E-151	98%
BraA03g09460	AT4G26990	7.0E-152	100%
BraA03g09450	AT4G27000	1.0E-161	93%
BraA03g09440	AT4G27030	2.0E-119	99%
BraA03g09430	AT4G27040	1.0E-141	100%
BraA03g09420	AT4G27070	0.0E+00	100%
BraA03g09410	AT4G26690	0.0E+00	53%
BraA03g09400	AT4G27070	3.0E-31	94%
BraA03g09390			
BraA03g09380	AT4G27080	0.0E+00	100%
BraA03g09370	AT4G27090	2.0E-70	99%
BraA03g09360	AT4G27120	6.0E-115	100%
BraA03g09350	AT4G27130	3.0E-63	99%
BraA03g09340	AT4G27170	5.0E-57	99%
BraA03g09330	AT4G18070	1.0E-47	74%
BraA03g09320			
BraA03g09310	AT4G27160	7.0E-57	99%
BraA03g09300			
BraA03g09290	AT4G27190	0.0E+00	96%
BraA03g09280	AT4G27250	3.0E-179	100%
BraA03g09270	AT4G27260	0.0E+00	100%
BraA03g09260			
BraA03g09250	AT4G27290	0.0E+00	83%
BraA03g09240	AT4G27310	6.0E-57	96%
BraA03g09230	AT4G27320	1.0E-82	100%
BraA03g09220	AT4G27330	2.0E-63	100%
BraA03g09210			
BraA03g09200	AT4G27390	6.0E-100	99%
BraA03g09190	AT4G27400	2.0E-154	100%
BraA03g09180	AT4G27410	6.0E-73	55%
BraA03g09170	AT4G27420	0.0E+00	99%
BraA03g09160			
BraA03g09150	AT4G27435	1.0E-92	99%
BraA03g09140	AT4G27440	6.0E-179	99%
BraA03g09130	AT4G27470	5.0E-99	94%
BraA03g09120	AT4G27480	0.0E+00	100%
BraA03g09110	AT4G27490	1.0E-17	79%
BraA03g09100	AT4G27500	0.0E+00	97%
BraA03g09090	AT4G27520	5.0E-13	55%
BraA03g09080	AT4G27540	2.0E-14	41%
BraA03g09070	AT4G27550	0.0E+00	100%

BraA03g09060	AT4G27580	1.0E-36	99%
BraA03g09050	AT4G16195	2.0E-05	86%
BraA03g09040	AT4G27600	0.0E+00	99%
BraA03g09030	AT4G27610	3.0E-148	100%
BraA03g09020	AT4G27640	0.0E+00	100%
BraA03g09010			
BraA03g09000			
BraA03g08990	AT4G27650	0.0E+00	100%
BraA03g08980	AT4G27657	4.0E-22	38%
BraA03g08970			
BraA03g08960			
BraA03g08950	AT4G27660	3.0E-54	96%
BraA03g08940	AT4G35160	3.0E-38	85%
BraA03g08930			
BraA03g08920	AT4G27680	0.0E+00	100%
BraA03g08910	AT4G27745	6.0E-38	71%
BraA03g08900	AT4G27760	0.0E+00	84%
BraA03g08890	AT4G27780	5.0E-93	98%
BraA03g08880	AT4G27800	2.0E-118	100%
BraA03g08870	AT4G27870	4.0E-145	99%
BraA03g08860	AT4G27870	8.0E-93	80%
BraA03g08850	AT4G27880	7.0E-09	80%
BraA03g08840			
BraA03g08830	AT4G27890	4.0E-77	92%
BraA03g08820	AT4G27890	1.0E-93	100%
BraA03g08810	AT4G27910	0.0E+00	100%
BraA03g08800	AT4G27950	8.0E-121	100%
BraA03g08790	AT4G27970	0.0E+00	100%
BraA03g08780	AT4G27980	7.0E-12	46%
BraA03g08770	AT4G32200	3.0E-20	22%
BraA03g08760			
BraA03g08750	AT4G27980	7.0E-132	99%
BraA03g08740	AT4G28000	0.0E+00	100%
BraA03g08730	AT4G28025	3.0E-59	99%
BraA03g08720			
BraA03g08710			
BraA03g08700	AT4G28030	3.0E-99	98%
BraA03g08690	AT4G28050	2.0E-139	99%
BraA03g08680	AT4G28060	5.0E-38	99%
BraA03g08670	AT4G28080	0.0E+00	100%
BraA03g08660			
BraA03g08650	AT4G18870	2.0E-18	77%
BraA03g08640			
BraA03g08630			
BraA03g08620			
BraA03g08610			
BraA03g08600			
BraA03g08590			
BraA03g08580	AT4G28100	7.0E-08	24%
BraA03g08570	AT4G28110	2.0E-36	65%
BraA03g08560	AT4G28130	0.0E+00	95%
BraA03g08550			
BraA03g08540	AT4G28140	2.0E-72	99%
BraA03g08530	AT4G28150	1.0E-142	89%
BraA03g08520	AT4G28160	6.0E-32	94%
BraA03g08510	AT4G28180	5.0E-13	31%
BraA03g08500	AT4G28190	2.0E-119	100%

BraA03g08490	AT4G28210	9.0E-160	96%
BraA03g08480	AT4G28220	0.0E+00	100%
BraA03g08470	AT4G28240	9.0E-37	99%
BraA03g08460	AT4G28250	2.0E-142	100%
BraA03g08450	AT4G28260	0.0E+00	93%
BraA03g08440	AT4G28270	1.0E-89	99%
BraA03g08430	AT4G28280	2.0E-53	71%
BraA03g08420	AT4G28300	0.0E+00	100%
BraA03g08410	AT4G28310	1.0E-63	95%
BraA03g08400	AT4G32480	6.0E-140	100%
BraA03g08390	AT4G28365	4.0E-07	41%
BraA03g08380			
BraA03g08370			
BraA03g08360			
BraA03g08350			
BraA03g08340	AT4G28395	4.0E-54	99%
BraA03g08330			
BraA03g08320			
BraA03g08310	AT4G28400	2.0E-149	100%
BraA03g08300	AT4G28410	2.0E-39	86%
BraA03g08290	AT4G28410	4.0E-43	84%
BraA03g08280	AT4G28410	4.0E-66	74%
BraA03g08270	AT4G28410	0.0E+00	100%
BraA03g08260	AT4G28440	2.0E-68	91%
BraA03g08250	AT4G28450	0.0E+00	100%
BraA03g08240	AT4G28460	6.0E-31	99%
BraA03g08230	AT4G28470	0.0E+00	100%
BraA03g08220	AT4G28480	3.0E-152	100%
BraA03g08210	AT4G18425	3.0E-49	89%
BraA03g08200	AT4G28500	1.0E-131	92%
BraA03g08190	AT4G28890	0.0E+00	100%
BraA03g08180			
BraA03g08170	AT4G28880	3.0E-90	82%
BraA03g08160	AT4G28840	1.0E-71	98%
BraA03g08150			
BraA03g08140	AT4G19870	2.0E-49	80%
BraA03g08130	AT4G28700	0.0E+00	98%
BraA03g08120	AT4G28660	3.0E-89	99%
BraA03g08110			
BraA03g08100	AT4G28640	4.0E-94	100%
BraA03g08090			
BraA03g08080	AT4G28610	3.0E-109	99%
BraA03g08070			
BraA03g08060	AT4G28570	0.0E+00	99%
BraA03g08050	AT4G28540	7.0E-77	70%
BraA03g08040	AT4G31900	3.0E-65	41%
BraA03g08030			
BraA03g08020	AT4G19870	2.0E-46	84%
BraA03g08010			
BraA03g08000	AT4G29370	3.0E-26	90%
BraA03g07990			
BraA03g07980	AT4G19870	4.0E-21	92%
BraA03g07970	AT4G28730	3.0E-69	99%
BraA03g07960	AT4G28740	2.0E-164	100%
BraA03g07950	AT4G28775	6.0E-40	66%
BraA03g07940	AT4G19540	4.0E-48	47%
BraA03g07930	AT4G28775	7.0E-41	86%

BraA03g07920	AT4G28820	1.0E-53	98%
BraA03g07910	AT4G28830	6.0E-90	99%
BraA03g07900			
BraA03g07890	AT4G28840	1.0E-65	90%
BraA03g07880	AT4G28880	2.0E-90	72%
BraA03g07870	AT4G28890	0.0E+00	100%
BraA03g07860	AT4G28910	4.0E-166	100%
BraA03g07850	AT4G29760	3.0E-22	67%
BraA03g07840	AT4G28920	1.0E-52	69%
BraA03g07830	AT4G28940	1.0E-55	97%
BraA03g07820			
BraA03g07810	AT4G28990	3.0E-08	53%
BraA03g07800			
BraA03g07790			
BraA03g07780	AT4G28990	2.0E-22	74%
BraA03g07770	AT4G29000	0.0E+00	99%
BraA03g07760	AT4G29010	0.0E+00	100%
BraA03g07750			
BraA03g07740	AT4G29020	4.0E-15	30%
BraA03g07730	AT4G29030	9.0E-11	44%
BraA03g07720	AT4G29060	5.0E-26	67%
BraA03g07710	AT4G29040	3.0E-146	89%
BraA03g07700	AT4G29040	1.0E-06	10%
BraA03g07690	AT4G36840	4.0E-08	50%
BraA03g07680	AT4G16295	2.0E-55	98%
BraA03g07670			
BraA03g07660	AT4G29140	0.0E+00	96%
BraA03g07650	AT4G29150	2.0E-174	100%
BraA03g07640			
BraA03g07630	AT4G29160	5.0E-91	100%
BraA03g07620	AT4G29170	2.0E-123	100%
BraA03g07610	AT4G29180	0.0E+00	100%
BraA03g07600	AT4G29180	6.0E-45	99%
BraA03g07590	AT4G29190	6.0E-54	69%
BraA03g07580			
BraA03g07570			
BraA03g07560	AT4G29210	1.0E-146	90%
BraA03g07550	AT4G29210	1.0E-130	99%
BraA03g07540	AT4G29210	0.0E+00	100%
BraA03g07530	AT4G29230	0.0E+00	100%
BraA03g07520	AT4G29240	1.0E-175	85%
BraA03g07510	AT4G29260	1.0E-122	100%
BraA03g07500	AT4G29240	1.0E-175	85%
BraA03g07490	AT4G29260	1.0E-122	100%
BraA03g07480	AT4G16835	4.0E-64	86%
BraA03g07470	AT4G29340	2.0E-73	99%
BraA03g07460	AT4G19870	8.0E-56	89%
BraA03g07450	AT4G29370	1.0E-106	100%
BraA03g07440	AT4G29410	5.0E-73	99%
BraA03g07430	AT4G29510	0.0E+00	100%
BraA03g07420	AT4G29590	2.0E-157	99%
BraA03g07410	AT4G29610	9.0E-82	94%
BraA03g07400	AT4G29610	2.0E-84	95%
BraA03g07390	AT4G29670	5.0E-99	100%
BraA03g07380	AT4G29680	0.0E+00	100%
BraA03g07370			
BraA03g07360	AT4G29720	0.0E+00	100%

BraA03g07350	AT4G29735	3.0E-26	99%
BraA03g07340	AT4G29740	0.0E+00	94%
BraA03g07330	AT4G31910	7.0E-27	81%
BraA03g07320			
BraA03g07310			
BraA03g07300	AT4G31940	1.0E-109	92%
BraA03g07290	AT4G31910	3.0E-27	81%
BraA03g07280	AT4G29750	0.0E+00	98%
BraA03g07270	AT4G31910	1.0E-26	73%
BraA03g07260			
BraA03g07250	AT4G26380	1.0E-58	86%
BraA03g07240			
BraA03g07230	AT4G26380	4.0E-72	89%
BraA03g07220	AT4G29780	0.0E+00	100%
BraA03g07210	AT4G29790	0.0E+00	80%
BraA03g07200	AT4G19870	1.0E-44	72%
BraA03g07190			
BraA03g07180	AT4G30070	1.0E-36	99%
BraA03g07170			
BraA03g07160	AT4G29840	2.0E-39	95%
BraA03g07150	AT4G19870	6.0E-37	97%
BraA03g07140			
BraA03g07130	AT4G29850	1.0E-54	99%
BraA03g07120			
BraA03g07110	AT4G29880	6.0E-172	100%
BraA03g07100	AT4G29890	2.0E-178	91%
BraA03g07090	AT4G29900	0.0E+00	99%
BraA03g07080	AT2G07190	6.0E-31	36%
BraA03g07070	AT4G29905	8.0E-25	92%
BraA03g07060	AT4G29920	2.0E-21	54%
BraA03g07050	AT4G29930	3.0E-109	96%
BraA03g07040	AT4G29960	2.0E-41	69%
BraA03g07030	AT4G29980	6.0E-60	85%
BraA03g07020	AT4G30010	1.0E-42	99%
BraA03g07010	AT4G30020	0.0E+00	100%
BraA03g07000	AT4G30060	0.0E+00	98%
BraA03g06990	AT4G30070	2.0E-34	99%
BraA03g06980	AT4G30070	2.0E-35	99%
BraA03g06970	AT4G30074	2.0E-29	98%
BraA03g06960			
BraA03g06950	AT4G30080	0.0E+00	100%
BraA03g06940	AT4G30100	0.0E+00	100%
BraA03g06930	AT4G30110	0.0E+00	99%
BraA03g06920	AT4G17530	1.0E-32	56%
BraA03g06910			
BraA03g06900	AT4G30160	0.0E+00	100%
BraA03g06890			
BraA03g06880	AT4G30120	7.0E-130	94%
BraA03g06870	AT4G30110	0.0E+00	97%
BraA03g06860	AT4G30170	3.0E-168	100%
BraA03g06850	AT4G30190	0.0E+00	100%
BraA03g06840	AT4G30190	0.0E+00	100%
BraA03g06830			
BraA03g06820	AT4G29980	2.0E-25	90%
BraA03g06810			
BraA03g06800			
BraA03g06790			

BraA03g06780	AT4G30200	0.0E+00	97%
BraA03g06770	AT4G30210	0.0E+00	99%
BraA03g06760	AT4G29980	8.0E-13	60%
BraA03g06750	AT4G30220	1.0E-41	72%
BraA03g06740	AT4G30220	2.0E-46	99%
BraA03g06730			
BraA03g06720	AT4G30260	1.0E-44	52%
BraA03g06710	AT4G30270	7.0E-146	100%
BraA03g06700	AT4G30280	1.0E-136	100%
BraA03g06690	AT4G30280	1.0E-139	100%
BraA03g06680	AT4G30310	0.0E+00	50%
BraA03g06670	AT4G30320	3.0E-76	94%
BraA03g06660			
BraA03g06650	AT4G30350	0.0E+00	100%
BraA03g06640	AT4G30360	6.0E-24	88%
BraA03g06630	AT4G30360	8.0E-45	67%
BraA03g06620	AT4G30400	2.0E-178	100%
BraA03g06610	AT4G30420	8.0E-23	72%
BraA03g06600	AT4G26340	1.0E-20	96%
BraA03g06590	AT4G30420	4.0E-160	97%
BraA03g06580	AT4G30420	3.0E-30	99%
BraA03g06570	AT4G30420	3.0E-150	97%
BraA03g06560	AT4G30440	0.0E+00	100%
BraA03g06550	AT4G30450	2.0E-07	37%
BraA03g06540			
BraA03g06530	AT4G30460	4.0E-10	23%
BraA03g06520	AT4G30470	3.0E-166	100%
BraA03g06510	AT4G30480	4.0E-17	40%
BraA03g06500	AT4G30490	0.0E+00	100%
BraA03g06490	AT4G30500	5.0E-84	99%
BraA03g06480	AT4G30510	9.0E-171	84%
BraA03g06470	AT4G30520	0.0E+00	99%
BraA03g06460	AT4G30530	8.0E-131	100%
BraA03g06450			
BraA03g06440	AT4G30560	0.0E+00	100%
BraA03g06430	AT4G30570	1.0E-141	42%
BraA03g06420	AT4G30610	0.0E+00	100%
BraA03g06410	AT4G30630	5.0E-49	98%
BraA03g06400	AT4G30640	1.0E-87	99%
BraA03g06390	AT4G18250	5.0E-27	94%
BraA03g06380			
BraA03g06370	AT4G18250	2.0E-87	42%
BraA03g06360	AT4G30640	7.0E-90	90%
BraA03g06350	AT4G30640	2.0E-91	98%
BraA03g06340	AT4G30660	3.0E-32	95%
BraA03g06330	AT4G30670	1.0E-17	69%
BraA03g06320	AT4G30680	4.0E-91	29%
BraA03g06310			
BraA03g06300	AT4G30690	2.0E-124	100%
BraA03g06290	AT4G30700	0.0E+00	100%
BraA03g06280	AT4G30710	0.0E+00	100%
BraA03g06270	AT4G30720	0.0E+00	100%
BraA03g06260	AT4G30770	5.0E-70	99%
BraA03g06250	AT4G30780	0.0E+00	99%
BraA03g06240	AT4G30790	0.0E+00	100%
BraA03g06230			
BraA03g06220	AT4G30810	0.0E+00	100%

BraA03g06210	AT4G30820	5.0E-75	99%
BraA03g06200	AT4G30830	4.0E-147	100%
BraA03g06190	AT4G30840	0.0E+00	99%
BraA03g06180	AT4G30845	3.0E-45	99%
BraA03g06170	AT4G30850	0.0E+00	100%
BraA03g06160	AT4G30890	0.0E+00	99%
BraA03g06150	AT4G30930	1.0E-105	100%
BraA03g06140	AT4G30935	2.0E-120	61%
BraA03g06130			
BraA03g06120			
BraA03g06110			
BraA03g06100	AT4G33870	5.0E-117	89%
BraA03g06090			
BraA03g06080	AT4G30960	0.0E+00	100%
BraA03g06070			
BraA03g06060			
BraA03g06050			
BraA03g06040	AT4G27130	9.0E-39	64%
BraA03g06030	AT4G30990	0.0E+00	100%
BraA03g06020	AT4G30996	2.0E-50	86%
BraA03g06010	AT4G31020	2.0E-168	100%
BraA03g06000	AT4G31100	3.0E-17	73%
BraA03g05990	AT4G31100	4.0E-98	98%
BraA03g05980	AT4G31100	1.0E-93	91%
BraA03g05970	AT4G31070	8.0E-50	98%
BraA03g05960	AT4G31070	0.0E+00	97%
BraA03g05950	AT4G31115	2.0E-65	94%
BraA03g05940	AT4G31120	0.0E+00	100%
BraA03g05930	AT4G31140	0.0E+00	100%
BraA03g05920	AT4G31160	0.0E+00	98%
BraA03g05910	AT4G31170	0.0E+00	100%
BraA03g05900			
BraA03g05890	AT4G31180	0.0E+00	100%
BraA03g05880	AT4G31210	0.0E+00	79%
BraA03g05870	AT4G31230	0.0E+00	100%
BraA03g05860			
BraA03g05850	AT4G31300	3.0E-135	100%
BraA03g05840	AT4G31310	1.0E-79	98%
BraA03g05830	AT4G31320	6.0E-85	96%
BraA03g05820			
BraA03g05810	AT4G31330	8.0E-130	100%
BraA03g05800	AT4G31340	0.0E+00	96%
BraA03g05790	AT4G31370	1.0E-103	94%
BraA03g05780	AT4G31380	6.0E-58	99%
BraA03g05770			
BraA03g05760	AT4G31400	6.0E-153	99%
BraA03g05750			
BraA03g05740	AT4G31440	2.0E-31	93%
BraA03g05730	AT4G31450	1.0E-67	100%
BraA03g05720			
BraA03g05710			
BraA03g05700			
BraA03g05690	AT4G31440	1.0E-95	96%
BraA03g05680	AT4G31450	0.0E+00	99%
BraA03g05670	AT4G30074	6.0E-39	99%
BraA03g05660	AT4G31470	2.0E-82	99%
BraA03g05650	AT4G31480	0.0E+00	100%

BraA03g05640			
BraA03g05630			
BraA03g05620	AT4G31500	9.0E-108	97%
BraA03g05610	AT4G31530	2.0E-145	99%
BraA03g05600	AT4G31540	2.0E-35	37%
BraA03g05590	AT4G31550	1.0E-87	95%
BraA03g05580			
BraA03g05570			
BraA03g05560	AT4G31560	2.0E-57	99%
BraA03g05550			
BraA03g05540			
BraA03g05530	AT4G31570	0.0E+00	100%
BraA03g05520	AT4G31580	5.0E-26	95%
BraA03g05510	AT4G31590	0.0E+00	100%
BraA03g05500			
BraA03g05490	AT4G31630	8.0E-154	100%
BraA03g05480	AT4G31680	2.0E-59	86%
BraA03g05470	AT4G31710	1.0E-80	89%
BraA03g05460	AT4G31710	0.0E+00	99%
BraA03g05450	AT4G31620	4.0E-06	92%
BraA03g05440	AT4G31620	6.0E-39	92%
BraA03g05430	AT4G31730	4.0E-58	99%
BraA03g05420			
BraA03g05410			
BraA03g05400	AT4G31770	0.0E+00	83%
BraA03g05390	AT4G31780	0.0E+00	85%
BraA03g05380			
BraA03g05370	AT4G31800	1.0E-125	100%
BraA03g05360	AT4G31805	3.0E-102	99%
BraA03g05350			
BraA03g05340	AT4G31840	5.0E-74	99%
BraA03g05330	AT4G31850	0.0E+00	100%
BraA03g05320	AT4G31860	0.0E+00	100%
BraA03g05310	AT4G31870	4.0E-102	98%
BraA03g05300	AT4G31875	2.0E-11	36%
BraA03g05290			
BraA03g05280			
BraA03g05270			
BraA03g05260	AT4G31880	0.0E+00	42%
BraA03g05250	AT4G31910	0.0E+00	99%
BraA03g05240	AT4G31920	0.0E+00	97%
BraA03g05230	AT4G31930	4.0E-111	96%
BraA03g05220	AT4G31985	2.0E-23	89%
BraA03g05210	AT4G31990	0.0E+00	100%
BraA03g05200	AT4G32010	0.0E+00	100%
BraA03g05190	AT4G32020	3.0E-55	99%
BraA03g05180	AT4G32030	3.0E-55	99%
BraA03g05170	AT4G32040	0.0E+00	100%
BraA03g05160	AT4G32070	0.0E+00	100%
BraA03g05150	AT4G32090	1.0E-38	98%
BraA03g05140	AT4G32160	0.0E+00	100%
BraA03g05130	AT4G32160	2.0E-111	98%
BraA03g05120			
BraA03g05110	AT4G32270	3.0E-61	69%
BraA03g05100	AT4G32272	2.0E-82	68%
BraA03g05090	AT4G32280	9.0E-75	99%
BraA03g05080	AT4G32290	0.0E+00	100%

BraA03g05070	AT4G32295	9.0E-114	98%
BraA03g05060	AT4G32300	0.0E+00	77%
BraA03g05050	AT4G32320	1.0E-156	99%
BraA03g05040	AT4G32330	1.0E-148	99%
BraA03g05030	AT4G32410	0.0E+00	100%
BraA03g05020	AT4G32551	1.0E-13	76%
BraA03g05010	AT4G32551	3.0E-118	100%
BraA03g05000	AT4G32551	4.0E-21	98%
BraA03g04990	AT4G32610	5.0E-45	72%
BraA03g04980	AT4G32650	0.0E+00	96%
BraA03g04970			
BraA03g04960			
BraA03g04950	AT4G32730	0.0E+00	70%
BraA03g04940	AT4G32750	5.0E-65	89%
BraA03g04930	AT4G32760	0.0E+00	100%
BraA03g04920	AT4G32770	0.0E+00	100%
BraA03g04910			
BraA03g04900			
BraA03g04890	AT4G32860	4.0E-94	99%
BraA03g04880	AT4G32870	3.0E-66	99%
BraA03g04870	AT4G33000	2.0E-119	98%
BraA03g04860	AT4G33030	0.0E+00	99%
BraA03g04850	AT4G33040	5.0E-67	99%
BraA03g04840	AT4G33110	0.0E+00	100%
BraA03g04830	AT4G35280	2.0E-05	55%
BraA03g04820			
BraA03g04810			
BraA03g04800			
BraA03g04790	AT4G35810	1.0E-38	25%
BraA03g04780	AT4G33140	7.0E-145	100%
BraA03g04770	AT4G33150	0.0E+00	98%
BraA03g04760	AT4G33150	0.0E+00	99%
BraA03g04750			
BraA03g04740			
BraA03g04730			
BraA03g04720	AT4G32980	5.0E-180	99%
BraA03g04710	AT4G32960	4.0E-133	96%
BraA03g04700	AT4G32950	3.0E-161	99%
BraA03g04690	AT4G32930	2.0E-93	99%
BraA03g04680	AT4G32920	0.0E+00	100%
BraA03g04670	AT4G33160	4.0E-79	99%
BraA03g04660			
BraA03g04650	AT4G33180	3.0E-161	99%
BraA03g04640	AT4G33200	0.0E+00	100%
BraA03g04630			
BraA03g04620			
BraA03g04610			
BraA03g04600			
BraA03g04590	AT4G33210	0.0E+00	100%
BraA03g04580			
BraA03g04570	AT4G33230	0.0E+00	100%
BraA03g04560	AT4G33250	4.0E-120	100%
BraA03g04550	AT4G33270	0.0E+00	100%
BraA03g04540	AT4G33280	7.0E-133	99%
BraA03g04530	AT4G33350	6.0E-136	100%
BraA03g04520	AT4G33355	8.0E-48	99%
BraA03g04510	AT4G33360	3.0E-175	100%

BraA03g04500	AT4G33380	1.0E-09	58%
BraA03g04490	AT4G33390	0.0E+00	99%
BraA03g04480	AT4G33410	0.0E+00	99%
BraA03g04470	AT4G33420	3.0E-163	100%
BraA03g04460	AT4G33430	0.0E+00	100%
BraA03g04450	AT4G33440	0.0E+00	99%
BraA03g04440	AT4G33450	2.0E-28	99%
BraA03g04430			
BraA03g04420	AT4G33467	3.0E-32	99%
BraA03g04410	AT4G33565	9.0E-26	58%
BraA03g04400	AT4G33580	7.0E-144	99%
BraA03g04390	AT4G33590	0.0E+00	99%
BraA03g04380	AT4G33610	4.0E-16	99%
BraA03g04370	AT4G37390	7.0E-154	99%
BraA03g04360	AT4G37390	2.0E-148	97%
BraA03g04350	AT4G37390	2.0E-78	77%
BraA03g04340	AT4G26090	5.0E-109	92%
BraA03g04330	AT4G29750	4.0E-41	67%
BraA03g04320			
BraA03g04310	AT4G33160	3.0E-88	99%
BraA03g04300			
BraA03g04290	AT4G33180	3.0E-161	99%
BraA03g04280	AT4G33200	0.0E+00	100%
BraA03g04270			
BraA03g04260			
BraA03g04250	AT4G33210	0.0E+00	100%
BraA03g04240			
BraA03g04230	AT4G33230	0.0E+00	100%
BraA03g04220	AT4G33250	4.0E-120	100%
BraA03g04210	AT4G33270	0.0E+00	100%
BraA03g04200	AT4G33280	7.0E-133	99%
BraA03g04190	AT4G33350	2.0E-137	100%
BraA03g04180	AT4G33355	8.0E-48	99%
BraA03g04170	AT4G33360	3.0E-175	100%
BraA03g04160	AT4G33390	0.0E+00	83%
BraA03g04150	AT4G33410	0.0E+00	99%
BraA03g04140	AT4G33420	3.0E-166	100%
BraA03g04130	AT4G33430	0.0E+00	100%
BraA03g04120	AT4G33440	0.0E+00	99%
BraA03g04110	AT4G33450	6.0E-78	97%
BraA03g04100	AT4G33467	3.0E-32	99%
BraA03g04090	AT4G33565	3.0E-87	100%
BraA03g04080	AT4G33580	7.0E-144	99%
BraA03g04070	AT4G33590	0.0E+00	99%
BraA03g04060			
BraA03g04050	AT4G37390	7.0E-154	99%
BraA03g04040	AT4G26090	5.0E-109	92%
BraA03g04030	AT4G29750	3.0E-41	36%
BraA03g04020			
BraA03g04010	AT4G33625	8.0E-86	91%
BraA03g04000	AT4G33630	0.0E+00	100%
BraA03g03990	AT4G33650	0.0E+00	98%
BraA03g03980	AT4G33660	8.0E-05	99%
BraA03g03970	AT4G33670	2.0E-174	98%
BraA03g03960	AT4G33720	1.0E-73	99%
BraA03g03950			
BraA03g03940			

BraA03g03930			
BraA03g03920	AT4G36840	4.0E-26	70%
BraA03g03910	AT4G33720	1.0E-73	99%
BraA03g03900	AT4G33710	4.0E-52	89%
BraA03g03890			
BraA03g03880	AT4G33710	1.0E-54	99%
BraA03g03870	AT4G33720	7.0E-73	99%
BraA03g03860	AT4G33720	5.0E-74	99%
BraA03g03850	AT4G33720	6.0E-20	65%
BraA03g03840	AT4G33730	2.0E-04	19%
BraA03g03830	AT4G33740	7.0E-128	96%
BraA03g03820	AT4G33770	2.0E-171	99%
BraA03g03810			
BraA03g03800			
BraA03g03790	AT4G29990	2.0E-59	72%
BraA03g03780	AT4G33625	3.0E-39	86%
BraA03g03770	AT4G33630	2.0E-58	92%
BraA03g03760	AT4G33630	9.0E-19	39%
BraA03g03750			
BraA03g03740			
BraA03g03730			
BraA03g03720			
BraA03g03710			
BraA03g03700			
BraA03g03690			
BraA03g03680	AT4G20270	7.0E-19	43%
BraA03g03670			
BraA03g03660			
BraA03g03650			
BraA03g03640			
BraA03g03630			
BraA03g03620			
BraA03g03610			
BraA03g03600			
BraA03g03590			
BraA03g03580			
BraA03g03570			
BraA03g03560	AT4G21300	4.0E-05	29%
BraA03g03550	AT4G33920	0.0E+00	100%
BraA03g03540	AT4G33905	2.0E-35	47%
BraA03g03530			
BraA03g03520	AT4G34720	4.0E-88	99%
BraA03g03510	AT4G34730	9.0E-105	50%
BraA03g03500	AT4G34760	7.0E-57	99%
BraA03g03490			
BraA03g03480			
BraA03g03470			
BraA03g03460	AT4G34790	5.0E-17	94%
BraA03g03450	AT4G34850	0.0E+00	100%
BraA03g03440			
BraA03g03430	AT4G34970	2.0E-70	99%
BraA03g03420	AT4G34980	0.0E+00	100%
BraA03g03410			
BraA03g03400	AT4G35000	5.0E-150	100%
BraA03g03390	AT4G35010	0.0E+00	100%
BraA03g03380			
BraA03g03370	AT4G35500	6.0E-59	91%

BraA03g03360			
BraA03g03350	AT4G35070	1.0E-88	100%
BraA03g03340	AT4G34400	8.0E-21	36%
BraA03g03330	AT4G35090	0.0E+00	100%
BraA03g03320	AT4G35100	4.0E-157	100%
BraA03g03310	AT4G35120	3.0E-66	97%
BraA03g03300			
BraA03g03290	AT4G35160	0.0E+00	100%
BraA03g03280	AT4G35160	0.0E+00	100%
BraA03g03270	AT4G35170	2.0E-129	100%
BraA03g03260	AT4G35180	0.0E+00	99%
BraA03g03250			
BraA03g03240			
BraA03g03230	AT4G35200	1.0E-85	99%
BraA03g03220			
BraA03g03210			
BraA03g03200	AT4G34460	3.0E-04	26%
BraA03g03190			
BraA03g03180			
BraA03g03170	AT4G35270	0.0E+00	100%
BraA03g03160	AT4G35280	1.0E-121	100%
BraA03g03150	AT4G35310	6.0E-30	88%
BraA03g03140	AT4G35310	2.0E-83	99%
BraA03g03130	AT4G35335	0.0E+00	100%
BraA03g03120			
BraA03g03110			
BraA03g03100	AT4G35450	4.0E-160	100%
BraA03g03090	AT4G35460	1.0E-175	100%
BraA03g03080	AT4G35470	0.0E+00	100%
BraA03g03070	AT4G35480	1.0E-85	99%
BraA03g03060	AT4G35490	6.0E-76	99%
BraA03g03050	AT4G35490	8.0E-85	99%
BraA03g03040	AT4G35500	0.0E+00	100%
BraA03g03030	AT4G35510	6.0E-44	86%
BraA03g03020	AT4G35520	0.0E+00	100%
BraA03g03010	AT4G35530	7.0E-88	99%
BraA03g03000	AT4G35550	1.0E-88	96%
BraA03g02990			
BraA03g02980			
BraA03g02970			
BraA03g02960	AT4G35560	0.0E+00	100%
BraA03g02950	AT4G35570	1.0E-45	57%
BraA03g02940	AT4G35600	3.0E-65	95%
BraA03g02930	AT4G35600	1.0E-108	100%
BraA03g02920	AT4G35600	0.0E+00	100%
BraA03g02910			
BraA03g02900			
BraA03g02890			
BraA03g02880			
BraA03g02870	AT4G35620	4.0E-160	98%
BraA03g02860	AT4G35660	4.0E-114	99%
BraA03g02850	AT4G35670	5.0E-163	96%
BraA03g02840			
BraA03g02830			
BraA03g02820			
BraA03g02810	AT4G35690	9.0E-92	93%
BraA03g02800	AT4G35720	7.0E-147	100%

BraA03g02790			
BraA03g02780	AT4G35725	2.0E-16	94%
BraA03g02770			
BraA03g02760	AT4G35790	0.0E+00	100%
BraA03g02750			
BraA03g02740	AT4G35840	4.0E-123	100%
BraA03g02730	AT4G35860	6.0E-122	100%
BraA03g02720			
BraA03g02710	AT4G35890	7.0E-157	99%
BraA03g02700	AT4G35900	2.0E-74	74%
BraA03g02690	AT4G35905	3.0E-36	97%
BraA03g02680	AT4G35920	0.0E+00	100%
BraA03g02670	AT4G35930	1.0E-138	99%
BraA03g02660	AT4G35940	1.0E-56	69%
BraA03g02650	AT4G35950	5.0E-103	99%
BraA03g02640	AT4G35985	0.0E+00	100%
BraA03g02630	AT4G36020	9.0E-86	99%
BraA03g02620	AT4G36030	0.0E+00	100%
BraA03g02610			
BraA03g02600	AT4G36040	2.0E-67	99%
BraA03g02590	AT4G36060	1.0E-09	58%
BraA03g02580	AT4G36070	8.0E-45	100%
BraA03g02570			
BraA03g02560	AT4G36130	2.0E-147	100%
BraA03g02550	AT4G36160	1.0E-108	99%
BraA03g02540	AT4G36160	6.0E-30	85%
BraA03g02530	AT4G36195	0.0E+00	100%
BraA03g02520	AT4G36220	0.0E+00	100%
BraA03g02510			
BraA03g02500	AT4G36250	0.0E+00	100%
BraA03g02490	AT4G36260	1.0E-95	91%
BraA03g02480	AT4G36350	0.0E+00	100%
BraA03g02470	AT4G36360	1.0E-40	63%
BraA03g02460	AT4G36360	7.0E-92	99%
BraA03g02450	AT4G36360	0.0E+00	93%
BraA03g02440			
BraA03g02430	AT4G36380	0.0E+00	94%
BraA03g02420	AT4G36400	0.0E+00	85%
BraA03g02410	AT4G36410	3.0E-84	99%
BraA03g02400	AT4G36420	4.0E-57	99%
BraA03g02390	AT4G36430	9.0E-176	100%
BraA03g02380	AT4G36480	0.0E+00	100%
BraA03g02370	AT4G36470	2.0E-180	100%
BraA03g02360	AT4G36500	7.0E-58	99%
BraA03g02350	AT4G36540	5.0E-127	100%
BraA03g02340	AT4G36550	6.0E-78	48%
BraA03g02330	AT4G36620	5.0E-68	99%
BraA03g02320	AT4G36630	0.0E+00	100%
BraA03g02310	AT4G36640	9.0E-145	99%
BraA03g02300			
BraA03g02290	AT4G36650	0.0E+00	100%
BraA03g02280	AT4G36660	6.0E-77	99%
BraA03g02270	AT4G36670	0.0E+00	100%
BraA03g02260			
BraA03g02250	AT4G36700	2.0E-161	82%
BraA03g02240	AT4G36700	2.0E-12	57%
BraA03g02230	AT4G30360	1.0E-28	60%

BraA03g02220	AT4G36710	5.0E-85	80%
BraA03g02210			
BraA03g02200			
BraA03g02190			
BraA03g02180	AT4G36720	6.0E-46	70%
BraA03g02170			
BraA03g02160	AT4G27960	6.0E-10	14%
BraA03g02150	AT4G35120	5.0E-70	96%
BraA03g02140	AT4G36740	4.0E-95	100%
BraA03g02130	AT4G36750	2.0E-127	100%
BraA03g02120	AT4G36780	3.0E-74	60%
BraA03g02110			
BraA03g02100			
BraA03g02090	AT4G36800	5.0E-22	98%
BraA03g02080	AT4G37580	0.0E+00	100%
BraA03g02070			
BraA03g02060	AT4G37590	0.0E+00	100%
BraA03g02050	AT4G37608	9.0E-18	74%
BraA03g02040	AT4G37610	0.0E+00	100%
BraA03g02030	AT4G30070	2.0E-29	96%
BraA03g02020			
BraA03g02010			
BraA03g02000			
BraA03g01990			
BraA03g01980			
BraA03g01970	AT4G37630	6.0E-139	100%
BraA03g01960	AT4G37640	0.0E+00	100%
BraA03g01950			
BraA03g01940	AT4G37650	0.0E+00	100%
BraA03g01930	AT4G37660	2.0E-68	99%
BraA03g01920	AT4G37670	0.0E+00	88%
BraA03g01910			
BraA03g01900	AT4G37680	0.0E+00	100%
BraA03g01890			
BraA03g01880			
BraA03g01870			
BraA03g01860	AT4G37690	0.0E+00	100%
BraA03g01850	AT4G37700	4.0E-52	96%
BraA03g01840	AT4G37710	3.0E-27	99%
BraA03g01830	AT4G37720	5.0E-29	99%
BraA03g01820	AT4G37730	2.0E-91	94%
BraA03g01810	AT4G37740	2.0E-162	100%
BraA03g01800			
BraA03g01790	AT4G37750	0.0E+00	100%
BraA03g01780			
BraA03g01770			
BraA03g01760	AT4G37790	6.0E-120	100%
BraA03g01750			
BraA03g01740	AT4G37800	1.0E-167	100%
BraA03g01730			
BraA03g01720	AT4G37870	0.0E+00	100%
BraA03g01710			
BraA03g01700			
BraA03g01690	AT4G37890	0.0E+00	100%
BraA03g01680	AT4G37900	0.0E+00	89%
BraA03g01670			
BraA03g01660			

BraA03g01650
BraA03g01640
BraA03g01630
BraA03g01620
BraA03g01610
BraA03g01600
BraA03g01590
BraA03g01580
BraA03g01570
BraA03g01560
BraA03g01550
BraA03g01540
BraA03g01530
BraA03g01520
BraA03g01510
BraA03g01500
BraA03g01490
BraA03g01480
BraA03g01470
BraA03g01460
BraA03g01450
BraA03g01440
BraA03g01430
BraA03g01420
BraA03g01410
BraA03g01400
BraA03g01390
BraA03g01380
BraA03g01370
BraA03g01360
BraA03g01350
BraA03g01340
BraA03g01330
BraA03g01320
BraA03g01310
BraA03g01300
BraA03g01290
BraA03g01280
BraA03g01270
BraA03g01260
BraA03g01250
BraA03g01240
BraA03g01230
BraA03g01220
BraA03g01210
BraA03g01200
BraA03g01190
BraA03g01180
BraA03g01170
BraA03g01160
BraA03g01150
BraA03g01140
BraA03g01130
BraA03g01120
BraA03g01110
BraA03g01100
BraA03g01090

BraA03g01080
BraA03g01070
BraA03g01060
BraA03g01050
BraA03g01040
BraA03g01030
BraA03g01020
BraA03g01010
BraA03g01000
BraA03g00990
BraA03g00980
BraA03g00970
BraA03g00960
BraA03g00950
BraA03g00940
BraA03g00930
BraA03g00920
BraA03g00910
BraA03g00900
BraA03g00890
BraA03g00880
BraA03g00870
BraA03g00860
BraA03g00850
BraA03g00840
BraA03g00830
BraA03g00820
BraA03g00810
BraA03g00800
BraA03g00790
BraA03g00780
BraA03g00770
BraA03g00760
BraA03g00750
BraA03g00740
BraA03g00730
BraA03g00720
BraA03g00710
BraA03g00700
BraA03g00690
BraA03g00680
BraA03g00670
BraA03g00660
BraA03g00650
BraA03g00640
BraA03g00630
BraA03g00620
BraA03g00610
BraA03g00600
BraA03g00590
BraA03g00580
BraA03g00570
BraA03g00560
BraA03g00550
BraA03g00540
BraA03g00530
BraA03g00520

BraA03g00510
BraA03g00500
BraA03g00490
BraA03g00480
BraA03g00470
BraA03g00460
BraA03g00450
BraA03g00440
BraA03g00430
BraA03g00420
BraA03g00410
BraA03g00400
BraA03g00390
BraA03g00380
BraA03g00370
BraA03g00360
BraA03g00350
BraA03g00340
BraA03g00330
BraA03g00320
BraA03g00310
BraA03g00300
BraA03g00290
BraA03g00280
BraA03g00270
BraA03g00260
BraA03g00250
BraA03g00240
BraA03g00230
BraA03g00220
BraA03g00210
BraA03g00200
BraA03g00190
BraA03g00180
BraA03g00170
BraA03g00160
BraA03g00150
BraA03g00140
BraA03g00130
BraA03g00120
BraA03g00110
BraA03g00100
BraA03g00090
BraA03g00080
BraA03g00070
BraA03g00060
BraA03g00050
BraA03g00040
BraA03g00030
BraA03g00020
BraA03g00010
